# *S*-Ethyl-Isothiocitrullin-Based Dipeptides and 1,2,4-Oxadiazole Pseudo-Dipeptides: Solid Phase Synthesis and Evaluation as NO Synthase Inhibitors

**DOI:** 10.3390/molecules28135085

**Published:** 2023-06-29

**Authors:** Elodie Mauchauffée, Jérémy Leroy, Jihanne Chamcham, Abdelaziz Ejjoummany, Manon Maurel, Lionel Nauton, Booma Ramassamy, Karima Mezghenna, Jean-Luc Boucher, Anne-Dominique Lajoix, Jean-François Hernandez

**Affiliations:** 1 Institut des Biomolécules Max Mousseron, CNRS, Univ. Montpellier, ENSCM, Pôle Chimie Balard, 34293 Montpellier, France; 2 Centre Biocommunication en Cardio-Métabolique, Univ. Montpellier, UFR Pharmacie, 34093 Montpellier, France; 3 Institut de Chimie de Clermont-Ferrand, Université Clermont-Auvergne, CNRS, 63178 Aubière, France; 4 Laboratoire de Chimie et Biochimie Pharmacologiques et Toxicologiques, UMR 8601, CNRS, Université Paris Descartes, CEDEX 06, 75270 Paris, France

**Keywords:** NO synthases, enzyme inhibitors, pseudo-dipeptides, isothiocitrulline, 1,2,4-oxadiazole, solid phase synthesis

## Abstract

We previously reported dipeptidomimetic compounds as inhibitors of neuronal and/or inducible NO synthases (n/iNOS) with significant selectivity against endothelial NOS (eNOS). They were composed of an *S*-ethylisothiocitrullin-like moiety linked to an extension through a peptide bond or a 1,2,4-oxadiazole link. Here, we developed two further series where the extension size was increased to establish more favorable interactions in the NOS substrate access channel. The extension was introduced on the solid phase by the reductive alkylation of an amino-piperidine moiety or an aminoethyl segment in the case of dipeptide-like and 1,2,4-oxadiazole compounds, respectively, with various benzaldehydes. Compared to the previous series, more potent inhibitors were identified with IC_50_ in the micromolar to the submicromolar range, with significant selectivity toward nNOS. As expected, most compounds did not inhibit eNOS, and molecular modeling was carried out to characterize the reasons for the selectivity toward nNOS over eNOS. Spectral studies showed that compounds were interacting at the heme active site. Finally, selected inhibitors were found to inhibit intra-cellular iNOS and nNOS expressed in RAW264.7 and INS-1 cells, respectively.

## 1. Introduction

Nitric oxide (NO) is a widespread cellular signaling molecule involved in a wide range of physiological functions, which depend on its production localization [1,2]. Indeed, in mammals, NO is produced from *L*-Arginine (*L*-Arg) by three enzyme isoforms called nitric oxide synthases (NOS), which first differ by their localization and mode of regulation [3]. Two are constitutively expressed and are activated by increased intracellular calcium, leading to the production of low levels of NO (i.e., nanomolar), which will mainly activate the soluble guanylate cyclase. (i) The neuronal NOS (nNOS) is mainly present in the brain where NO acts as a neurotransmitter but also in pancreatic β-cells to control insulin secretion [4,5]. (ii) The endothelial NOS (eNOS) is present in the vessel endothelium and mainly contributes to smooth muscle relaxation, impacting blood pressure. The third NOS is the inducible NOS (iNOS). It is expressed by macrophages in response to various pro-inflammatory cytokines and is intrinsically active (i.e., no need for elevated calcium concentration), producing high NO levels (i.e., micromolar), which lead to the formation of toxic species, such as peroxynitrite or other radical species [6], making iNOS expression an important host defense mechanism [7]. Both NOS isoforms share the same structure organization with two domains separated by a calmodulin-binding sequence. (i) The *N*-terminal oxygenase domain contains the heme prosthetic group, a tetrahydrobiopterine co-factor and the substrate binding site, which is connected to the protein surface through the substrate access channel; (ii) the *C*-terminal reductase domain contains two flavine cofactors: FAD and FMN [3,8,9].

Overproduction of NO by nNOS and iNOS is associated with various pathophysiological states [2,10,11,12,13]. Overstimulation of nNOS is mainly involved in ischemia reperfusion injury following stroke [14,15] and is also shown to contribute to melanoma progression and metastasis [16]. In contrast, uncontrolled iNOS production is observed in numerous diseases, including sepsis, inflammation (e.g., rheumatoid arthritis), insulin resistance, some cancers, and asthma [5,17,18,19,20,21,22]. Thus, a potential therapeutical strategy is to develop NOS inhibitors [23], but one clinically useful inhibitor should be selective of the targeted isoform to spare the essential physiological roles of the two others. It is particularly true for eNOS, whose unwanted inhibition exposed to serious side effects. Unfortunately, obtaining such selectivity is a challenge because the substrate binding sites of the three isoforms are highly conserved [24,25,26,27]. Anyway, thanks to minor structural differences or discriminating inhibition mechanisms, selective inhibitors have been identified. For instance, Silverman’s team exploited one residue difference between nNOS and eNOS to discover highly selective nNOS inhibitors [28,29,30,31]. Several types of selective iNOS inhibitors have been identified, including (i) amidine-type substrate analogs, such as NIL or W1400, which irreversibly inactivate iNOS only [32,33,34]; (ii) dimerization iNOS inhibitors [35,36]; and (iii) competitive and reversible inhibitors exploiting a variable second shell residue in the substrate access channel [37]. Unfortunately, whereas several iNOS inhibitors have been the object of clinical studies, none were found to be useful because of toxicity, bioavailability, or inefficiency issues [38,39,40,41]. While adverse outcomes might originate from insufficient selectivity toward eNOS or, more generally, the difficulty to spare NO beneficial effects in chronic treatments [23], the usefulness of iNOS inhibition is questioned [42]. However, NOS inhibition could be useful in acute conditions, such as stroke [42] and/or in a synergistic association with another drug [43,44,45]. Finally, although potent, reversible and highly selective nNOS inhibitors with potential interests in stroke and other neuronal disorders have been identified, attaining sufficient permeability across the blood–brain barrier still requires some effort [46,47].

**Figure 1:**
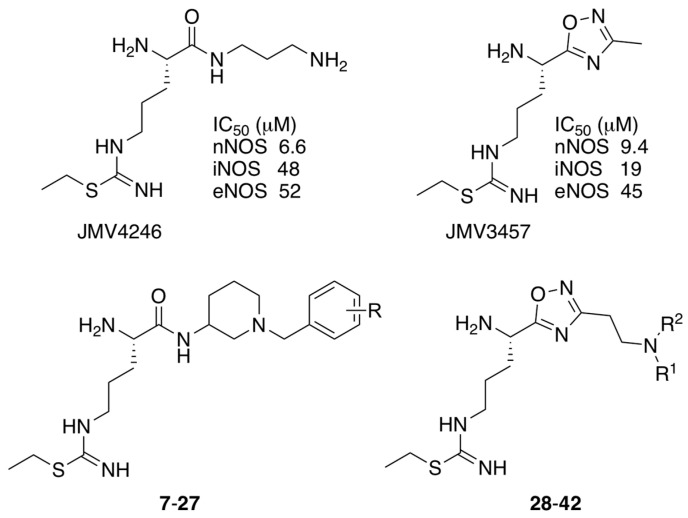
The structure of JMV4246 and 3457 and the general structure of present series of dipeptides **7**–**27** and 1,2,4-oxadiazoles **28**–**42** (R^1^ = H or CH_2_-Ar; R^2^ = H or R^1^).

In this context, we develop substrate-based dipeptidomimetic inhibitors following Silverman’s strategy [28]. We initially attached an extension onto the carboxyl side of a non-selective substrate-based inhibitor such as thiocitrulline (Tci), *S*-alkyl-isothiocitrullines (*S*-Me- and *S*-Et-Itc), and *N*-alkyl-arginines (*N*-Me- and *N*-Et-Arg) [48]. The extension is expected to interact in the less conserved substrate access channel of the protein, thus potentially affording affinity and selectivity. This approach is supported by an original solid-phase synthetic strategy [49], which allows the convenient synthesis of a large number of compounds from a single supported precursor. In this study, we prepared libraries of thiocitrullines, *S*-alkyl-isothiocitrullines, and *N*-alkyl-arginines extended on their carboxylate group via an amide bond (dipeptide-like) or by replacing the amide bond with isosteric heterocycles (i.e., 1,2,4- and 1,3,4-oxadiazoles, 1,2,4-triazoles) [48]. Although no highly potent NOS inhibitor has been identified in this first study, inhibition data generally showed significant selectivity toward eNOS, as most compounds did not inhibit this isoform, and most were in favor of nNOS. By comparing the inhibition efficiency for each of the five substrate-based inhibitors and each type of linker, we found that the best inhibitors generally contained an *S*-ethyl-isothiourea moiety and either an amide bond or a 1,2,4-oxadiazole heterocycle as a linker (see JMV4246 and JMV3457 as examples in Figure 1). Among favorable extensions, we identified aminoalkyl moieties as observed in previous reports [50,51]. Indeed, it was proposed that a basic amine group would establish an important ionic or hydrogen bond within the substrate access channel [28].

Based on these results, we kept these structural elements and further diversified the extension moiety. In particular, compared to our previously described compounds [48], we increased the length and/or volume of the extension to reach other regions of the substrate access channel where divergent interactions may take place, leading to higher potency and/or selectivity. We considered introducing variously substituted benzyl groups via reductive amination as a straightforward means to generate an extended series of compounds. The various substitutions on the benzene rings were chosen to obtain a diversity of chemical groups at different positions to try to establish beneficial interactions with the enzymes.

We presented a dipeptide-like series (compounds **7**–**27**) containing diverse benzyl groups attached to piperidine and several series of 1,2,4-oxadiazole compounds (**28**–**42**) containing mono- or dibenzylated aminoethyl substituents (Figure 1).

## 2. Results and Discussion

### 2.1. Chemistry

**Scheme 1:**
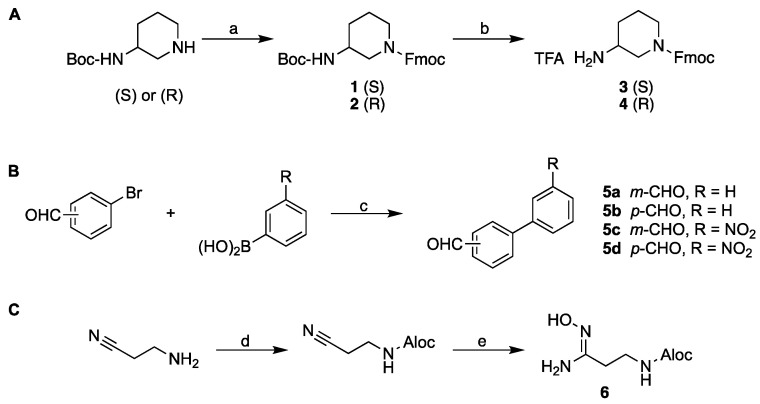
Synthesis of (**A**) (*S*) and (*R*) 9*H*-fluoren-9-ylmethyl 3-amino-1-piperidinecarboxylate **3** and **4**. Reagents and conditions: (a) 1 equiv. Fmoc-Cl, 2.5 equiv. Na_2_CO_3_, dioxane/water (1:1), rt, 4 h; (b) DCM/TFA (1:1), rt, 1 h; (**B**) biphenylaldehydes **5a**–**d**: (c) 0.03 equiv. Pd(OAc)_2_, 0.15 equiv. PPh_3_, EtOH/toluene (1:1), benzaldehyde, 2.5 equiv. Na_2_CO_3_, 1.3 equiv. boronic acid, 100 °C, 15 h; (**C**) 3-(*N*-allyloxycarbonyl-amino)-*N*-hydroxy-propanimidamide **6**: (d) 1.2 equiv. aloc-Cl, 2.0 equiv. Na_2_CO_3_, dioxane/water (1:1), rt, 15 h; (e) 5 equiv. NH_2_OH (50% in water), EtOH, 75 °C, 15 h.

Several intermediates were first synthesized as described in Scheme 1. For the dipeptide-like series, the two stereoisomers of 3-amino-1-Fmoc-piperidine **3** and **4** were prepared from the corresponding 3-(Boc-amino)-piperidines by Fmoc protection in the presence of Fmoc-Cl, followed by Boc removal from the resulting compounds **1** and **2** in acidic conditions (Scheme 1A). Biphenylaldehydes **5a**–**5d** were obtained in one step by a Suzuki cross-coupling reaction between *m*- and *p*-bromobenzaldehydes and the corresponding phenylboronic acids (Scheme 1B). For the 1,2,4-oxadiazole series, aloc-protected 2-aminoethyl-amidoxime **6** was prepared in two steps from 2-cyano-ethylamine by amine protection in the presence of aloc-Cl followed by treatment with aqueous hydroxylamine (Scheme 1C).

**Scheme 2:**
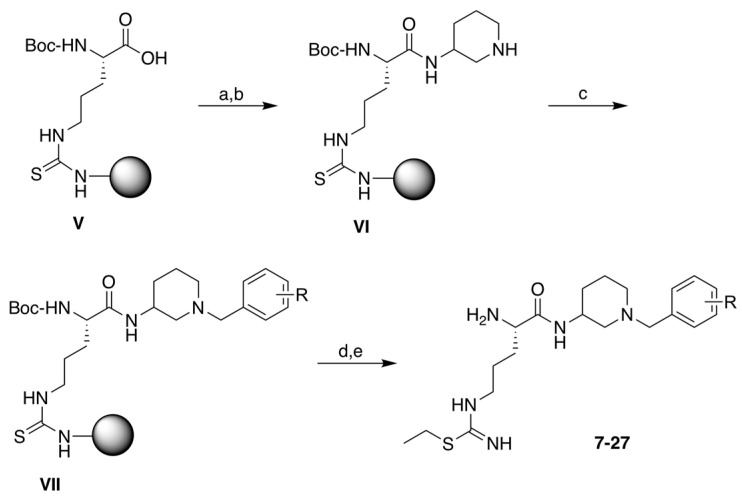
Synthesis of the dipeptidic compounds **7**–**27**. Reagents and conditions: (a) (S) (**3**) or (R) (**4**) *N*-Fmoc-(3-amino)-piperidine (1.2 equiv.), HBTU (1.5 equiv.), DIEA (3 equiv.), NMP, 4 h (twice); (b) 20% piperidine in DMF, 5 + 25 min; (c) benzaldehyde compound (4 equiv.) including **5a**–**5d**, NaBH_3_CN (2 equiv.), DMF/AcOH (98:2), rt, overnight; (d) 0.2 M EtI in DMF, 50 °C, 3 × 1 h; (e) TFA/TIS/H_2_0 (95:2.5:2.5), 40 °C, 2 × 2 h.

**Scheme 3:**
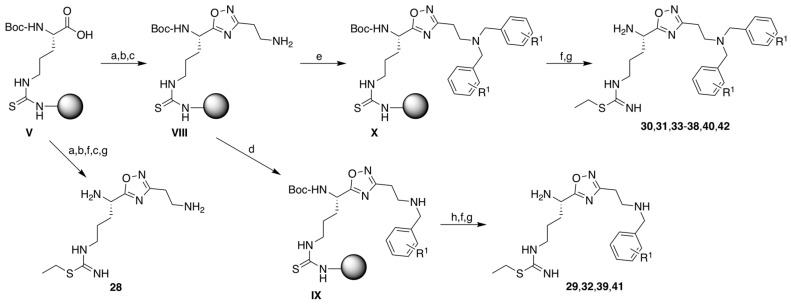
Synthesis of monosubstituted and homo-disubstituted 1,2,4-oxadiazole analogs **28**–**42**. Reagents and conditions: (a) amidoxime **6** (1.3 equiv.), HOBt, DIC, NMP, rt, 4 h; (b) AcONa (1.2 equiv.), THF/water (7/3), reflux, overnight (steps a and b were repeated one more time); (c) Pd(PPh_3_)_4_ (0.2 equiv.), PhSiH_3_ (24 equiv.), DCM, rt, 4 h (twice); (d) benzaldehyde compound (3 equiv.), NaBH_4_ (1.5 equiv.), DMF/MeOH (95:5), 0 °C, 15 min, + rt, 15 h; (e) benzaldehyde compound (3 equiv.), NaBH_3_CN (1 equiv.), DMF/AcOH (95:5), rt, overnight; (f) 0.2 M EtI in DMF, 50 °C, 3 × 1 h; (g) TFA/TIS/H_2_O (95:2.5:2.5), 40 °C, 2 × 2 h; (h) (Boc)_2_O (3 equiv.), TEA (3 equiv.), DMF, 2 × 2 h.

All final compounds were prepared on the solid phase from a single intermediate, a *N*α-Boc-protected thiocitrulline linked to the solid support via its thiourea group (compound **V**, Scheme 2 and Scheme 3). The solid-supported intermediate **V** was synthesized as previously reported [48,49] (Scheme S1).

#### 2.1.1. Synthesis of Dipeptide-like Compounds **7**–**27** (Scheme 2)

The solid-supported intermediate **V** was coupled to (S) (**3**) or (R) (**4**) 9*H*-fluoren-9-ylmethyl 3-amino-1-piperidinecarboxylate using HBTU and DIEA, followed by Fmoc removal in basic conditions. The free secondary amine was then submitted to reductive amination in the presence of various benzaldehydes, including biphenylaldehydes **5a**–**5d**, using NaBH_3_CN as the reducing agent. Finally, the thiourea was ethylated by treatment with ethyl iodide, as described [48], and compounds **7**–**27** were deprotected and released from the resin by cleavage with TFA (Scheme 2). The completion of several steps (i.e., coupling, reductive amination, and *S*-ethylation) was assessed by cleaving a small quantity of resin in acidic conditions and analyzing the obtained residue with LC-MS.

#### 2.1.2. Synthesis of 1,2,4-Oxadiazole Analogs **28**–**42** (Scheme 3)

Compared to dipeptide-like compounds, the replacement of the amide bond by the isosteric 1,2,4-oxadiazole ring might modulate affinity, selectivity, metabolic stability, and bioavailability [52].

The solid-supported intermediate **V** was coupled to amidoxime **6** (3-(*N*-allyloxycarbonyl-amino)-*N*-hydroxy-propanimidamide, Scheme 1C) using DIC/HOBt as coupling agents. The resulting compound was then cyclodehydrated in a mixture of THF/water at 80 °C and in the presence of sodium acetate, as previously described [48], followed by aloc removal with tetrakis (triphenylphosphine) palladium (0) in the presence of phenylsilane to yield the free supported primary amine **VIII**. Resin **VIII** was then treated following two sets of reductive amination conditions. Reaction with various benzaldehydes using NaBH_4_ led to supported monoalkylated derivatives **IX**. To obtain complete and only single substitution, aldehydes (3 equiv.) were first incubated with the resin for 3–4 h, followed by the addition of the reducing agent (1.5 equiv.) solubilized in methanol. The reaction time should not exceed 10 h as longer times led to lower yields, maybe because of damage caused to the resin. Using NaBH_3_CN led to the supported homo-dialkylated supported derivatives **X**. The best conditions included pre-incubation of benzaldehydes (3 equiv.) for 30 min in a mixture of 5% AcOH in DMF, followed by the addition of 1 equiv. reducing agent. *S*-Ethylation and TFA cleavage gave the final compounds **29**–**42**. In the case of monosubstituted compounds, the secondary amine of **IX** resins was first protected by treatment with Boc_2_O to prevent its ethylation. Compound **28** was similarly prepared from resin **V** but *S*-ethylation was performed before aloc deprotection.

In general, the completion of each reaction was checked by an LC-MS analysis of the residue obtained after the cleavage of a small portion of the resin. The linkage between the *S*-ethylisothiourea group and the Rink amide support is quite resistant, requiring a twice-repeated prolonged TFA treatment and light warming to recover the compounds from the solid support. All compounds were obtained in low-to-medium yields (5–50%) after reverse-phase HPLC purification.

### 2.2. Biological Evaluation

#### 2.2.1. Inhibition of NO Synthases

The inhibitory effects of all compounds against the three recombinant NOSs (rat nNOS, mouse iNOS, and bovine eNOS) were evaluated in 96-well plates using the oxyhemoglobin test [53]. A first screening at 100 and 10 μM was performed, and the IC_50_ values were measured for compounds showing more than 50% inhibition at 100 μM (Table 1 and Table 2).

In the dipeptide-like series (Table 1), eighteen different benzyl groups have been introduced on an (*S*)-amino-piperidine moiety, and three of them have also been combined with the (*R*) isomer to check the importance of piperidine relative orientation.

**Table 1:** Structures of dipeptide-like compounds **7**–**27** and in vitro NOS inhibition (IC_50_ in μM) ^1^.

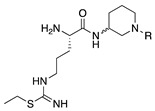					
Cpds	R	St ^2^	IC_50_ (μM)		
			nNOS (Rat)	iNOS (Mouse)	eNOS (Bovine)
**7**	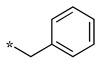	(S)	17.5	73.4	NI
**8**	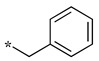	(R)	15.6	25.6	NI
**9**	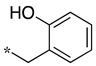	(S)	51.1	51.0	NI
**10**	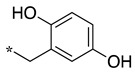	(S)	2.0	23.9	26.7
**11**	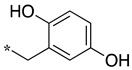	(R)	17.5	73.4	25.4
**12**	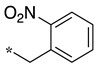	(S)	NI	46.0	NI
**13**	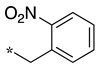	(R)	30.6	78.9	NI
**14**	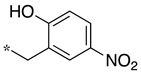	(S)	5.4	27.8	ND
**15**	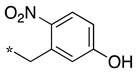	(S)	25.2	31.3	NI
**16**	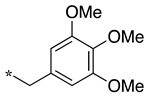	(S)	49.6	54.9	NI
**17**	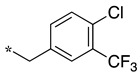	(S)	5.0	12.9	NI
**18**	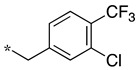	(S)	6.0	NI	NI
**19**	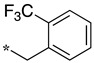	(S)	7.7	NI	NI
**20**	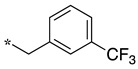	(S)	15.4	NI	NI
**21**	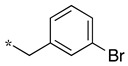	(S)	1.0	45.5	NI
**22**	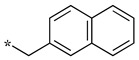	(S)	12.7	11.9	NI
**23**	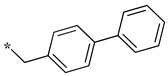	(S)	5.4	46.9	NI
**24**	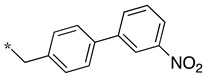	(S)	3.5	21.6	18.0
**25**	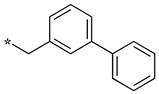	(S)	2.8	36.5	NI
**26**	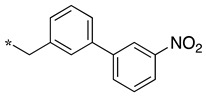	(S)	1.3	48.3	NI
**27**	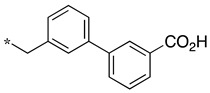	(S)	9.5	NI	NI

^1^ NI = no significant inhibition (<50% at 100 μM); ND = not determined. ^2^ Stereochemistry of piperidine C^3^. Assays were performed in triplicate.

As observed in the previous series [48], most compounds did not inhibit eNOS and were generally not or only modest inhibitors of iNOS. In the case of nNOS, the presence of hydroxy and/or nitro or methoxy groups on the benzyl moiety was generally not favorable (**9**–**13**, **15**, **16**). In addition, these compounds often showed no selectivity against iNOS, and sometimes, eNOS (i.e., the two isomers, **10** and **11**). One exception was compound **14**, showing an IC_50_ value in the micromolar range. In contrast, the substitution by halogens and halogenated groups (**17**–**21**) generally afforded more interesting compounds. Indeed, **17**–**19** and **21** were micromolar nNOS inhibitors, inactive against eNOS, and three of them (**18**, **19**, **21**) showed no or modest activity against iNOS. The introduction of a biaryl group (naphth-2-yl for compound **22**, *p*- or *m*-biphenyls for **23**–**27**) was somewhat less favorable, although most compounds displayed IC_50_ values in the low micromolar range. Indeed, lower selectivity against iNOS was observed, and the *m*-nitro-biphenyl **24** significantly inhibited eNOS. Finally, the stereochemistry of the amino-piperidine moiety did not show a significant effect on the inhibition profile (**8** vs. **7**, **11** vs. **10**, **13** vs. **12**). This is in contrast to the result reported with similar but unsubstituted amino-piperidine-containing compounds, for which a significant difference in nNOS inhibition was observed in favor of the R isomer [51].

In the 1,2,4-oxadiazole series (Table 2), four mono- and ten disubstituted compounds have been synthesized. For comparison, the non-substituted amino compound **28** was also prepared. The latter only poorly inhibited nNOS and iNOS and was inactive against eNOS. The mono- or disubstitution of the primary amine generally led to more potent compounds (up to 200 more for **37** in the case of nNOS inhibition). It is the case of the simplest analogs bearing one or two benzyl groups (**29** and **30**, respectively) that significantly inhibited both NOSs with the exception of eNOS for **30**. Concerning other mono/di pairs, disubstituted analogs were generally better inhibitors of nNOS and/or iNOS than their monosubstituted counterparts (i.e., **30** vs. **29** and **42** vs. **41** for both enzymes, **33** vs. **32** for nNOS only, and **40** vs. **39** for iNOS only). In the case of eNOS, the disubstitution was generally detrimental to its inhibition. One exception was compound **38**, which inhibited the enzyme with an IC_50_ value in the micromolar range.

**Table 2:** Structures of mono- and disubstituted 1,2,4-oxadiazoles analogs **28**–**42** and in vitro NOS inhibition (IC_50_ in μM) ^1^.

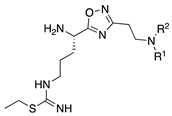					
Cpds	R^1^	R^2^	IC_50_ (μM)		
			nNOS (Rat)	iNOS (Mouse)	eNOS (Bovine)
**28**	-H	-H	58.9	83.7	NI
**29**	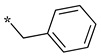	-H	13.4	11.1	17.9
**30**	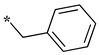	-R^1^	1.6	1.3	99.6
**31**	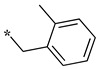	-R^1^	9.9	11.1	NI
**32**	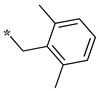	-H	60.1	51.6	ND
**33**	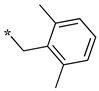	-R^1^	10.1	71.5	NI
**34**	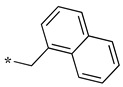	-R^1^	1.4	4.0	NI
**35**	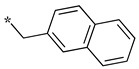	-R^1^	0.4	11.7	NI
**36**	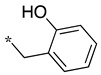	-R^1^	3.1	24.3	20.2
**37**	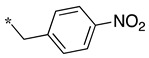	-R^1^	0.3	15.8	11.8
**38**	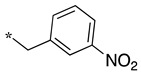	-R^1^	0.6	15.9	3.9
**39**	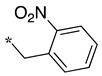	-H	27.0	8.0	48.2
**40**	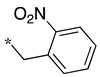	-R^1^	61.0	2.0	NI
**41**	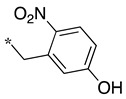	-H	3.0	36.3	ND
**42**	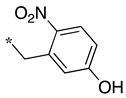	-R^1^	1.1	7.0	NI

^1^ NI = no significant inhibition (<50% at 100 μM); ND = not determined. Assays were performed in triplicate.

Overall, as observed in the dipeptide-like series, the highest inhibitory potencies were obtained against nNOS. Indeed, several compounds displayed IC_50_ values in the low micromolar (**30**, **34**, **36**, **41**, **42**) to submicromolar (**35**, **37**, **38**) range. These compounds were all disubstituted analogs (except **41**) and possessed either an unsubstituted phenyl (**30**) or naphthyl group (**34**, **35**) or a nitro group at any position on a phenyl ring (**37**, **38**, **41**, **42**), suggesting the importance of hydrophobic interactions and/or hydrogen bonding. Therefore, a few compounds were better nNOS inhibitors than any analog in the dipeptide-like series. Unfortunately, they generally showed lower selectivity toward iNOS and/or eNOS. Anyway, the best compounds, **35** and **37,** showed approximately 30- and 50-fold and >250- and 40-fold selectivity against iNOS and eNOS, respectively.

One exception was the mono/di pair **39** and **40** (*o*-nitrophenyl), which better inhibited iNOS. In particular, the disubstituted **40** showed a 30- and >50-fold selectivity toward nNOS and eNOS, respectively. This result suggested that introducing diverse bulky substituents susceptible to interaction with the substrate access channel might indeed modulate the inhibitory potency as well as the selectivity.

#### 2.2.2. Spectral Studies

We performed UV–visible difference spectroscopy to analyze the interaction mode of some inhibitors with the heme-active site of NOS [54]. We have studied the effects of dipeptide-like compounds **7**, **17**, **19**, **23,** and **27** and 1,2,4-oxadiazole **35** on the UV–visible spectra of the oxygenase domain of nNOS (nNOS_oxy_) and iNOS (iNOS_oxy_) [54]. As expected [48], when added stepwise to n- and iNOS_oxy_, all selected compounds elicited different spectra with a trough at ~425 nm and a peak at ~395 nm, similar to the substrate *L*-arginine. This profile is characteristic of type I interactions, meaning that they are bound in close proximity to the heme prosthetic group and shifted the spin state equilibrium to the pentacoordinated heme–Fe^III^ state. Double reverse plotting of the differences in absorbance between the peak at ~395 nm and valley at ~425 nm (ΔA_395–425 nm_) as a function of the concentration of the added compounds allowed calculating apparent binding constants (*K*_s_) (Table 3). The reference inhibitor SEITU [55] tightly bound both NOS isoforms with high affinity but no selectivity. With the exception of the dipeptide-like **27** and the heterocyclic analog **35**, which similarly interacted with both proteins, other compounds preferentially bound nNOS_oxy_ over iNOS_oxy_ with apparent affinities close to SEITU for this isoform. Interestingly, in this series of compounds, similar selectivity of nNOS over iNOS was observed when comparing inhibitory potencies, except compounds **27** and **35**, which showed significant selectivity toward nNOS (Table 1 and Table 2).

#### 2.2.3. Cell Toxicity

Cell viability was evaluated in the murine macrophage RAW 264.7 cells using an MTT assay for compounds **18**, **21**, **26,** and **30**. This experiment was performed without or with LPS (1 µg/mL), which induces iNOS expression (Figure S1). In the absence of LPS, compounds **21** and **30** did not modify cell viability up to 100 μM, but compounds **18** and **26** were fully toxic at this concentration (Figure S1). Compound **30** only killed 20% of the cells at 500 μM (Figure S1A). In the presence of LPS (1 µg/mL), cell viability was reduced by 30% due to the inhibition by NO of key enzymes involved in cell metabolism [56] (Figure S1B). Compounds **18**, **21,** and **26** showed similar behavior as observed without LPS, but none partially reversed the LPS effect in contrast to L-NIL, SEITU, and a few of our previously reported compounds [48].

#### 2.2.4. Inhibition of NOS in Cells

To study the potential usefulness of these compounds to inhibit NOS expressed in cells, we performed several series of experiments on selected compounds.

**Figure 2:**
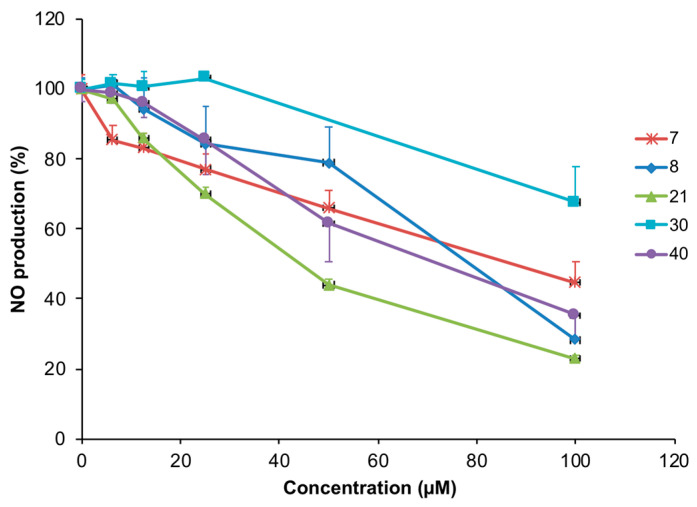
Inhibitory effects of dipeptides **7**, **8**, and **21** and 1,2,4 oxadiazoles **30** and **40** on NO production by RAW 264.7 cells stimulated by LPS (1 μg/mL) (n = 3).

We evaluated the ability of selected compounds (dipeptides **7**, **8**, and **21** and 1,2,4-oxadiazoles **30** and **40**) to inhibit iNOS in RAW 264.7 cells (Figure 2), where iNOS was induced by LPS (1 µg/mL). iNOS activity was measured following LPS induction by quantifying nitrite accumulation in the supernatant. Overall, dipeptide-like compounds showed better intracellular inhibition of NO production (up to 80% inhibition at 100 μM for **21**) than oxadiazoles, despite the latter being about 20- to 30-fold more potent iNOS inhibitors. This result suggests that the 1,2,4-oxadiazole compounds were less prone to enter cells than the dipeptide-like analogs. A similar result was previously reported [48].

**Figure 3:**
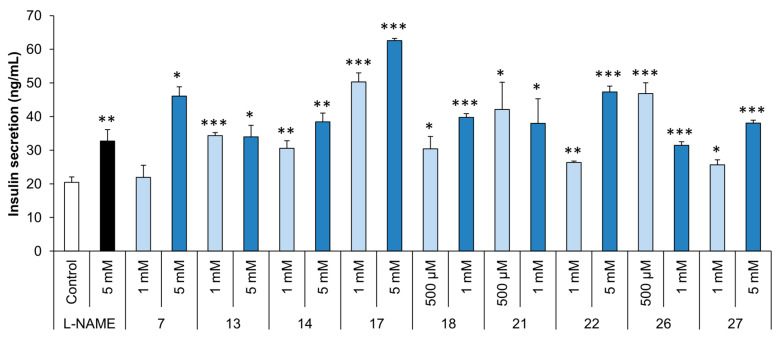
The effect of *L*-NAME and compounds **7**, **13**, **14**, **17**, **18**, **21**, **22**, **26,** and **27** on insulin secretion by INS-1 cells in the presence of 5.6 mM glucose and without iNOS induction with IL-1β. Compounds were incubated simultaneously with stimulating glucose for one hour on INS-1 cells and then supernatants were collected for the measurement of insulin release. *, *p* < 0.05; **, *p* < 0.01; ***, *p* < 0.001; (n = 3–5).

Finally, as some compounds are found to be more selective for nNOS rather than iNOS, we assessed the ability of dipeptide-like compounds to modulate insulin secretion in INS-1 cells in the presence of 5.6 mM glucose and in the absence of IL-1β and, therefore, iNOS induction. Indeed, pancreatic β-cells are known to constitutively express the nNOS isoform, which negatively modulates glucose-induced insulin secretion [5]. As previously described [57], the NOS inhibitor *L*-NAME (5 mM) potentiated insulin release induced by 5.6 mM glucose (Figure 3). Dipeptide compounds, such as **7**, **14**, **17**, **18**, **22,** and **27** (from 500 µM to 5 mM), were also incubated for one hour to avoid potential toxic effects and were able to dose-dependently stimulate glucose insulin secretion with better efficiency than *L*-NAME. Three compounds, **13**, **21,** and **26,** displayed a maximum effect at 1 mM or 500 µM, respectively.

Overall, these results support these compounds being able to inhibit intracellular nNOS and affect cellular functions.

### 2.3. Modeling Study

We investigated the putative binding mode of dipeptide-like **21** and **26** and 1,2,4-oxadiazole **30** and **35** compounds within the active site of nNOS and eNOS via docking experiments. In these experiments, we assumed that the isothiourea moiety of the inhibitors will interact with the heme propionate and Glu592 and Glu363 residues in nNOS and eNOS, respectively, like the arginine guanidinium [27]. The objective was to identify potential interactions within the substrate access channel and explain the selectivity of these compounds for nNOS against eNOS

The docking experiments were performed with AutoDock 4.2 software using the Local Search algorithm on nNOS and eNOS models generated from the crystallographic structures 1P6J and 1P6N [29], respectively, available in the Protein Data Bank (PDB). 1P6J and 1P6N are the three-dimensional structures of the complex formed between the highly selective nNOS inhibitor *L*-*Nω*-nitroarginine-(4*R*)-amino-*L*-prolinamide (called DP9, https://www.rcsb.org/ligand/DP9, accessed on 16 March 2023) and nNOS and eNOS, respectively. DP9 possesses an *Nω*-nitroarginine as a substrate analog and a 4-aminoprolinamide moiety as an extension. Our compounds were expected to similarly bind in both nNOS and eNOS. The high selectivity (i.e., 1000-fold [29]) of DP9 in favor of nNOS is mainly due to one residue variation, Asp597 in nNOS replaced by Asn368 in eNOS. In the complexes of the two isoforms with their substrate arginine (pdb codes 1OM4 and 2G6O for nNOS and eNOS, respectively), the side chain groups (protonated carboxylic for Asp597, primary amide for Asn368) make the H-bond with the α-carboxylate group of the substrate, and the H-bond network is fully identical to the two complexes. However, when introducing an extension on the α-carboxylate group of a substrate analog (as in DP9), this difference very significantly impacts the binding with eNOS [29].

We first docked the original ligand DP9 in both isoforms to check that our protocol allowed crystallographic positioning. This study was performed in the presence and absence of water molecules involved in the H-bond network formed between the enzymes and inhibitor. For each docking, only 1 cluster of 1000 solutions was obtained. As expected, while the guanidine moiety of DP9 makes a bifurcated hydrogen bonding interaction to the conserved active site glutamate in both nNOS (Glu592) and eNOS (Glu363), its α-amine establishes a strong electrostatic interaction with Glu592 in nNOS, but not Glu363 in eNOS. Compared to the crystallographic structure, an extra interaction was apparent in the docking structure between the primary amide of DP9 with one propionic acid of the heme in both isoforms. However, overall, the superimposition of the best docking pose with the crystallographic one showed very similar positioning of DP9 in both enzyme binding sites. In addition, the difference in mean docking scores was about 100-fold in favor of nNOS, in satisfactory agreement with the difference in experimental *K*_i_ values (0.1 vs. 110 μM for nNOS and eNOS, respectively [29]).

The docking of DP9 in nNOS and eNOS was, therefore, satisfactory, and its conditions were applied to the docking of our four inhibitors. Several residues expected to be involved in binding were let flexible, including Glu592/363, Gln478/249, Arg481/252, Asn569/340, Ser477/248, and Tyr706,477 (numbering in nNOS and eNOS, respectively).

#### 2.3.1. Compound **21**

**Figure 4:**
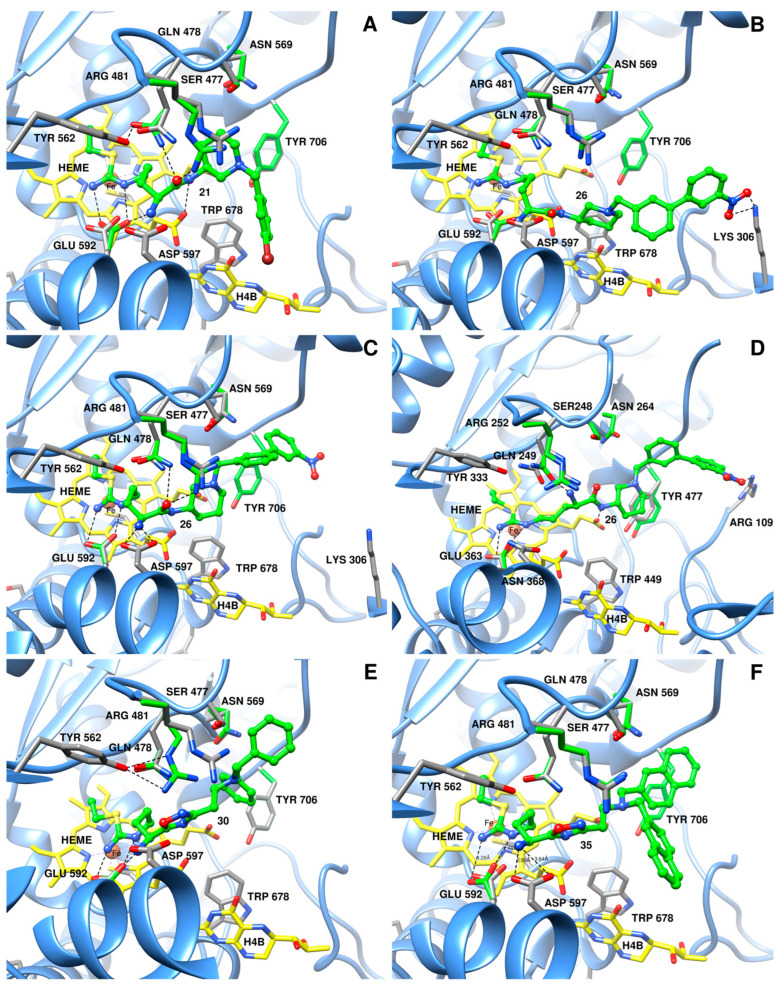
The best docking pose of (**A**) compound **21** in nNOS; (**B**) compound **26** in nNOS (pose 1); (**C**) compound **26** in nNOS (pose 2); (**D**) compound **26** in eNOS; (**E**) compound **30** in nNOS; (**F**) compound **35** in nNOS. Blue: protein ribbon; green stick: protein side chain adaptation in docking; green ball and stick: molecules of interest; yellow: cofactors of NOS (heme and tetrahydrobiopterin (H4B)).

Dipeptide-like **21** is characterized by a piperidine moiety *N*-substituted by a 3-bromobenzyl group. Its docking in nNOS delivered only one pose, which corresponds to DP9 with Glu592 binding to both the isothiourea nitrogens and the α-amine (Figure 4A). However, this solution positions the bromine in a hydrophilic environment involving Arg596, Asp600, and Ser602, which is expected to be poorly favorable. Docking in eNOS yielded heterogeneous results with unfavorable mean binding energies (>−6.5 kcal/mol). The most probable pose corresponds to that observed in nNOS, and no significant difference is observed in the substrate access channel. In this case, the difference in mean binding energies (−8.3 vs. −6.5 kcal/mol for nNOS and eNOS, respectively) is probably mainly due to the difference in electrostatic energy related to the Asp597/Asn368 variation.

#### 2.3.2. Compound **26**

The second dipeptide-like compound possesses an *m*-nitro-*m*-biphenyl substituent. In contrast to **21**, several poses were obtained when docked into nNOS. The major one shows the interaction of the α-amine with Asp597 and the nitro group with Lys304 (Figure 4B). Despite the latter interaction, the docking score is modest (a mean binding energy of −8.0 kcal/mol), possibly because it destabilizes the conserved binding between the isothiourea and Glu592. The latter is present in the second most probable pose, as well as the interaction between the α-amine and Asp597, but the nitro group is solvent-exposed and does not establish any interaction (Figure 4C).

A different pose was preferentially observed in eNOS with possible stabilizing interactions between the extending moiety of the molecule and the residues Tyr477 and Arg109 (nitro group) (Figure 4D). In this case, the positioning of the thiourea over Glu363 is not optimal, and the α-amine interacts with Gln249, which might explain why the mean binding energies are not favorable (>−8 kcal/mol) and why the compound was found to not inhibit eNOS.

#### 2.3.3. Compound **30**

The ethylamine moiety of oxadiazole analog **30** is substituted by two benzyl groups. When docked in nNOS, the pose with the lowest binding energy corresponds to the expected one (Figure 4E). The isothiourea nitrogens and the α-amine interact with Glu592. The two benzyl groups are oriented toward Ser477 and Asn569 without performing specific interactions, and the tertiary amine is exposed to the solvent. Finally, the oxadiazole moiety does not establish any interaction. The heterocycle can only accept H-bonds, and there is no H-bond donor in its immediate environment. However, it cannot be excluded to be involved in solvent-mediated interactions.

The docking result for eNOS is identical to the result obtained with nNOS. However, a significant difference in docking score was observed in favor of nNOS (mean binding energies of −9.1 and −6.9 kcal/mol for nNOS and eNOS, respectively). It is again explained by the higher electrostatic weight for nNOS due to the Asp597/Asn368 difference between nNOS and eNOS.

#### 2.3.4. Compound **35**

Compared to **30**, the ethylamine moiety of **35** is substituted by two naphth-2-yl groups. In nNOS, the isothiourea nitrogens interact with Glu592 and the α-amine with Asp597 in most solutions. Because of their size, the naphthyls cannot stand at the same position as the benzyls of **30**. One is oriented toward Tyr706, while the second is close to Ala497. This forces the other part of the molecule to position lower in the binding site, possibly allowing the tertiary amine to interact with the propionic acid moieties of the heme group (Figure 4F). Compound **35** obtained a very good docking score (a mean binding energy of −10.2 kcal/mol) in accordance with its experimentally determined inhibitory potency (an IC_50_ value of 0.4 μM, Table 2).

When docked in the eNOS active site, two main poses were defined. In the most populated one (a mean binding energy of −8.8 kcal/mol), the molecule shifted from the DP9-like pose where the conserved interaction with Glu363 is lost, and the α-amine makes an H-bond with Gln249. The second solution is the same for nNOS. In particular, the two naphthyls are similarly positioned. In this case, the difference in docking scores (mean binding energies of −10.2 vs. −8.4 kcal/mol for nNOS and eNOS, respectively) results again from the difference in electrostatic energies related to Asp597/Asn368 variation.

Overall, each docking showed the possibility for each molecule to interact in the substrate binding site, like DP9. However, the docking study did not show any contribution of the molecule extensions to the selectivity between nNOS and eNOS. It seems that this selectivity is only due to Asp597/Asn368 variability between the two isoforms, as shown for DP9 [29], resulting in a higher contribution of electrostatic energy to nNOS binding. The calculated mean binding energies are consistent with the experimental results, which showed that the molecules more efficiently inhibit nNOS. Whereas the extensions seemed to only marginally contribute to the binding energy, the docking experiment suggests that their size could have an impact. Compared to **21**, the docking of the more extended **26** in nNOS opens up the possibility of a favorable interaction between the nitro group and a residue in the substrate access channel, which could be achieved by the design of new molecules. In addition, the binding of oxadiazole analog **35** was found to be favorably influenced by the bulkiness of its two naphthyl groups compared to dibenzyl analog **30**. This result is consistent with the higher inhibitory potency of **35** compared to **30** (IC_50_ values of 0.4 and 1.6 μM, respectively).

## 3. Conclusions

In this study, we have synthesized 36 new compounds (21 dipeptide-like compounds, 15 1,2,4-oxadiazoles) using our original solid-phase synthetic strategy.

All compounds were tested against three recombinant NOS isoforms. Overall, several new compounds showed significant inhibitory activity (an IC_50_ of about 0.3 to 15 μM) toward at least one NOS, with a global preference for nNOS that was followed by iNOS, whereas eNOS was scarcely ever inhibited. These results are in accordance with those obtained by Silverman’s team on nitro-arginine-containing dipeptides and our previous studies [28]. While some dipeptide-like compounds inhibited nNOS with IC_50_ values in the micromolar range, several 1,2,4-oxadiazole analogs were submicromolar inhibitors. However, the latters were less efficient than the formers in cell tests, suggesting that the 1,2,4-oxadiazole did not favor cell penetration. Finally, docking studies gave insight into the binding mode of our compounds in the substrate access channel, which will help the design of further analogs with improved activity.

## 4. Materials and Methods

### 4.1. General

#### 4.1.1. Materials

Protected ornithine, DIC, HOBt, HBTU, DIEA, TFA, piperidine, solvents, and other reagents were purchased from Iris-Biotech, Novabiochem, Riedel-de Haën, Carlo Erba, or Acros organics and used without further purification. Fmoc Rink amide polystyrene resin (100–200 mesh, 0.94 mmol/g) was purchased from Iris-Biotech. Solvents used for RP-HPLC and LC-MS were of HPLC grade.

#### 4.1.2. NMR Spectroscopy

^1^H NMR spectra were recorded at 300, 400, or 500 MHz using DMSO-*d*_6_. Splitting patterns were designated as follows: s, singlet; d, doublet; t, triplet; q, quartet; m, multiplet; and br, broad. ^13^C NMR spectra were recorded at 75, 101, or 125 MHz using DMSO-*d*_6_.

#### 4.1.3. Analysis and Purification

RP-HPLC analysis was performed on a Chromolith SpeedRod C18 column (0.46 cm × 5 cm) using a linear gradient (0–100%) of eluent B in A for 5 min at a flow rate of 3 mL/min.

Compounds were purified by preparative RP-HPLC on a Waters Delta Pak C18 column (40 mm × 100 mm, 15 µm, 100 Å) by using a linear gradient of eluent B in A at a 1%/min rate and a flow rate of 28 mL/min. Some compounds were purified on a preparative RP-HPLC coupled to a mass spectrometer (Autopurif system from Waters driven by the software MassLynx 4.0 Fractonlynx) using a Waters X Bridge Prep C18 column (19 mm × 100 mm, 5 μm) and a linear gradient of eluent B in A (flow rate: 20 mL/min).

Eluent A: water/0.1%TFA and eluent B: acetonitrile/0.1% TFA. Detection was made at 214 nm.

Purification of precursors was performed on a column loaded with Merck silica gel 60 with a particle size of 40–63 μm.

Mass spectrometry: samples were prepared in an acetonitrile/water (50/50 *v*/*v*) mixture. The LC-MS system consisted of a Waters Alliance 2690 HPLC coupled to a Waters-Micromass ZQ spectrometer (electrospray ionization mode, ESI+). All separations were carried out using an RP C18 monolithic Onyx Phenomenex column (25 mm × 4.6 mm) by means of a linear gradient (0–100%) of eluent B in A for 3 min at a flow rate of 3 mL/min. Eluent A: water/0.1% formic acid and eluent B: acetonitrile/0.1% formic acid. Positive ion electrospray mass spectra were acquired at a solvent flow rate of 100–500 µL/min. Nitrogen was used as both the nebulizing and drying gas. These data were obtained in a scan mode in 0.1 s intervals; 10 scans were summed up to obtain the final spectrum.

All final compounds were obtained as TFA salts. The HPLC retention times, calculated monoisotopic mass, measured high-resolution mass of the compounds, NMR data, and yields of purified products are reported in the Supplementary Materials.

### 4.2. Chemical Synthesis of the Supported Intermediates

The supported thiocitrulline intermediate **V** was prepared as reported [48] and briefly described in the Supplementary Materials (Scheme S1).

#### 4.2.1. Synthesis of the Supported Dipeptide-like Intermediates **VI** (S) and (R)

The supported thiocitrulline intermediate **V** (1.40 mmol) was swelled in NMP for 15 min and filtered. We then successively added NMP solutions of (S) 1-*N*-Fmoc-(3-amino)-piperidine **3** (Scheme S2) (733 mg, 1.68 mmol, 1.2 equiv.), DIEA (734 μL, 4.20 mmol, 3 equiv.), and HBTU (910 mg, 2.40 mmol, 1.5 equiv.). After 4 h of stirring, the resin was filtered and washed with DMF, methanol, and DCM. The residue obtained after a cleavage test was analyzed by LC-MS (*t*_R_: 1.29 min; *m*/*z* (ES+) 496.2 (M + H^+^)).

The (R) isomer was similarly obtained from the corresponding protected 3-amino-piperidine **4**.

Fmoc removal was performed by two treatments with a 20% piperidine solution in DMF for 10 and 30 min. The resin was then washed as described above.

#### 4.2.2. Synthesis of the Supported Dipeptide-like Intermediates **VII**: Reductive Amination

Each resin **VI** was swelled in DMF/AcOH (98/2) and a benzaldehyde (4 equiv.), including biphenyl aldehydes **5a**–**5d** (Scheme S3), was added. After 30 min of stirring at rt, NaBH_3_CN (2 equiv.) was added, and stirring continued overnight. The resin was finally filtered and washed as described above.

#### 4.2.3. Synthesis of the Supported 1,2,4-Oxadiazole Intermediate **VIII**

The supported thiocitrulline **V** (2.26 mmol) was placed in a balloon, swelled in NMP for 15 min, and cooled to –10 °C. We then added NMP solutions of amidoxime **6** (Scheme S4) (550 mg, 2.94 mmol, 1.3 equiv.), HOBt, H_2_O (397 mg, 2.94 mmol, 1.3 equiv.), and DIC (458 μL, 2.94 mmol, 1.3 equiv.). After 20 min at –10 °C and 4 h at room temperature, the resin was filtered and washed with DMF, methanol, and THF. For the cyclodehydration step, the resin was conditioned in THF/H_2_O (7:3), an aqueous solution of sodium acetate (222 mg, 2.71 mmol, 1.2 equiv.) was added, and the mixture was refluxed for 5 h under mild stirring. After washing with DMF, methanol, and NMP, coupling and cyclodehydration were repeated.

To remove the aloc group, the resin was swelled in dry DCM, and tetrakis Pd(PPh_3_)_4_ (0.2 equiv.) and phenylsilane (24 equiv.) were added. The resin was stirred for 4 h and washed with DCM, methanol, and DCM. This treatment was repeated.

#### 4.2.4. General Synthesis of the Supported 1,2,4-Oxadiazole Intermediates **IX**: Mono-Alkylation

Resin **VIII** was swelled in dry DMF and cooled to 0 °C, and a benzaldehyde compound (3 equiv.) was added. After 15 min of stirring at 0 °C and 3 h at rt, the mixture was cooled again to 0 °C, and NaBH_4_ (1.5 equiv.) solubilized in a minimal volume of dry MeOH was added. After 15 min of stirring at 0 °C and 10 h at rt, the resin was filtered and washed with DMF, MeOH, and DCM.

#### 4.2.5. General Synthesis of the Supported 1,2,4-Oxadiazole Intermediates **X**: Homo-Dialkylation

Resin **VIII** was swelled in a mixture of dry DMF/AcOH (95:5), and a benzaldehyde (3 equiv.) was added. After 30 min of stirring at rt, NaBH_3_CN (1.5 equiv.) was added. The mixture was stirred overnight at rt, filtered, and washed with DMF, MeOH, and DCM.

#### 4.2.6. Synthesis of Supported S-Ethyl-Isothiourea Derivatives

A supported thiourea derivative was swelled in DMF for 15 min and filtered and a 0.2 M solution of EtI (15 equiv.), and DMF was added. The reaction was stirred at 50 °C for 1 h and was repeated twice. The resin was then washed with DMF and DCM and dried.

#### 4.2.7. Cleavage from the Solid Support

Deprotection and final cleavage of the compounds from the solid support were performed with TFA/TIS/H_2_O (95:2.5:2.5, 10 mL/g resin) at 40 °C for 2 h. This treatment was repeated twice. After resin filtration, the filtrate was concentrated under a vacuum, and the compounds were precipitated by diethyl ether addition and recovered after centrifugation. The pellet was washed twice with a diethyl ether. When no precipitation occurred, the residues were solubilized in water/MeCN (50:50) and freeze-dried. All compounds were obtained with an average yield of 5–50% after reverse-phase HPLC purification. All compounds were above 90–95% purity.

For the cleavage test, some reaction steps (coupling, 1,2,4-oxadiazole formation, reductive amination, *S*-ethylation for selected compounds) were checked by cleaving 2–3 mg of resin with a solution of TFA/TIS/H_2_O (95:2.5:2.5, *v*/*v*/*v*, 0.5 mL) for 1 h at room temperature. After filtration, the mixture evaporated under a nitrogen stream. The residue was solubilized in 50% MeCN/H_2_O and submitted to LCMS analysis.

### 4.3. Biological Evaluation

L-Arginine, L-citrulline, *Nω*-nitro-L-arginine, dithiothreitol (DTT), hemoglobin, superoxide dismutase, catalase, bovine serum albumin, L-NAME, SEITU, (*6R*)-5,6,7,8-tetrahydrobiopterin, NADPH, porcine brain calmodulin, and all common salts and buffers were purchased from Sigma-Aldrich (St. Louis, MO, USA).

#### 4.3.1. Production of Recombinant NOSs

Full-length recombinant rat nNOS, mouse iNOS, bovine eNOS, and the heme domains of rat brain nNOS and mouse iNOS were expressed in *Escherichia coli* and purified as described previously [58,59,60,61,62].

Protein concentrations were determined by Bradford’s method using bovine serum albumin as a standard and the Bradford reagent from Biorad [63]. The heme concentrations of the purified NOS were determined optically from the [Fe^II^-CO]–[Fe^II^] difference spectrum using an ΔA_445–480 nm_ of 74 mM^−1^·cm^−1^ [64]. They were estimated to be more than 95% pure by SDS-PAGE electrophoresis.

#### 4.3.2. Measurement of NO Production by Recombinant NOSs

NOS catalytic activity was measured using the hemoglobin capture assay [53]. The test was performed at 30 °C in 96-well microplates using a final volume of 200 µL. The assay mixture contained 100 µM NADPH, 10 µM BH_4_, 6 µM HbO_2_, 100 µM DTT, 5 µM FAD, 5 µM FMN, 10 µM (nNOS and eNOS), or 20 µM (iNOS) arginine, all in a 100 mM Hepes buffer, pH 7.5, and, for constitutive n- and e-NOS, 10 µg/mL calmodulin, and 1 mM CaCl_2_. The studied compounds were introduced as 2 μL of ×100 concentrated solutions in DMSO, and the control experiments were performed with DMSO or the buffer only. The kinetics of NO production were measured at 401 nm using an Infinite F500 microplate reader (Tecan, Singapore).

#### 4.3.3. Effects of Selected Compounds on UV-Visible Spectra of n- and iNOSoxy

This study was performed as previously described [48].

#### 4.3.4. Evaluation of Cellular Models

The macrophage cell line RAW 264.7 (a gift from Dr. A. Blangy, Montpellier, France) was cultured in Dulbecco’s Modified Eagle’s Medium (DMEM) supplemented with 10% FCS, 100 units/mL penicillin, 100 µg/mL streptomycin, and 2 mM L-glutamine.

The insulin-secreting cell line INS-1 (a gift from Prof. C. Wolheim, Geneva, Switzerland) was cultured in RPMI-1640 supplemented with 10% FCS, 100 units/mL penicillin, 100 µg/mL streptomycin, 2 mM L-glutamine, 10 mM HEPES, 1 mM sodium pyruvate, and 50 mM 2-mercaptoethanol, according to M. Asfari et al.’s method [65].

In RAW 264.7 cells, iNOS was induced by LPS (1 µg/mL, Sigma-Aldrich) for 24 h. All studied compounds were incubated with cells during the last 24 h.

Cellular NO production was measured using the Griess reagent (Sigma-Aldrich), which evaluates the nitrite content in culture media [66]. Cellular toxicity was estimated by the MTT assay (Sigma-Aldrich) in RAW 264.7 cells after the induction (or not) of iNOS by LPS, according to the manufacturer’s recommendations.

The functional effects of compounds were evaluated on INS-1 cells. Cells were preincubated for 1 h at 37 °C in a Krebs–Ringer bicarbonate buffer (108 mM NaCl, 1.19 mM KH_2_PO_4_, 4.74 mM KCl, 2.54 mM CaCl_2_, 1.19 mM MgSO_4_·7H_2_O, and 18 mM NaHCO_3_) containing 2 g/L BSA in the absence of glucose. After removal of the medium, the cells were stimulated for another hour at 37°C in the same buffer in the presence of 5.6 mM glucose and the presence of increasing concentrations of *L*-NAME or compounds (from 500 µM to 5 mM). At the end of the incubation period, the medium was collected, and the insulin was measured using an insulin high-range assay kit (CisBio, Codolet, France). Data are expressed as means ± SEM of the experiments (indicated in the result part).

### 4.4. Modeling Study

The geometric optimization of the compound structures was performed using Gaussian 16 at the DFT level of theory with B3LYP hybrid functional and 6–31 g(d,p) basis set. The resulting structures were registered as pdb files. The molecules were then the object of charge calculation using the AM1-BCC model, and the files were registered in mol2 format using the Dockprep module found in Chimera software. Then, the docking studies were performed with AutoDock 4.2.6 [67], with Local Search as the docking algorithm and 1000 iterations. All other parameters were default values. The docking search was performed in a grid box centered on a ligand of 80 × 80 × 80 (Å^3^) with the standard 0.375 Å resolution to encompass all flexible side chains chosen. Files for the docking were prepared from (i) the structure of the complexes formed by *L*-*Nω*-nitroarginine-(4*R*)-amino-*L*-prolinamide (called DP9, https://www.rcsb.org/ligand/DP9, accessed on 16 March 2023) with nNOS (1P6J.pdb) and eNOS (1P6N.pdb) [29], which were treated as follows. Water molecules and small molecules were removed, the protein was protonated with the Dockprep module found in Chimera, charges were calculated using the AM1-BCC model, and the file was registered as the mol2 format. (ii) Compounds and protein pdbqt files were prepared with AutoDockTools (ADT) [68]. For each protein, side chains of six conserved residues were let to be flexible: Glu592/363, Gln478/249, Arg481/252, Asn569/340, Ser477/248, and Tyr706/477 (numbering for nNOS and eNOS, respectively).

Molecular graphics and analyses were performed with UCSF Chimera, developed by the Resource of Biocomputing, Visualization, and Informatics at the University of California, San Francisco, with support from NIH P41-GM103311.

## Figures and Tables

**Table 3:** Apparent equilibrium constants (*K*_s_, μM) for the binding of some dipeptide-like analogs **7**, **17**, **19**, **23**, and **27**, and 1,2,4-oxadiazole **35** to nNOS_oxy_ and iNOS_oxy_ ^1^.

Cpds	*K*_s_ (μM)	
	nNOS_oxy_	iNOS_oxy_
**SEITU**	6.4 ± 1.5	1.7 ± 0.1
**7**	7.1 ± 2.6	406 ± 97
**17**	3.6 ± 1.5	36.4 ± 4.6
**19**	5.2 ± 1.1	96 ± 36
**23**	11.4 ± 3.8	92.5 ± 8.8
**27**	70.6 ± 22.8	57.9 ± 8.0
**35**	47.1 ± 13.0	42.4 ± 8.7

^1^ Titrations were performed by UV/Vis difference spectroscopy, as described in Experimental Section. Values ± SD from three different experiments. SEITU was added for comparison.

## Data Availability

Data are contained within the article or Supplementary Materials.

## Supplementary Materials

The following supporting information can be downloaded at https://www.mdpi.com/article/10.3390/molecules28135085/s1. Synthesis and characterization of precursors; Characterization data for dipeptides **7**–**27**; Characterization data for 1,2,4-oxadiazole compounds **28**–**42**; Figure S1: cell toxicity on RAW264.7 cells; Figure S2: molecular modelling (DP9); Selected ^1^H and ^13^C NMR spectra; Scheme S1 (Synthesis of the supported thiocitrulline intermediate **V**); Scheme S2 (Synthesis of (*S*) and (*R*) 9*H*-fluoren-9-ylmethyl 3-amino-1-piperidinecarboxylate **3** and **4**); Scheme S3 (Synthesis of biphenylaldehydes **5a**–**d**); Scheme S4 (Synthesis of 3-(*N*-allyloxycarbonyl-amino)-*N*-hydroxy-propanimidamide **6**).
