# Supplementary material for: S-Ethyl-Isothiocitrullin-Based Dipeptides and 1,2,4-Oxadiazole Pseudo-Dipeptides: Solid Phase Synthesis and Evaluation as NO Synthase Inhibitors
Source: Molecules. 2023 Jun 29;28(13):5085. doi: 10.3390/molecules28135085 (PMC10343299; doi:10.3390/molecules28135085)
Supplement: Supplementary file 1 [file molecules-28-05085-s001.zip › molecules-2458990-supplementary.pdf]

Supplementary Materials

S-Ethyl-Isothiocitrullin-Based Dipeptides and 1,2,4-Oxadiazole Pseudo-Dipeptides: Solid Phase Synthesis and Evaluation as NO Synthase Inhibitors

Elodie Mauchauffée[1], Jérémy Leroy[2], Jihanne Chamcham[1], Abdelaziz Ejjoummany[1], Manon Maurel[1], Lionel Nauton[3], Booma Ramassamy[4], Karima Mezghenna[2], Jean-Luc Boucher[4], Anne-Dominique Lajoix[2,*], and Jean-François Hernandez[1,*]

Table of contents.

- Synthesis and characterization of precursors S2

- Characterization data for dipeptides **7-27** S6

- Characterization data for 1,2,4-oxadiazole compounds **28-42** S13

- Figure S1: cell toxicity on RAW264.7 cells S19

- Figure S2: molecular modelling (DP9) S20

- Selected ^{1}H and ^{13}C NMR spectra S22

Synthesis

1- Synthesis of the supported thiocitrullin intermediate V:

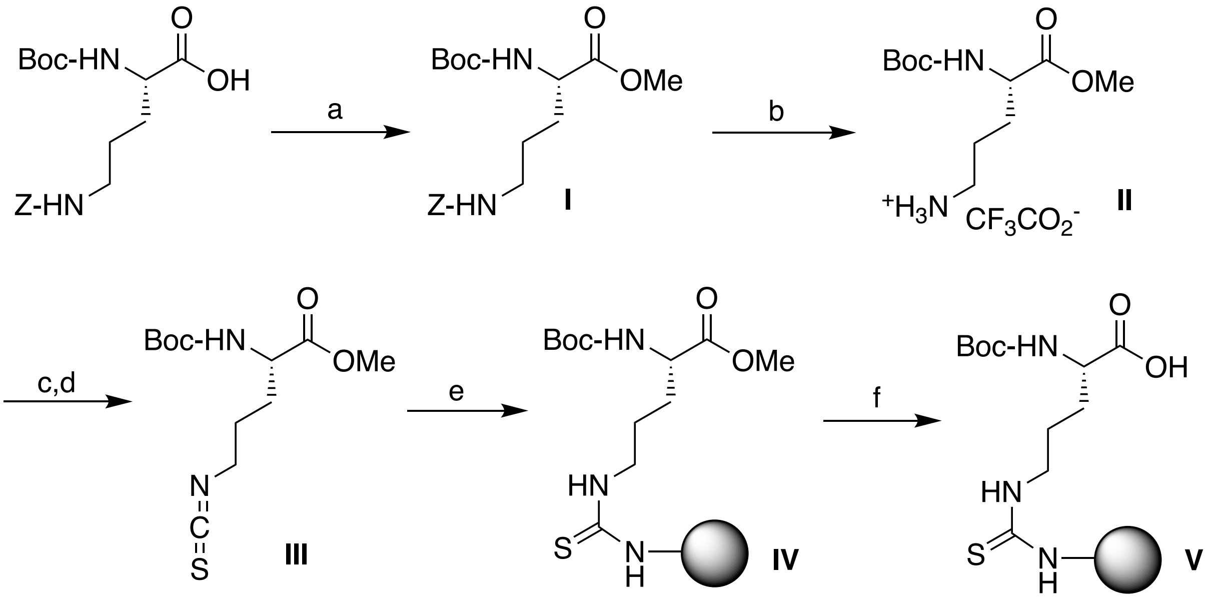

Scheme S1. Synthesis of the supported thiocitrulline intermediate **V**: (a) MeI, Na_2CO_3, DMF, rt, 5h; (b) H_2, 10% Pd/C, EtOH, 1.5 equiv. TFA, 4h; (c) CS_2, TEA, THF, 0 °C, 45 min; (d) H_2O_2, 30 min addition, then 5 min, 0 °C; (e) 3 equiv. **III**, Rink amide solid support, TEA, THF, 60 °C, 3h; (f) 5 equiv. LiOH, THF/water (7/3), rt, overnight.

The solid-supported intermediate **V** was synthesized as previously reported [Touati-Jallabe Y. et al. 2020, ref. 48] (Scheme 1S). The methyl ester **I** was obtained by reaction of Boc-Orn(Z)-OH with MeI, and then treated with hydrogen in the presence of 10% Pd/C and 1.5 equiv. TFA (in order to trap the free δ-amino group and prevent its intramolecular reaction with the methyl ester) to remove the Z protecting group, yielding the TFA salt **II**. **II** was treated with CS_2 followed by desulfurization with H_2O_2 to afford the isothiocyanate **III**, which was then loaded onto a Rink amide solid support. The completion of the reaction was assessed using the TNBS (trinitrobenzenesulfonic acid) test. The resulting solid-supported thiocitrulline derivative **IV** was finally saponified with LiOH in a mixture of THF/H_2O (7/3), giving the intermediate **V**.

2- Synthesis of 1-Fmoc-protected 3-amino-piperidines (*S*) and (*R*), 3 and 4:

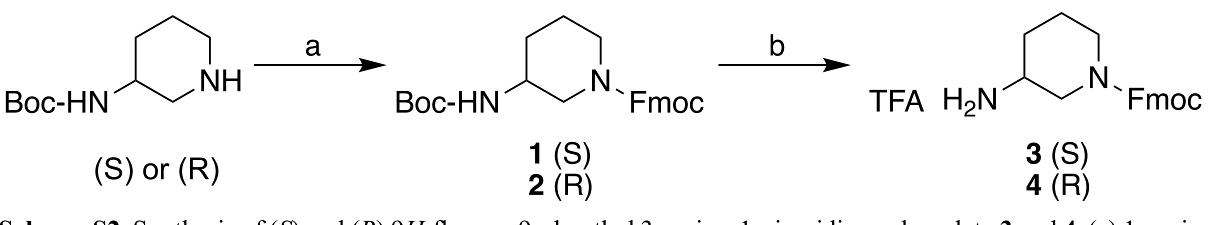

Scheme S2. Synthesis of (*S*) and (*R*) 9*H*-fluoren-9-ylmethyl 3-amino-1-piperidinecarboxylate **3** and **4**: (a) 1 equiv. Fmoc-Cl, 2.5 equiv. Na_2CO_3, dioxane/water (1:1), rt, 4h; (b) DCM/TFA (1:1), rt, 1h.

The (*S*) isomer **3** was synthesized as follows (same protocol for the (*R*) isomer **4**).

- To a solution of (*S*)-3-(Boc-amino)piperidine (500 mg, 2.5 mmol) in dioxane (10 mL), were added a solution of Na_2CO_3 (663 mg, 6.25 mmol) in water (15 mL) and a solution of Fmoc-Cl (647 mg, 2.5 mmol) in dioxane (8 mL). The resulting white suspension was stirred at rt for 4 h. 10 mL water were then added and the aqueous phase was extracted with AcOEt (3 x 10 mL). The organic pahses were mixed, washed with saturated aqueous $NaHCO_3$, water and brine, dried over Na_2SO_4, filtered and concentrated under vacuum to afford the intermediate **1** as a white solid (1.02 g, 97%).

- *(S) 9H-Fluoren-9-ylmethyl 3-(tert-butyloxycarbonylamino)-1-piperidinecarboxylate,* **1**.
White solid, 1.02 g (97%); LC-MS t_R: 2.10 min; *m/z* (ES+) 423.2 ($M+H^+$); ^1H NMR (400 MHz, DMSO-d_6): 1.40 (s, 9H), 1.58-1.78 (m, 2H), 2.73 (m, 1H), 3.21 (bs, 1H), 3.61-3.99 (m, 2H), 4.25-4.40 (m, 3H), 6.85-6.91 (m, 1H), 7.31-7.36 (m, 2H), 7.42 (td, J = 1.2, 7.4 Hz, 2H), 7.64 (m, 2H), 7.89 (d, J = 7.4 Hz, 2H).

- *(R) 9H-Fluoren-9-ylmethyl 3-(tert-butyloxycarbonylamino)-1-piperidinecarboxylate,* **2**.
White solid, 443 mg (99%); LC-MS t_R: 2.10 min; *m/z* (ES+) 423.2 ($M+H^+$); ^1H NMR (400 MHz, DMSO-d_6): 1.40 (s, 9H), 1.57-1.78 (m, 2H), 2.73 (m, 1H), 3.21 (m, 1H), 3.61-3.98 (m, 2H), 4.25 (m, 2H), 4.32-4.40 (m, 1H), 6.85-6.91 (m, 1H), 7.32-7.36 (m, 2H), 7.42 (td, J = 1.2, 7.5 Hz, 2H), 7.64 (m, 2H),7.89 (d, J = 7.4 Hz, 2H).

- Compounds **1** and **2** were dissolved in DCM/TFA (1:1, 30 mL) and the mixtures were stirred at rt for 1 h and evaporated. The residues were freeze-dried and triturated into diethyl ether. The white solids were filtered, washed with the same solvent and dried to yield compounds **3** and **4**, respectively.

(S) 9H-Fluoren-9-ylmethyl 3-amino-1-piperidinecarboxylate, **3**.
White solid, 994 mg (95%); LC-MS t_R: 1.29 min; *m/z* (ES+) 323.3 ($M+H^+$); ^1H NMR (400 MHz, DMSO-d_6): 1.34 (bs, 1H), 1.49-1.53 (m, 1H), 1.67 (m, 1H), 1.92-1.96 (m, 1H), 2.92-3.08 (m, 3H), 3.56 (bs, 1H), 4.00 (bs, 1H), 4.25-4.40 (m, 3H), 7.31-7.37 (m, 2H), 7.40-7.45 (m, 2H), 7.63 (m, 2H), 7.89 (d, J = 7.5 Hz, 2H), 8.10 (bs, 3H); ^{13}C NMR (100 MHz, DMSO-d_6): 22.1, 27.7, 43.2, 45.9, 46.0, 46.7, 66.9, 120.2, 125.1, 127.1, 127.7, 140.8, 143.7, 154.5.

(R) 9H-Fluoren-9-ylmethyl 3-amino-1-piperidinecarboxylate, **4**.
White solid, 383 mg (84%); LC-MS t_R: 1.27 min; *m/z* (ES+) 323.3 ($M+H^+$); ^1H NMR (400 MHz, DMSO-d_6): 1.32 (m, 1H), 1.49 (bs, 1H), 1.67 (bs, 1H), 1.93 (bs, 1H), 2.96-3.16 (m, 3H), 3.54-3.67 (m, 1H), 3.97 (bs, 1H), 4.26-4.42 (m, 3H), 7.32-7.37 (m, 2H), 7.43 (t, J = 7.4 Hz, 2H), 7.64 (m, 2H), 7.90 (d, J = 7.5 Hz, 2H), 8.03 (bs, 3H); ^{13}C NMR (100 MHz, DMSO-d_6): 22.1, 27.7, 43.2, 45.9, 46.0, 46.7, 66.7, 120.2, 125.1, 127.2, 127.7, 140.8, 143.7, 154.4.

3- Synthesis of biphenylaldehydes, 5a-d:

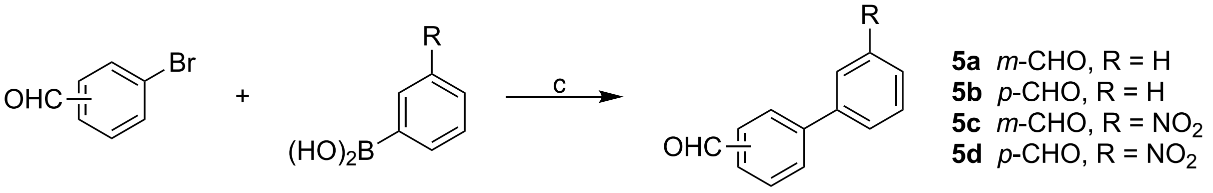

Scheme S3. Synthesis of biphenylaldehydes **5a-d**: (a) 0.03 Equiv. $Pd(OAc)_2$, 0.15 equiv. PPh_3, EtOH/toluene (1:1), benzaldehyde, 2.5 equiv. Na_2CO_3, 1.3 equiv. boronic acid, 100 °C, 15h.

The compounds were synthesized from 3- or 4-bromobenzaldehydes and phenyl- or 3-nitrophenyl-boronic acids.

$Pd(OAc)_2$ (0.03 equiv.) and PPh_3 (0.15 equiv.) were stirred in EtOH/toluene (1:1, 2.5 mL/mmol) for 10 min at rt. The aldehyde, Na_2CO_3 (2.5 equiv.) and the boronic acid (1.3 equiv.) were then added and the mixture was warmed at 100 °C for 15 h. After cooling, the mixture was diluted in water (5 mL/mmol) and extracted three times with AcOEt. The organic phases were mixed, dried over $MgSO_4$, filtered and evaporated under vacuum. The residue was finally purified on a silica gel column to afford **5a-d**.

[1,1'-Biphenyl]-3-carboxaldehyde, **5a**.
Grey solid, 193 mg (98%); LC-MS t_R: 1.84 min; *m/z* (ES+) 183.2 (M+H$^+$); ^{1}H NMR (400 MHz, $CDCl_3$): 7.38-7.43 (m, 1H), 7.46-7.50 (m, 2H), 7.59-7.65 (m, 3H), 7.86 (dd, J = 2.0, 7.6 Hz, 2H), 8.11 (t, J = 1.5 Hz, 1H), 10.09 (s, 1H); ^{13}C NMR (100 MHz, $CDCl_3$): 127.1, 127.9, 128.1, 128.5, 128.9, 129.4, 133.0, 136.9, 139.6, 142.1, 192.2.

[1,1'-Biphenyl]-4-carboxaldehyde, **5b**.
Grey solid, 382 mg (78%); LC-MS t_R: 1.83 min; *m/z* (ES+) 183.2 (M+H$^+$); ^{1}H NMR (400 MHz, $CDCl_3$): 7.38-7.53 (m, 3H), 7.61-7.67 (m, 2H), 7.76 (d, J = 8.2 Hz, 2H), 7.96 (d, J = 8.4 Hz, 2H), 10.07 (s, 1H); ^{13}C NMR (100 MHz, $CDCl_3$): 120.1, 127.5, 127.9, 128.6, 129.2, 130.4, 147.4, 192.1.

3'-Nitro-[1,1'-biphenyl]-3-carboxaldehyde, **5c**.
Yellow solid, 476 mg (77%); LC-MS t_R: 1.78 min; *m/z* (ES+) 228.2 (M+H$^+$); ^{1}H NMR (400 MHz, $CDCl_3$): 7.70 (q, J = 8.0 Hz, 2H), 7.90-8.01 (m, 3H), 8.16 (t, J = 1.7 Hz, 1H), 8.27 (ddd, J = 1.0, 2.2, 8.1 Hz, 1H), 8.50 (t, J = 1.8 Hz, 1H), 10.14 (s, 1H); ^{13}C NMR (100 MHz, $CDCl_3$): 122.0, 122.8, 127.9, 129.9, 130.0, 130.1, 132.9, 133.1, 137.2, 139.7, 141.4, 148.8, 191.8.

3'-Nitro-[1,1'-biphenyl]-4-carboxaldehyde, **5d**.
Yellow solid, 458 mg (74%); LC-MS t_R: 1.76 min; *m/z* (ES+) 228.2 (M+H$^+$); ^{1}H NMR (400 MHz, $CDCl_3$): 7.68 (t, J = 8.1 Hz, 1H), 7.81 (dt, J = 1.7, 8.3 Hz, 2H), 7.97 (ddd, J = 1.0, 2.0, 7.6 Hz, 1H), 8.02 (dt, J = 1.7, 8.6 Hz, 2H), 8.28 (ddd, J = 1.0, 2.2, 8.1 Hz, 1H), 8.50 (t, J = 1.7 Hz, 1H), 10.10 (s, 1H); ^{13}C NMR (100 MHz, $CDCl_3$): 122.2, 123.1, 127.9, 130.1, 130.5, 133.2, 136.1, 141.4, 144.4, 148.8, 191.6.

4- Synthesis of the aloc-protected *N*-hydroxy-propanimidamide, 6:

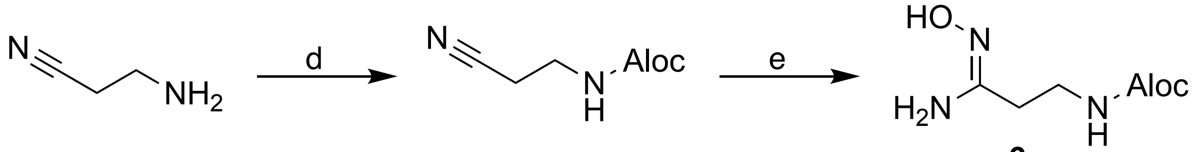

6

Scheme S4. Synthesis of 3-(*N*-allyloxycarbonyl-amino)-N-hydroxy-propanimidamide **6**: (a) 1.2 equiv. Aloc-Cl, 2.0 equiv. Na_2CO_3, dioxane/water (1:1), rt, 15h; (b) 5 equiv. NH_2OH (50% in water), EtOH, 75 °C, 15h.

A solution of 3-aminopropionitrile (2.5 g, 35.7 mmol) and Na_2CO_3 (7.56 g, 71.3 mmol) in water (125 mL) was cooled to 0 °C and Aloc-Cl (5.16 g, 42.8 mmol) was added. The mixture was stirred at rt for 15 h and extracted with AcOEt. The aqueous phase was adjusted to pH 7 with AcOH and extracted again with AcOEt. The organic phases were mixed, dried over Na_2SO_4, filtered and evaporated to yield the protected 3-aminopropionitrile as a colourless liquid, which was used in the following step without further purification.
The preceding compound was diluted in EtOH (125 mL) and a 50% aqueous solution of hydroxylamine (10.1 mL, 171 mmol) was added. The mixture was heated at 75 °C for 15 h and cooled to rt. The mixture was concentrated under vacuum and freeze-dried. The crude product was purified on a silica gel column (5-10% MeOH in DCM) to yield 5 as a grey solid (4.60 g, 69%).

3-(N-Allyloxycarbonyl-amino)-N-hydroxy-propanimidamide, **6**.
Grey solid, 4.60 g (69%); LC-MS t_R: 0.27 min; *m/z* (ES+) 188.1 (M+H$^+$); ^{1}H NMR (300 MHz, $CDCl_3$): 2.36 (t, J = 6.4 Hz, 2H), 3.42 (q, J = 6.4 Hz, 2H), 4.55 (d, J = 5.5 Hz, 2H), 4.69 (s, 2H), 5.18-5.32 (m, 2H), 5.42 (bs, 1H), 5.84-5.97 (m, 1H); ^{13}C NMR (75 MHz, $CDCl_3$): 31.9, 37.9, 65.8, 117.9, 133.0, 152.4, 156.5.

5- Characterization data for dipeptides.

The piperidine moiety of these compounds shows conformational equilibrium, explaining splitted and/or broad signals. In some cases, NMR spectra were also recorderd at high temperature (indicated in bold, as well as on the spectra copies). The two quadruplets at 117.0 and 158.2 ppm on the ^{13}C NMR spectra are due to the TFA salt of these compounds.

Ethyl *N*-{(*4S*)-4-amino-5-[(*S*)-(1-benzyl-piperidin-3-yl)amino]-5-oxopentyl}-imidothiocarbamate: 7.

Obtained after HPLC purification: white powder, 20 mg (19%)[a].

HPLC (Gr3) t_R 0.78 min; LC-MS t_R: 0.58 min; *m/z* (ES+) 392.2 [M + H]$^+$; ^{1}H NMR (500 MHz, DMSO-d_6): two conformers: 1.25 (t, J = 7.3 Hz, 3H), 1.41-1.49 (m, 1H), 1.53-1.65 (m, 2H), 1.70-1.79 (m, 2H), 1.84-1.92 (m, 2H), 2.74-2.96 (m, 2H), 3.22-3.26 (m, 1H), 3.30 (q, J = 7.3 Hz, 2H), 3.34-3.37 (m, 1H), 3.43 (m, 3H), 3.79-3.87 (m, 1H), 4.16-4.21 (m, 1H), 4.30-4.40 (bs, 2H), 7.43-7.46 (m, 3H), 7.62-7.69 (m, 2H), 8.40 (m, 3H), 9.23 & 9.30 (2d, J = 7.1 & 7.6 Hz, 1H), 9.42 & 9.46 (2bs, 2H), 9.87 & 9.99 (2t, J = 5.4 & 5.6 Hz, 1H); ^{13}C NMR (125 MHz, DMSO-d_6): 14.2/14.3, 20.8, 22.8/23.0, 25.0/25.4, 27.9, 28.0, 42.6, 43.6, 50.5/51.0, 51.3/51.7, 52.8/53.3, 58.3/59.0, 128.8, 129.6, 129.6, 131.5/131.6, 165.5/165.7, 168.0/168.1; HRMS (ESI+) calcd for $C_{20}H_{34}N_5OS^+$ (M + H)$^+$ 392.2479, found 392.2481 .

Ethyl *N*-{(*4S*)-4-amino-5-[(*R*)-(1-benzyl-piperidin-3-yl)amino]-5-oxopentyl}-imidothiocarbamate: 8.

Obtained after HPLC purification: white powder, 21 mg (20%)[a].

HPLC (Gr3) t_R 0.81 min; LC-MS t_R: 0.51 min; *m/z* (ES+) 392.3 [M + H]$^+$; ^{1}H NMR (500 MHz, DMSO-d_6): 1.23-1.28 (m, 3H), 1.32-1.44 (m, 1H), 1.48-1.55 (m, 2H), 1.65-1.93 (m, 5H), 2,68 (m, 1H), 2.90 (m, 1H), 3.15-3.22 (m, 2H), 3.32 (m, 3H), 3.70 (bs, 1H), 3.99 (bs, 1H), 4.38 (m, 1H), 7.48 (m, 4H), 8.23 bs, 3H), 8.82 (m, 1H), 9.19 (bs, 1H), 9.26 (bs, 1H), 9.64 (bs, 1H), 10.19 (bs, 1H); ^{1}H NMR (500 MHz, DMSO-d_6, **373K**): 1.28-1.32 (m, 3H), 1.36-1.46 (m, 1H), 1.57-1.64 (m, 2H), 1.69-1.79 (m, 3H), 1.82 (m, 2H), 3.07 (m, 2H), 3.18-3.24 (m, 2H), 3.34-3.38 (m, 3H), 3.73-3.76 (bs, 1H), 3.98 (m, 2H), 7.39-7.44 (m, 4H), 8.38 (bs, 1H), 8.78 (bs, 3H); ^{13}C NMR (125 MHz, DMSO-d_6): 14.2, 21.1, 23.1, 25.2, 27.8, 28.2, 42.8, 43.7, 51.2, 51.8, 53.2, 59.3, 129.0, 129.5, 129.8, 131.4, 165.9, 168.1; HRMS (ESI+) calcd for $C_{20}H_{34}N_5OS^+$ (M + H)$^+$ 392.2479, found 392.2481 .

Ethyl *N*-{(*4S*)-4-amino-5-[(*S*)-[1-(2-hydroxyphenylmethyl)-piperidin-3-yl]amino]-5-oxopentyl}-imidothiocarbamate: 9.

Obtained after HPLC purification: white powder, 21 mg (20%)[a].

HPLC (Gr3) t_R 0.97 min; LC-MS t_R: 0.50 min; *m/z* (ES+) 408.2 [M + H]$^+$; ^{1}H NMR (500 MHz, DMSO-d_6, **373K**): 1.31 (t, J = 7.3 Hz, 3H), 1.44-1.51 (m, 1H), 1.58-1.64 (m, 2H), 1.69-1.80 (m, 3H), 1.88 (m, 2H), 2.61 (m, 1H), 2.72 (bs, 1H), 3.12 (bs, 2H), 3.22 (q, J = 7.3 Hz, 2H), 3.36 (t, J = 7.0 Hz, 2H), 3.77 (t, J = 6.7 Hz, 1H), 4.02 (bs, 1H), 4.08 (bs, 2H), 6.84 (t, J = 7.4 Hz, 1H), 6.91 (d, J = 8.1 Hz, 1H), 7.24 (t, J = 7.7 Hz, 1H), 7.28 (d, J = 7.6 Hz, 1H), 8.42 (bs, 2H), 8.72 (bs, 3H); ^{13}C NMR (125 MHz, DMSO-d_6, **373K**): 13.5, 21.0, 22.8, 25.1, 27.4, 27.8, 42.8, 44.2, 51.2, 51.7, 54.6, 55.7, 115.4, 118.7, 129.8, 131.2, 156.3, 167.2; HRMS (ESI+) calcd for $C_{20}H_{34}N_5O_2S^+$ (M + H)$^+$ 408.2428, found 408.2431.

Ethyl *N*-{(*4S*)-4-amino-5-[(*S*)-[1-(2,5-dihydroxyphenylmethyl)-piperidin-3-yl]amino]-5-oxopentyl}-imidothiocarbamate: 10.

Obtained after HPLC purification: white powder, 19 mg (17%)[a].

HPLC (Gr3) t_R 0.90 min; LC-MS t_R: 0.28 min; *m/z* (ES+) 424.2 [M + H]$^+$; ^{1}H NMR (500 MHz, DMSO-d_6): 1.27 (t, J = 7.3 Hz, 3H), 1.37-1.45 (m, 1H), 1.50-1.56 (2H), 1.66-1.71 (m, 3H), 1.82-1.91 (m, 2H), 2.72 (bs, 1H), 2.90 (bs, 1H), 3.19 (q, J = 7.3 Hz, 2H), 3.33 (m, 4H), 3.75 (bs, 1H), 4.03 (bs, 1H), 4.16 (bs, 2H), 6.71-6.78 (m, 3H), 8.25 (bs, 3H), 8.79 (bs, 1H), 9.08 (bs, 1H), 9.21 (bs, 1H), 9.27 (bs, 1H), 9.60 (m, 3H); ^{1}H NMR (500 MHz, DMSO-d_6, **373K**): 1.31 (t, J = 7.3 Hz, 3H), 1.43-1.50 (m, 1H), 1.58-1.65 (2H), 1.71-1.81 (m, 3H), 1.84-1.91 (m, 2H), 2.61 (bs, 1H), 2.71 (bs, 1H), 3.13 (bs, 2H), 3.21 (q, J = 7.3 Hz, 2H), 3.36 (t, J = 7.3 Hz, 2H), 3.76 (t, J = 6.7 Hz, 1H), 4.00 (bs, 3H), 6.67-6.73 (m, 3H), 8.41 & 8.72 (bs, 6H);^{13}C NMR (125 MHz, DMSO-d_6): 14.2, 21.1, 23.1, 25.3, 27.4, 28.3, 42.9, 43.8, 50.9, 51.8, 54.1, 54.9, 116.0, 116.4, 118.1, 119.0, 149.2, 149.8, 165.9, 168.3; HRMS (ESI+) calcd for $C_{20}H_{34}N_5O_3S^+$ (M + H)$^+$ 424.2377, found 424.2380.

Ethyl *N*-{(*4S*)-4-amino-5-[(*R*)-[1-(2,5-dihydroxyphenylmethyl)-piperidin-3-yl]amino]-5-oxopentyl}-imidothiocarbamate: 11.

Obtained after HPLC purification: white powder, 25 mg (23%)[a].

HPLC (Gr3) t_R 0.80 min; LC-MS t_R: 0.49 min; *m/z* (ES+) 424.2 [M + H]$^+$; ^{1}H NMR (500 MHz, DMSO-d_6): 1.25 (t, J = 7.3 Hz, 3H), 1.36-1.43 (m, 1H), 1.48-1.55 (m, 2H), 1.66-1.70 (m, 3H), 1.79-1.90 (m, 2H), 2.70 (m, 1H), 2.95 (m, 1H), 3.18 (q, J = 7.3 Hz, 2H), 3.32 (m, 3H), 3.71 (bs, 1H), 4.05 (bs, 1H), 4.16-4.25 (m, 2H), 6.71-6.78 (m, 3H), 8.24 (bs, 3H), 8.79 (bs, 1H), 9.09 (bs, 1H), 9.20 (bs, 1H), 9.27 (bs, 1H), 9.63 (bs, 3H); ^{1}H NMR (500 MHz, DMSO-d_6, **373K**): 1.30 (t, J = 7.2 Hz, 3H), 1.38-1.46 (m, 1H), 1.58-1.64 (m, 2H), 1.74-1.78 (m, 3H), 1.83-1.89 (m, 2H), 2.75 (bs, 2H), 3.21 (q, J = 7.3 Hz, 2H), 3.35 (t, J = 6.9 Hz, 2H), 3.73 (t, J = 6.3 Hz, 1H),

4.00-4.09 (m, 3H), 6.67-6.74 (m, 3H), 8.44 (bs, 2H), 8.76 (bs, 3H); ^{13}C NMR (125 MHz, DMSO-d_6): 14.2, 21.1, 23.1, 25.2, 27.8, 28.2, 42.9, 43.6, 51.5, 51.9, 53.6, 54.8, 116.0, 116.4, 118.1, 119.0, 149.2, 149.8, 165.9, 168.1; HRMS (ESI+) calcd for $C_{20}H_{34}N_5O_3S^+$ $(M + H)^+$ 424.2377, found 424.2381.

Ethyl *N*-{(*4S*)-4-amino-5-[(*S*)-[1-(2-nitrophenylmethyl)-piperidin-3-yl]amino]-5-oxopentyl}-imidothiocarbamate: 12.

Obtained after HPLC purification: yellowish powder, 7 mg (6.3%)[a].

HPLC (Gr3) t_R 0.93 min; LC-MS t_R: 0.63 min; *m/z* (ES+) 437.2 $[M + H]^+$; ^{1}H NMR (400 MHz, DMSO-d_6): 1.27 (t, J = 7.2 Hz, 3H), 1.53-1.59 (m, 3H), 1.69-1.74 (m, 3H), 1.85-1.93 (m, 2H), 3.03 (m, 1H), 3.19 (q, J = 7.3 Hz, 2H), 3.28-3.36 (m, 4H), 3.78 (m, 1H), 4.03 (bs, 1H), 4.57 (bs, 2H), 7.76-7.95 (m, 3H), 8.27 (m, 4H), 8.82 (bs, 1H), 9.25 (m, 2H), 9.67 (s, 1H); HRMS (ESI+) calcd for $C_{20}H_{33}N_6O_3S^+$ $(M + H)^+$ 437.2329, found 437.2339.

Ethyl *N*-{(*4S*)-4-amino-5-[(*R*)-[1-(2-nitrophenylmethyl)-piperidin-3-yl]amino]-5-oxopentyl}-imidothiocarbamate: 13.

Obtained after HPLC purification: yellowish powder, 37 mg (34%)[a].

HPLC (Gr3) t_R 0.78 min; LC-MS t_R: 0.51 min; *m/z* (ES+) 437.2 $[M + H]^+$; ^{1}H NMR (500 MHz, DMSO-d_6): 1.25 (t, J = 7.3 Hz , 3H), 1.36- 1.45 (m, 1H), 1.58-1.64 (m, 2H), 1.67-1.70 (m, 1H), 1.80 (bs, 2H), 3.18 (q, J = 7.3 Hz , 2H), 3.21-3.28 (m, 1H), 3.32 (q, J = 6.7 Hz , 2H), 3.71 (bs, 2H), 3.94 (bs, 5H), 4.65 (bs, 1H), 7.75 (m, 3H), 8.24 (bs, 4H), 9.20 (bs, 1H), 9.28 (bs, 1H), 9.65 (m, 1H); ^{1}H NMR (500 MHz, DMSO-d_6, **373K**): 1.30 (t, J = 7.3 Hz , 3H), 1.46- 1.57 (m, 1H), 1.58-1.64 (m, 2H), 1.66-1.71 (m, 1H), 1.74-1.80 (m, 2H), 2.16 (m, 1H), 2.27 (1H), 2.72 (m, 1H), 2.84 (m, 1H), 3.21 (q, J = 7.3 Hz , 2H), 3.36 (t, J = 6.9 Hz , 2H), 3.73 (t, J = 6.4 Hz , 1H), 3.80 (m, 1H), 3.85-3.96 (m, 2H), 4.17 (bs, 2H), 755-7.58 (m, 1H), 7.68 (m, 2H), 7.90 (d, J = 8.0 Hz, 1H), 8.11 (m, 1H), 9.00 (bs, 3H); ^{13}C NMR (125 MHz, DMSO-d_6): 14.2, 23.1, 24.9, 25.2, 28.2, 42.9, 43.3, 51.9, 52.6, 54.7, 57.7, 125.1, 125.4, 131.6, 134.4, 134.6, 149.0, 165.9, 168.0; ^{13}C NMR (125 MHz, DMSO-d_6, **373K**): 13.5, 22.4, 22.8, 25.1, 27.9, 28.8, 42.8, 45.4, 51.7, 52.3, 56.8, 57.4, 123.7, 128.5, 131.2, 132.3, 149.3, 166.0, 167.1; HRMS (ESI+) calcd for $C_{20}H_{33}N_6O_3S^+$ $(M + H)^+$ 437.2329, found 437.2337.

Ethyl *N*-{(*4S*)-4-amino-5-[(*S*)-[1-(2-hydroxy-5-nitro-phenylmethyl)-piperidin-3-yl]amino]-5-oxopentyl}-imidothiocarbamate: 14.

Obtained after HPLC purification: yellowish powder, 11 mg (9%)[a].

HPLC (Gr3) t_R 1.00 min; LC-MS t_R: 0.66 min; m/z (ES+) 453.3 $[M + H]^+$; ^1H NMR (400 MHz, DMSO-d_6): 1.26 (t, J = 7.3 Hz , 3H), 1.40-1.50 (m, 1H), 1.51-1.59 (m, 2H), 1.68-1.74 (m, 3H), 1.83-1.92 (m, 2H), 2.81 (bs, 1H), 2.95 (bs, 1H), 3.19 (q, J = 7.3 Hz, 2H), 3.28-3.35 (m, 3H), 3.42 (bs, 1H), 3.77 (bs, 1H), 4.04 (bs, 1H), 4.30-4.38 (m, 2H), 7.15 (d, J = 9.1 Hz, 1H), 8.21 (dd, J = 2.9, 9.1 Hz, 1H), 8.29 (bs, 3H), 8.41 (d, J = 2.9 Hz, 1H), 8.82 (d, J = 7.5 Hz, 1H), 9.28 (bs, 2H), 9.66 (bs, 1H); ^{13}C NMR (100 MHz, DMSO-d_6): 14.2, 21.2, 23.1, 25.2, 27.3, 28.2, 42.9, 43.8, 51.0, 51.8, 53.6, 54.4, 116.2, 118.5, 127.3, 129.4, 139.3, 163.5, 166.0, 168.2; HRMS (ESI+) calcd for $C_{20}H_{33}N_6O_4S^+$ $(M + H)^+$ 453.2279, found 453.2285.

Ethyl *N*-{(*4S*)-4-amino-5-[(*S*)-[1-(5-hydroxy-2-nitro-phenylmethyl)-piperidin-3-yl]amino]-5-oxopentyl}-imidothiocarbamate: 15.

Obtained after HPLC purification: yellowish powder, 22 mg (19%)[a].

HPLC (Gr3) t_R 0.83 min; LC-MS t_R: 0.56 min; m/z (ES+) 453.2 $[M + H]^+$; ^1H NMR (400 MHz, DMSO-d_6): 1.26 (t, J = 7.3 Hz, 3H), 1.38-1.45 (m, 1H), 1.55-1.59 (m, 2H), 1.69-1.73 (m, 3H), 1.85 (m, 2H), 3.04 (m, 1H), 3.19 (q, J = 7.3 Hz, 2H), 3.34 (m, 3H), 3.77 (bs, 2H), 3.99 (bs, 1H), 4.52 (bs, 2H), 6.99-7.06 (m, 1H), 7.10 (d, J = 2.6 Hz, 1H), 8.27 (bs, 4H), 8.84 (bs, 1H), 9.21 (bs, 1H), 9.26 (bs, 1H), 9.64 (bs, 1H); ^1H NMR (500 MHz, DMSO-d_6, **373K**): 1.30 (t, J = 7.3 Hz, 3H), 1.27-1.36 (m, 1H), 1.55-1.64 (m, 3H), 1.71-1.78 (m, 3H), 1.80-1.83 (m, 1H), 2.69 (bs, 1H), 2.87 (bs, 1H), 3.20 (q, J = 7.3 Hz, 2H), 3.35 (m, 4H), 3.76 (t, J = 6.4 Hz, 1H), 3.87 (bs, 3H), 6.85-6.88 (m, 1H), 7.09 (d, J = 2.7 Hz, 1H), 7.91 (d, J = 8.9 Hz, 1H), 8.09 (bs, 1H); ^{13}C NMR (125 MHz, DMSO-d_6, **373K**): 13.5, 22.4, 22.8, 25.1, 27.9, 28.7, 42.8, 45.6, 51.8, 52.2, 57.3, 58.1, 114.2, 114.3, 117.1, 117.2, 127.0, 140.5, 161.6, 167.1; HRMS (ESI+) calcd for $C_{20}H_{33}N_6O_4S^+$ $(M + H)^+$ 453.2279, found 453.2286.

Ethyl *N*-{(*4S*)-4-amino-5-[(*S*)-[1-(3,4,5-trimethoxy-phenylmethyl)-piperidin-3-yl]amino]-5-oxopentyl}-imidothiocarbamate: 16.

Obtained after HPLC purification: white powder, 15 mg (12%)[a].

HPLC (Gr3) t_R 1.00 min; LC-MS t_R: 0.63 min; m/z (ES+) 482.2 $[M + H]^+$; ^1H NMR (500 MHz, DMSO-d_6): 1.24-1.27 (m, 3H), 1.34-1.43 (m, 1H), 1.54 (m, 2H), 1.67-1.71 (m, 3H), 1.85 (m, 2H), 2.84 (m, 1H), 3.03 (m, 1H), 3.19 (q, J = 7.4 Hz, 2H), 3.33 (bs, 3H), 3.66 (s, 3H), 3.78 (s, 6H), 4.03-4.31 (m, 2H), 6.61-6.87 (m, 2H), 8.25 (bs, 3H), 8.87 (m, 1H), 9.26 (bs, 2H), 9.69 (m, 1H); ^1H NMR (500 MHz, DMSO-d_6, **373K**): 1.30 (m, 3H), 1.41-1.47 (m, 1H), 1.59-1.66 (m, 2H), 1.69-1.80 (m, 3H), 1.85 (m, 2H), 2.98 (m, 1H), 3.13 (m, 1H), 3.19-3.24 (m, 2H), 3.35 (m, 3H), 3.71 (s, 3H), 3.81 (s, 6H), 3.76-3.88 (m, 2H), 4.00 (bs, 2H), 6.77 (m, 2H), 8.44 (bs, 2H), 8.66 (bs, 1H); ^{13}C NMR (125 MHz, DMSO-d_6): 14.2, 21.1, 23.1, 25.2, 27.7, 28.3, 42.9, 43.9,

50.6, 51.8, 53.9, 55.9, 60.1, 108.5, 125.0, 138.2, 152.9, 165.9, 168.1; HRMS (ESI+) calcd for $C_{23}H_{40}N_5O_4S^+$ (M + H)$^+$ 482.2796, found 482.2799.

Ethyl *N*-{(*4S*)-4-amino-5-[(*S*)-[1-(4-chloro-3-trifluoromethyl-phenylmethyl)-piperidin-3-yl]amino]-5-oxopentyl}-imidothiocarbamate: 17.

Obtained after HPLC purification: white powder, 12 mg (10%)[a].

HPLC (Gr3) t_R 1.25 min; LC-MS t_R: 0.79 min; *m/z* (ES+) 494.2 + 496.1 [M + H]$^+$; ^{1}H NMR (500 MHz, DMSO-d_6): 1.26 (t, *J* = 7.3 Hz, 3H), 1.37-1.45 (m, 1H), 1.52-1.57 (m, 2H), 1.67-1.73 (m, 3H), 1.84 (m, 2H), 3.20 (q, *J* = 7.3 Hz, 2H), 3.33 (m, 3H), 3.76 (m, 1H), 3.98 (m, 1H), 4.41 (m, 2H), 7.80 (bs, 2H), 8.02 (bs, 1H), 8.24 (bs, 3H), 8.79 (bs, 1H), 9.25 (m, 2H), 9.67 (bs, 1H); (500 MHz, DMSO-d_6, **373K**): 1.30 (t, *J* = 7.3 Hz, 3H), 1.37-1.47 (m, 1H), 1.59-1.65 (m, 3H), 1.71-1.85 (m, 4H), 2.82 (bs, 1H), 2.99 (bs, 1H), 3.22 (q, *J* = 7.3 Hz, 2H), 3.36 (t, *J* = 7.1 Hz, 3H), 3.80 (t, *J* = 6.5 Hz, 1H), 3.91 (m, 3H), 7.70 (bs, 2H), 7.86 (bs, 1H), 8.35 (bs, 1H), 8.92 (bs, 2H);^{13}C NMR (125 MHz, DMSO-d_6, **373K**): 13.4, 21.7, 22.8, 25.1, 27.9, 28.1, 42.7, 45.0, 51.5, 51.7, 55.9, 59.1, 122.4 (q, *J* = 273.2 Hz), 126.4 (q, *J* = 31.6 Hz), 128.3, 129.8, 131.2, 134.6, 166.0, 167.3; HRMS (ESI+) calcd for $C_{21}H_{32}ClF_3N_5OS^+$ (M + H)$^+$ 494.1963, found 494.1972.

Ethyl *N*-{(*4S*)-4-amino-5-[(*S*)-[1-(3-chloro-4-trifluoromethyl-phenylmethyl)-piperidin-3-yl]amino]-5-oxopentyl}-imidothiocarbamate: 18.

Obtained after HPLC purification: white powder, 24 mg (13%)[a].

HPLC (Gr3) t_R 1.25 min; LC-MS t_R: 0.90 min; *m/z* (ES+) 494.2 + 496.2 [M + H]$^+$; ^{1}H NMR (500 MHz, DMSO-d_6): 1.25 (t, *J* = 7.3 Hz, 3H), 1.41 (m, 1H), 1.50-1.56 (m, 2H), 1.66-1.71 (m, 3H), 1.84 (m, 2H), 2.80 (bs, 2H), 3.18 (t, *J* = 7.3 Hz, 2H), 3.30-3.34 (m, 3H), 3.74-3.78 (m, 2H), 3.96 (bs, 1H), 4.38 (bs, 2H), 7.64 (s, 1H), 7.87-7.95 (m, 2H), 8.25 (s, 3H), 8.79 (bs, 1H), 9.22-9.28 (m, 2H), 9.67 (s, 1H); ^{13}C NMR (126 MHz, DMSO-d_6): 14.2, 21.4, 23.1, 25.2, 27.7, 28.3, 43.0, 43.3, 44.2, 51.0, 51.8, 58.0, 122.8 (q, *J* = 272.7 Hz), 128.4, 131.0, 133.9, 136.3, 165.9, 168.1, 169.8; ^{13}C NMR (125 MHz, DMSO-d_6, **373K**): 13.7, 22.1, 23.1, 25.5, 28.2, 28.4, 43.1, 45.5, 52.1, 52.2, 56.4, 59.7, 122.8 (q, *J* = 273.0 Hz), 127.8 (q, *J* = 5.3 Hz), 128.4, 130.8, 131.9, 142.5, 166.6, 167.6; HRMS (ESI+) calcd for $C_{21}H_{32}ClF_3N_5OS^+$ (M + H)$^+$ 494.1963, found 494.1970.

Ethyl *N*-{(*4S*)-4-amino-5-[(*S*)-[1-(2-trifluoromethyl-phenylmethyl)-piperidin-3-yl]amino]-5-oxopentyl}-imidothiocarbamate: 19.

Obtained after HPLC purification: white powder, 53 mg (32%)[a].

HPLC (Gr3) t_R 1.08 min; LC-MS t_R: 0.76 min; *m/z* (ES+) 460.2 $[M + H]^+$; ^{1}H NMR (500 MHz, DMSO-d_6): 1.24 (t, *J* = 7.3 Hz, 3H), 1.32-1.41 (m, 1H), 1.50-1.55 (m, 2H), 1.65-1.70 (m, 3H), 1.82 (m, 2H), 3.18 (q, *J* = 7.3 Hz, 2H), 3.26 (m, 1H), 3.32 (m, 2H), 3.77 (m, 2H), 3.94 (bs, 2H), 4.47 (bs, 2H), 7.60 (bs, 1H), 7.77 (bs, 2H), 7.90 (bs, 1H), 8.24 (bs, 3H), 8.70 (bs, 1H), 9.22 (s, 1H), 9.28 (s, 1H), 9.66 (s, 1H); ^{1}H NMR (500 MHz, DMSO-d_6, **373K**): 1.29 (t, *J* = 7.3 Hz, 3H), 1.33-1.41 (m, 1H), 1.55-1.64 (m, 3H), 1.71-1.85 (m, 4H), 2.18-2.29 (m, 2H), 2.67-2.70 (m, 1H), 2.85 (m, 1H), 3.20 (q, *J* = 7.3 Hz, 2H), 3.35 (t, *J* = 7.0 Hz, 2H), 3.76 (bs, 2H), 3.80 (t, *J* = 6.5 Hz, 1H), 3.88 (bs, 1H), 7.49 (t, *J* = 7.6 Hz, 1H), 7.66 (t, *J* = 7.6 Hz, 1H), 7.70 (d, *J* = 7.9 Hz, 1H), 7.83 (d, *J* = 7.8 Hz, 1H), 8.21 (bs, 1H), 9.02 (bs, 2H); ^{13}C NMR (125 MHz, DMSO-d_6): 14.2, 23.2, 24.1, 24.9, 25.2, 28.3, 42.9, 43.3, 51.7, 52.3, 123.1, 125.3, 126.2, 133.0, 165.9, 168.0; ^{13}C NMR (125 MHz, DMSO-d_6, **373K**): 13.4, 22.4, 22.8, 25.1, 27.9, 28.7, 42.8, 45.6, 51.7, 52.4, 57.2, 124.1 (q, *J* = 274.1 Hz), 125.4 (q, *J* = 5.7 Hz), 127.0, 127.3, 130.4, 131.9, 135.9, 166.0, 167.2; HRMS (ESI+) calcd for $C_{21}H_{33}F_3N_5OS^+$ $(M + H)^+$ 460.2352, found 460.2360.

Ethyl *N*-{(*4S*)-4-amino-5-[(*S*)-[1-(3-trifluoromethyl-phenylmethyl)-piperidin-3-yl]amino]-5-oxopentyl}-imidothiocarbamate: 20.

Obtained after HPLC purification: white powder, 25 mg (34%)[a].

HPLC (Gr3) t_R 1.15 min; LC-MS t_R: 0.73 min; *m/z* (ES+) 460.2 $[M + H]^+$; ^{1}H NMR (500 MHz, DMSO-d_6): 1.26 (t, *J* = 7.3 Hz, 3H), 1.39-1.44 (m, 1H), 1.50-1.57 (m, 2H), 1.67-1.71 (m, 3H), 1.81-1.94 (m, 2H), 2.75 (bs, 1H), 2.89 (bs, 1H), 3.19 (q, *J* = 7.3 Hz, 2H), 3.34 (m, 3H), 3.76 (bs, 1H), 3.99 (bs, 1H), 4.42 (m, 2H), 7.70-7.93 (m, 4H), 8.25 (bs, 3H), 8.86 (bs, 1H), 9.26 (m, 2H), 9.69 (bs, 1H), 10.48 (m, 1H); ^{1}H NMR (500 MHz, DMSO-d_6, **373K**): 1.30 (t, *J* = 7.3 Hz, 3H), 1.37-1.45 (m, 1H), 1.58-1.66 (m, 3H), 1.70-1.90 (m, 4H), 2.86 (bs, 1H), 3.02 (m, 1H), 3.21 (q, *J* = 7.3 Hz, 2H), 3.35 (m, 3H), 3.79 (t, *J* = 6.5 Hz, 1H), 3.94 (bs, 3H), 7.62 (m, 1H), 7.70 (m, 2H), 7.76 (bs, 1H), 8.34 (bs, 1H), 8.90 (bs, 2H); ^{13}C NMR (125 MHz, DMSO-d_6): 14.2, 21.1, 23.1, 25.2, 27.6, 28.3, 42.9, 43.9, 50.8, 51.8, 53.9, 58.6, 124.0 (q, *J* = 271.6 Hz), 126.5 (q, *J* = 11.0 Hz), 128.0, 129.4, 130.0, 130.9, 135.5, 165.9, 168.1; HRMS (ESI+) calcd for $C_{21}H_{33}F_3N_5OS^+$ $(M + H)^+$ 460.2352, found 460.2359.

Ethyl *N*-{(*4S*)-4-amino-5-[(*S*)-[1-(3-bromo-phenylmethyl)-piperidin-3-yl]amino]-5-oxopentyl}-imidothiocarbamate: 21.

Obtained after HPLC purification: white powder, 40 mg (42%)[a].

HPLC (Gr3) t_R 1.10 min; LC-MS t_R: 0.65 min; *m/z* (ES+) 470.2, 472.3 $[M + H]^+$; ^{1}H NMR (500 MHz, DMSO-d_6): 1.26 (t, *J* = 7.3 Hz, 3H), 1.37-1.43 (m, 1H), 1.49-1.56 (m, 2H), 1.67-1.71

(m, 3H), 1.84-1.91 (m, 2H), 2.72 (bs, 1H), 2.86 (bs, 1H), 3.19 (q, J = 7.3 Hz, 2H), 3.31-3.35 (m, 3H), 3.74-3.78 (m, 1H), 3.98 (bs, 1H), 4.31-4.38 (m, 2H), 7.42-7.48 (m, 2H), 7.67-7.76 (m, 2H), 8.27 (s, 3H), 8.84 (bs, 1H), 9.24-9.30 (m, 2H), 9.69 (bs, 1H), 10.30 (bs, 1H); ^{13}C NMR (125 MHz, DMSO-d_6): 14.3, 21.2, 23.1, 25.3, 27.6, 28.3, 43.0, 44.0, 50.8, 51.8, 53.9, 58.6, 122.0, 130.5, 131.0, 132.1, 132.6, 134.0, 165.9, 168.2; HRMS (ESI+) calcd for $C_{20}H_{33}BrN_5OS^+$ $(M + H)^+$ 470.1584, found 470.1590.

Ethyl *N*-{(*4S*)-4-amino-5-[(*S*)-[1-(naphth-2-yl)methyl-piperidin-3-yl]amino]-5-oxopentyl}-imidothiocarbamate: 22.

Obtained after HPLC purification: white powder, 16 mg (14%)[a].

HPLC (Gr3) t_R 1.11 min; LC-MS t_R: 0.71 min; *m/z* (ES+) 442.3 $[M + H]^+$; ^{1}H NMR (500 MHz, DMSO-d_6): 1.26 (t, J = 7.2 Hz, 3H), 1.38-1.45 (m, 1H), 1.48-1.55 (m, 2H), 1.65-1.70 (m, 3H), 1.85-1.93 (m, 2H), 2.79 (m, 1H), 2.94 (m, 1H), 3.19 (q, J = 7.1 Hz, 2H), 3.33 (m, 3H), 3.75 (m, 1H), 4.00 (bs, 1H), 4.52 (m, 2H), 7.61 (m, 3H), 7.94-8.05 (m, 4H), 8.24 (bs, 3H), 8.82 (bs, 1H), 9.23 (bs, 1H), 9.29 (bs, 1H), 9.67 (bs, 1H), 10.26 (bs, 1H); ^{1}H NMR (500 MHz, DMSO-d_6, **373K**): 1.29 (t, J = 7.3 Hz, 3H), 1.40-1.51 (m, 1H), 1.55-1.62 (m, 2H), 1.66-1.79 (m, 3H), 1.86 (m, 2H), 3.02 (m, 2H), 3.20 (m, 2H), 3.33 (m, 3H), 3.77 (t, J = 6.4 Hz, 1H), 3.98 (bs, 1H), 4.13 (bs, 2H), 7.54-7.57 (m, 3H), 7.89-7.94 (m, 4H), 8.34 (bs, 2H), 8.72 (bs, 2H); ^{13}C NMR (125 MHz, DMSO-d_6): 14.3, 21.1, 23.1, 25.2, 27.6, 28.3, 42.9, 44.0, 50.8, 51.8, 53.8, 59.6, 127.0, 127.0, 127.3, 127.8, 128.1, 128.6, 131.2, 131,3, 132.6, 133.1, 165.9, 168.2; HRMS (ESI+) calcd for $C_{24}H_{36}N_5OS^+$ $(M + H)^+$ 442.2635, found 442.2637.

Ethyl *N*-{(*4S*)-4-amino-5-[(*S*)-[1-[([1,1'-biphenyl]-4-yl)methyl]-piperidin-3-yl]amino]-5-oxopentyl}-imidothiocarbamate: 23.

Obtained after HPLC purification: white powder, 14 mg (12%)[a].

HPLC (Gr3) t_R 1.26 min; LC-MS t_R: 0.90 min; *m/z* (ES+) 468.4 $[M + H]^+$; ^{1}H NMR (500 MHz, DMSO-d_6): 1.25 (t, J = 7.3 Hz, 3H), 1.37-1.47 (m, 1H), 1.50-1.56 (m, 2H), 1.69 (m, 3H), 1.86-1.95 (m, 2H), 2.74 (m, 1H), 2.90 (m, 1H), 3.19 (q, J = 7.3, 2H), 3.34 (bs, 3H), 3.76 (bs, 1H), 4.03 (bs, 1H), 4.40 (m, 2H), 7.38-7.44 (m, 1H), 7.50 (t, J = 7.7, 2H), 7.59-7.83 (m, 6H), 8.23 (bs, 3H), 8.85 (bs, 1H), 9.26 (m, 2H), 9.69 (m, 1H), 10.16 (bs, 1H); ^{1}H NMR (500 MHz, DMSO-d_6, **373K**): 1.29 (t, J = 7.3 Hz, 3H), 1.37-1.48 (m, 1H), 1.59-1.67 (m, 3H), 1.73-1.86 (m, 4H), 3.20 (q, J = 7.3, 2H), 3.36 (t, J = 7.0 Hz, 2H), 3.78 (t, J = 6.4 Hz, 1H), 3.96 (bs, 2H), 7.37-7.53 (m, 5H), 7.67 (m, 4H), 8.33 (bs, 2H), 8.64 (bs, 2H); ^{13}C NMR (125 MHz, DMSO-d_6): 14.3, 21.1, 23.1, 25.3, 27.6, 28.2, 42.9, 43.9, 50.7, 51.8, 53.7, 59.0, 126.8, 127.1, 128.1, 128.5, 129.1,

132.1, 139.2, 141.4, 165.9, 168.2; HRMS (ESI+) calcd for $C_{26}H_{38}N_5OS^+$ $(M + H)^+$ 468.2792, found 468.2797.

Ethyl *N*-{(*4S*)-4-amino-5-[(*S*)-[1-[(3'-nitro-[1,1'-biphenyl]-4-yl)methyl]-piperidin-3-yl]amino]-5-oxopentyl}-imidothiocarbamate: 24.

Obtained after HPLC purification: yellowish powder, 27 mg (25%)[a].

HPLC (Gr3) t_R 1.27 min; LC-MS t_R: 0.94 min; *m/z* (ES+) 513.4 $[M + H]^+$; ^{1}H NMR (500 MHz, DMSO-d_6): 1.25 (t, J = 7.3 Hz, 3H), 1.36-1.48 (m, 1H), 1.02.1.56 (m, 2H), 1.67-1.72 (m, 3H), 1.86-1.94 (m, 2H), 2.76 (bs, 1H), 2.85-2.95 (m, 1H), 3.18 (q, J = 7.1 Hz, 2H), 3.34 (m, 3H), 3.77 (bs, 1H), 4.02 (bs, 1H), 4.39-4.46 (bs, 2H), 7.65 (m, 2H), 7.80 (t, J = 8.0 Hz, 1H), 7.93 (m, 2H), 8.19-8.28 (m, 5H), 8.48 (s, 1H), 8.86 (bs, 1H), 9.25 (bs, 1H), 9.30 (bs, 1H), 9.70 (bs, 1H), 10.32 (bs, 1H); ^{1}H NMR (500 MHz, DMSO-d_6, **373K**): 1.27-1.33 (m, 3H), 1.42-1.50 (m, 1H), 1.60-1.66 (m, 2H), 1.73-1.80 (m, 3H), 1.85-1.90 (m, 2H), 2.63 (bs, 1H), 3.02 (bs, 2H), 3.18-3.24 (m, 2H), 3.34-3.40 (m, 3H), 3.78-3.83 (m, 1H), 4.01 (bs, 2H), 7.57-7.62 (m, 2H), 7.76-7.85 (m, 3H), 8.13-8.26 (m, 2H), 8.44 (m, 2H), 8.64 (bs, 2H); ^{13}C NMR (125 MHz, DMSO-d_6): 14.2, 21.1, 23.1, 25.2, 27.6, 28.3, 42.9, 43.9, 50.7, 51.8, 53.8, 58.9, 121.3, 122.7, 127.5, 129.8, 130.8, 132.3, 133.4, 139.0, 140.8, 148.5, 165.9, 168.2; HRMS (ESI+) calcd for $C_{26}H_{37}N_6O_3S^+$ $(M + H)^+$ 513.2642, found 513.2651.

Ethyl *N*-{(*4S*)-4-amino-5-[(*S*)-[1-[([1,1'-biphenyl]-3-yl)methyl]-piperidin-3-yl]amino]-5-oxopentyl}-imidothiocarbamate: 25.

Obtained after HPLC purification: white powder, 3.2 mg (2.7%)[a].

HPLC (Gr3) t_R 1.26 min; LC-MS t_R: 0.89 min; *m/z* (ES+) 468.4 $[M + H]^+$; ^{1}H NMR (500 MHz, DMSO-d_6): 1.23-1.27 (m, 3H), 1.38-1.55 (m, 3H), 1.65-1.73 (m, 3H), 1.87-1.95 (m, 2H), 2.73-2.79 (m, 1H), 2.87-2.94 (m, 1H), 3.20 (m, 2H), 3.25-3.32 (m, 3H), 3.75 (bs, 1H), 4.03 (bs, 1H), 4.37-4.48 (m, 2H), 7.42 (m, 1H), 7.49-7.59 (m, 4H), 7.70 (m, 2H), 7.79 (m, 1H), 7.86 (m, 1H), 8.23 (m, 3H), 8.85 (bs, 1H), 9.23-9.27 (m, 2H), 9.61-9.68 (m, 1H), 10.18 (bs, 1H); ^{13}C NMR (126 MHz, DMSO-d_6): 14.3, 21.1, 23.1, 25.3, 27.6, 28.3, 42.9, 43.9, 50.8, 51.8, 53.9, 59.5, 126.8, 128.0, 129.1, 129.6, 129.8, 130.2, 130.4, 139.4, 140.8, 165.9, 168.2; HRMS (ESI+) calcd for $C_{26}H_{38}N_5OS^+$ $(M + H)^+$ 468.2792, found 468.2800.

Ethyl *N*-{(*4S*)-4-amino-5-[(*S*)-[1-[(3'-nitro-[1,1'-biphenyl]-3-yl)methyl]-piperidin-3-yl]amino]-5-oxopentyl}-imidothiocarbamate: 26.

Obtained after HPLC purification: yellowish powder, 14 mg (13%)[a].

HPLC (Gr3) t_R 1.28 min; LC-MS t_R: 0.90 min; *m/z* (ES+) 513.4 $[M + H]^+$; ^{1}H NMR (500 MHz, DMSO-d_6): 1.26 (t, J = 7.3 Hz, 3H), 1.43-1.47 (m, 1H), 1.51-1.55 (m, 2H), 1.66-1.71 (m, 3H),

1.87-1.95 (m, 2H), 2.77 (bs, 1H), 2.92 (bs, 1H), 3.18 (q, *J* = 7.4 Hz, 2H), 3.31 (m, 3H), 3.75 (bs, 1H), 4.02 (bs, 1H), 4.31-4.56 (m, 2H), 7.56-7.62 (m, 2H), 7.81 (t, *J* = 8.0 Hz, 1H), 7.93-7.98 (m, 2H), 8.18-8.29 (m, 5H), 8.49 (s, 1H), 8.86 (bs, 1H), 9.22-9.28 (m, 2H), 9.66 (bs, 1H), 10.22 (bs, 1H); ^{13}C NMR (126 MHz, DMSO-d_6): 14.3, 21.2, 23.1, 25.3, 27.6, 28.3, 42.9, 44.0, 50.8, 51.8, 54.0, 59.3, 121.1, 122.7, 128.3, 129.9, 130.0, 130.6, 130.8, 131.5, 133.4, 138.3 141.0, 148.5, 165.9, 168.2; HRMS (ESI+) calcd for $C_{26}H_{37}N_6O_3S^+$ (M + H)$^+$ 513.2642, found 513.2652.

Ethyl *N*-{(*4S*)-4-amino-5-[(*S*)-[1-[(3'-carboxy-[1,1'-biphenyl]-3-yl)methyl]-piperidin-3-yl]amino]-5-oxopentyl}-imidothiocarbamate: 27.

Obtained after HPLC purification: white powder, 8 mg (6 %)[a].

HPLC (Gr3) t_R 1.12 min; LC-MS t_R: 0.80 min; *m/z* (ES+) 512.3 [M + H]$^+$; ^{1}H NMR (500 MHz, DMSO-d_6): 1.26 (t, *J* = 7.3 Hz, 3H), 1.41-1.56 (m, 3H), 1.66-1.71 (m, 3H), 1.83-1.96 (m, 2H), 2.77 (bs, 1H), 2.92 (bs, 1H), 3.18 (q, *J* = 7.1 Hz, 2H), 3.32 (m, 3H), 3.75 (bs, 1H), 4.01 (bs, 1H), 4.22 (bs, 1H), 4.41 (bs, 1H), 7.51 (bs, 1H), 7.59 (bs, 1H), 7.63 (t, *J* = 7.7 Hz, 1H), 7.84 (bs, 1H), 7.91 (m, 1H), 7.95-7.99 (m, 2H), 8.25 (bs, 4H), 8.85 (bs, 1H), 9.22 (bs, 1H), 9.28 (bs, 1H), 9.66 (bs, 1H), 10.14 (bs, 1H), 13.21 (s, 1H); ^{1}H NMR (500 MHz, DMSO-d_6, **373K**): 1.30 (t, *J* = 7.3 Hz, 3H), 1.41-1.49 (m, 1H), 1.57-1.63 (m, 2H), 1.69-1.79 (m, 3H), 1.87 (m, 2H), 3.05 (bs, 2H), 3.20 (q, *J* = 7.3 Hz, 2H), 3.34 (t, *J* = 7.2 Hz, 3H), 3.78 (t, *J* = 6.4 Hz, 1H), 3.99 & 4.10 (2bs, 3H), 7.47 (d, *J* = 7.1 Hz, 1H), 7.53 (t, *J* = 7.6 Hz, 1H), 7.61 (t, *J* = 7.7 Hz, 1H), 7.71 (d, *J* = 7.4 Hz, 1H), 7.77 (s, 1H), 7.91 (d, *J* = 7.7 Hz, 1H), 7.97 (d, *J* = 7.7 Hz, 1H), 8.22 (s, 1H), 8.38 (bs, 1H), 8.87 (bs, 2H);^{13}C NMR (125 MHz, DMSO-d_6): 14.2, 21.2, 23.1, 25.2, 27.6, 28.3, 42.9, 44.0, 50.8, 51.8, 53.9, 59.4, 127.4, 128.1, 128.8, 129.5, 129.7, 129.8, 130.4, 130.8, 131.2, 131.7, 139.7, 139.7, 165.9, 167.2, 168.2; HRMS (ESI+) calcd for $C_{27}H_{38}N_5O_3S^+$ (M + H)$^+$ 512.2690, found 512.2698.

6- Characterization data for 1,2,4-oxadiazole compounds.

The two quadruplets at 117.0 and 158.2 ppm on the ^{13}C NMR spectra are due to the TFA salt of these compounds.

Ethyl *N*-{(*4S*)-4-amino-4-[3-(2-aminoethyl)-1,2,4-oxadiazol-5-yl]-butyl}-imidothio-carbamate: 28.

Obtained after HPLC purification: white powder, 12 mg (33%)[b].

HPLC (Gr5) t_R 0.71 min; LC-MS t_R: 0.21 min; *m/z* (ES+) 287.3 $[M + H]^+$; ^{1}H NMR (500 MHz, DMSO-d_6) 1.26 (t, *J* = 7.3, Hz, 3H), 1.57-1.70 (m, 2H), 1.96 (q, *J* = 7.7 Hz, 2H), 3.09-3.12 (m, 2H), 3.16-3.20 (m, 4H), 3.33-3.36 (m, 2H), 4.86 (t, *J* = 6.9, Hz, 1H), 8.13 (bs, 3H), 9.05 & 9.24 (2bs, 5H); ^{13}C NMR (125 MHz, DMSO-d_6) δ 14.2, 23.2, 23.8, 25.2, 28.7, 36.2, 42.8, 46.6, 165.9, 167.2, 176.4; HRMS (ESI+) calcd for $C_{11}H_{23}N_6OS^+$ $(M + H)^+$ 287.1649, found 287.1646.

Ethyl *N*-{(*4S*)-4-amino-4-[3-(2-(*N*-benzyl-amino)ethyl)-1,2,4-oxadiazol-5-yl]-butyl}-imidothiocarbamate: 29.

Obtained after HPLC purification: white powder, 6 mg (14%)[b].

HPLC (Gr5) t_R 1.24 min; LC-MS t_R: 0.60 min; *m/z* (ES+) 377.1 $[M + H]^+$; ^{1}H NMR (500 MHz, DMSO-d_6) 1.25 (t, *J* = 7.3 Hz, 3H), 1.57-1.68 (m, 2H), 1.96 (q, *J* = 7.7 Hz, 2H), 3.16-3.23 (m, 4H), 3.31-3.36 (m, 4H), 4.26 (s, 2H), 4.87 (t, *J* = 6.9, Hz, 1H), 7.43-7.52 (m, 5H), 8.86-9.57 (m, 7H); ^{13}C NMR (125 MHz, DMSO-d_6) δ 14.2, 22.6, 23.2, 25.2, 28.6, 42.7, 43.1, 46.5, 50.0, 128.9, 129.2, 130.0, 131.9, 166.0, 167.1, 176.3; HRMS (ESI+) calcd for $C_{18}H_{29}N_6OS^+$ $(M + H)^+$ 377.2118, found 377.2117.

Ethyl *N*-{(*4S*)-4-amino-4-[3-(2-(*N*,*N*-dibenzyl-amino)ethyl)-1,2,4-oxadiazol-5-yl]-butyl}-imidothiocarbamate: 30.

Obtained after HPLC purification: white powder, 9 mg (6.5%)[a].

HPLC (Gr3) t_R 0.86 min; LC-MS t_R: 0.69 min; *m/z* (ES+) 467.2 $[M + H]^+$; ^{1}H NMR (500 MHz, DMSO-d_6) 1.27 (t, *J* = 7.3 Hz, 3H), 1.54-1.60 (m, 2H), 1.75-1.80 (m, 2H), 2.31-2.38 (m, 1H), 2.59-2.66 (m, 1H), 3.23 (q, *J* = 7.1 Hz, 2H), 3.39 (m, 2H), 4.01-4.14 (m, 3H), 4.80-4.87 (m, 2H), 4.94-4.98 (m, 2H), 7.45-7.49 (m, 4H), 7.53 (m, 6H), 8.47 (s, 3H), 9.34 (bs, 2H), 9.78 (m, 1H); ^{13}C NMR (125 MHz, DMSO-d_6) δ 14.3, 22.9, 25.3, 27.8, 34.9, 42.7, 52.5, 56.6, 68.6, 69.0, 128.2, 128.2, 128.8, 128.9, 130.4, 133.1, 133.2, 165.9, 167.2, 169.3; HRMS (ESI+) calcd for $C_{25}H_{35}N_6OS^+$ $(M + H)^+$ 467.2588, found 467.2585.

Ethyl *N*-{(*4S*)-4-amino-4-[3-(2-(*N*,*N*-di-(*o*-toluyl-methyl)-amino)ethyl)-1,2,4-oxadiazol-5-yl]-butyl}-imidothiocarbamate: 31.

Obtained after HPLC purification: white powder, 11 mg (8%)[a].

HPLC (Gr3) t_R 1.26 min; LC-MS t_R: 0.85 min; *m/z* (ES+) 495.2 $[M + H]^+$; ^{1}H NMR (500 MHz, DMSO-d_6) 1.28 (t, *J* = 7.3 Hz, 3H), 1.46-1.54 (m, 2H), 1.63-1.71 (m, 2H), 2.37 & 2.40 (2s, 6H), 2.70-2.77 (m, 2H), 3.21 (q, *J* = 7.3 Hz, 2H), 3.96 (bs, 1H), 4.04-4.10 (m, 1H), 4.22 (m, 1H), 4.93-5.03 (m, 3H), 7.26-7.31 (m, 4H), 7.38-7.42 (m, 2H), 7.55 (d, *J* = 7.6 Hz, 1H), 7.61 (d, *J* = 7.6 Hz, 1H), 8.34 (bs, 2H), 9.28 & 9.68 (2bs, 3H); ^{13}C NMR (125 MHz, DMSO-d_6) 14.3,

19.8, 19.9, 23.0, 25.3, 34.6, 42.8, 52.7, 57.0, 66.0, 66.4, 126.1, 126.2, 126.8, 126.9, 130.5, 130.6, 131.2, 131.3, 134.1, 140.4, 140.5, 166.0, 166.3, 169.4; HRMS (ESI+) calcd for $C_{27}H_{39}N_6OS^+$ $(M + H)^+$ 495.2901, found 495.2905.

Ethyl *N*-{(*4S*)-4-amino-4-[3-(2-(*N*-(2,6-dimethyl-phenylmethyl)amino)ethyl)-1,2,4-oxadiazol-5-yl]-butyl}-imidothiocarbamate: 32.

Obtained after HPLC purification: white powder, 4 mg (3%)[a].

HPLC (Gr3) t_R 1.13 min; LC-MS t_R: 0.74 min; *m/z* (ES+) 405.2 $[M + H]^+$; ^1H NMR (300 MHz, DMSO-d_6) 1.26 (t, J = 7.3 Hz, 3H), 1.58-1.69 (m, 2H), 1.97 (q, J = 7.7 Hz, 2H), 2.28 (s, 3H), 2.34 (s, 3H), 3.18 (q, J = 7.3 Hz, 2H), 3.24 (m, 2H), 3.35 (t, J = 7.1 Hz, 2H), 3.40 (m, 2H), 4.21 (bs, 2H), 4.89 (t, J = 6.9 Hz, 1H), 7.07-7.10 (m, 2H), 7.33 (d, J = 7.7 Hz, 1H), 9.05 (bs, 2H), 9.18 (bs, 2H), 9.25 (bs, 1H), 9.62 (m, 1H); ^{13}C NMR (75 MHz, DMSO-d_6) 14.2, 18.8, 20.7, 22.5, 23.1, 25.2, 28.5, 42.7, 43.5, 46.5, 47.3, 130.3, 131.3, 137.2, 138.6, 166.0, 167.1, 176.1; HRMS (ESI+) calcd for $C_{20}H_{33}N_6OS^+$ $(M + H)^+$ 405.2431, found 405.2437.

Ethyl *N*-{(*4S*)-4-amino-4-[3-(2-(*N,N*-di-(2,6-dimethyl-phenylmethyl)amino)ethyl)-1,2,4-oxadiazol-5-yl]-butyl}-imidothiocarbamate: 33.

Obtained after HPLC purification: white powder, 15 mg (10%)[a].

HPLC (Gr3) t_R 1.46 min; LC-MS t_R: 1.13 min; *m/z* (ES+) 523.3 $[M + H]^+$; ^1H NMR (300 MHz, DMSO-d_6) 1.25 (t, J = 7.3 Hz, 3H), 1.51-1.65 (m, 2H), 1.86-1.96 (m, 2H), 2.23 (s, 12H), 2.72-2.78 (m, 2H), 2.89-2.95 (m, 2H), 3.18 (q, J = 7.3 Hz, 2H), 3.32 (m, 2H), 3.57 (s, 4H), 4.79 (m, 1H), 6.95-7.07 (m, 6H), 8.87-9.55 (4bs, 5H); ^{13}C NMR (75 MHz, DMSO-d_6) 14.2, 19.7, 23.1, 23.9, 25.2, 28.5, 42.7, 46.4, 50.4, 51.5, 127.0, 128.0, 134.6, 137.8, 165.9, 169.3, 175.3; HRMS (ESI+) calcd for $C_{29}H_{43}N_6OS^+$ $(M + H)^+$ 523.3214, found 523.3217.

Ethyl *N*-{(*4S*)-4-amino-4-[3-(2-(*N,N*-di-(naphth-1-yl-methyl)amino)ethyl)-1,2,4-oxadiazol-5-yl]-butyl}-imidothiocarbamate: 34.

Obtained after HPLC purification: white powder, 12 mg (7.5%)[a].

HPLC (Gr3) t_R 1.47 min; LC-MS t_R: 1.02 min; *m/z* (ES+) 567.2 $[M + H]^+$; ^1H NMR (500 MHz, DMSO-d_6) 1.29 (t, J = 7.3 Hz, 3H), 1.31-1.48 (m, 4H), 1.90-1.97 (m, 1H), 2.59-2.66 (m, 1H), 3.23 (q, J = 7.3 Hz, 2H), 3.32 (m, 2H), 3.82 (bs, 1H), 4.01-4.07 (m, 1H), 4.27-4.33 (m, 1H), 5.55-5.64 (m, 2H), 5.70-5.76 (m, 2H), 7.58-7.64 (m, 4H), 7.68-7.73 (m, 2H), 7.82-7.87 (m, 2H), 7.99-8.05 (m, 2H), 8.12 (t, J = 8.4 Hz, 2H), 8.24 (bs, 3H), 8.48 (d, J = 8.7 Hz, 1H), 8.56 (d, J = 8.6 Hz, 1H), 9.29 & 9.33 (2bs, 2H), 9.66 (bs, 1H); ^{13}C NMR (125 MHz, DMSO-d_6) 14.3, 22.8, 25.3, 27.8, 34.8, 42.9, 52.4, 56.7, 65.3, 65.8, 124.3, 124.4, 124.5, 124.5, 125.3, 126.3, 126.4,

127.5, 127.5, 128.8, 129.0, 131.5, 131.6, 132.7, 133.0, 133.5, 133.6, 134.0, 134.0, 166.0, 166.6, 169.1; HRMS (ESI+) calcd for $C_{33}H_{39}N_6OS^+$ $(M + H)^+$ 567.2901, found 567.2906.

Ethyl *N*-{(*4S*)-4-amino-4-[3-(2-(*N,N*-di-(naphth-2-yl-methyl)amino)ethyl)-1,2,4-oxadiazol-5-yl]-butyl}-imidothiocarbamate: 35.

Obtained after HPLC purification: white powder, 10 mg (6.5%)[a].

HPLC (Gr3) t_R 1.47 min; LC-MS t_R: 1.05 min; *m/z* (ES+) 567.2 $[M + H]^+$; ^1H NMR (300 MHz, DMSO-*d6*) 1.28 (t, *J* = 7.3 Hz, 3H), 1.51-1.58 (m, 2H), 1.67-1.78 (m, 2H), 2.38-2.47 (m, 1H), 2.69-2.82 (m, 1H), 3.22 (q, *J* = 7.3 Hz, 2H), 4.02 (s, 1H), 4.14-4.31 (m, 2H), 5.03-5.19 (m, 4H), 7.59-7.66 (m, 6H), 7.96-8.01 (m, 6H), 8.18 (s, 2H), 8.38 (bs, 2H), 9.30-9.62 (2bs, 2H); ^{13}C NMR (75 MHz, DMSO-*d6*) 14.1, 22.8, 25.3, 27.8, 34.8, 42.8, 52.6, 56.6, 68.8, 69.1, 125.6, 125.7, 126.8, 127.6, 128.1, 128.2, 128.3, 129.4, 132.3, 132.4, 133.2, 133.2, 133.4, 133.5, 166.0, 167.2, 169.3; HRMS (ESI+) calcd for $C_{33}H_{39}N_6OS^+$ $(M + H)^+$ 567.2901, found 567.2905.

Ethyl *N*-{(*4S*)-4-amino-4-[3-(2-(*N,N*-di-(2-hydroxy-phenylmethyl)amino)ethyl)-1,2,4-oxadiazol-5-yl]-butyl}-imidothiocarbamate: 36.

Obtained after HPLC purification: white powder, 10 mg (7%)[a].

HPLC (Gr3) t_R 1.16 min; LC-MS t_R: 0.83 min; *m/z* (ES+) 499.2 $[M + H]^+$; ^1H NMR (300 MHz, DMSO-*d6*) 1.25 (t, *J* = 7.3 Hz, 3H), 1.54-1.67 (m, 2H), 1.95 (q, *J* = 7.8 Hz, 2H), 3.18 (q, *J* = 7.3 Hz, 2H), 3.30-3.35 (m, 5H), 4.21 (bs, 4H), 4.85 (t, *J* = 6.8 Hz, 1H), 6.84 (t, *J* = 7.4 Hz, 2H), 6.90 (d, *J* = 8.1 Hz, 4H), 7.23 (t, *J* = 7.6 Hz, 2H), 7.32 (d, *J* = 7.6 Hz, 2H), 9.26 (bs, 3H), 9.57 (bs, 2H); ^{13}C NMR (75 MHz, DMSO-*d6*) 14.1, 23.1, 25.2, 27.8, 28.5, 42.7, 46.5, 52.6, 63.2, 63.6, 114.2, 115.4, 115.5, 119.1, 119.2, 132.1, 134.9, 135.0, 155.9, 156.6, 157.2, 157.2, 166.0, 166.9, 169.2; HRMS (ESI+) calcd for $C_{25}H_{35}N_6O_3S^+$ $(M + H)^+$ 499.2486, found 499.2490.

Ethyl *N*-{(*4S*)-4-amino-4-[3-(2-(*N,N*-di-(4-nitro-phenylmethyl)amino)ethyl)-1,2,4-oxadiazol-5-yl]-butyl}-imidothiocarbamate: 37.

Obtained after HPLC purification: yellowish powder, 16 mg (10%)[a].

HPLC (Gr3) t_R 1.23 min; LC-MS t_R: 0.83 min; *m/z* (ES+) 557.2 $[M + H]^+$; ^1H NMR (300 MHz, DMSO-*d6*) 1.27 (t, *J* = 7.3 Hz, 3H), 1.51-1.61 (m, 2H), 1.73-1.82 (m, 2H), 2.65-2.77 (m, 1H), 2.85-2.96 (m, 2H), 3.20 (q, *J* = 7.3 Hz, 2H), 3.35 (m, 2H), 4.04-4.22 (m, 3H), 4.96-5.16 (m, 4H), 7.81-7-85 (m, 4H), 8.31 (dd, *J* = 1.5, 8.8 Hz, 4H), 8.46 (bs, 3H), 9.27 (m, 2H), 9.66 (bs, 1H); ^{13}C NMR (75 MHz, DMSO-*d6*) 14.2, 22.9, 25.3, 27.8, 34.8, 42.9, 52.7, 57.7, 67.3, 67.6, 123.6, 123.6, , 134.7, 135.1, 135.2, 148.6, 148.6, 166.0, 167.7, 169.4; HRMS (ESI+) calcd for $C_{25}H_{33}N_8O_5S^+$ $(M + H)^+$ 557.2289, found 557.2293.

Ethyl *N*-{(*4S*)-4-amino-4-[3-(2-(*N,N*-di-(3-nitro-phenylmethyl)amino)ethyl)-1,2,4-oxadiazol-5-yl]-butyl}-imidothiocarbamate: 38.

Obtained after HPLC purification: yellowish powder, 6.8 mg (4%)[a].

HPLC (Gr3) t_R 1.20 min; LC-MS t_R: 0.82 min; *m/z* (ES+) 557.2 $[M + H]^+$; ^1H NMR (300 MHz, DMSO-d_6) 1.28 (t, *J* = 7.3 Hz, 3H), 1.49-1.59 (m, 2H), 1.70-1.78 (m, 2H), 2.68-2.80 (m, 1H), 2.86-2.98 (m, 1H), 3.21 (q, *J* = 7.3 Hz, 2H), 3.35 (m, 2H), 4.02 (bs, 1H), 4.15 (m, 2H), 5.00-5.16 (m, 4H), 7.77 (td, *J* = 2.5, 8.0 Hz, 2H), 7.96 (d, *J* = 7.7 Hz, 2H), 8.36-8.49 (m, 6H), 9.27 (m, 2H), 9.65 (bs, 1H); ^{13}C NMR (75 MHz, DMSO-d_6) 14.2, 22.9, 25.3, 27.8, 34.8, 42.9, 52.7, 57.3, 67.2, 67.5, 125.2, 128.0, 128.0, 129.9, 130.2, 130.3, 139,6, 147.8, 147.9, 166.0, 167.8, 169.3; HRMS (ESI+) calcd for $C_{25}H_{33}N_8O_5S^+$ $(M + H)^+$ 557.2289, found 557.2291.

Ethyl *N*-{(*4S*)-4-amino-4-[3-(2-(*N*-(2-nitro-phenylmethyl)amino)ethyl)-1,2,4-oxadiazol-5-yl]-butyl}-imidothiocarbamate: 39.

Obtained after HPLC purification: yellowish powder, 10 mg (7.5%)[a].

HPLC (Gr3) t_R 0.83 min; LC-MS t_R: 0.56 min; *m/z* (ES+) 422.2 $[M + H]^+$; HRMS (ESI+) calcd for $C_{18}H_{28}N_7O_3S^+$ $(M + H)^+$ 422.1969, found 422.1979.

Ethyl *N*-{(*4S*)-4-amino-4-[3-(2-(*N,N*-di-(2-nitro-phenylmethyl)amino)ethyl)-1,2,4-oxadiazol-5-yl]-butyl}-imidothiocarbamate: 40.

Obtained after HPLC purification: yellowish powder, 6.7 mg (4%)[a].

HPLC (Gr3) t_R 1.10 min; LC-MS t_R: 1.18 min; *m/z* (ES+) 557.4 $[M + H]^+$; ^1H NMR (500 MHz, DMSO-d_6) 1.27 (t, *J* = 7.3 Hz, 3H), 1.52-1.64 (m, 2H), 1.78-1.86 (m, 2H), 2.72-2.79 (m, 1H), 3.11-3.16 (m, 1H), 3.20 (t, *J* = 7.3 Hz, 2H), 3.38 (m, 2H), 4.05 (bs, 1H), 4.08-4.14 (m, 2H), 5.23-5.44 (m, 4H), 7.79-7.91 (m, 5H), 8.00 (m, 1H), 8.03-8.07 (m, 2H), 8.38 (bs, 3H), 9.23-9.28 (2bs, 2H), 9.68 (bs, 1H); ^{13}C NMR (125 MHz, DMSO-d_6) 14.2, 22.5, 23.2, 25.3, 28.5, 42.7, 46.5, 51.1, 54.3, 124.4, 128.7, 131.0, 133.0, 133.1, 149.3, 166.9, 168.9, 175.6; HRMS (ESI+) calcd for $C_{25}H_{33}N_8O_5S^+$ $(M + H)^+$ 557.2289, found 557.2292.

Ethyl *N*-{(*4S*)-4-amino-4-[3-(2-(*N*-(5-hydroxy-2-nitro-phenylmethyl)-*N*-benzyl-amino)ethyl)-1,2,4-oxadiazol-5-yl]-butyl}-imidothiocarbamate: 41.

Obtained after HPLC purification: yellowish powder, 15 mg (11%)[a].

HPLC (Gr3) t_R 0.93 min; LC-MS t_R: 0.68 min; *m/z* (ES+) 438.2 $[M + H]^+$; ^1H NMR (300 MHz, DMSO-d_6) 1.26 (t, *J* = 7.3 Hz, 3H), 1.58-1.69 (m, 2H), 1.98 (q, *J* = 7.6 Hz, 2H), 3.18 (q, *J* = 7.3 Hz, 2H), 3.24-3.29 (m, 2H), 3.35 (t, *J* = 6.9 Hz, 2H), 3.47 (t, *J* = 7.9 Hz, 2H), 4.52 (s, 2H), 4.90 (t, *J* = 6.9 Hz, 1H), 7.03-7.09 (m, 2H), 8.21 (d, *J* = 8.9 Hz, 1H), 9.27 (bs, 6H); ^{13}C NMR (75 MHz, DMSO-d_6) 14.1, 22.6, 23.1, 25.2, 28.6, 42.7, 44.0, 46.5, 48.4, 116.7, 119.9, 128.8,

130.4, 139.6, 163.2, 166.0, 167.1, 176.1; HRMS (ESI+) calcd for $C_{18}H_{28}N_7O_4S^+$ $(M + H)^+$ 438.1918, found 438.1923.

Ethyl *N*-{(*4S*)-4-amino-4-[3-(2-(*N,N*-di-(5-hydroxy-2-nitro-phenylmethyl)amino)ethyl)-1,2,4-oxadiazol-5-yl]-butyl}-imidothiocarbamate: 42.

Obtained after HPLC purification: yellowish powder, 20 mg (12%)[a].

HPLC (Gr3) t_R 1.17 min; LC-MS t_R: 0.74 min; *m/z* (ES+) 589.2 $[M + H]^+$; ^1H NMR (400 MHz, DMSO-d_6) 1.24 (t, J = 7.3 Hz, 3H), 1.52-1.65 (m, 2H), 1.92-1.97 (m, 2H), 2.81-2.87 (m, 2H), 3.02 (m, 2H), 3.17 (q, J = 7.3 Hz, 2H), 3.33 (t, J = 7.0 Hz, 2H), 3.99 (s, 4H), 4.84 (t, J = 6.9 Hz, 1H), 6.81 (dd, J = 2.6, 8.9 Hz, 2H), 7.08 (d, J = 2.7 Hz, 2H), 7.91 (d, J = 8.9 Hz, 2H), 8.99 (bs, 2H), 9.16-9.25 (2bs, 2H), 9.57 (bs, 1H), 11.01 (bs, 2H); ^{13}C NMR (101 MHz, DMSO-d_6) δ 14.1, 22.9, 23.1, 25.2, 28.5, 42.7, 46.5, 51.4, 54.7, 55.0, 114.3, 116.5, 127.9, 137.7, 140.4, 162.3, 165.9, 168.8, 175.6; HRMS (ESI+) calcd for $C_{25}H_{33}N_8O_7S^+$ $(M + H)^+$ 589.2187, found 589.2193.

Global yield obtained from 0.2 mmol resin[a] or 0.07 mmol lantern[b] and based on an apparent molecular weight of M + one or two TFA.

7- Cell toxicity.

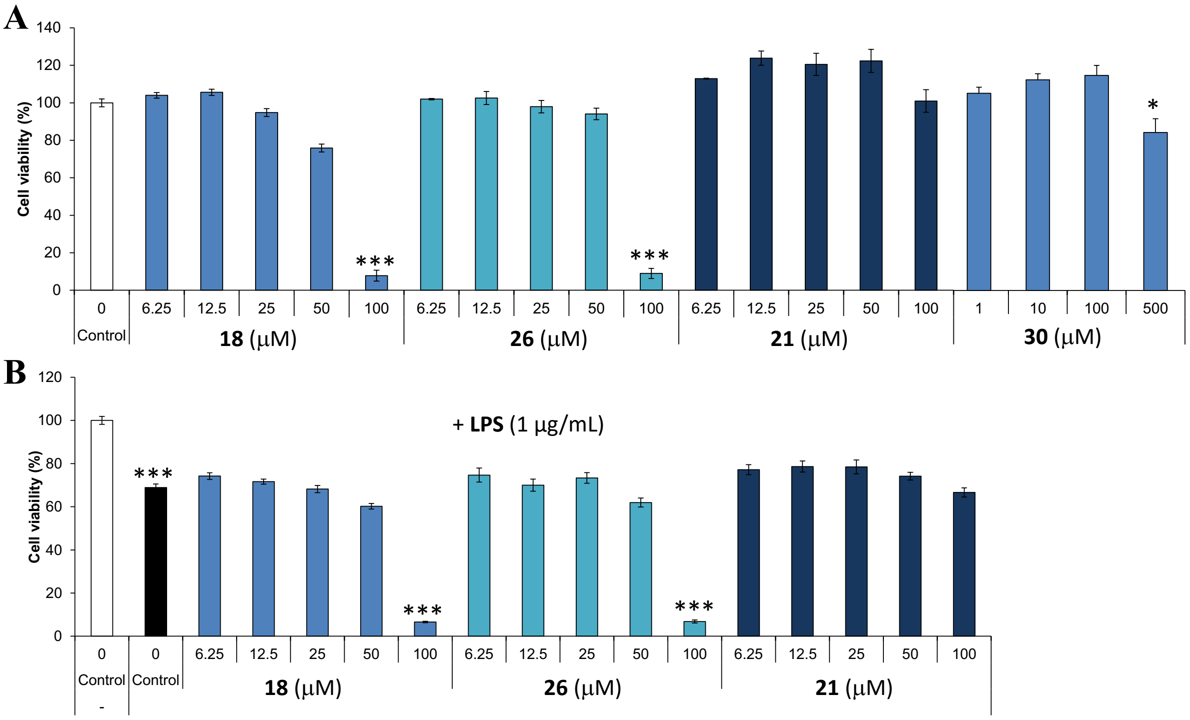

Figure S1. Effects of compounds **18**, **21**, **26** and **30** on cell viability: MTT assay on RAW 264.7 in the absence (A) or the presence (B) (for **18**, **21**, **26**) of LPS (1 µg/mL). *, P<0.05; ***, P<0.001.

8- Molecular modelling.

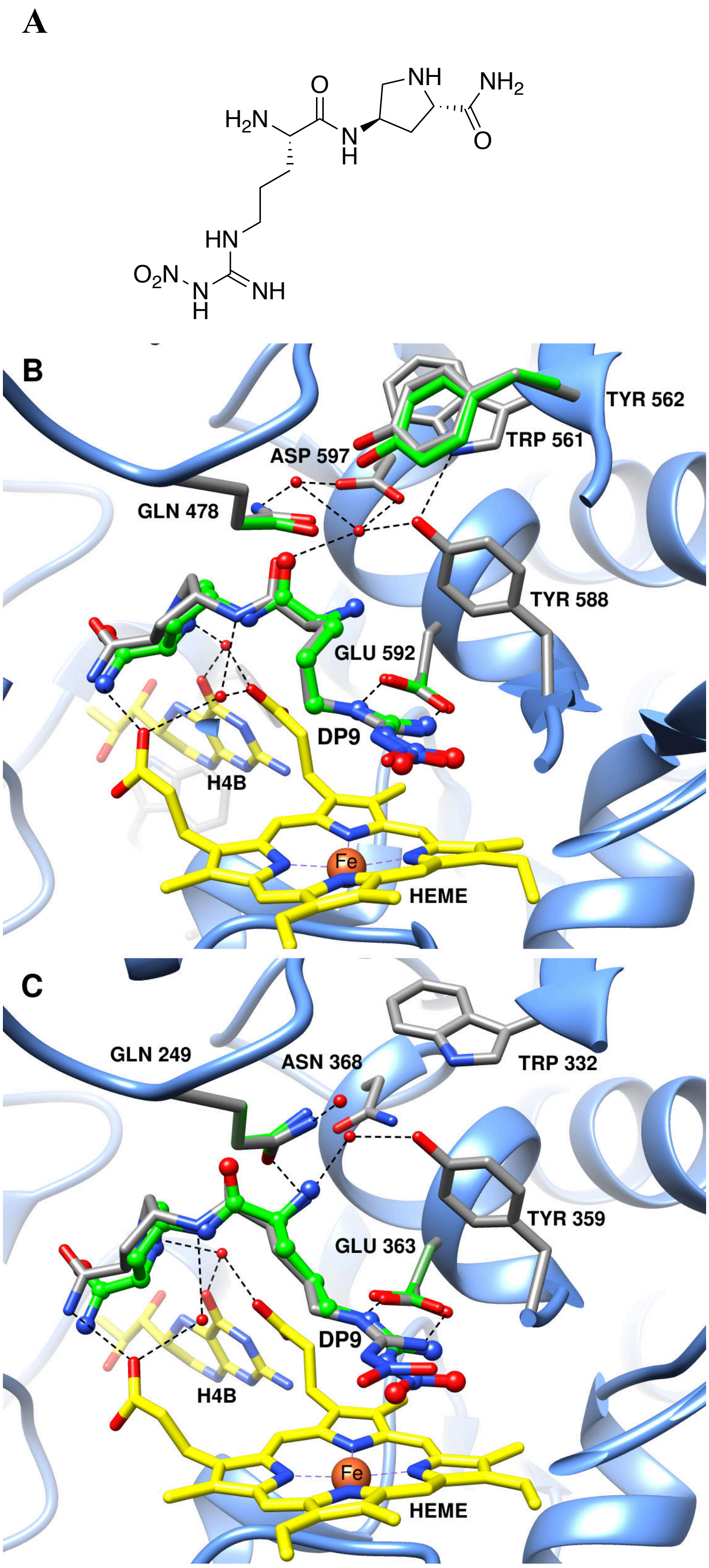

Figure S2. (A) Structure of DP9 https://www.rcsb.org/ligand/DP9); (B) Best docking pose of DP9 in nNOS compared to the crystallographic pose (pdb code 1P6J); (C) Best docking pose of DP9 in eNOS compared to the crystallographic pose (pdb code 1P6N). Blue: protein ribbon; green stick: protein side chain adaptation during docking; green ball and stick: docking results for DP9; yellow: cofactors of NOS, heme and tetrahydrobiopterin (H4B).

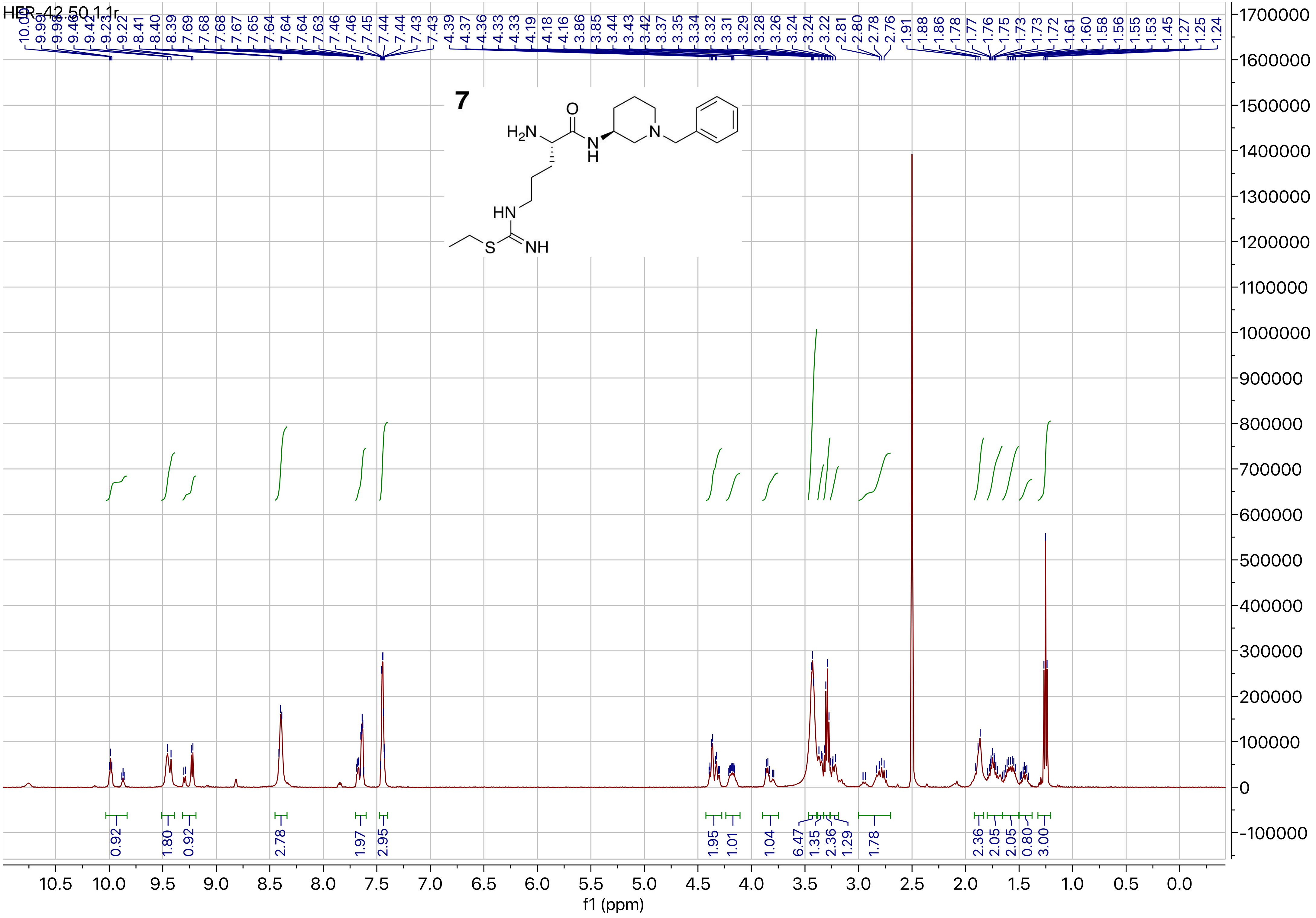
HER-42.50.1.1r
7
H2N
O
N
H
N
HN
S
NH
9.99 9.98 9.46 9.42 9.23 9.22 8.41 8.40 8.39 7.69 7.68 7.68 7.67 7.65 7.64 7.64 7.64 7.63 7.46 7.46 7.45 7.44 7.44 7.43 7.43 4.39 4.37 4.36 4.33 4.33 4.19 4.18 4.16 3.86 3.85 3.44 3.43 3.42 3.37 3.35 3.34 3.32 3.31 3.29 3.28 3.26 3.24 3.24 3.22 2.81 2.80 2.78 2.76 1.91 1.88 1.86 1.78 1.77 1.76 1.75 1.73 1.72 1.61 1.60 1.58 1.56 1.55 1.53 1.45 1.27 1.25 1.24
0.92 1.80 0.92 2.78 1.97 2.95 1.95 1.01 1.04 6.47 1.35 2.36 1.29 1.78 2.36 2.05 2.05 0.80 3.00
10.5 10.0 9.5 9.0 8.5 8.0 7.5 7.0 6.5 6.0 5.5 5.0 4.5 4.0 3.5 3.0 2.5 2.0 1.5 1.0 0.5 0.0
f1 (ppm)
1700000 1600000 1500000 1400000 1300000 1200000 1100000 1000000 900000 800000 700000 600000 500000 400000 300000 200000 100000 0 -100000

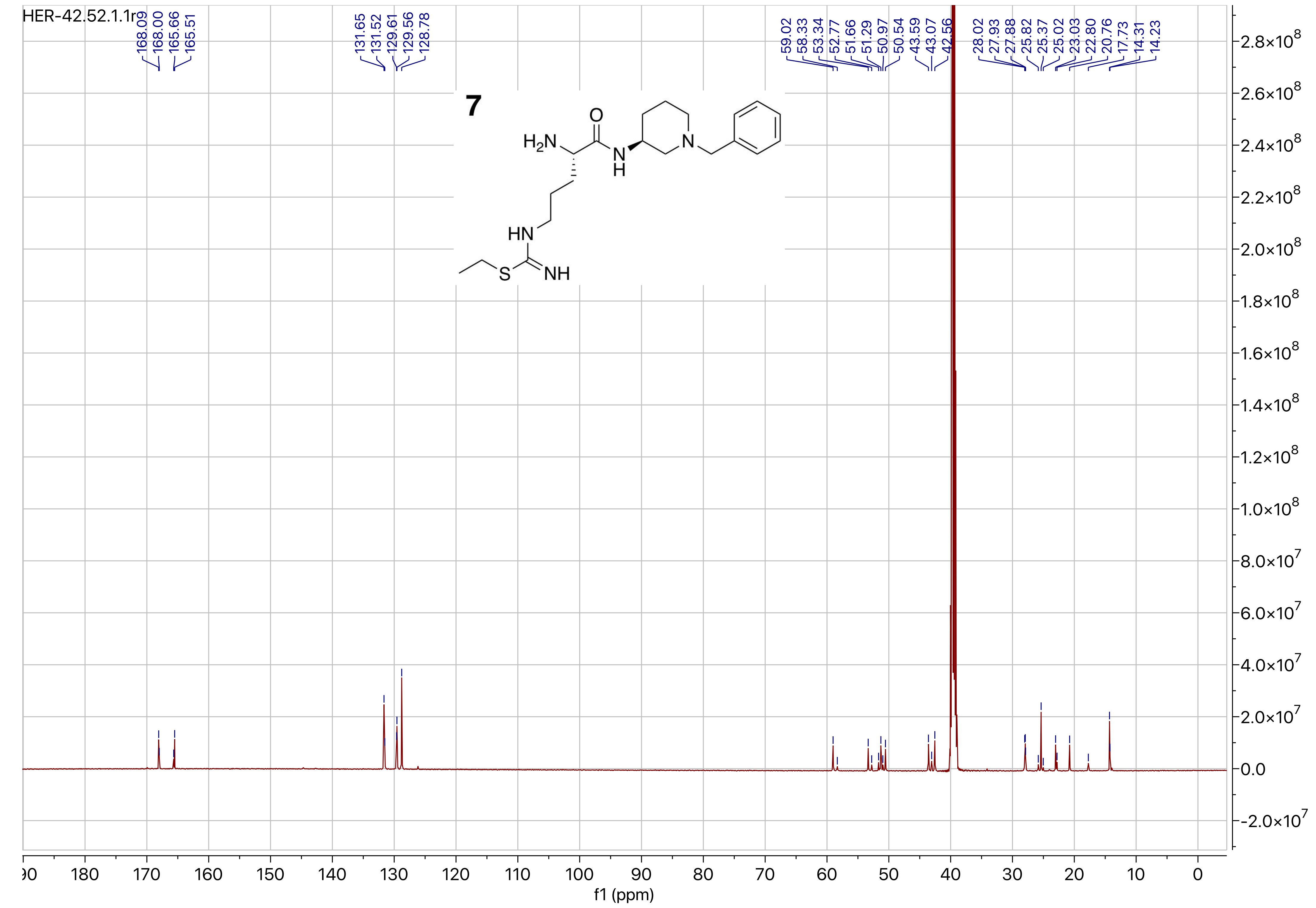

HER-42.52.1.1r
168.09
168.00
165.66
165.51
131.65
131.52
129.61
129.56
128.78
59.02
58.33
53.34
52.77
51.66
51.29
50.97
50.54
43.59
43.07
42.56
28.02
27.93
27.88
25.82
25.37
25.02
23.03
22.80
20.76
17.73
14.31
14.23
7
O
H2N
N
H
N
HN
S
NH
f1 (ppm)

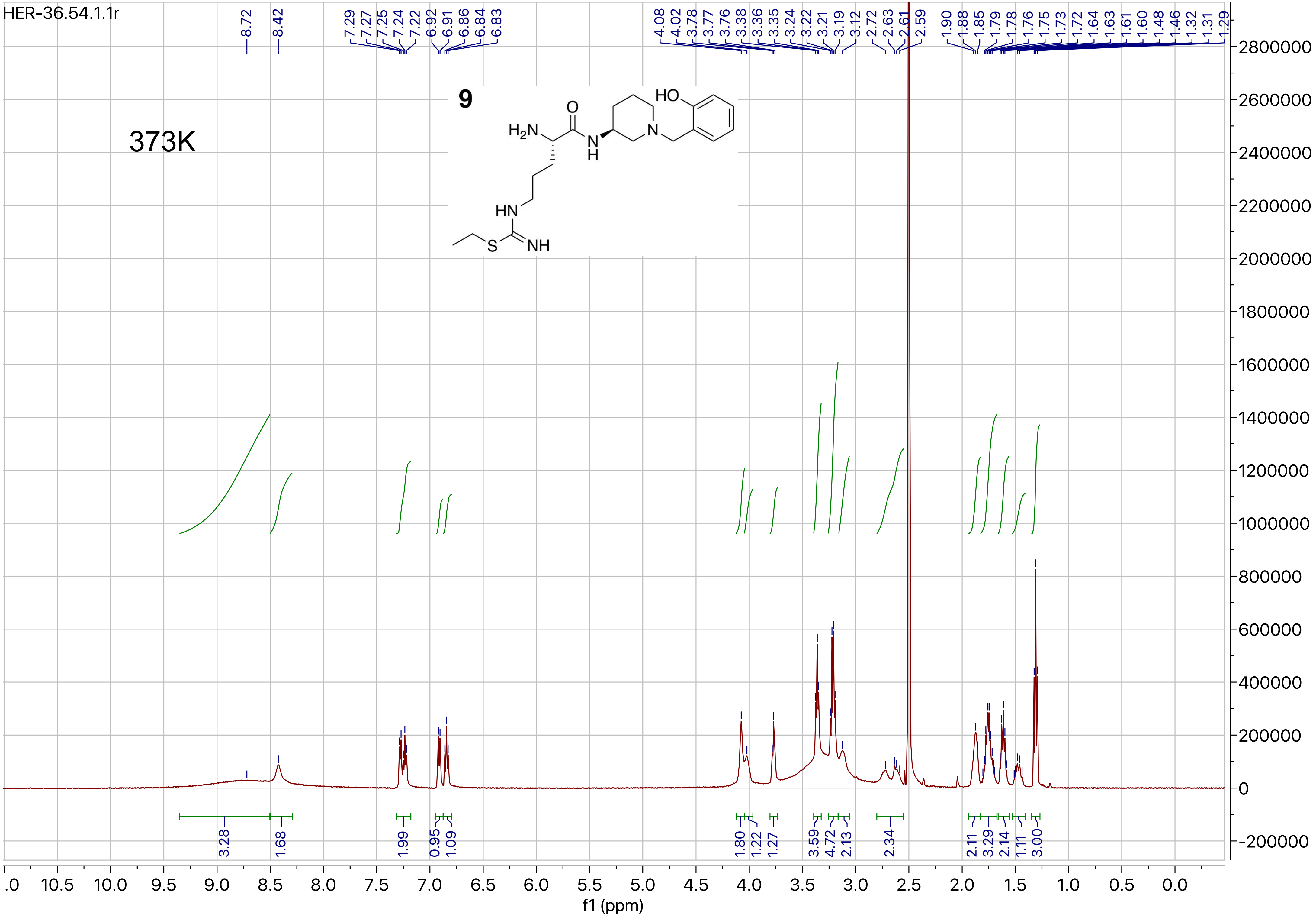

HER-36.54.1.1r
373K
9
8.72
8.42
7.29
7.27
7.25
7.24
7.22
6.92
6.91
6.86
6.84
6.83
4.08
4.02
3.78
3.77
3.76
3.38
3.36
3.35
3.24
3.22
3.21
3.19
3.12
2.72
2.63
2.61
2.59
1.90
1.88
1.85
1.79
1.78
1.76
1.75
1.73
1.72
1.64
1.63
1.61
1.60
1.48
1.46
1.32
1.31
1.29
3.28
1.68
1.99
0.95
1.09
1.80
1.22
1.27
3.59
4.72
2.13
2.34
2.11
3.29
2.14
1.11
3.00
f1 (ppm)

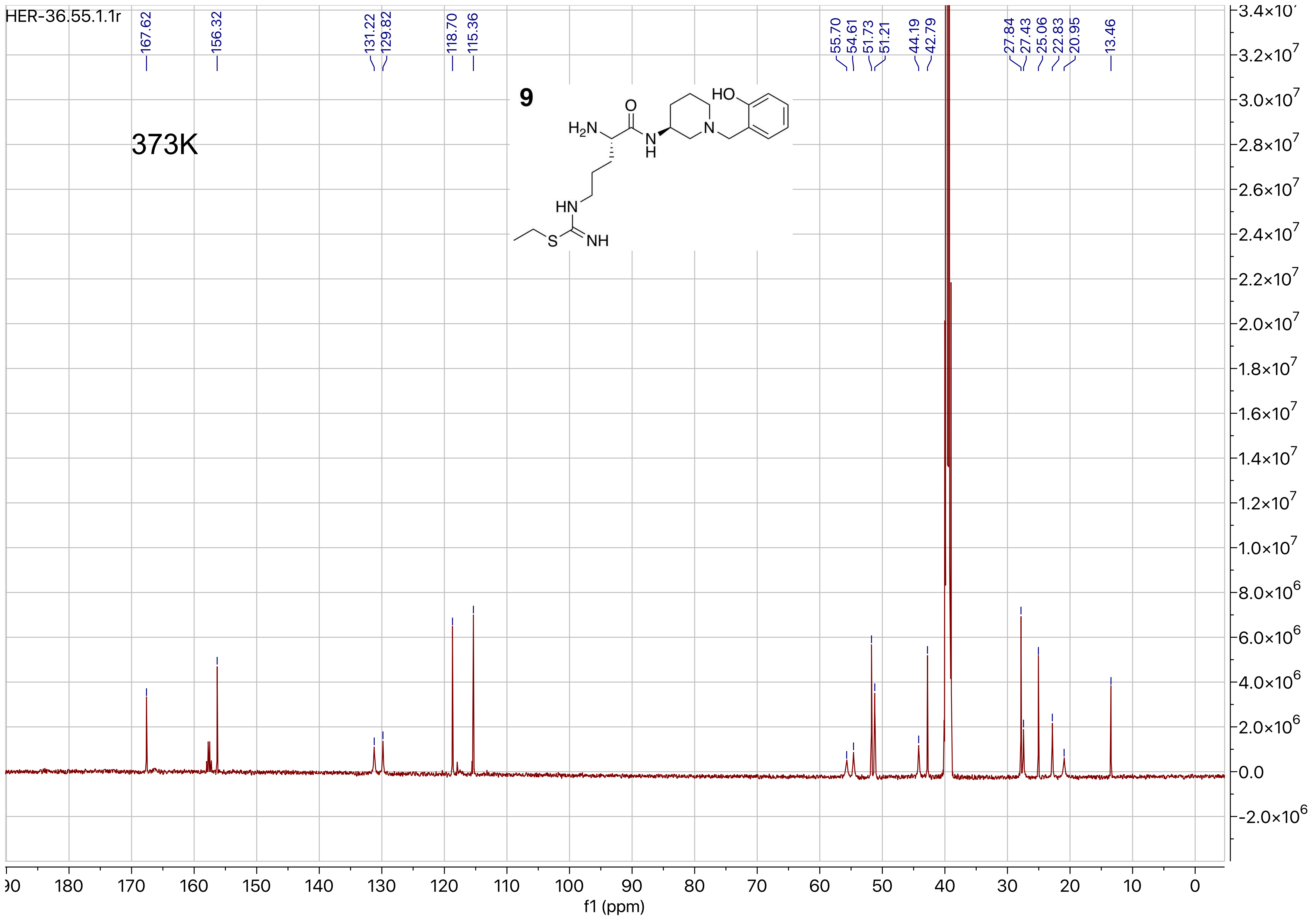
HER-36.55.1.1r
167.62
156.32
131.22
129.82
118.70
115.36
55.70
54.61
51.73
51.21
44.19
42.79
27.84
27.43
25.06
22.83
20.95
13.46
373K
9
HO
O
H2N
N
H
N
HN
S
NH
f1 (ppm)

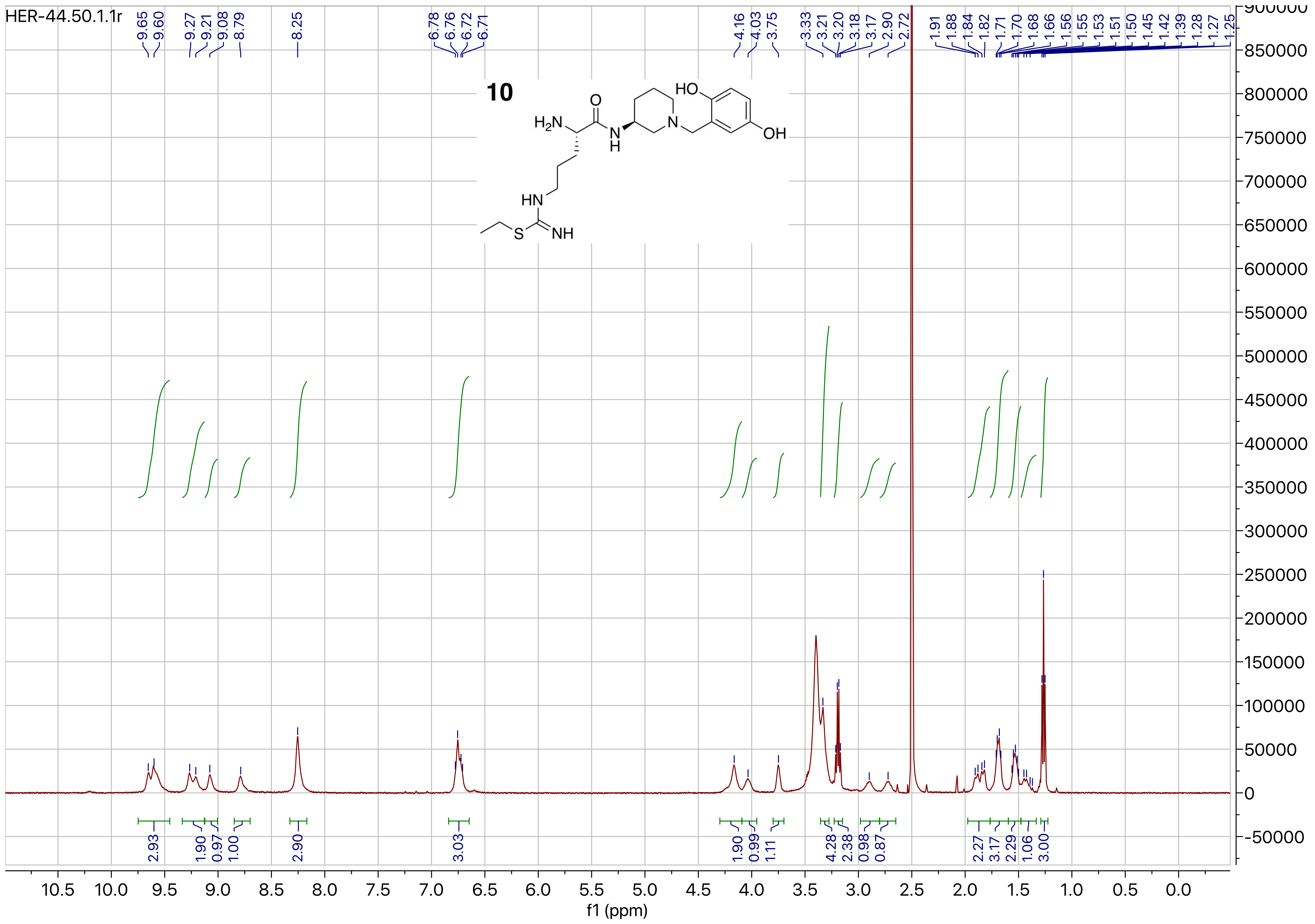

HER-44.50.1.1r
10
9.65 9.60 9.27 9.21 9.08 8.79 8.25 6.78 6.76 6.72 6.71 4.16 4.03 3.75 3.33 3.21 3.20 3.18 3.17 2.90 2.72 1.91 1.88 1.84 1.82 1.71 1.70 1.68 1.66 1.56 1.55 1.53 1.51 1.50 1.45 1.42 1.39 1.28 1.27 1.25
2.93 1.90 0.97 1.00 2.90 3.03 1.90 0.99 1.11 4.28 2.38 0.98 0.87 2.27 3.17 2.29 1.06 3.00
f1 (ppm)

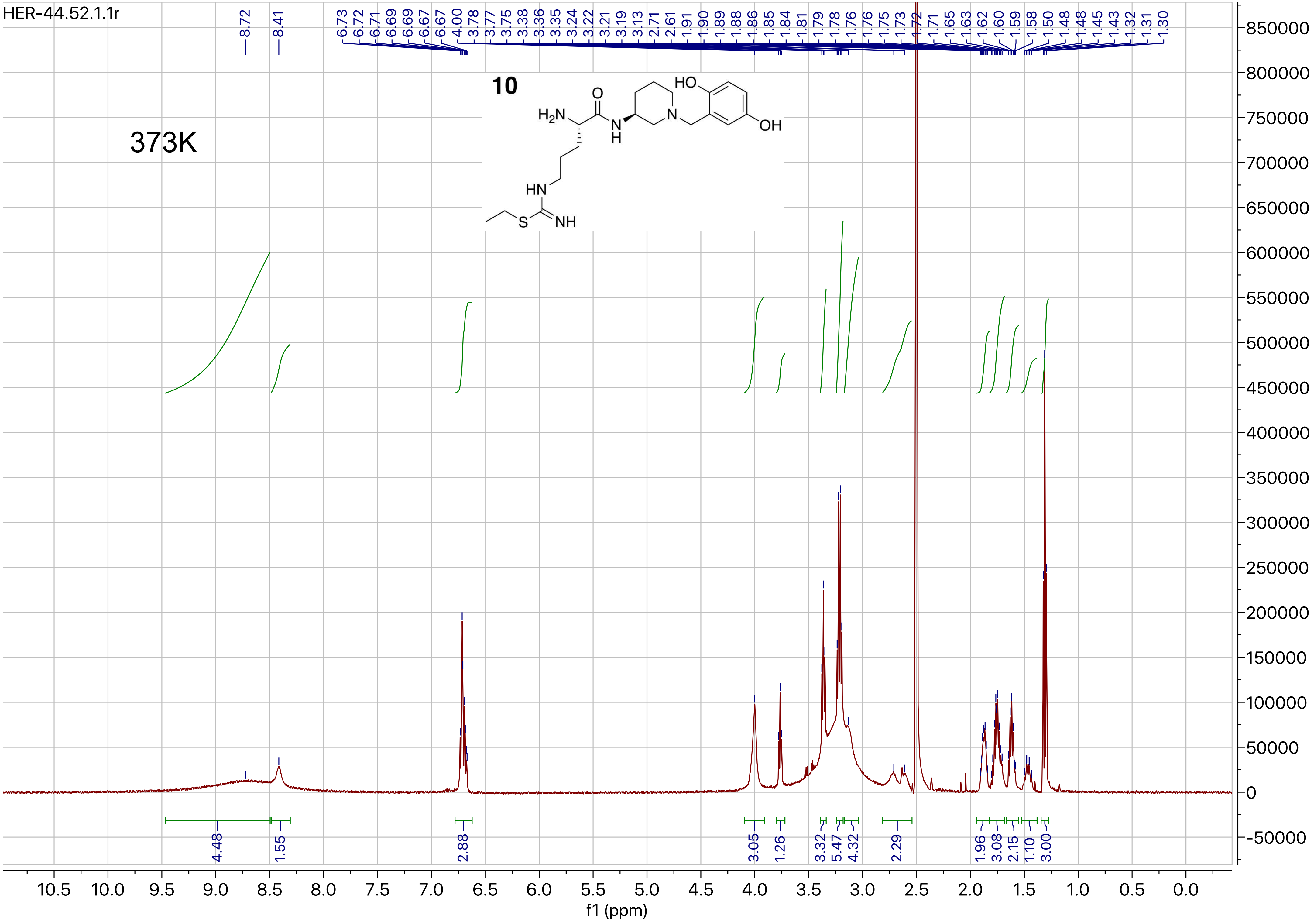
HER-44.52.1.1r
373K
10
H2N
O
N
H
N
HO
OH
HN
S
NH
8.72
8.41
6.73
6.72
6.71
6.69
6.69
6.67
6.67
4.00
3.78
3.77
3.75
3.38
3.36
3.35
3.24
3.22
3.21
3.19
3.13
2.71
2.61
1.91
1.90
1.89
1.88
1.86
1.85
1.84
1.81
1.79
1.78
1.76
1.76
1.75
1.73
1.72
1.71
1.65
1.63
1.62
1.60
1.59
1.58
1.50
1.48
1.48
1.45
1.43
1.32
1.31
1.30
4.48
1.55
2.88
3.05
1.26
3.32
5.47
4.32
2.29
1.96
3.08
2.15
1.10
3.00
f1 (ppm)

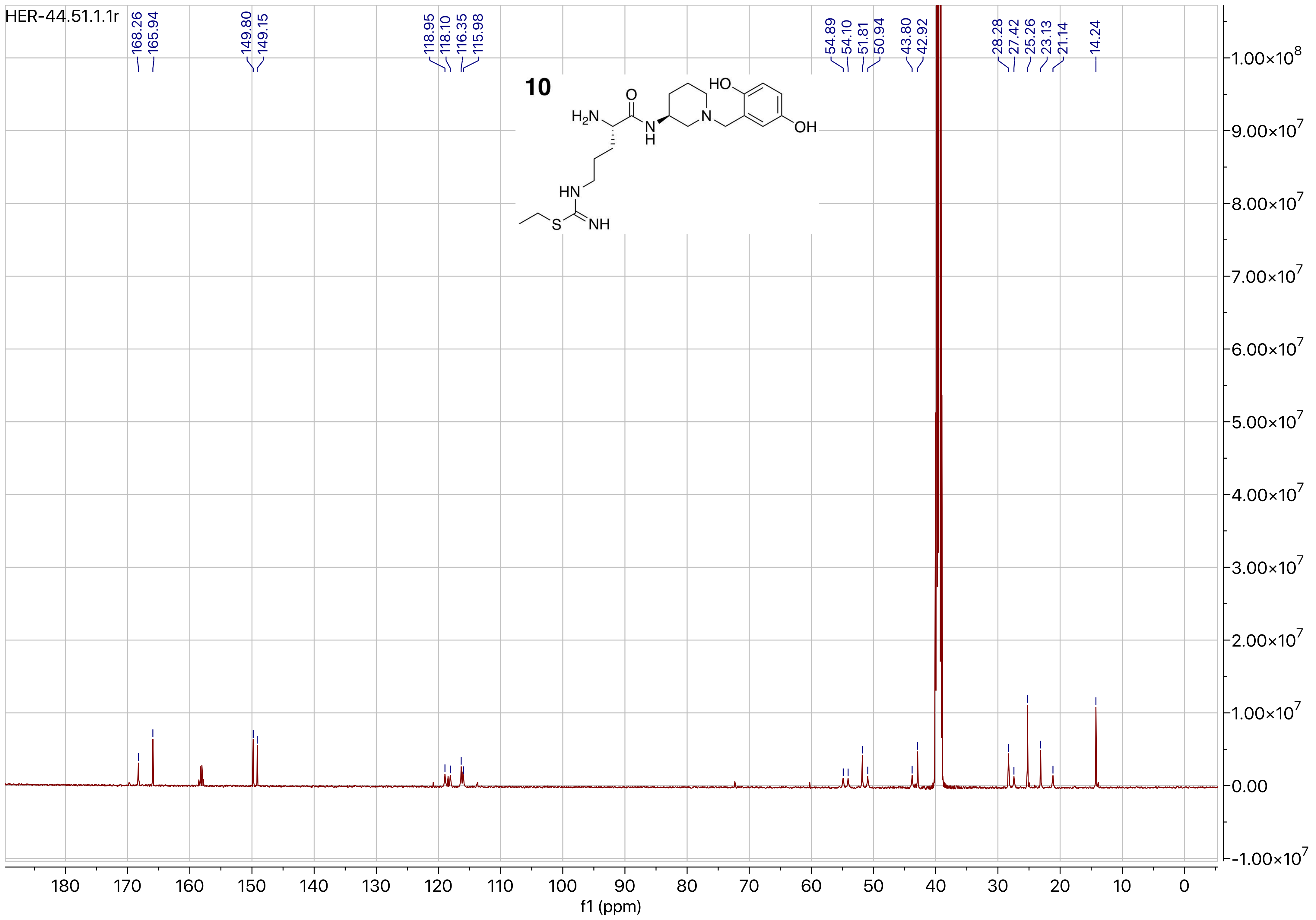
HER-44.51.1.1r
168.26
165.94
149.80
149.15
118.95
118.10
116.35
115.98
54.89
54.10
51.81
50.94
43.80
42.92
28.28
27.42
25.26
23.13
21.14
14.24
10
H2N
O
N
H
N
HO
OH
HN
NH
S
1.00×10^8
9.00×10^7
8.00×10^7
7.00×10^7
6.00×10^7
5.00×10^7
4.00×10^7
3.00×10^7
2.00×10^7
1.00×10^7
0.00
-1.00×10^7
180 170 160 150 140 130 120 110 100 90 80 70 60 50 40 30 20 10 0
f1 (ppm)

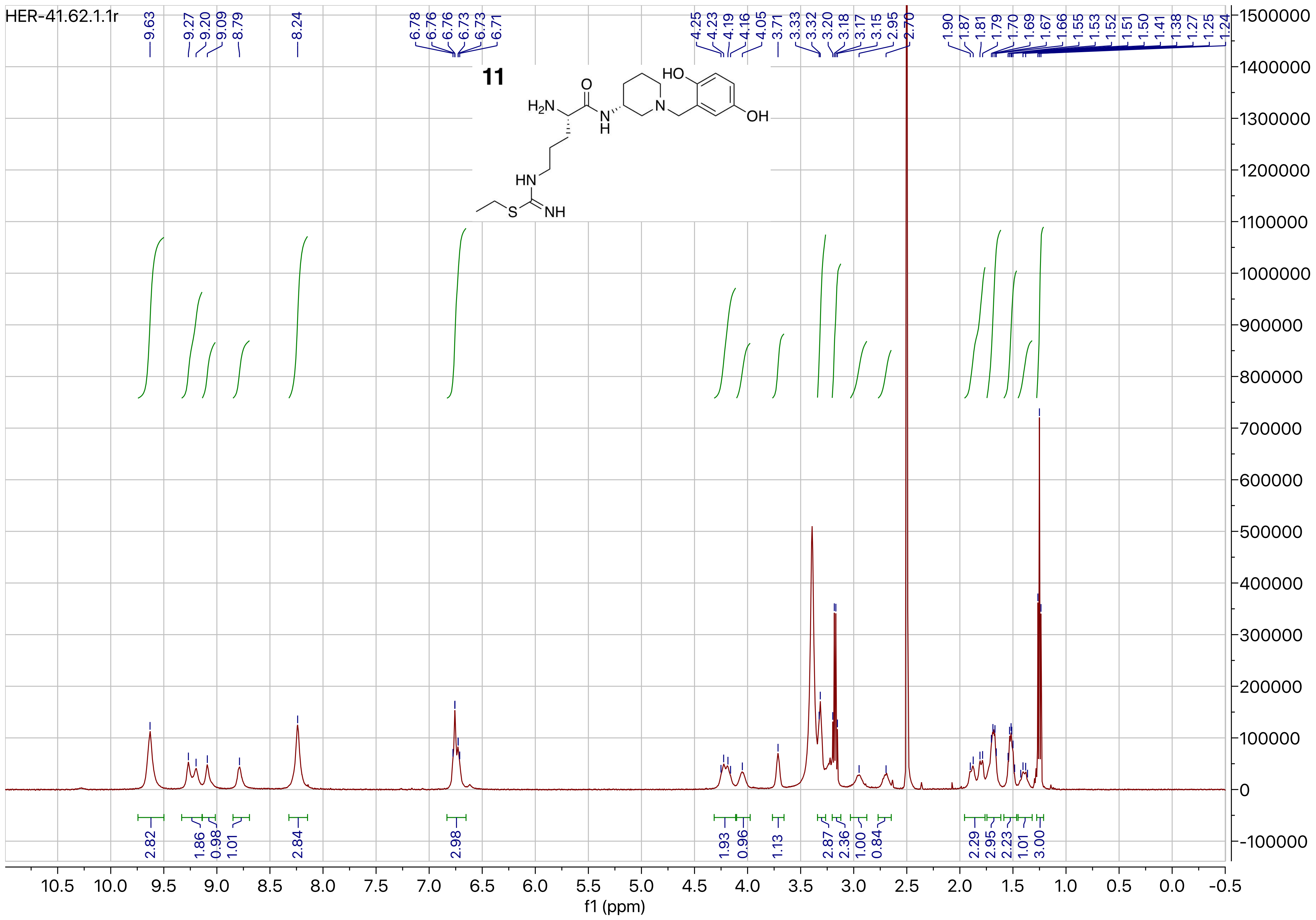
HER-41.62.1.1r
9.63
9.27
9.20
9.09
8.79
8.24
6.78
6.76
6.76
6.73
6.73
6.71
4.25
4.23
4.19
4.16
4.05
3.71
3.33
3.32
3.20
3.18
3.17
3.15
2.95
2.70
1.90
1.87
1.81
1.79
1.70
1.69
1.67
1.66
1.55
1.53
1.52
1.51
1.50
1.41
1.38
1.27
1.25
1.24
11
H2N
O
N
H
N
HO
OH
HN
S
NH
2.82
1.86
0.98
1.01
2.84
2.98
1.93
0.96
1.13
2.87
2.36
1.00
0.84
2.29
2.95
2.23
1.01
3.00
f1 (ppm)

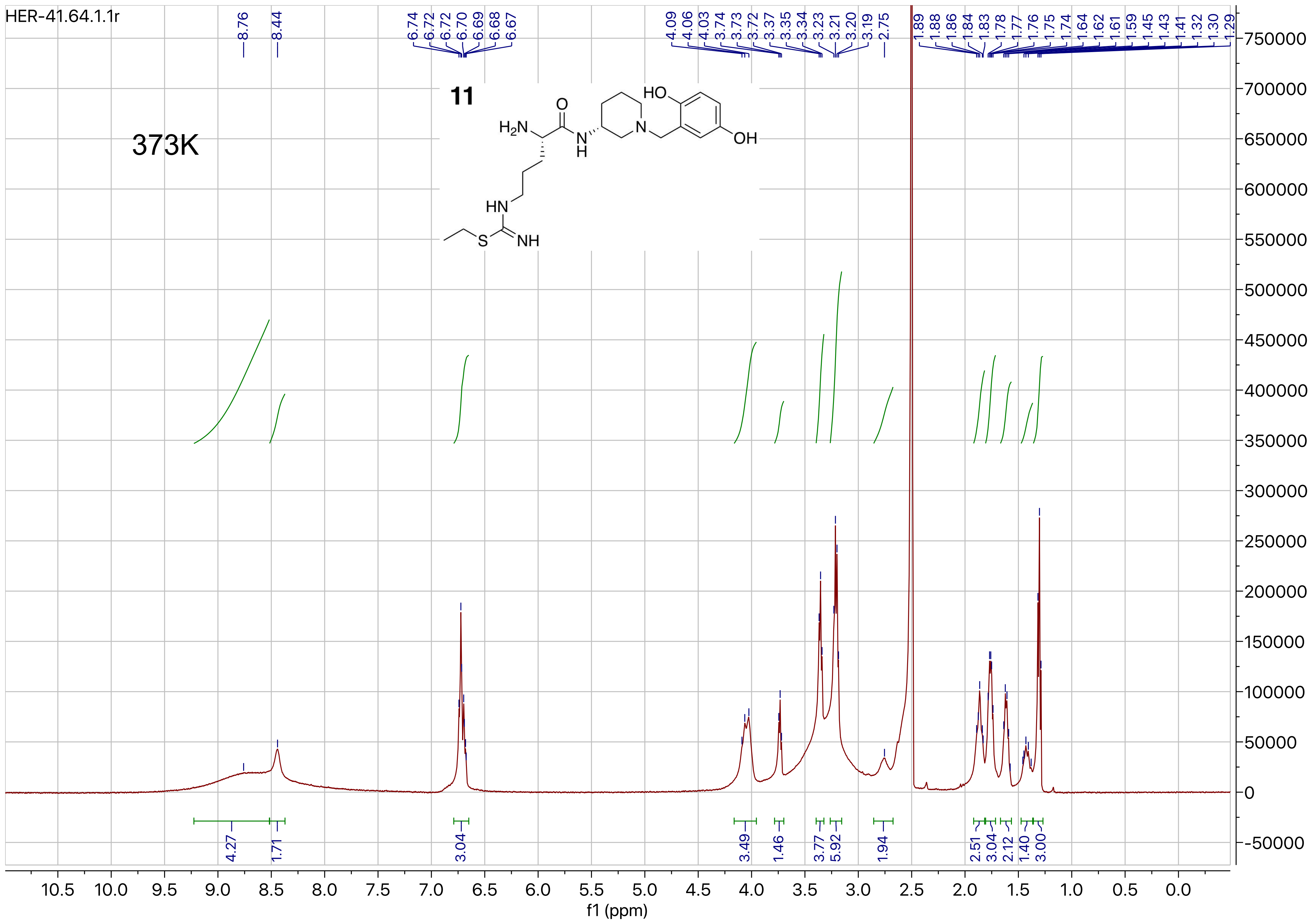
HER-41.64.1.1r
373K
11
H2N
O
N
H
N
HO
OH
HN
S
NH
8.76
8.44
6.74
6.72
6.72
6.70
6.69
6.68
6.67
4.09
4.06
4.03
3.74
3.73
3.72
3.37
3.35
3.34
3.23
3.21
3.20
3.19
2.75
1.89
1.88
1.86
1.84
1.83
1.78
1.77
1.76
1.75
1.74
1.64
1.62
1.61
1.59
1.45
1.43
1.41
1.32
1.30
1.29
4.27
1.71
3.04
3.49
1.46
3.77
5.92
1.94
2.51
3.04
2.12
1.40
3.00
f1 (ppm)

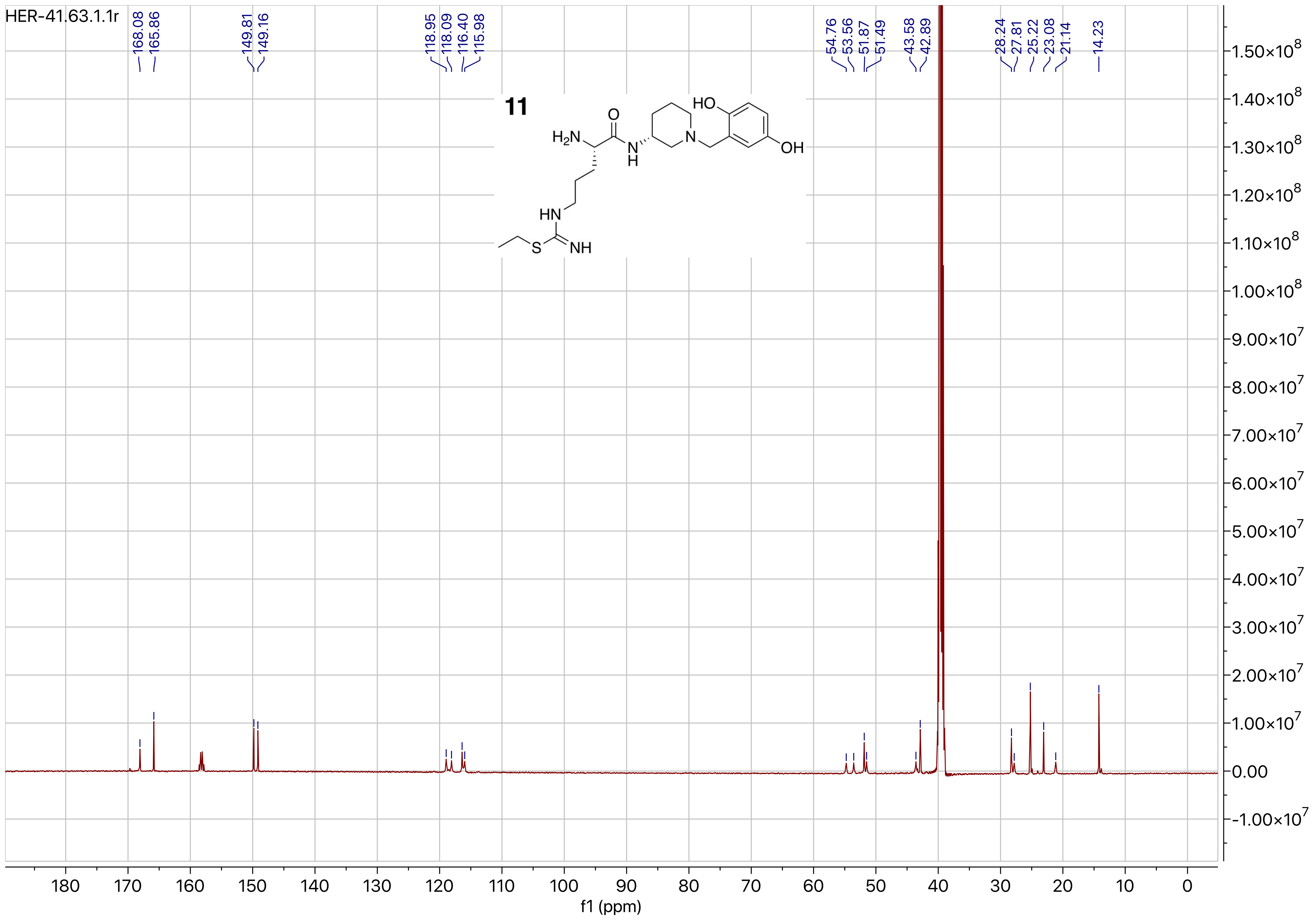
HER-41.63.1.1r
168.08
165.86
149.81
149.16
118.95
118.09
116.40
115.98
54.76
53.56
51.87
51.49
43.58
42.89
28.24
27.81
25.22
23.08
21.14
14.23
11
f1 (ppm)

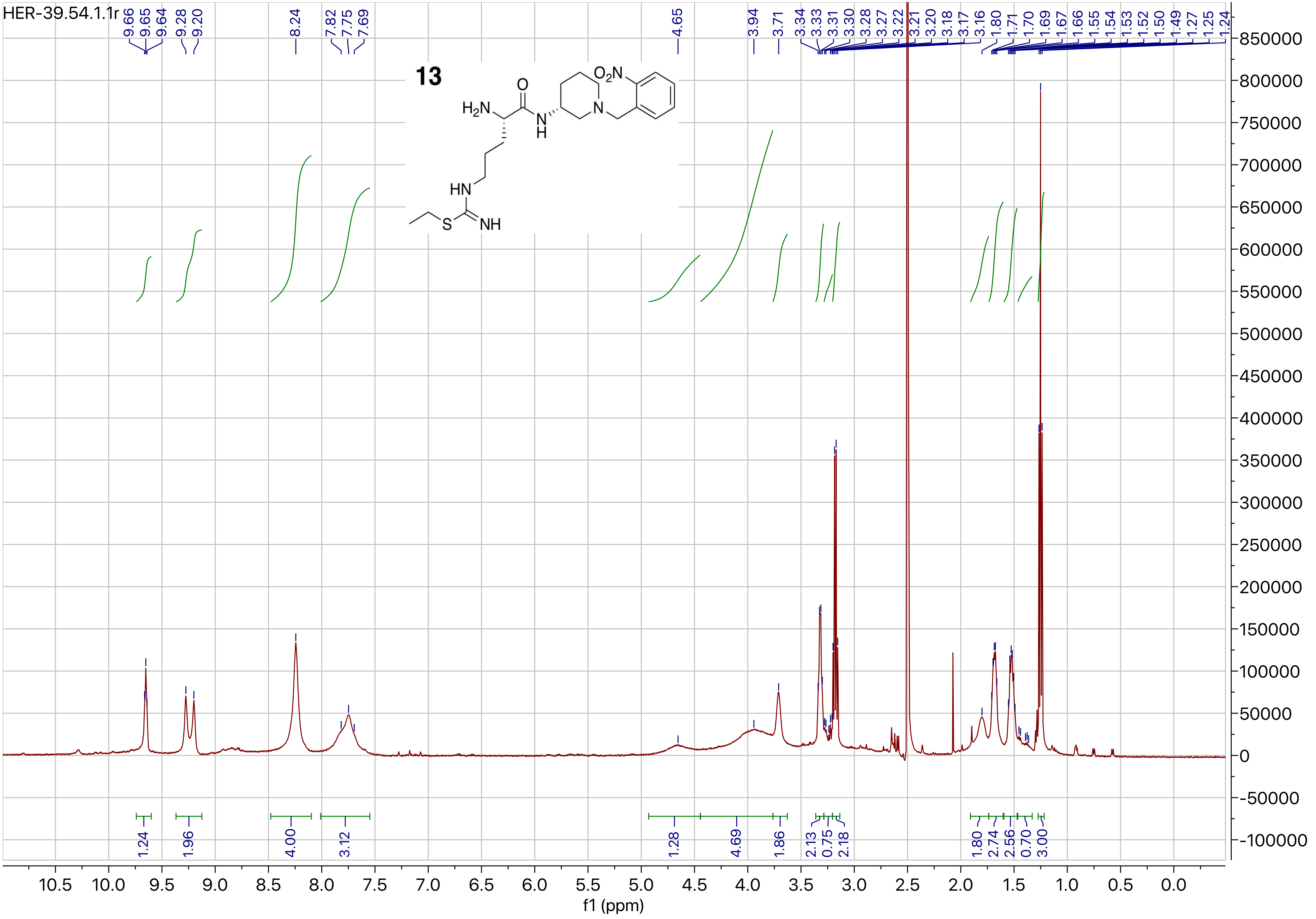
HER-39.54.1.1r
9.66 9.65 9.64 9.28 9.20 8.24 7.82 7.75 7.69 4.65 3.94 3.71 3.34 3.33 3.31 3.30 3.28 3.27 3.22 3.21 3.20 3.18 3.17 3.16 1.80 1.71 1.70 1.69 1.67 1.66 1.55 1.54 1.53 1.52 1.50 1.49 1.27 1.25 1.24
13
O_2N
O
H_2N
N
H
HN
S
NH
1.24 1.96 4.00 3.12 1.28 4.69 1.86 2.13 0.75 2.18 1.80 2.74 2.56 0.70 3.00
f1 (ppm)

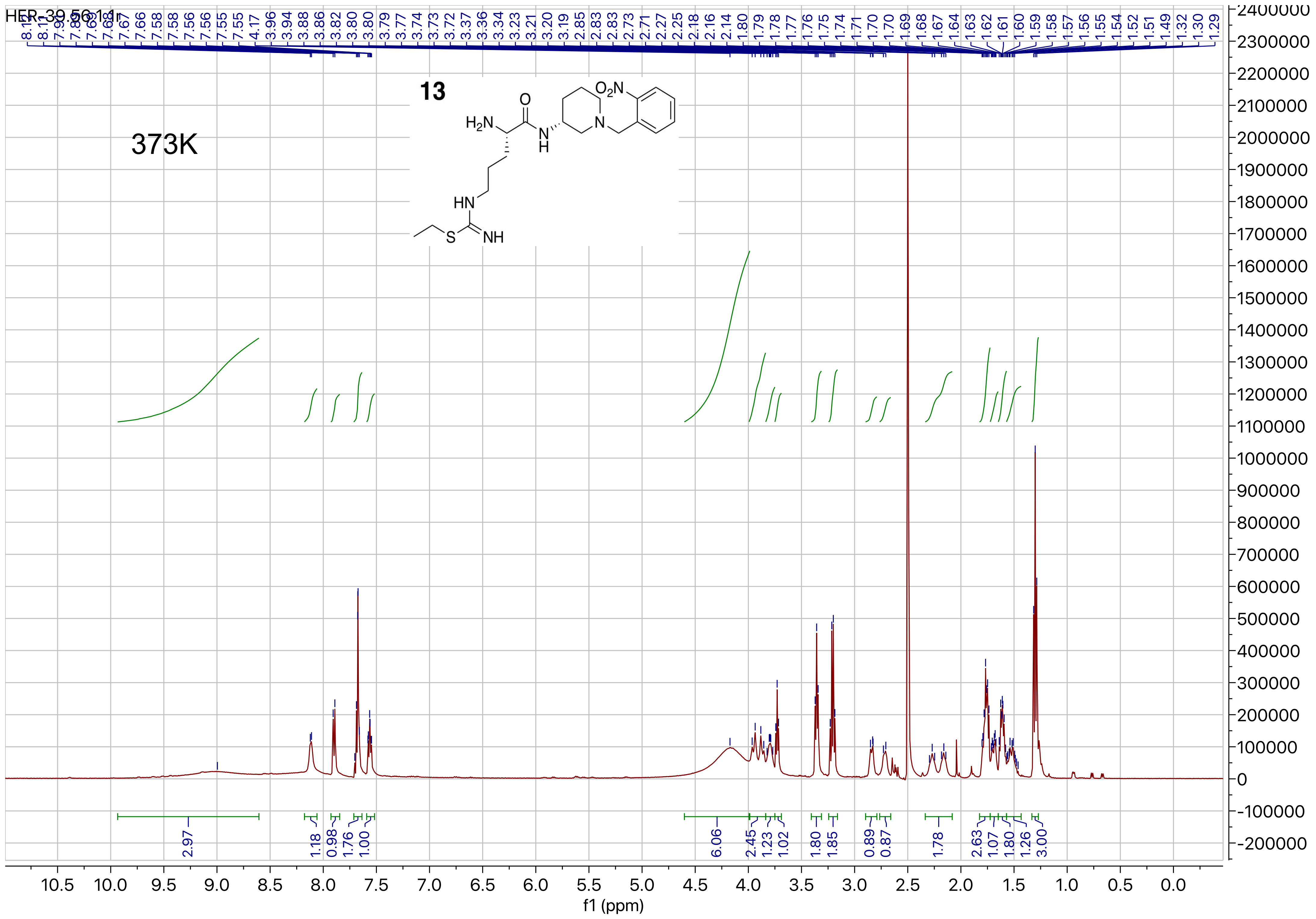
HER-39.56.1.1r
13
373K
f1 (ppm)

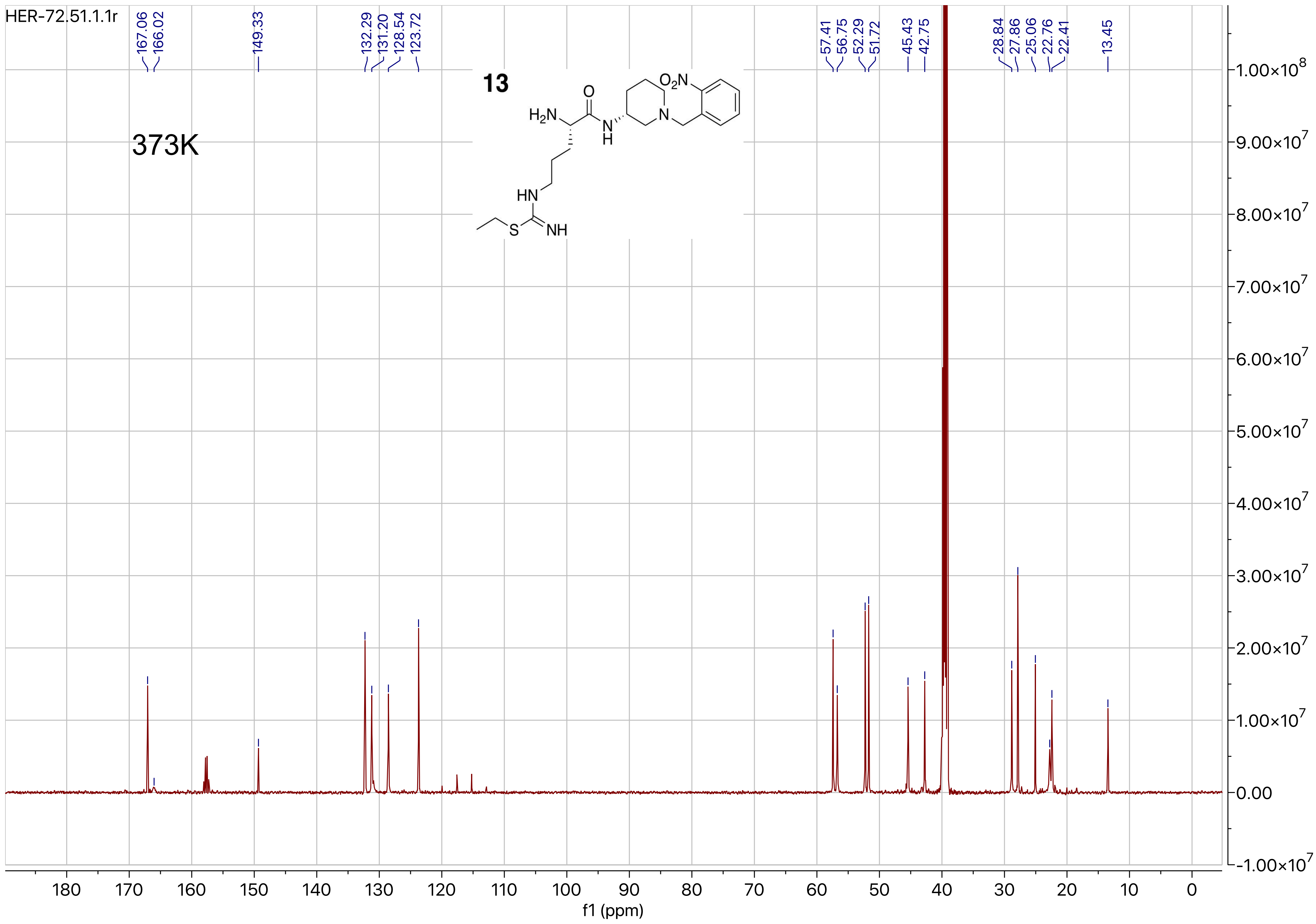
HER-72.51.1.1r
373K
13
167.06
166.02
149.33
132.29
131.20
128.54
123.72
57.41
56.75
52.29
51.72
45.43
42.75
28.84
27.86
25.06
22.76
22.41
13.45
H2N
O
N
H
N
O2N
HN
S
NH
1.00×10^8
9.00×10^7
8.00×10^7
7.00×10^7
6.00×10^7
5.00×10^7
4.00×10^7
3.00×10^7
2.00×10^7
1.00×10^7
0.00
-1.00×10^7
180
170
160
150
140
130
120
110
100
90
80
70
60
50
40
30
20
10
0
f1 (ppm)

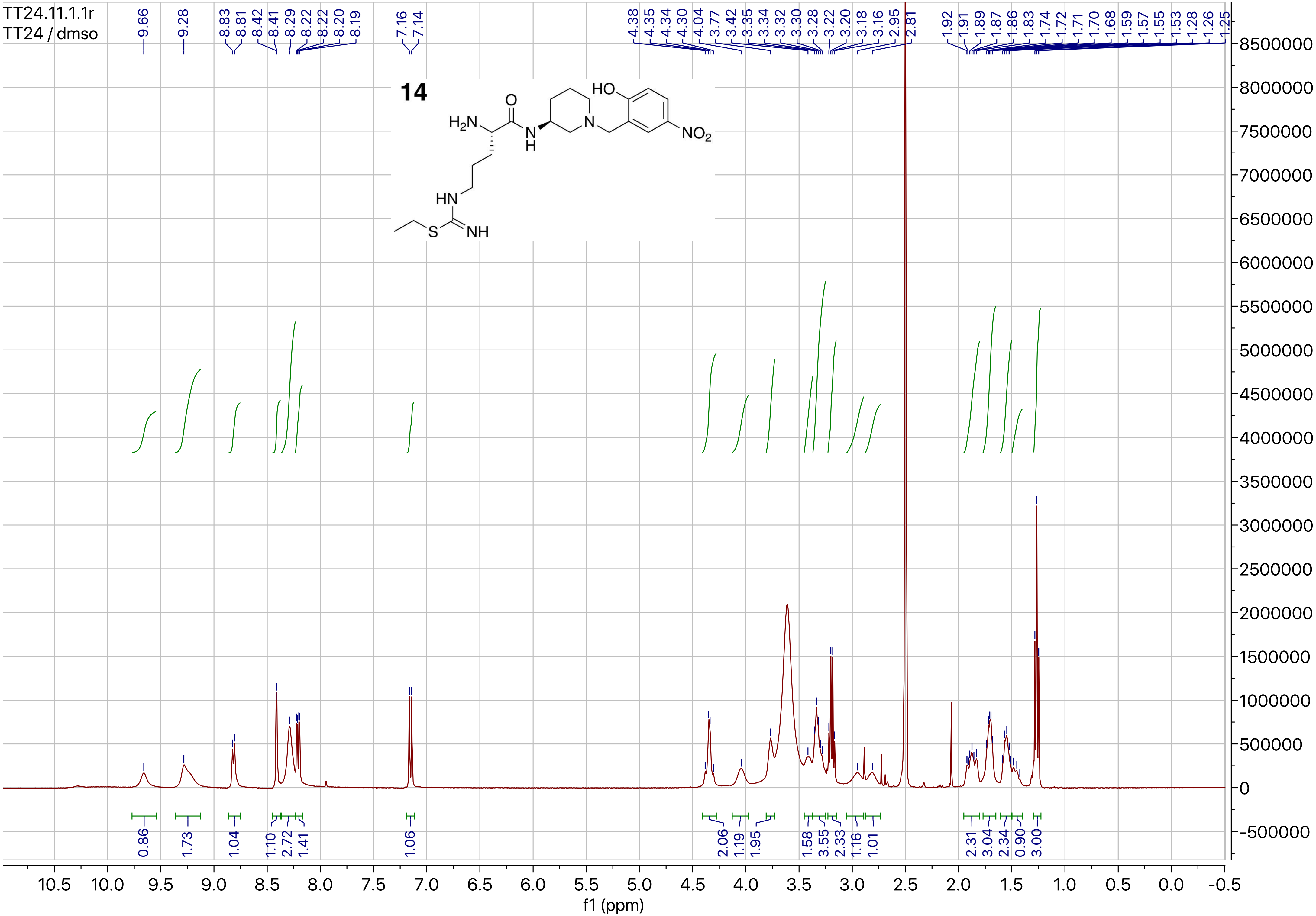

TT24.11.1.1r
TT24 / dmso
14
H2N
O
N
H
N
HO
NO2
HN
S
NH
f1 (ppm)

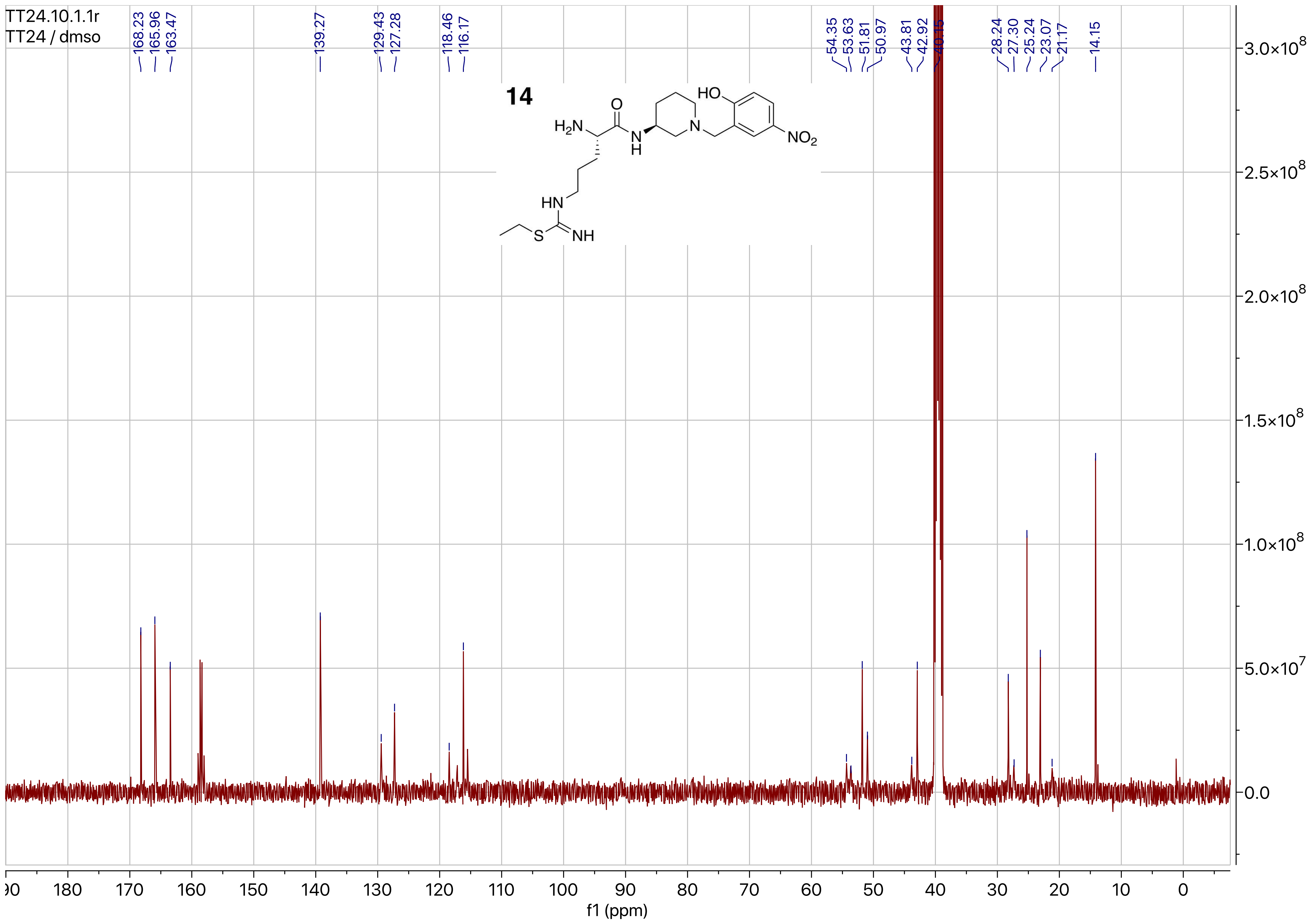

TT24.10.1.1r
TT24 / dmso
168.23
165.96
163.47
139.27
129.43
127.28
118.46
116.17
54.35
53.63
51.81
50.97
43.81
42.92
40.15
28.24
27.30
25.24
23.07
21.17
14.15
14
H2N
O
N
H
N
HO
NO2
HN
S
NH
f1 (ppm)

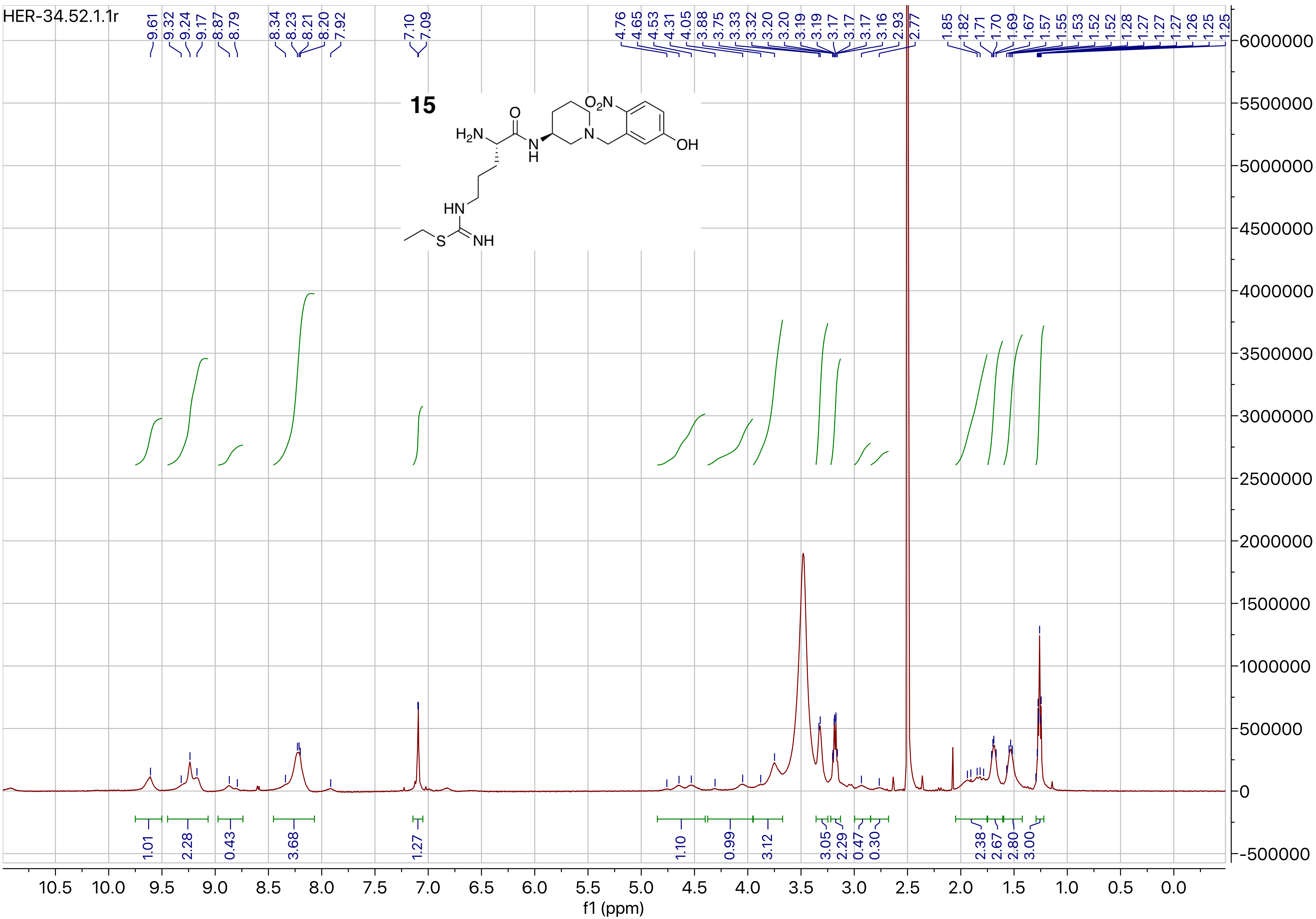

HER-34.52.1.1r
9.61
9.32
9.24
9.17
8.87
8.79
8.34
8.23
8.21
8.20
7.92
7.10
7.09
4.76
4.65
4.53
4.31
4.05
3.88
3.75
3.33
3.32
3.20
3.20
3.19
3.19
3.17
3.17
3.17
3.16
2.93
2.77
1.85
1.82
1.71
1.70
1.69
1.67
1.57
1.55
1.53
1.52
1.52
1.28
1.27
1.27
1.27
1.26
1.25
1.25
15
H_2N
O
N
H
O_2N
N
OH
HN
S
NH
1.01
2.28
0.43
3.68
1.27
1.10
0.99
3.12
3.05
2.29
0.47
0.30
2.38
2.67
2.80
3.00
f1 (ppm)

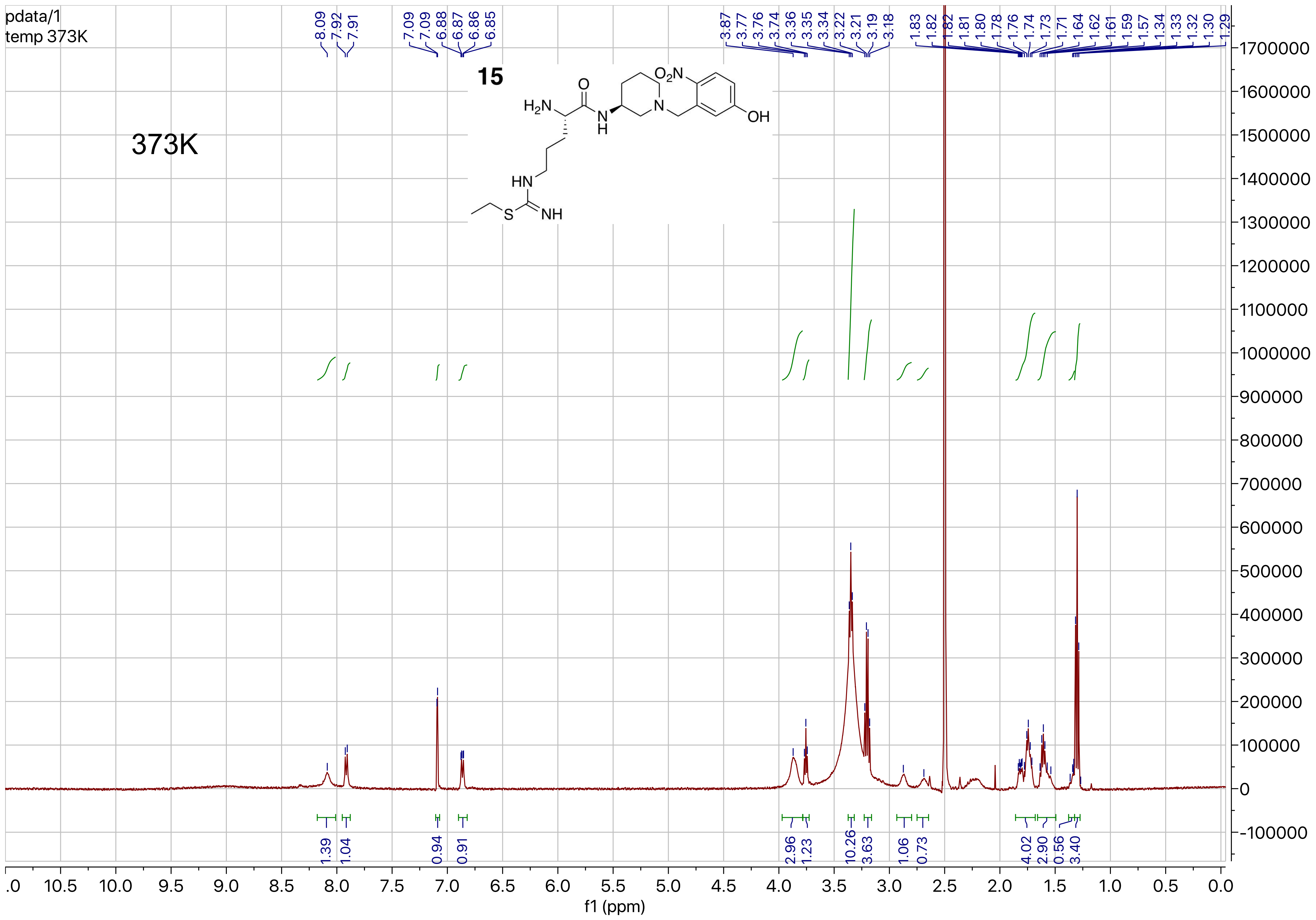
pdata/1
temp 373K
373K
15
H2N
O
N
H
N
O2N
OH
HN
S
NH
8.09 7.92 7.91
7.09 7.09 6.88 6.87 6.86 6.85
3.87 3.77 3.76 3.74 3.36 3.35 3.34 3.22 3.21 3.19 3.18
1.83 1.82 1.82 1.81 1.80 1.78 1.76 1.74 1.73 1.71 1.64 1.62 1.61 1.59 1.57 1.34 1.33 1.32 1.30 1.29
1.39 1.04 0.94 0.91 2.96 1.23 10.26 3.63 1.06 0.73 4.02 2.90 0.56 3.40
f1 (ppm)

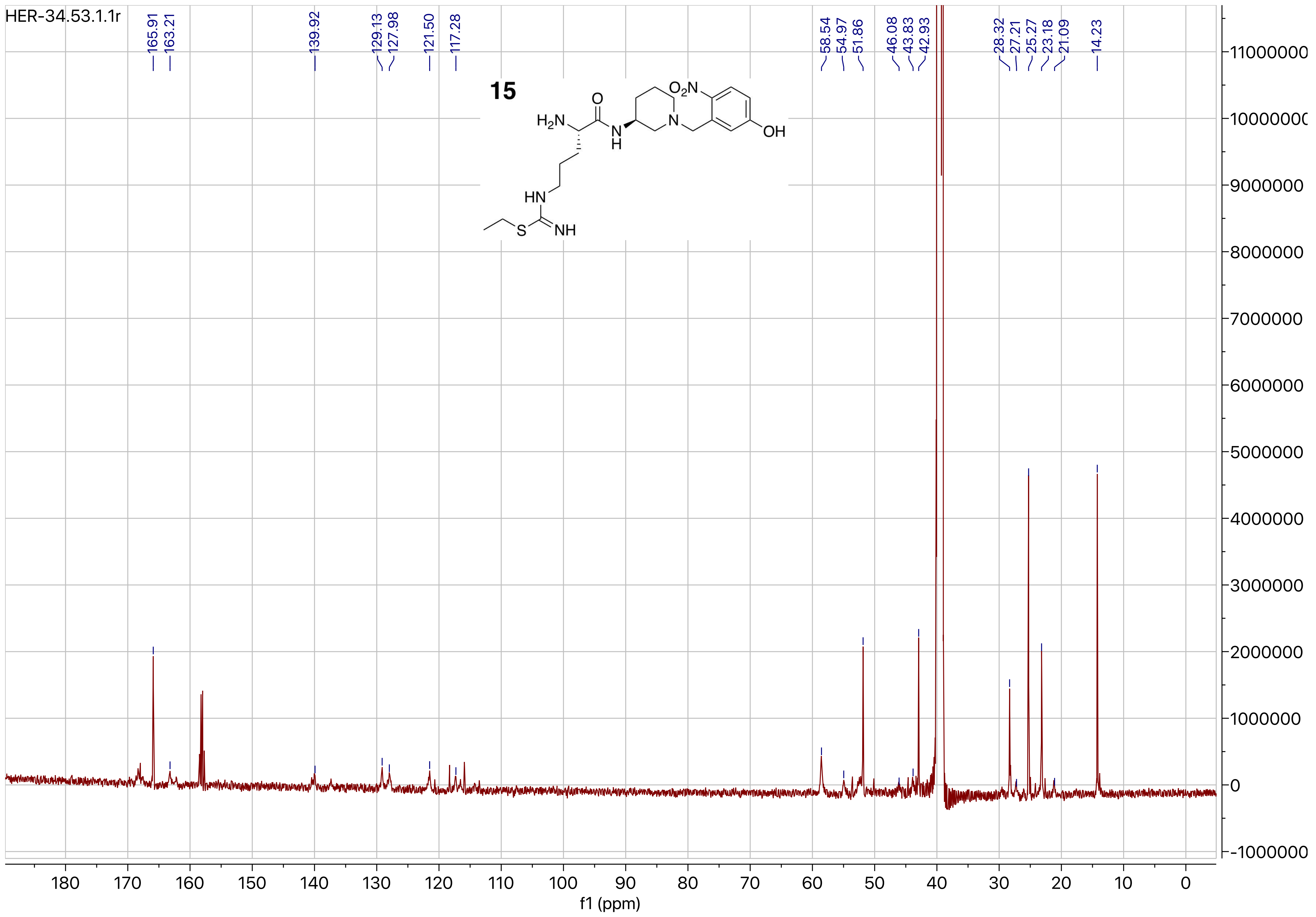
HER-34.53.1.1r
165.91
163.21
139.92
129.13
127.98
121.50
117.28
58.54
54.97
51.86
46.08
43.83
42.93
28.32
27.21
25.27
23.18
21.09
14.23
15
H_2N
O
N
H
O_2N
N
OH
HN
S
NH
11000000
9000000
8000000
7000000
6000000
5000000
4000000
3000000
2000000
1000000
0
-1000000
180
170
160
150
140
130
120
110
100
90
80
70
60
50
40
30
20
10
0
f1 (ppm)

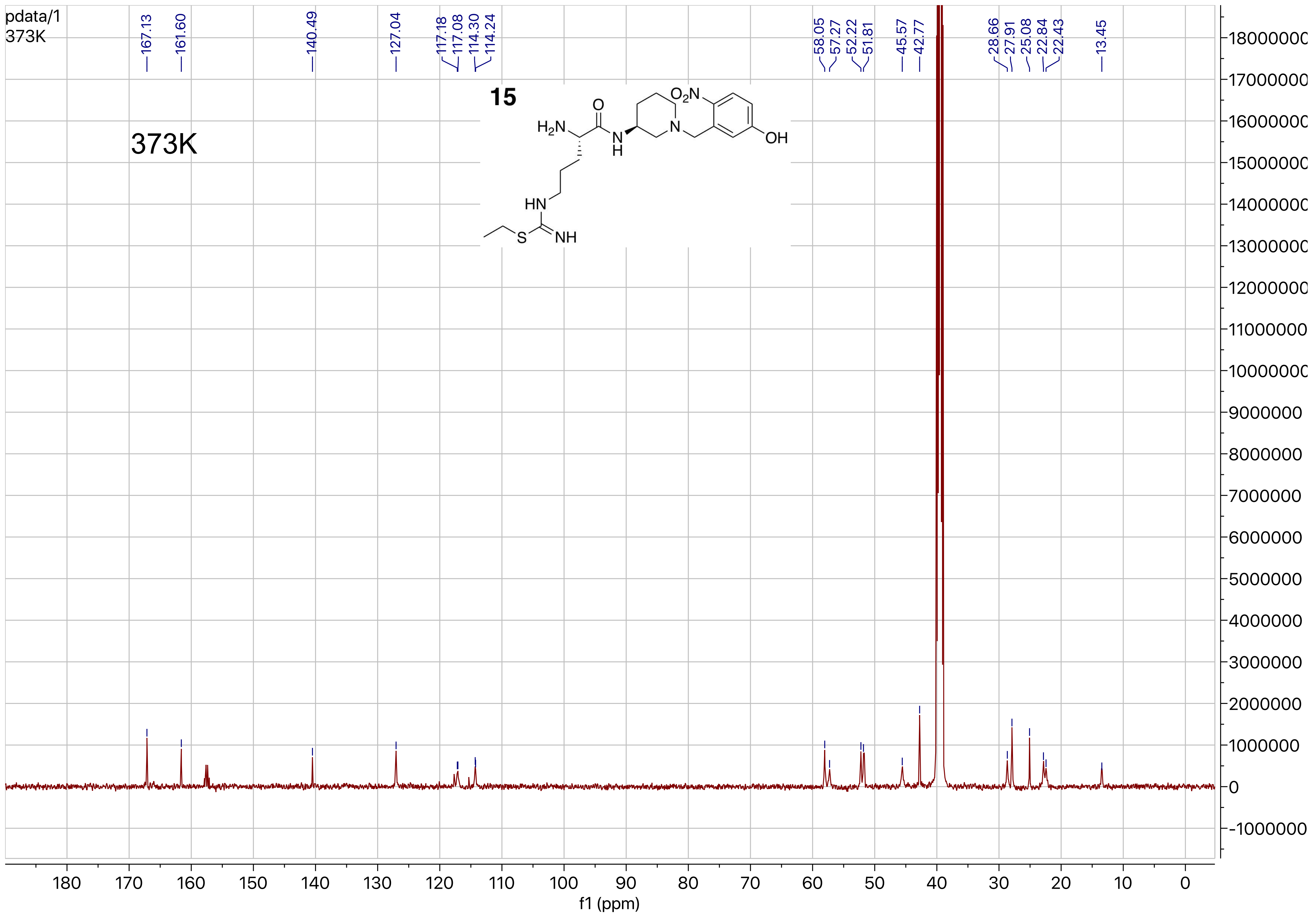
pdata/1
373K
167.13
161.60
140.49
127.04
117.18
117.08
114.30
114.24
58.05
57.27
52.22
51.81
45.57
42.77
28.66
27.91
25.08
22.84
22.43
13.45
15
373K
H2N
O
N
H
O2N
N
OH
HN
S
NH
f1 (ppm)

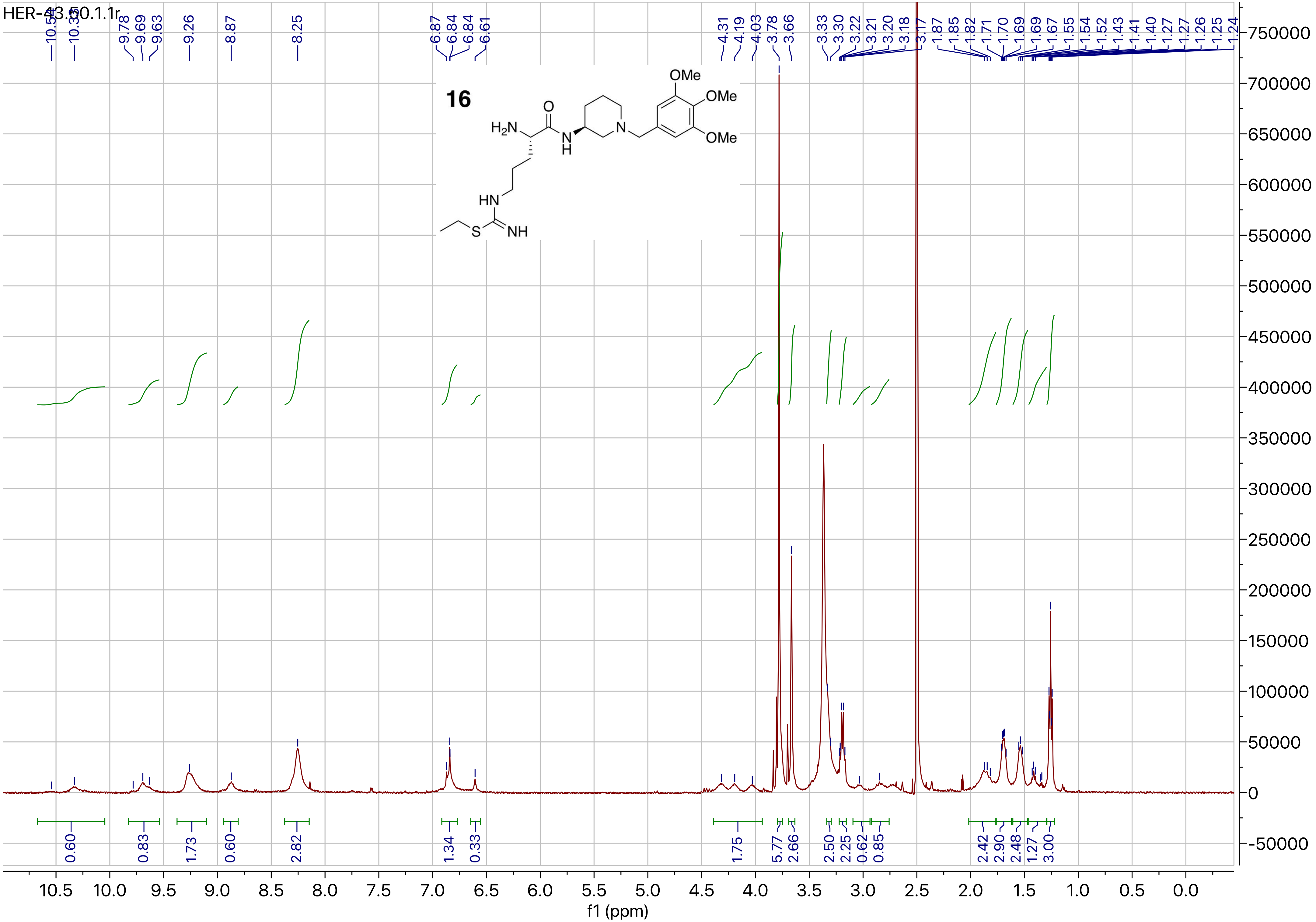

HER-43.50.1.1r
9.78
9.69
9.63
9.26
8.87
8.25
6.87
6.84
6.84
6.61
4.31
4.19
4.03
3.78
3.66
3.33
3.30
3.22
3.21
3.20
3.18
3.17
1.87
1.85
1.82
1.71
1.70
1.69
1.69
1.67
1.55
1.54
1.52
1.43
1.41
1.40
1.27
1.27
1.26
1.25
1.24
16
OMe
OMe
OMe
O
H2N
N
H
N
HN
S
NH
0.60
0.83
1.73
0.60
2.82
1.34
0.33
1.75
5.77
2.66
2.50
2.25
0.62
0.85
2.42
2.90
2.48
1.27
3.00
f1 (ppm)

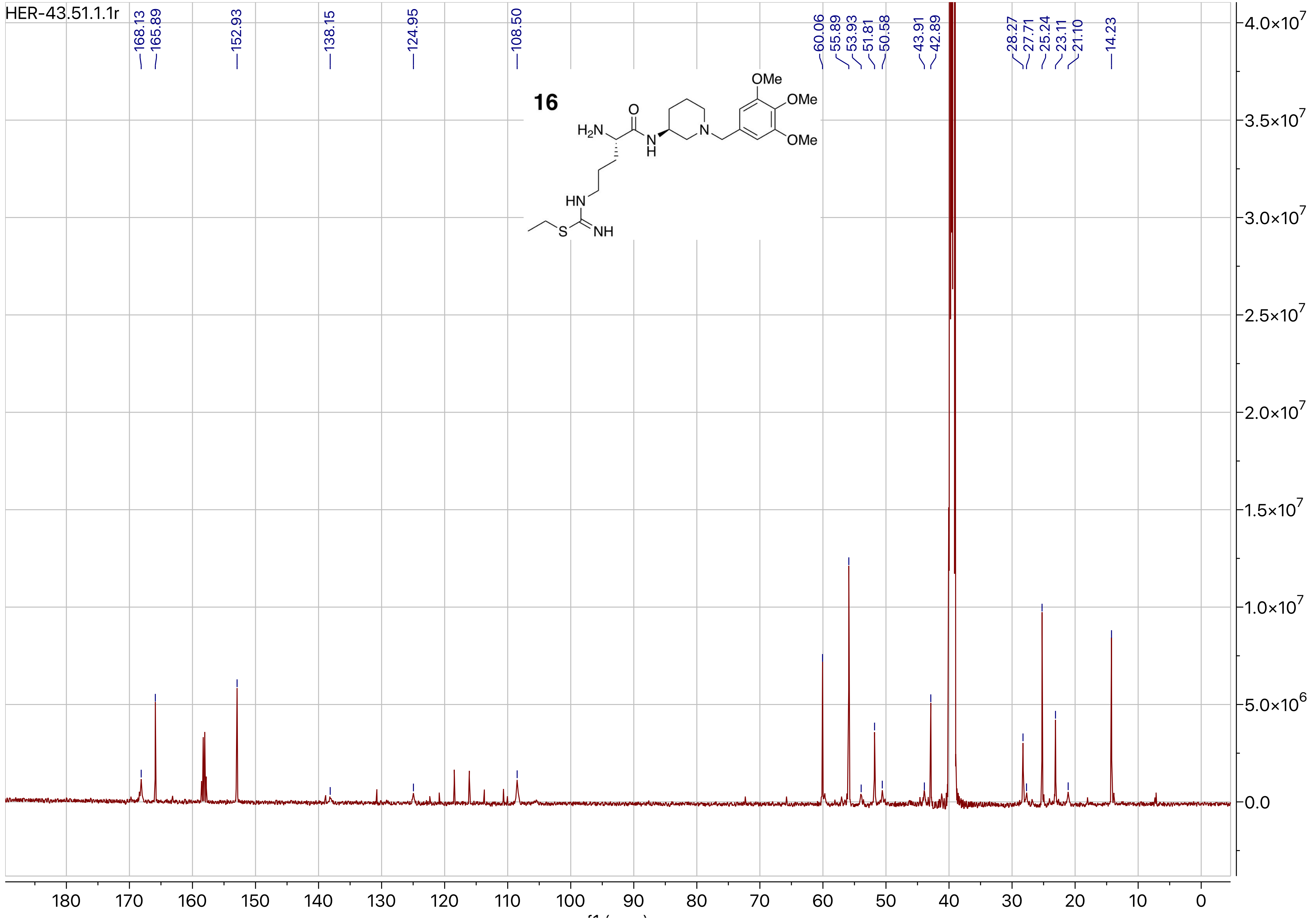
HER-43.51.1.1r
168.13
165.89
152.93
138.15
124.95
108.50
60.06
55.89
53.93
51.81
50.58
43.91
42.89
28.27
27.71
25.24
23.11
21.10
14.23
16
OMe
OMe
OMe
O
H2N
N
H
N
HN
S
NH
4.0×10^7
3.5×10^7
3.0×10^7
2.5×10^7
2.0×10^7
1.5×10^7
1.0×10^7
5.0×10^6
0.0
180 170 160 150 140 130 120 110 100 90 80 70 60 50 40 30 20 10 0

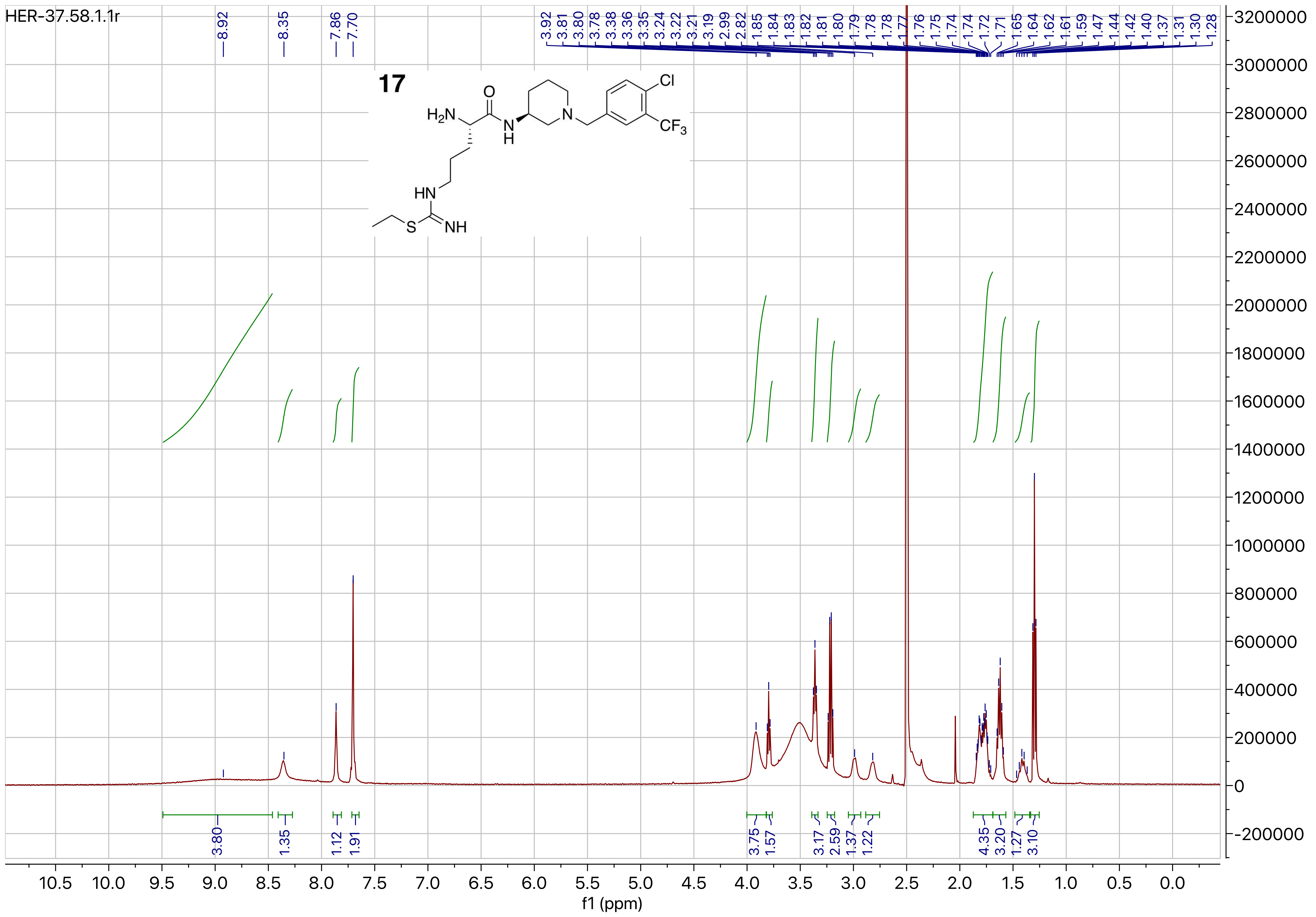
HER-37.58.1.1r
8.92
8.35
7.86
7.70
3.92 3.81 3.80 3.78 3.38 3.36 3.35 3.24 3.22 3.21 3.19 2.99 2.82 1.85 1.84 1.83 1.82 1.81 1.80 1.79 1.78 1.78 1.77 1.76 1.75 1.74 1.74 1.72 1.71 1.65 1.64 1.62 1.61 1.59 1.47 1.44 1.42 1.40 1.37 1.31 1.30 1.28
17
H2N
O
N
H
N
Cl
CF3
HN
S
NH
3.80
1.35
1.12
1.91
3.75
1.57
3.17
2.59
1.37
1.22
4.35
3.20
1.27
3.10
f1 (ppm)

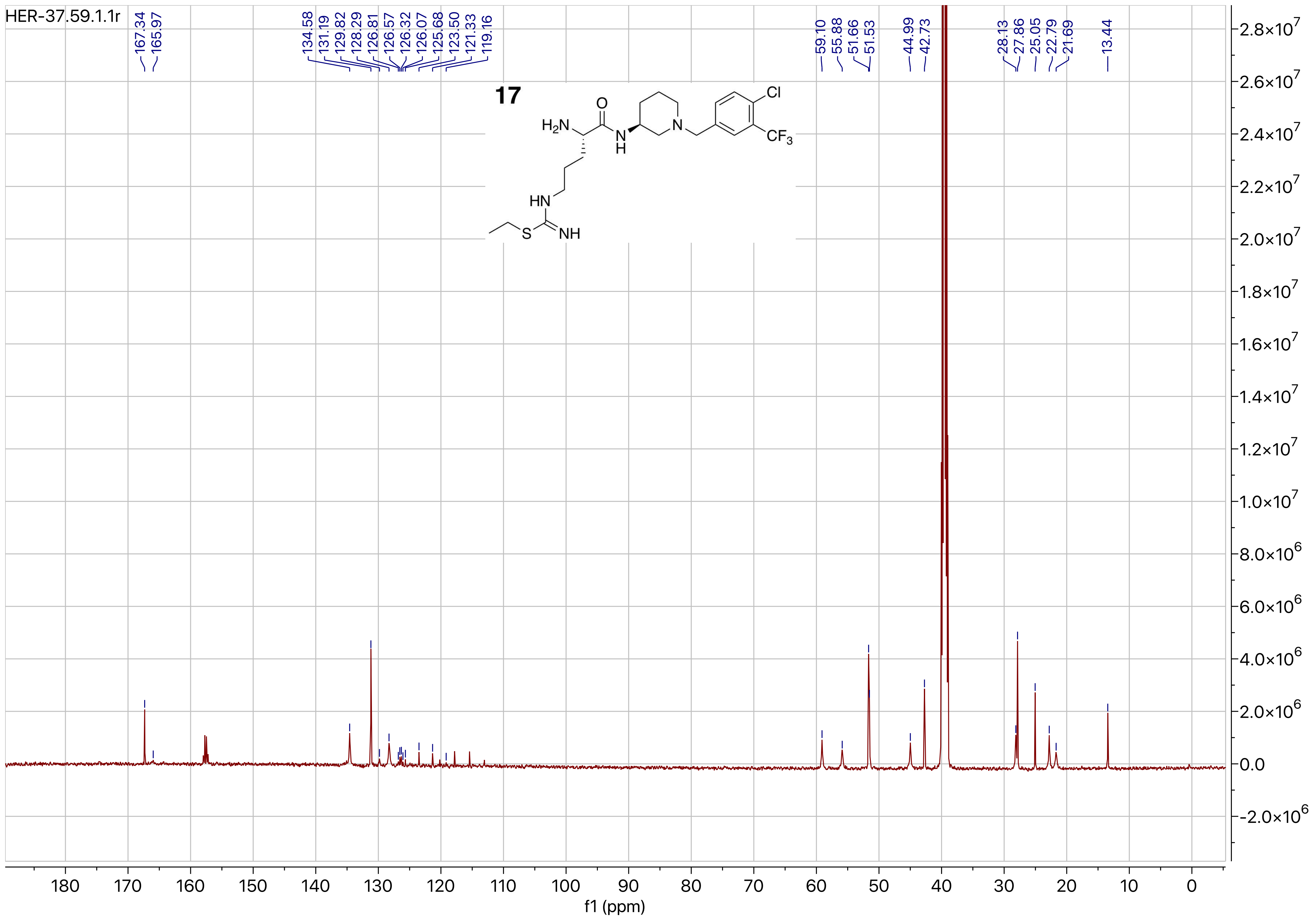
HER-37.59.1.1r
167.34
165.97
134.58
131.19
129.82
128.29
126.81
126.57
126.32
126.07
125.68
123.50
121.33
119.16
59.10
55.88
51.66
51.53
44.99
42.73
28.13
27.86
25.05
22.79
21.69
13.44
17
O
H2N
N
H
N
Cl
CF3
HN
S
NH
f1 (ppm)

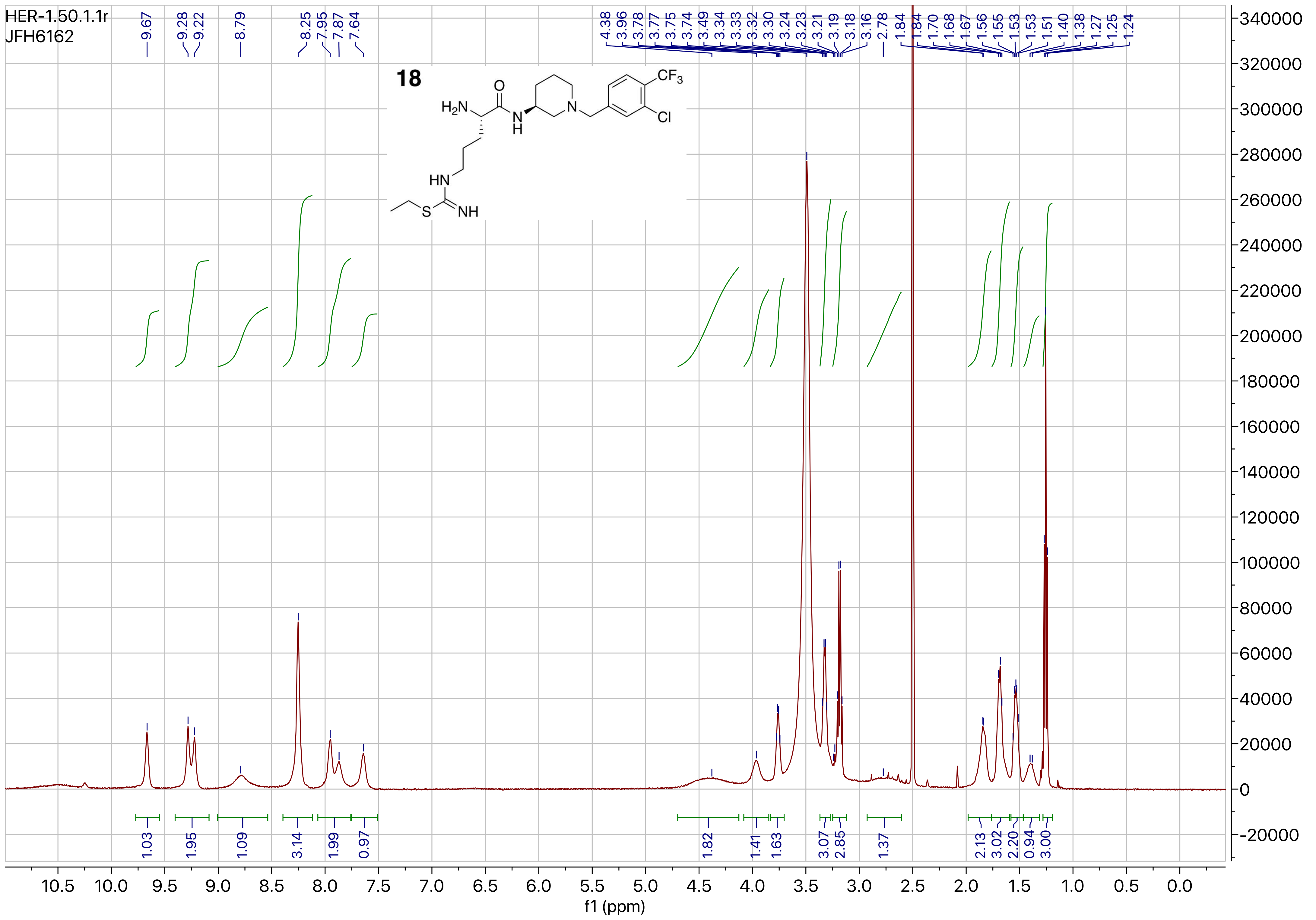

HER-1.50.1.1r
JFH6162
18
H2N
O
N
H
N
CF3
Cl
HN
S
NH
9.67
9.28
9.22
8.79
8.25
7.95
7.87
7.64
4.38
3.96
3.78
3.77
3.75
3.74
3.49
3.34
3.33
3.32
3.30
3.24
3.23
3.21
3.19
3.18
3.16
2.78
1.84
1.84
1.70
1.68
1.67
1.56
1.55
1.53
1.53
1.51
1.40
1.38
1.27
1.25
1.24
1.03
1.95
1.09
3.14
1.99
0.97
1.82
1.41
1.63
3.07
2.85
1.37
2.13
3.02
2.20
0.94
3.00
f1 (ppm)

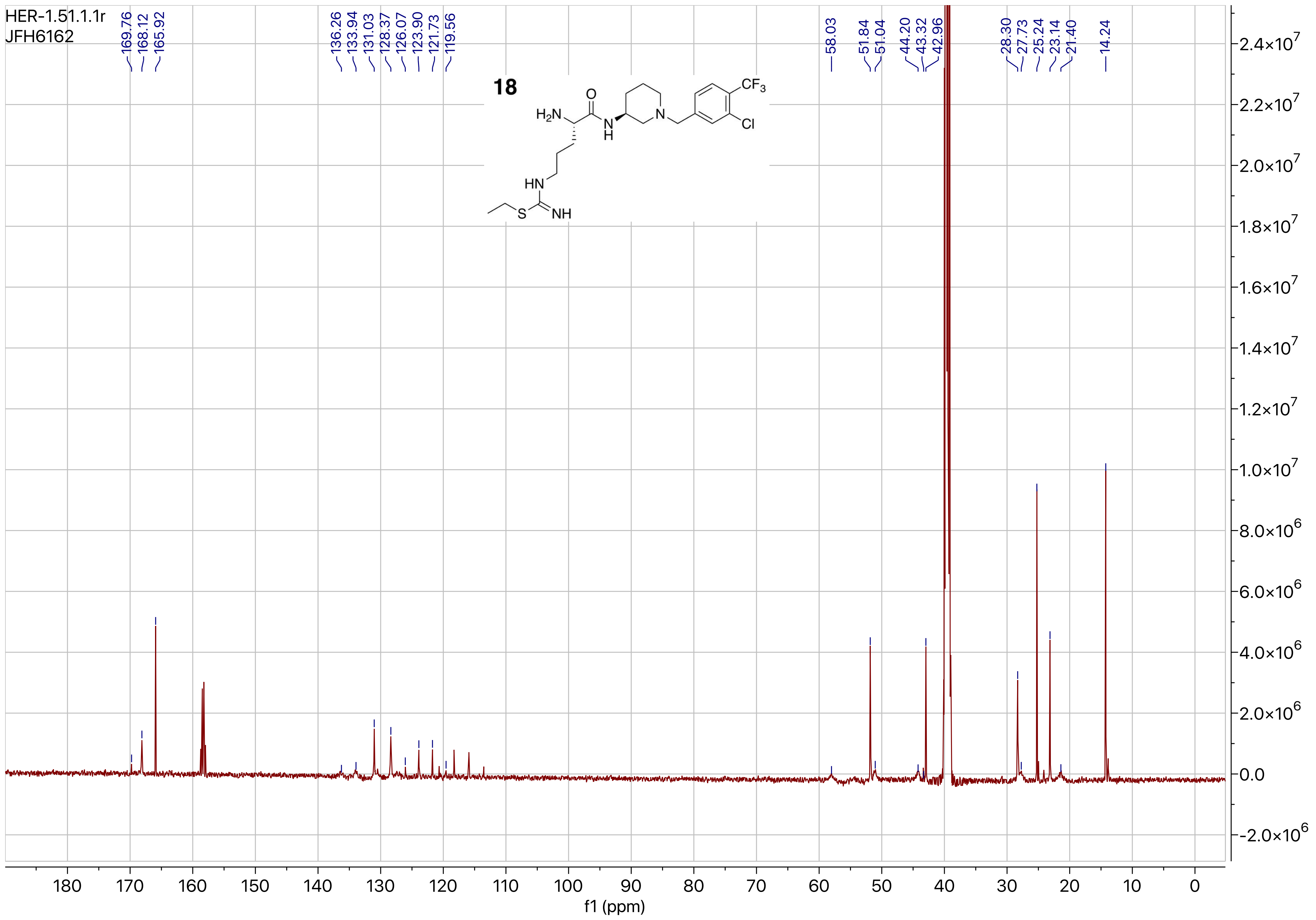
HER-1.51.1.1r
JFH6162
169.76
168.12
165.92
136.26
133.94
131.03
128.37
126.07
123.90
121.73
119.56
58.03
51.84
51.04
44.20
43.32
42.96
28.30
27.73
25.24
23.14
21.40
14.24
18
O
H_2N
N
H
N
CF_3
Cl
HN
S
NH
f1 (ppm)

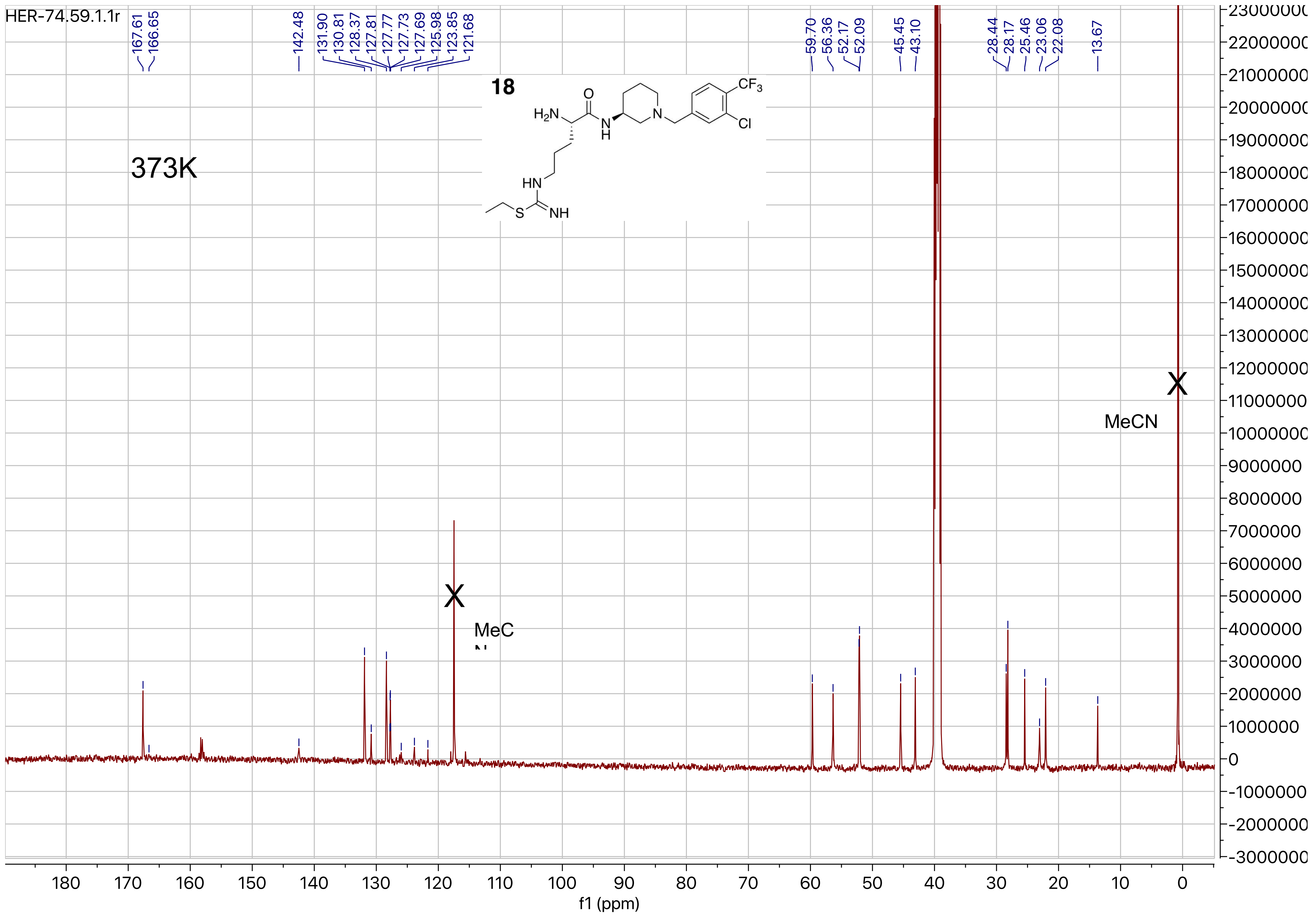

HER-74.59.1.1r
167.61
166.65
142.48
131.90
130.81
128.37
127.81
127.77
127.73
127.69
125.98
123.85
121.68
59.70
56.36
52.17
52.09
45.45
43.10
28.44
28.17
25.46
23.06
22.08
13.67
18
373K
MeCN
X
f1 (ppm)

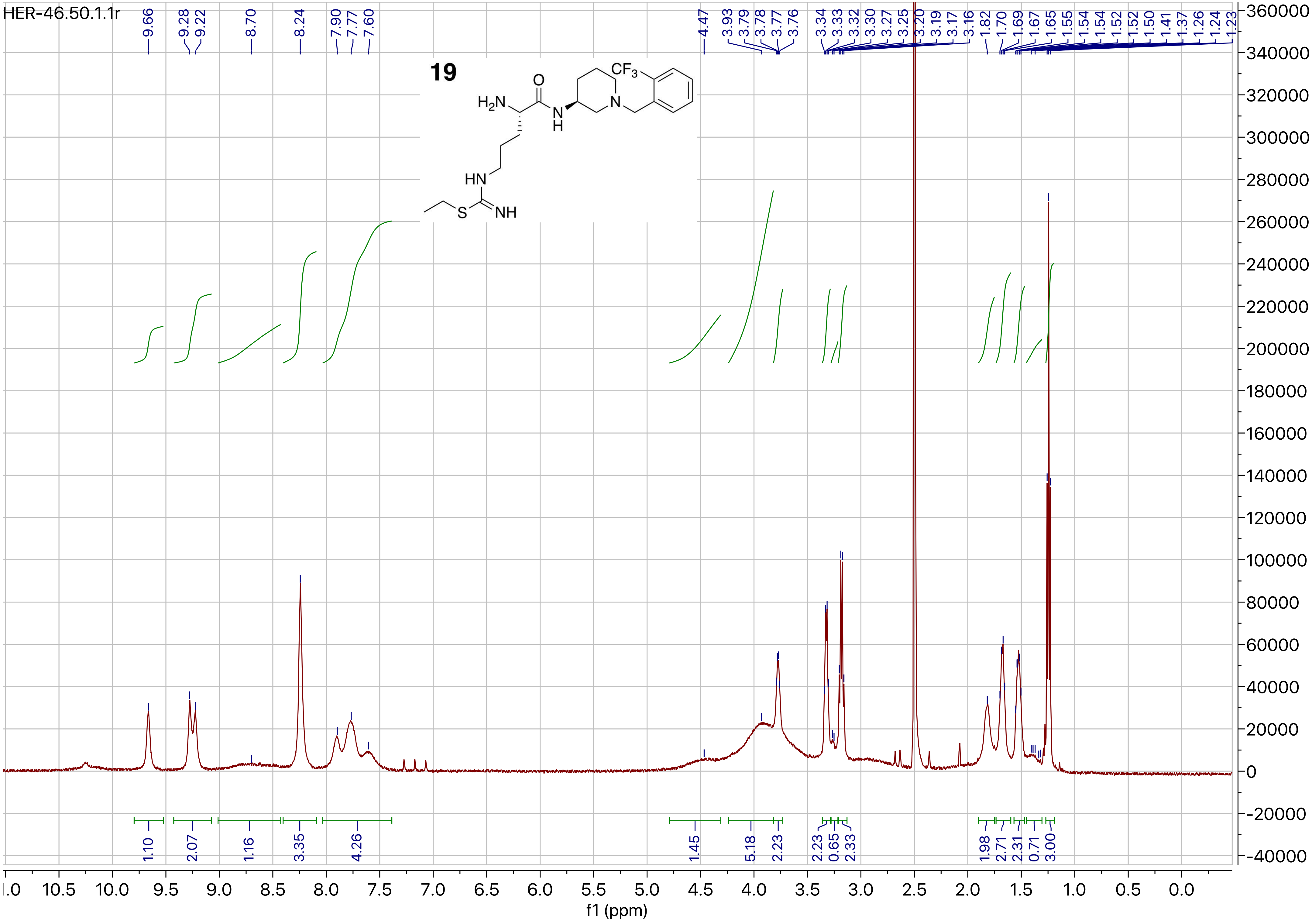
HER-46.50.1.1r
9.66
9.28
9.22
8.70
8.24
7.90
7.77
7.60
4.47
3.93
3.79
3.78
3.77
3.76
3.34
3.33
3.32
3.30
3.27
3.25
3.20
3.19
3.17
3.16
1.82
1.70
1.69
1.67
1.65
1.55
1.54
1.54
1.52
1.52
1.50
1.41
1.37
1.26
1.24
1.23
19
O
CF_3
H_2N
N
H
N
HN
S
NH
1.10
2.07
1.16
3.35
4.26
1.45
5.18
2.23
2.23
0.65
2.33
1.98
2.71
2.31
0.71
3.00
f1 (ppm)

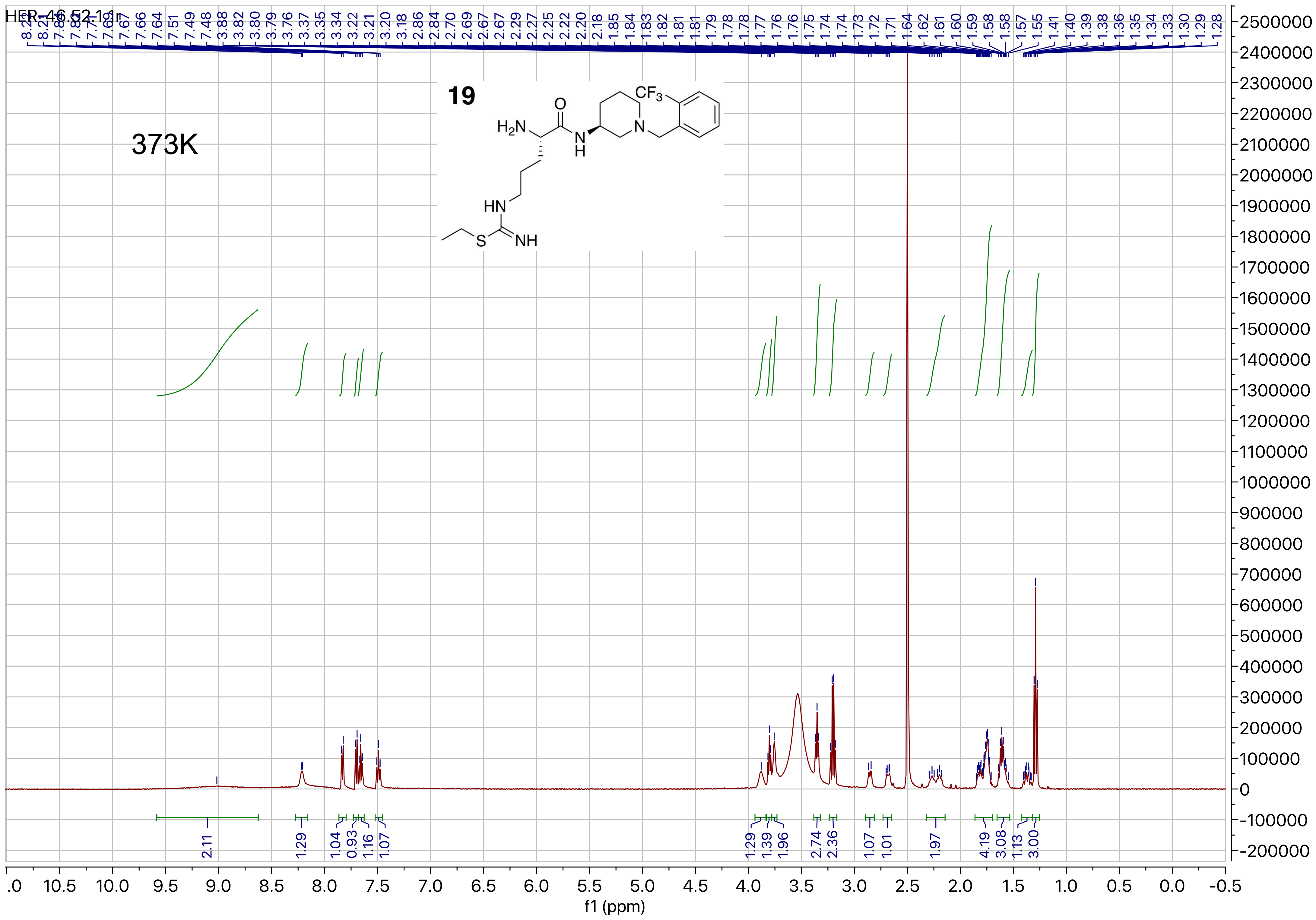
373K
19
H2N
O
N
H
CF3
N
HN
S
NH
7.67
7.66
7.64
7.51
7.49
7.48
3.88
3.82
3.80
3.79
3.76
3.37
3.35
3.34
3.22
3.21
3.20
3.18
2.86
2.84
2.70
2.69
2.67
2.67
2.29
2.27
2.25
2.22
2.20
2.18
1.85
1.84
1.83
1.82
1.81
1.81
1.79
1.78
1.78
1.77
1.76
1.76
1.75
1.74
1.74
1.73
1.72
1.71
1.64
1.62
1.61
1.60
1.59
1.58
1.58
1.57
1.55
1.41
1.40
1.39
1.38
1.36
1.35
1.34
1.33
1.30
1.29
1.28
2.11
1.29
1.04
0.93
1.16
1.07
1.29
1.39
1.96
2.74
2.36
1.07
1.01
1.97
4.19
3.08
1.13
3.00
f1 (ppm)

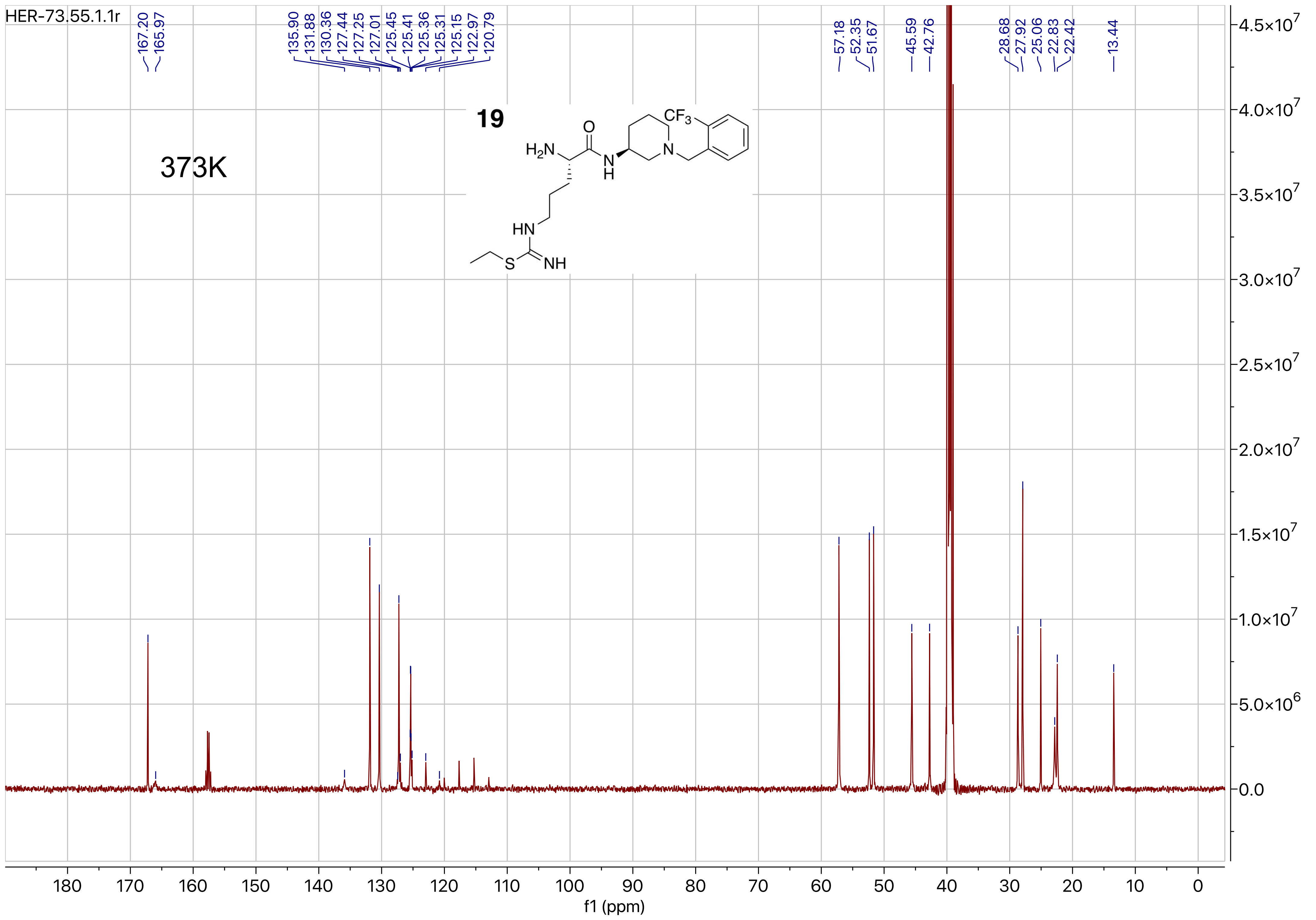
HER-73.55.1.1r
167.20
165.97
135.90
131.88
130.36
127.44
127.25
127.01
125.45
125.41
125.36
125.31
125.15
122.97
120.79
57.18
52.35
51.67
45.59
42.76
28.68
27.92
25.06
22.83
22.42
13.44
19
373K
CF3
O
H2N
N
H
HN
S
NH
4.5×10^7
4.0×10^7
3.5×10^7
3.0×10^7
2.5×10^7
2.0×10^7
1.5×10^7
1.0×10^7
5.0×10^6
0.0
180 170 160 150 140 130 120 110 100 90 80 70 60 50 40 30 20 10 0
f1 (ppm)

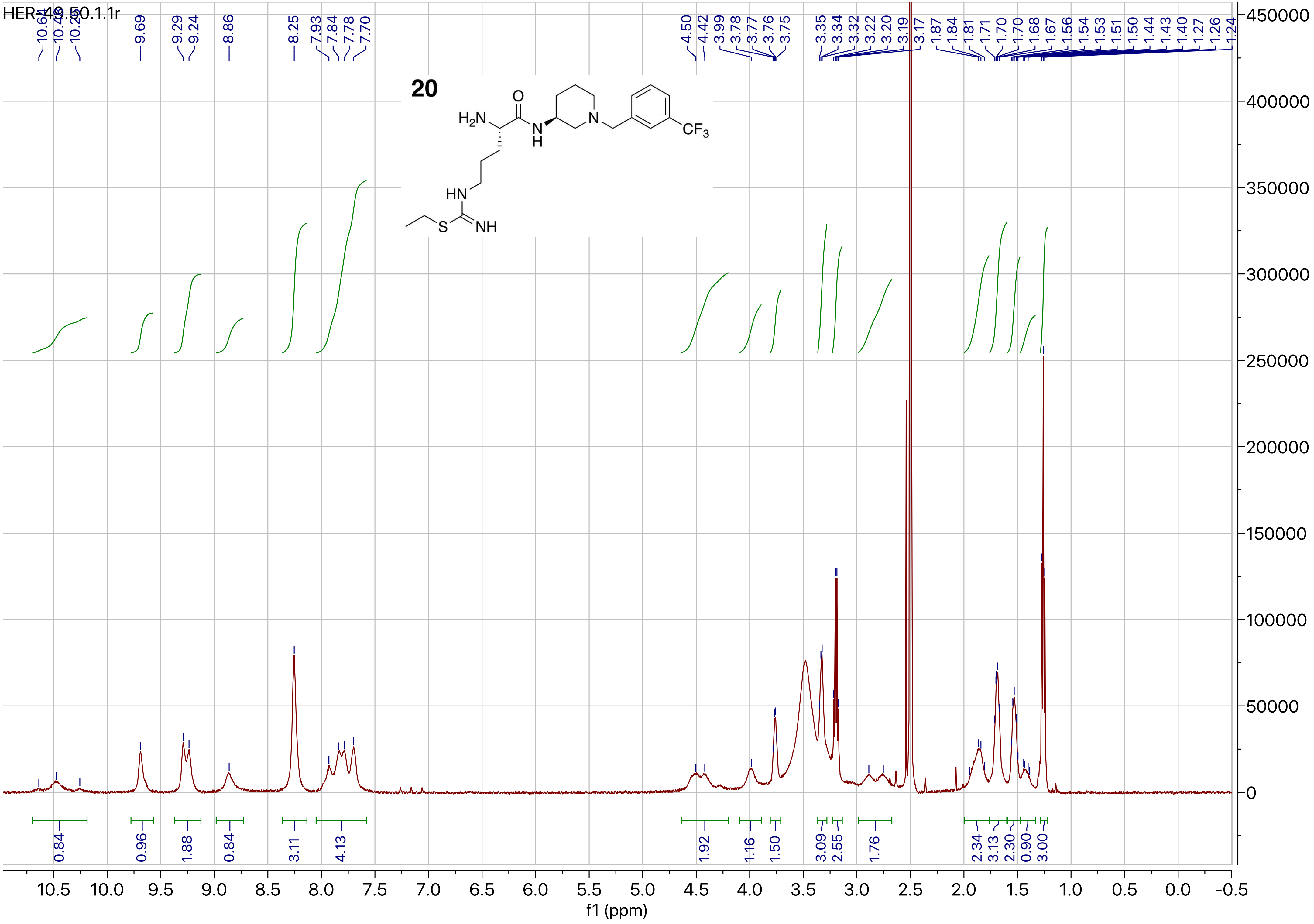
HER-49.50.1.1r
20
9.69
9.29
9.24
8.86
8.25
7.93
7.84
7.78
7.70
4.50
4.42
3.99
3.78
3.77
3.76
3.75
3.35
3.34
3.32
3.22
3.20
3.19
3.17
1.87
1.84
1.81
1.71
1.70
1.70
1.68
1.67
1.56
1.54
1.53
1.51
1.50
1.44
1.43
1.40
1.27
1.26
1.24
H_2N
O
N
H
N
CF_3
HN
S
NH
0.84
0.96
1.88
0.84
3.11
4.13
1.92
1.16
1.50
3.09
2.55
1.76
2.34
3.13
2.30
0.90
3.00
f1 (ppm)

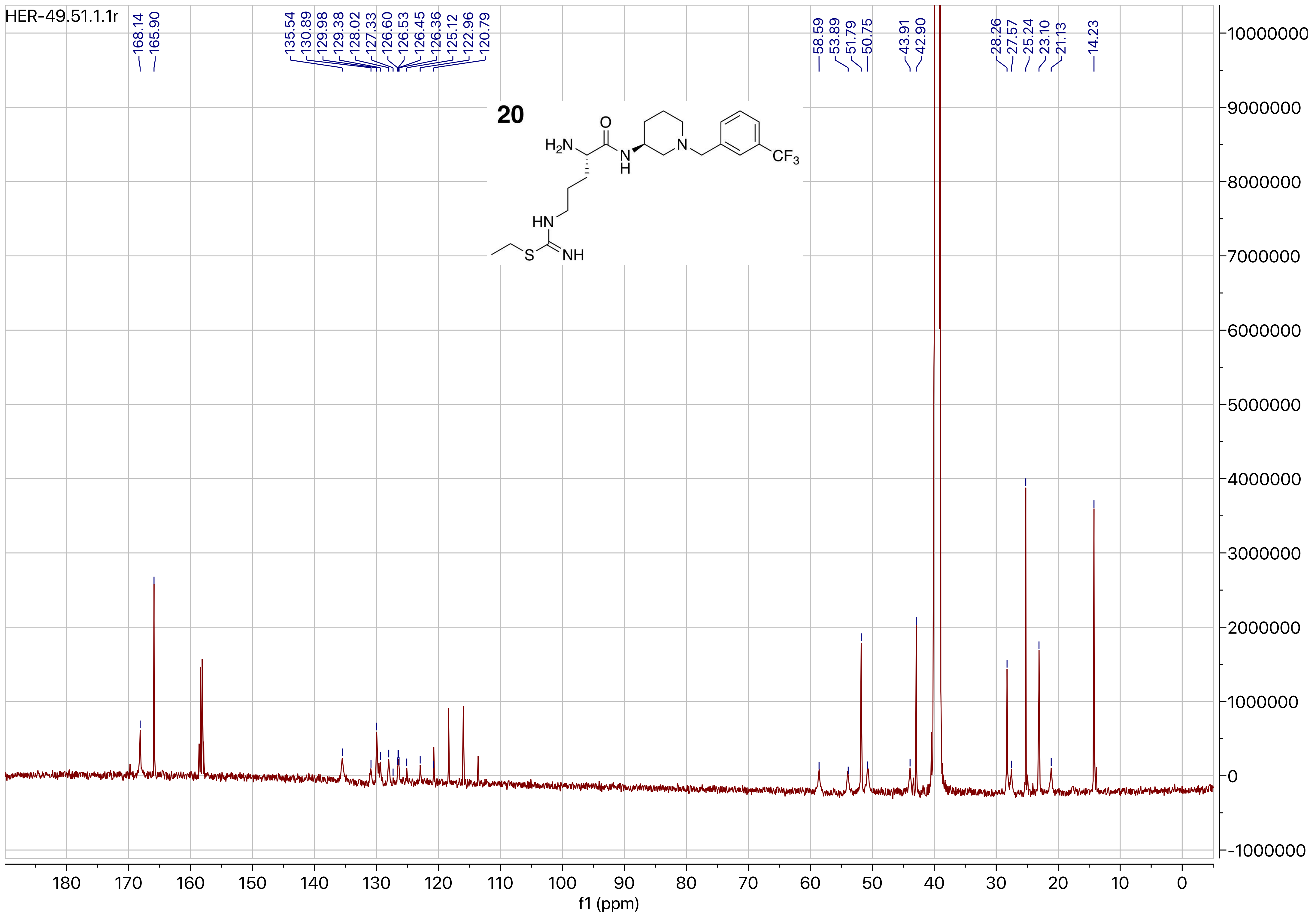

HER-49.51.1.1r
168.14
165.90
135.54
130.89
129.98
129.38
128.02
127.33
126.60
126.53
126.45
126.36
125.12
122.96
120.79
58.59
53.89
51.79
50.75
43.91
42.90
28.26
27.57
25.24
23.10
21.13
14.23
20
H_2N
O
N
H
N
CF_3
HN
S
NH
f1 (ppm)

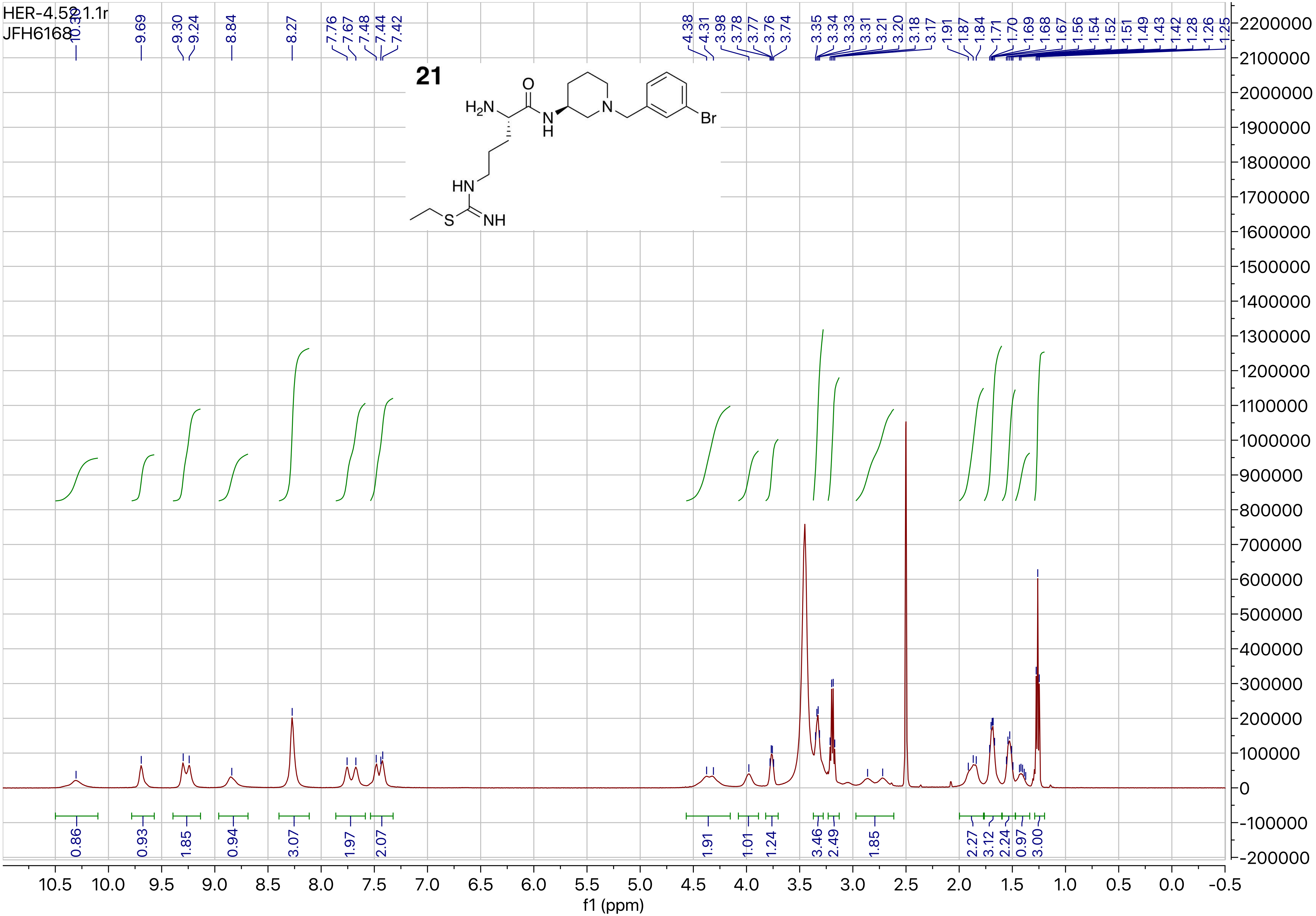
HER-4.521.1r
JFH6168
21
H2N
O
N
H
N
Br
HN
S
NH
10.30
9.69
9.30
9.24
8.84
8.27
7.76
7.67
7.48
7.44
7.42
4.38
4.31
3.98
3.78
3.77
3.76
3.74
3.35
3.34
3.33
3.31
3.21
3.20
3.18
3.17
1.91
1.87
1.84
1.71
1.70
1.69
1.68
1.67
1.56
1.54
1.52
1.51
1.49
1.43
1.42
1.28
1.26
1.25
0.86
0.93
1.85
0.94
3.07
1.97
2.07
1.91
1.01
1.24
3.46
2.49
1.85
2.27
3.12
2.24
0.97
3.00
f1 (ppm)

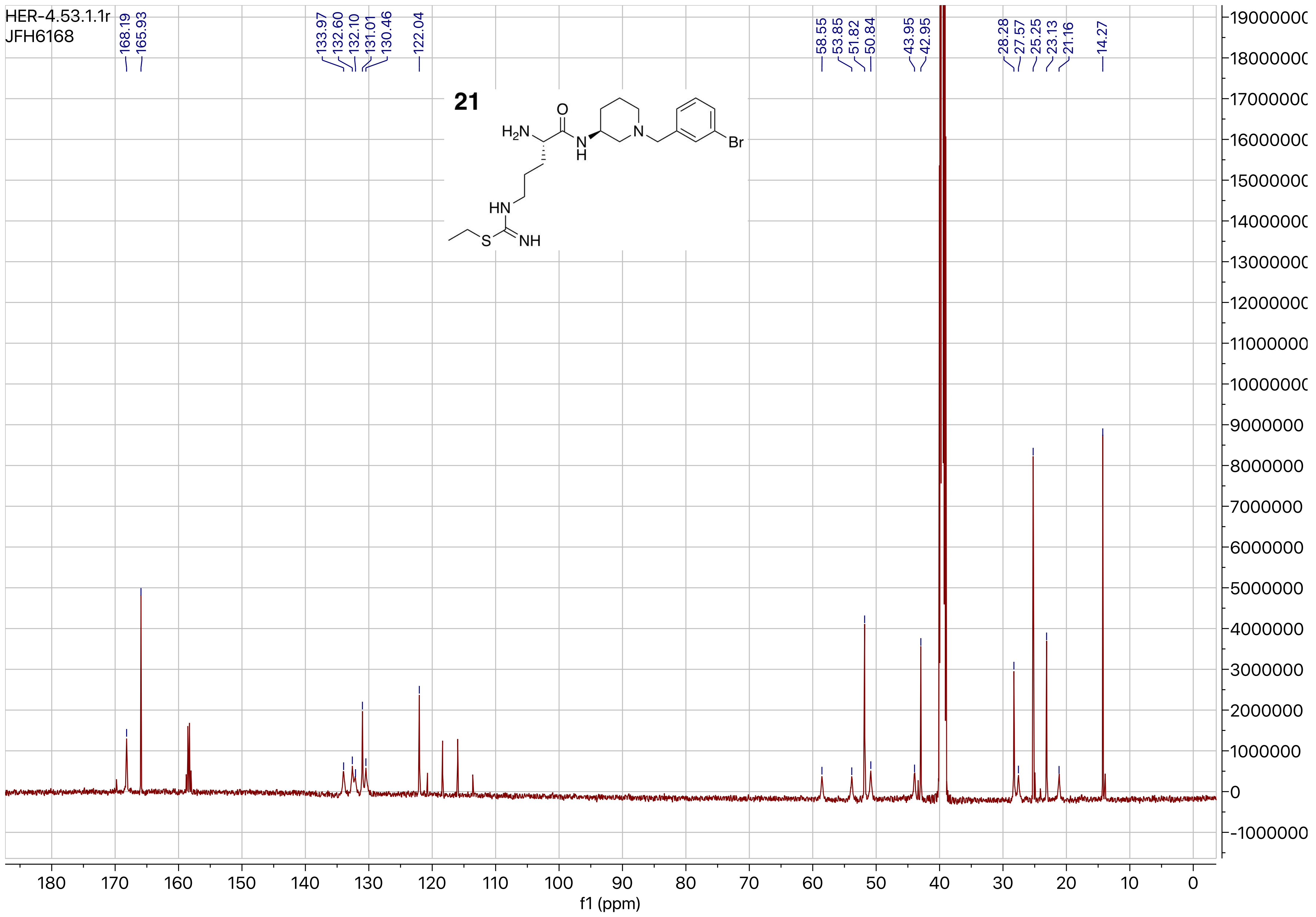
HER-4.53.1.1r
JFH6168
168.19
165.93
133.97
132.60
132.10
131.01
130.46
122.04
58.55
53.85
51.82
50.84
43.95
42.95
28.28
27.57
25.25
23.13
21.16
14.27
21
O
H2N
N
H
N
Br
HN
S
NH
f1 (ppm)

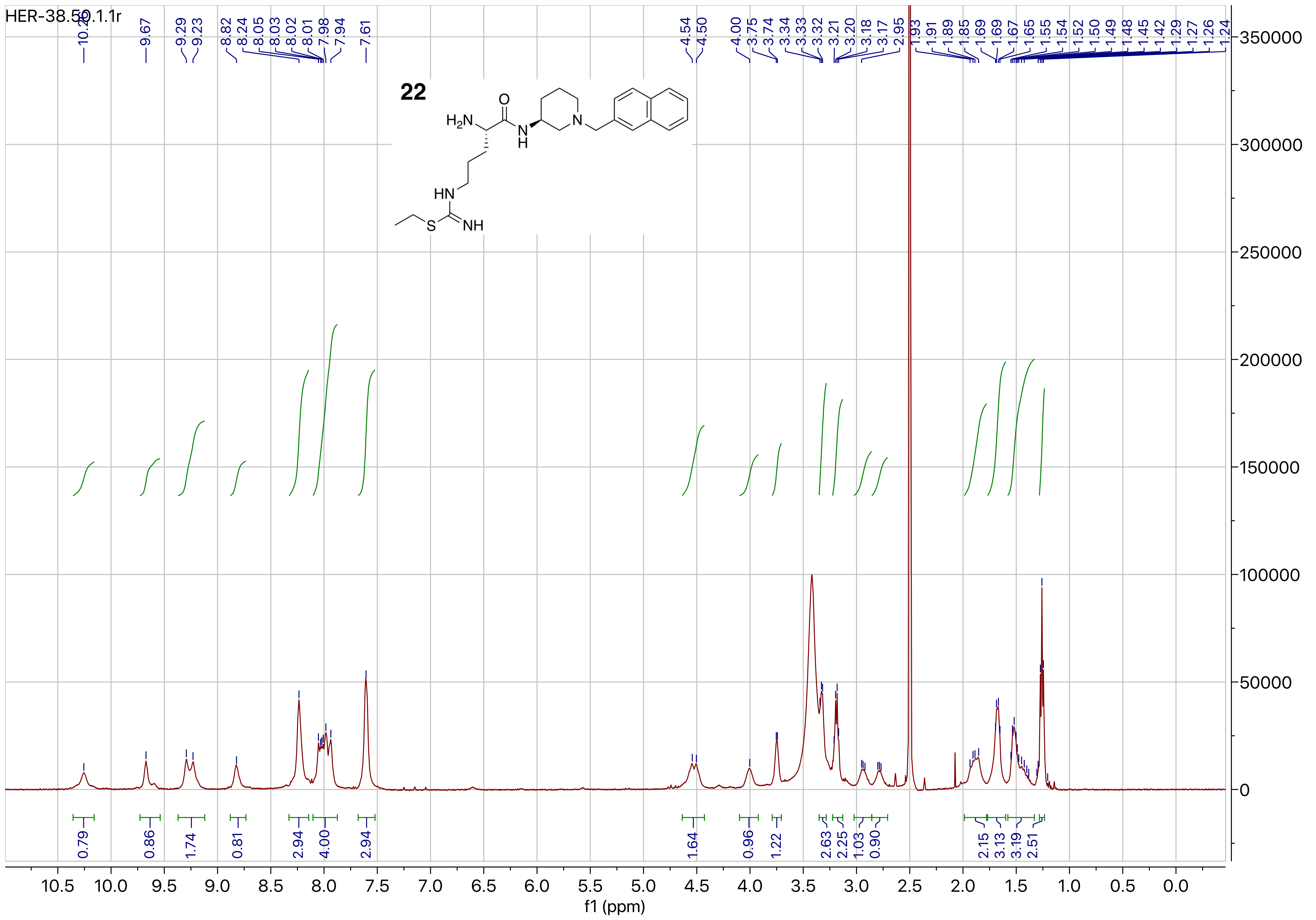
HER-38.50.1.1r
10.2[illegible]
9.67
9.29
9.23
8.82
8.24
8.05
8.03
8.02
8.01
7.98
7.94
7.61
4.54
4.50
4.00
3.75
3.74
3.34
3.33
3.32
3.21
3.20
3.18
3.17
2.95
1.93
1.91
1.89
1.85
1.69
1.69
1.67
1.65
1.55
1.54
1.52
1.50
1.49
1.48
1.45
1.42
1.29
1.27
1.26
1.24
22
O
H_2N
N
H
N
HN
S
NH
0.79
0.86
1.74
0.81
2.94
4.00
2.94
1.64
0.96
1.22
2.63
2.25
1.03
0.90
2.15
3.13
3.19
2.51
350000
300000
250000
200000
150000
100000
50000
0
10.5 10.0 9.5 9.0 8.5 8.0 7.5 7.0 6.5 6.0 5.5 5.0 4.5 4.0 3.5 3.0 2.5 2.0 1.5 1.0 0.5 0.0
f1 (ppm)

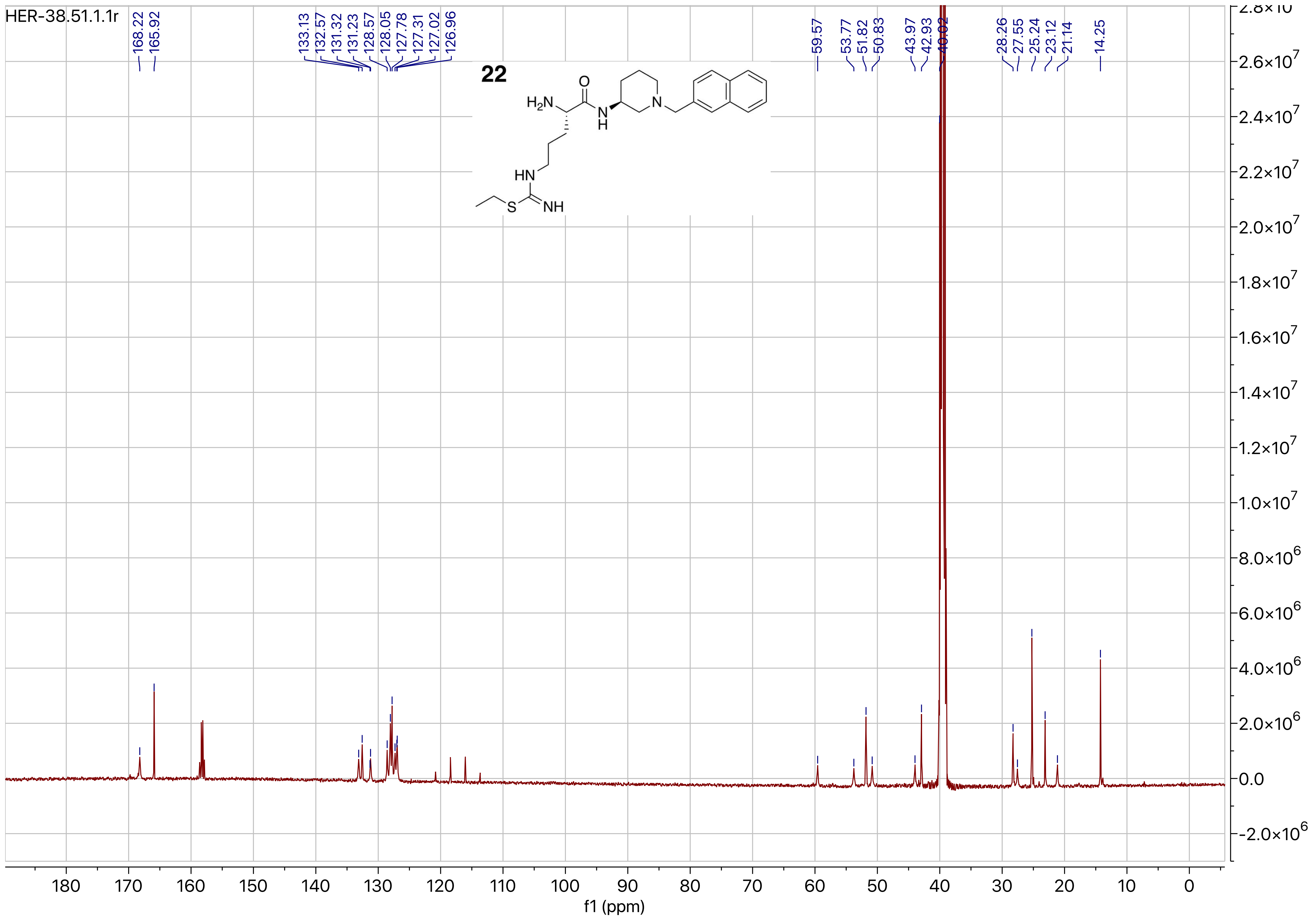
HER-38.51.1.1r
168.22
165.92
133.13
132.57
131.32
131.23
128.57
128.05
127.78
127.31
127.02
126.96
59.57
53.77
51.82
50.83
43.97
42.93
40.02
28.26
27.55
25.24
23.12
21.14
14.25
22
H2N
O
N
H
N
HN
S
NH
f1 (ppm)

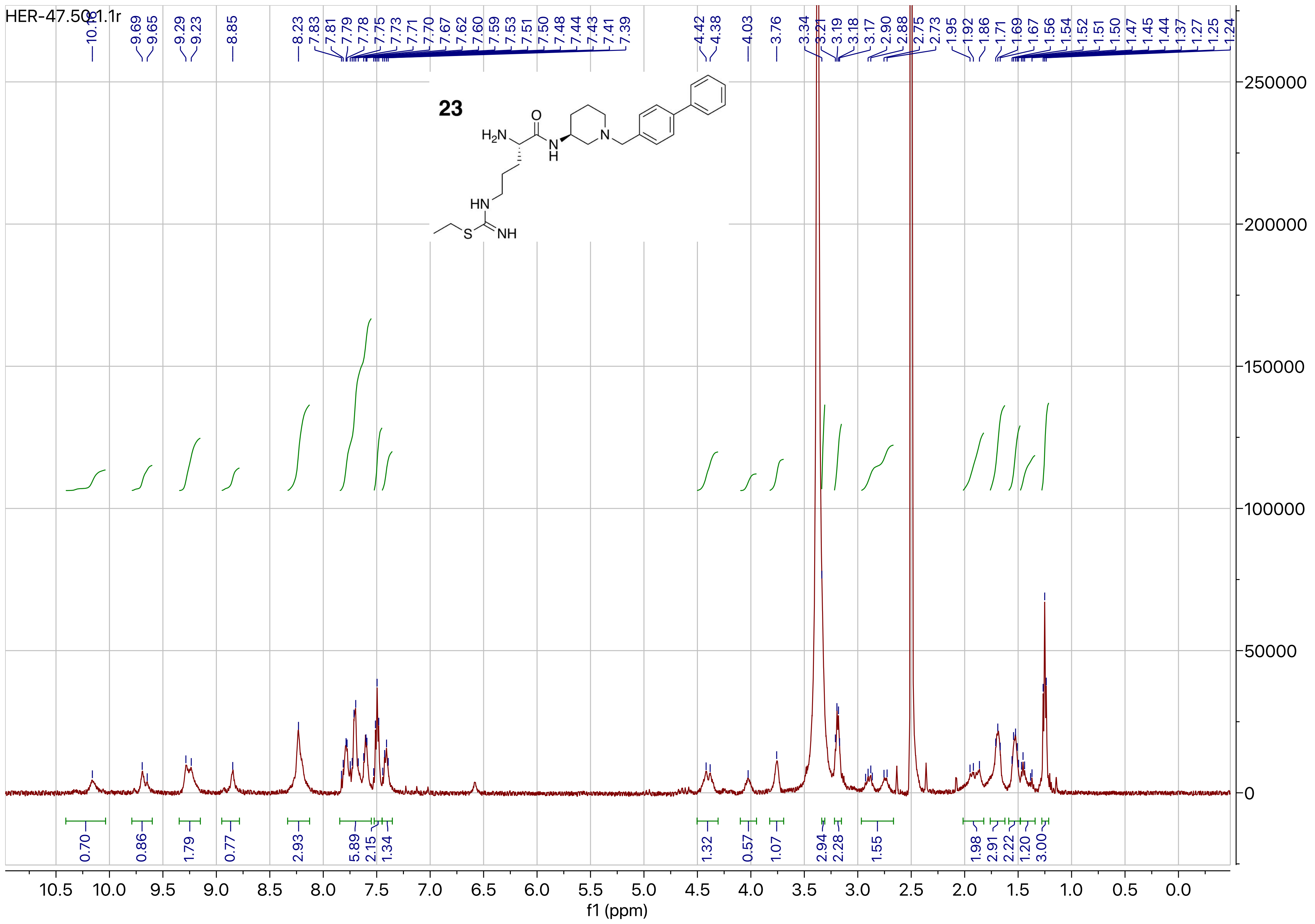

23
H2N
O
N
H
N
HN
S
NH
9.69
9.65
9.29
9.23
8.85
8.23
7.83
7.81
7.79
7.78
7.75
7.73
7.71
7.70
7.67
7.62
7.60
7.59
7.53
7.51
7.50
7.48
7.44
7.43
7.41
7.39
4.42
4.38
4.03
3.76
3.34
3.21
3.19
3.18
3.17
2.90
2.88
2.75
2.73
1.95
1.92
1.86
1.71
1.69
1.67
1.56
1.54
1.52
1.51
1.50
1.47
1.45
1.44
1.37
1.27
1.25
1.24
0.70
0.86
1.79
0.77
2.93
5.89
2.15
1.34
1.32
0.57
1.07
2.94
2.28
1.55
1.98
2.91
2.22
1.20
3.00
250000
200000
150000
100000
50000
0
10.5
10.0
9.5
9.0
8.5
8.0
7.5
7.0
6.5
6.0
5.5
5.0
4.5
4.0
3.5
3.0
2.5
2.0
1.5
1.0
0.5
0.0
f1 (ppm)

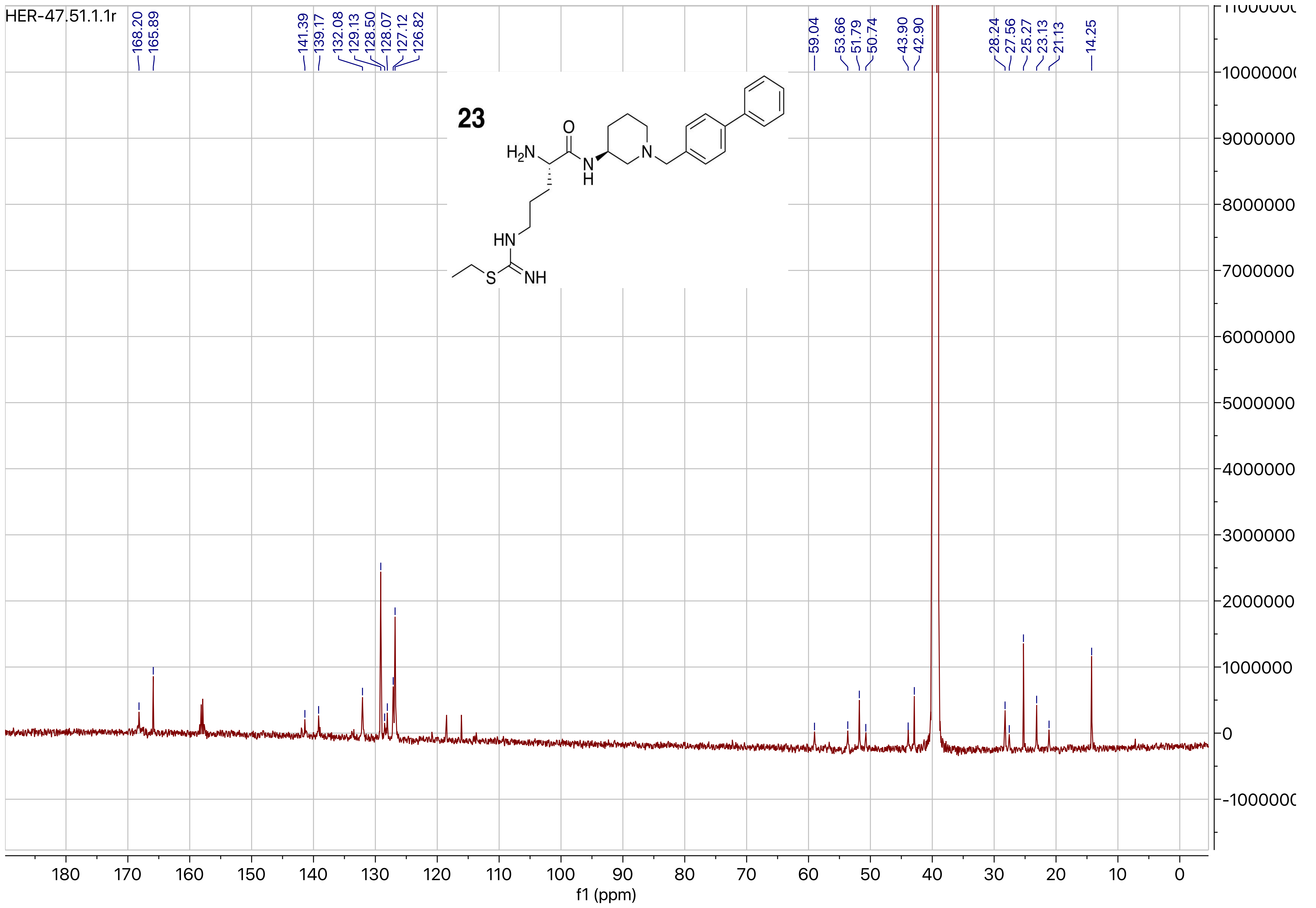
HER-47.51.1.1r
168.20
165.89
141.39
139.17
132.08
129.13
128.50
128.07
127.12
126.82
59.04
53.66
51.79
50.74
43.90
42.90
28.24
27.56
25.27
23.13
21.13
14.25
23
O
H_2N
N
H
N
HN
S
NH
10000000
9000000
8000000
7000000
6000000
5000000
4000000
3000000
2000000
1000000
0
180
170
160
150
140
130
120
110
100
90
80
70
60
50
40
30
20
10
0
f1 (ppm)

HER-48.50.1.1r

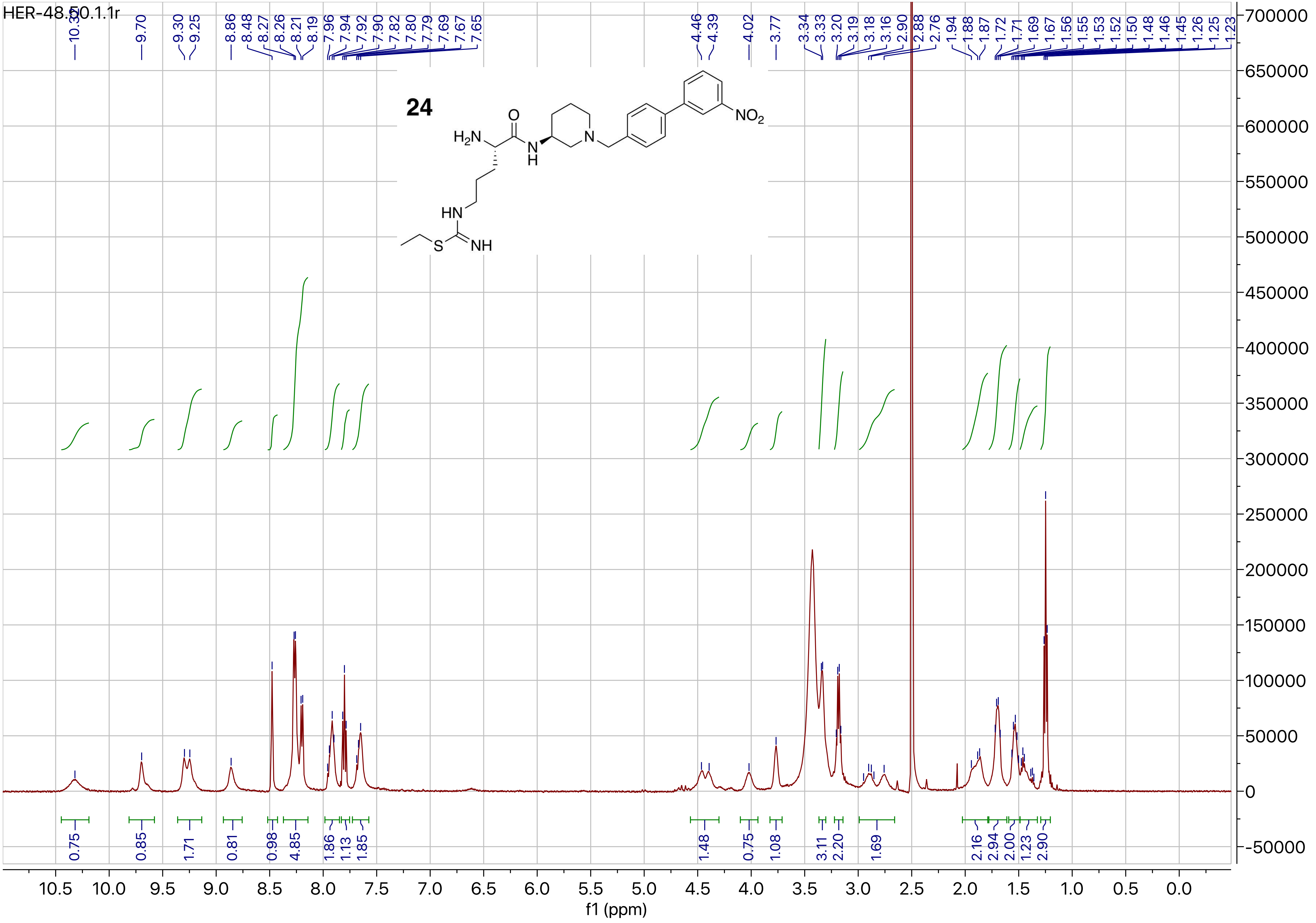

24
H_2N
O
N
H
N
NO_2
HN
S
NH
10.30
9.70
9.30
9.25
8.86
8.48
8.27
8.26
8.21
8.19
7.96
7.94
7.92
7.90
7.82
7.80
7.79
7.69
7.67
7.65
4.46
4.39
4.02
3.77
3.34
3.33
3.20
3.19
3.18
3.16
2.90
2.88
2.76
1.94
1.88
1.87
1.72
1.71
1.69
1.67
1.56
1.55
1.53
1.52
1.50
1.48
1.46
1.45
1.26
1.25
1.23
0.75
0.85
1.71
0.81
0.98
4.85
1.86
1.13
1.85
1.48
0.75
1.08
3.11
2.20
1.69
2.16
2.94
2.00
1.23
2.90
f1 (ppm)

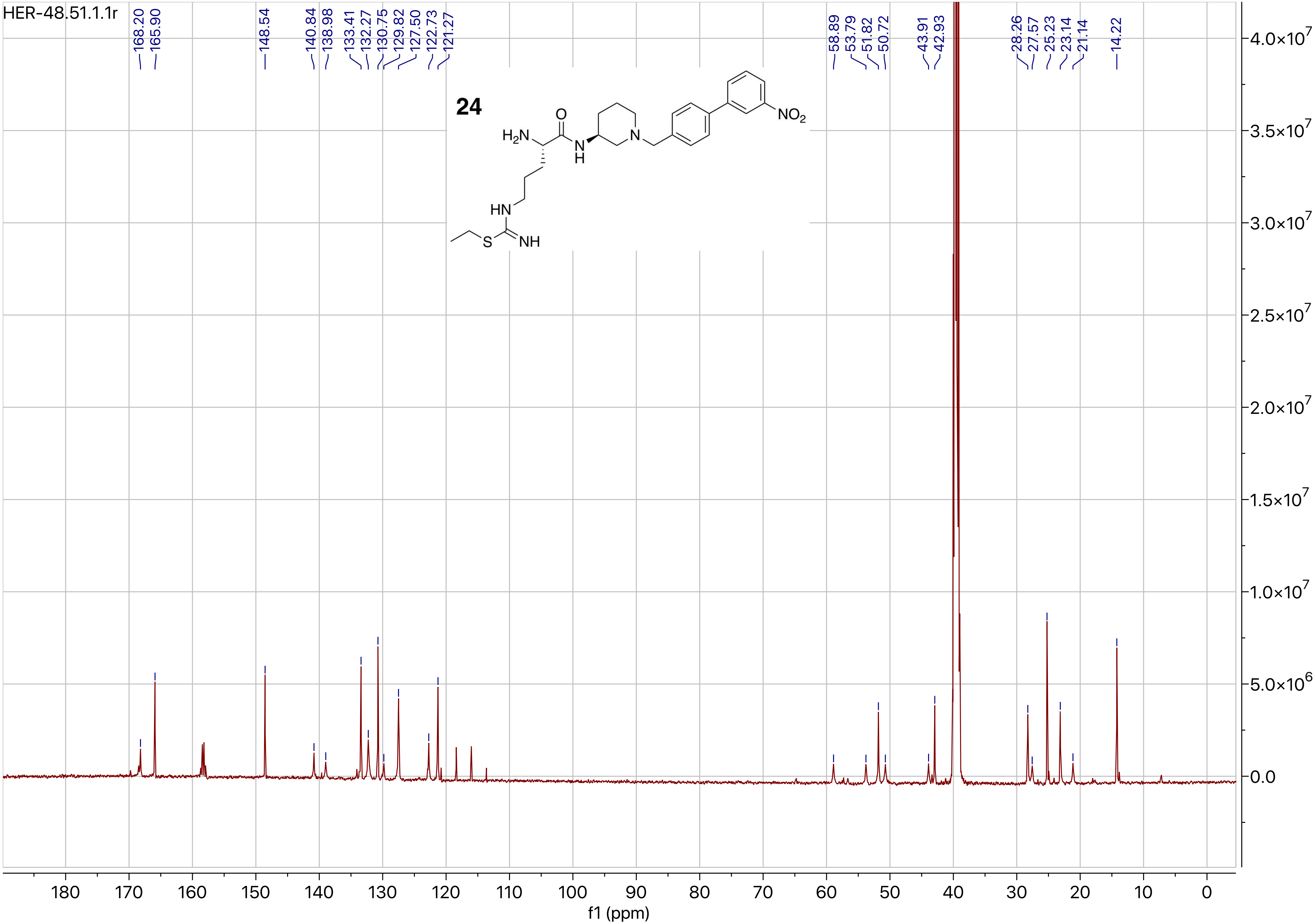

HER-48.51.1.1r
168.20
165.90
148.54
140.84
138.98
133.41
132.27
130.75
129.82
127.50
122.73
121.27
58.89
53.79
51.82
50.72
43.91
42.93
28.26
27.57
25.23
23.14
21.14
14.22
24
H_2N
O
N
H
N
NO_2
HN
S
NH
f1 (ppm)

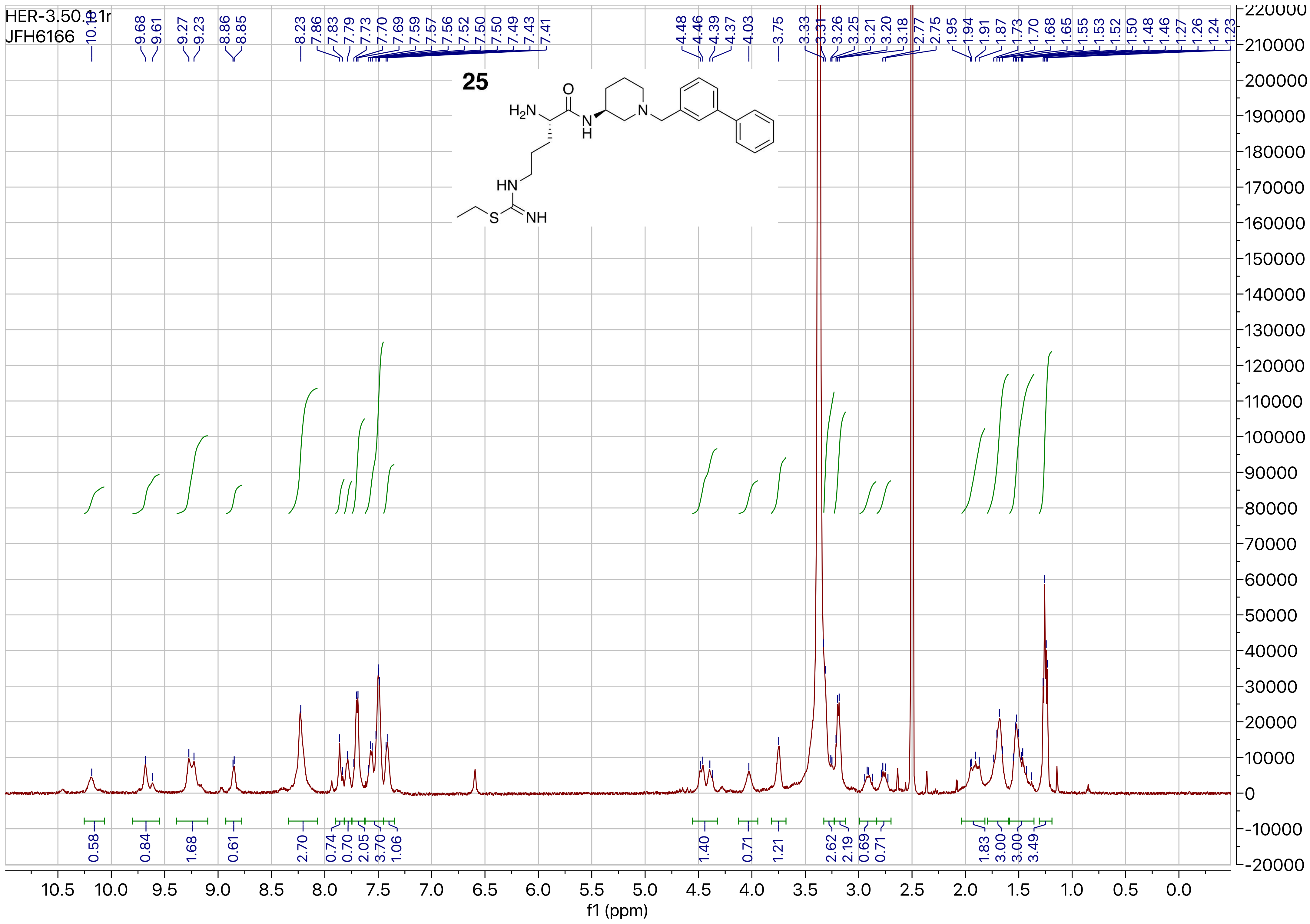
JFH6166
25
H2N
O
N
H
N
HN
S
NH
9.68 9.61 9.27 9.23 8.86 8.85 8.23 7.86 7.83 7.79 7.73 7.70 7.69 7.59 7.57 7.56 7.52 7.50 7.50 7.49 7.43 7.41
4.48 4.46 4.39 4.37 4.03 3.75 3.33 3.31 3.26 3.25 3.21 3.20 3.18 2.77 2.75 1.95 1.94 1.91 1.87 1.73 1.70 1.68 1.65 1.55 1.53 1.52 1.50 1.48 1.46 1.27 1.26 1.24 1.23
0.58 0.84 1.68 0.61 2.70 0.74 0.70 2.05 3.70 1.06 1.40 0.71 1.21 2.62 2.19 0.69 0.71 1.83 3.00 3.00 3.49
f1 (ppm)

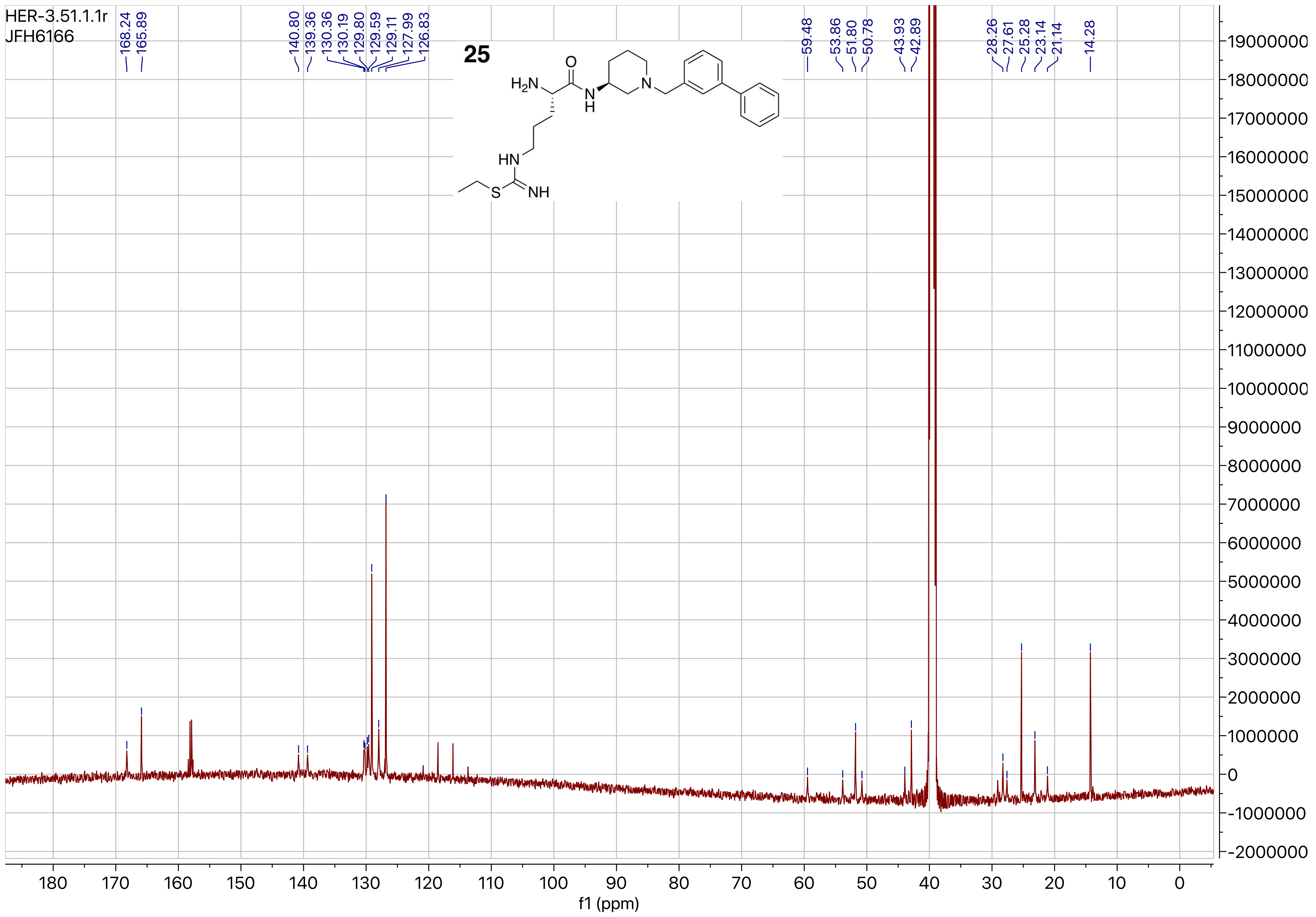

HER-3.51.1.1r
JFH6166
25
168.24
165.89
140.80
139.36
130.36
130.19
129.80
129.59
129.11
127.99
126.83
59.48
53.86
51.80
50.78
43.93
42.89
28.26
27.61
25.28
23.14
21.14
14.28
f1 (ppm)

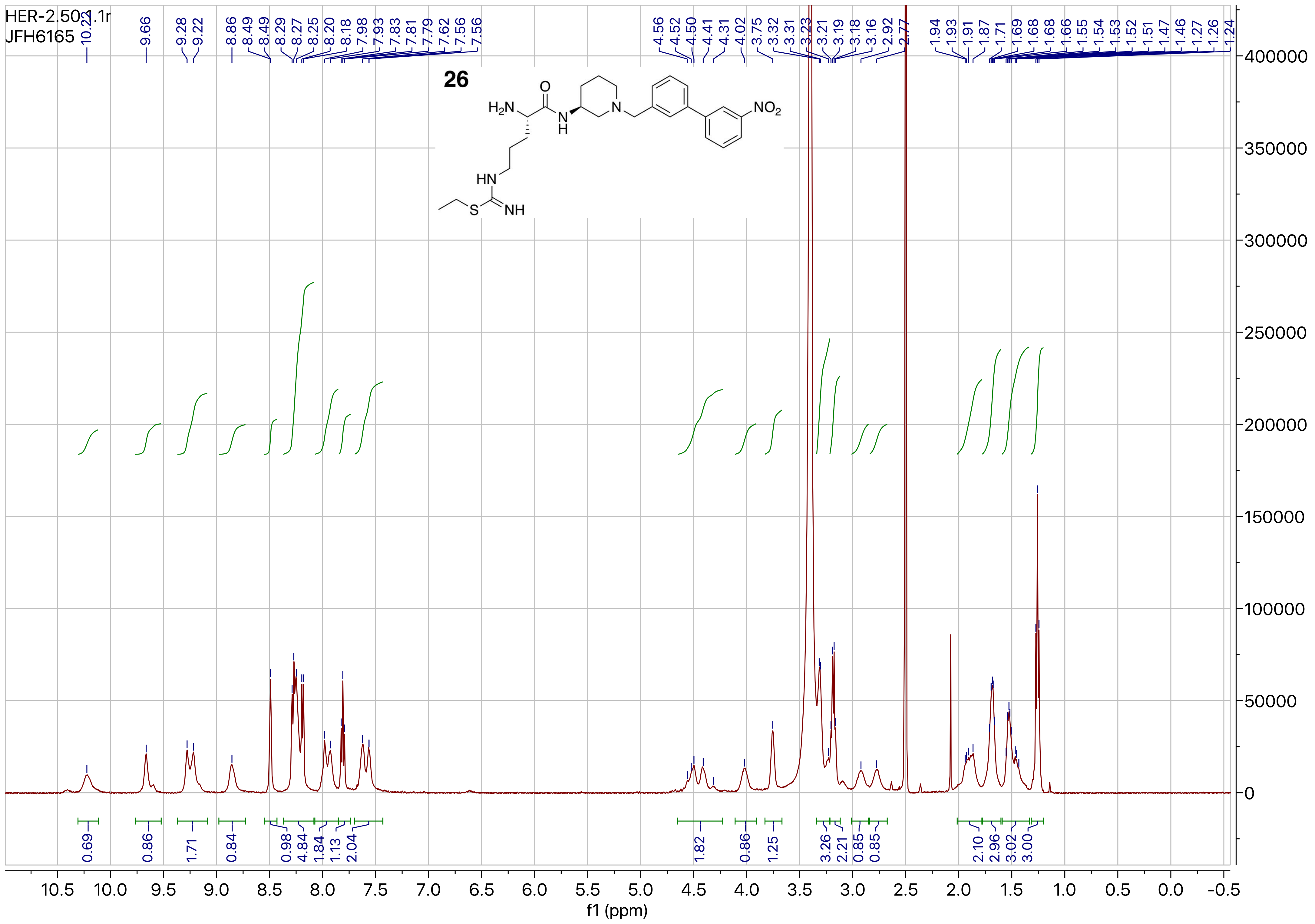
JFH6165
26
H2N
O
N
H
N
NO2
HN
S
NH
10.22
9.66
9.28
9.22
8.86
8.49
8.49
8.29
8.27
8.25
8.20
8.18
7.98
7.93
7.83
7.81
7.79
7.62
7.56
7.56
4.56
4.52
4.50
4.41
4.31
4.02
3.75
3.32
3.31
3.23
3.21
3.19
3.18
3.16
2.92
2.77
1.94
1.93
1.91
1.87
1.71
1.69
1.68
1.68
1.66
1.55
1.54
1.53
1.52
1.51
1.47
1.46
1.27
1.26
1.24
0.69
0.86
1.71
0.84
0.98
4.84
1.84
1.13
2.04
1.82
0.86
1.25
3.26
2.21
0.85
0.85
2.10
2.96
3.02
3.00
400000
350000
300000
250000
200000
150000
100000
50000
0
10.5 10.0 9.5 9.0 8.5 8.0 7.5 7.0 6.5 6.0 5.5 5.0 4.5 4.0 3.5 3.0 2.5 2.0 1.5 1.0 0.5 0.0 -0.5
f1 (ppm)

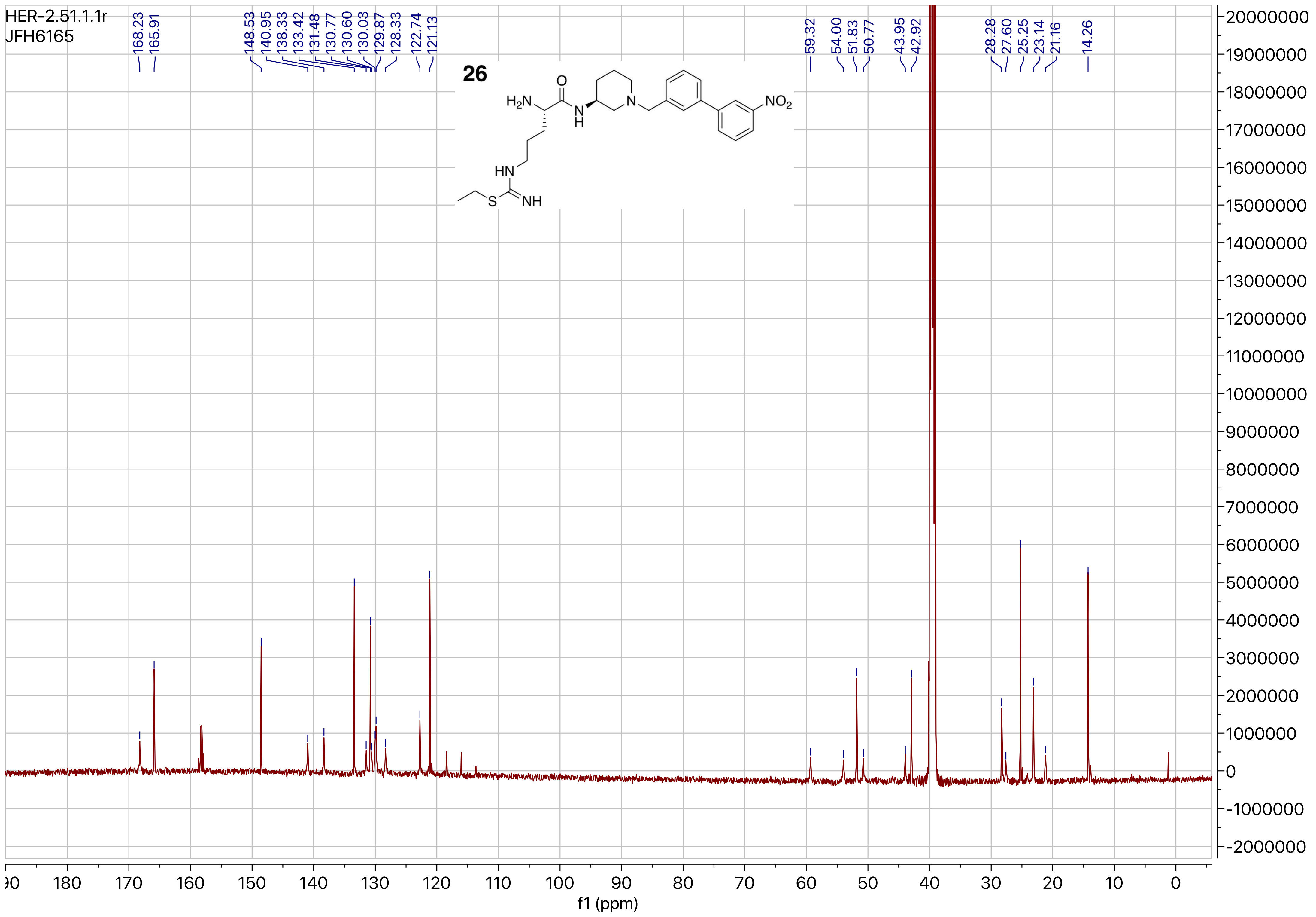

HER-2.51.1.1r
JFH6165
168.23
165.91
148.53
140.95
138.33
133.42
131.48
130.77
130.60
130.03
129.87
128.33
122.74
121.13
59.32
54.00
51.83
50.77
43.95
42.92
28.28
27.60
25.25
23.14
21.16
14.26
26
H_2N
O
N
H
N
NO_2
HN
S
NH
f1 (ppm)

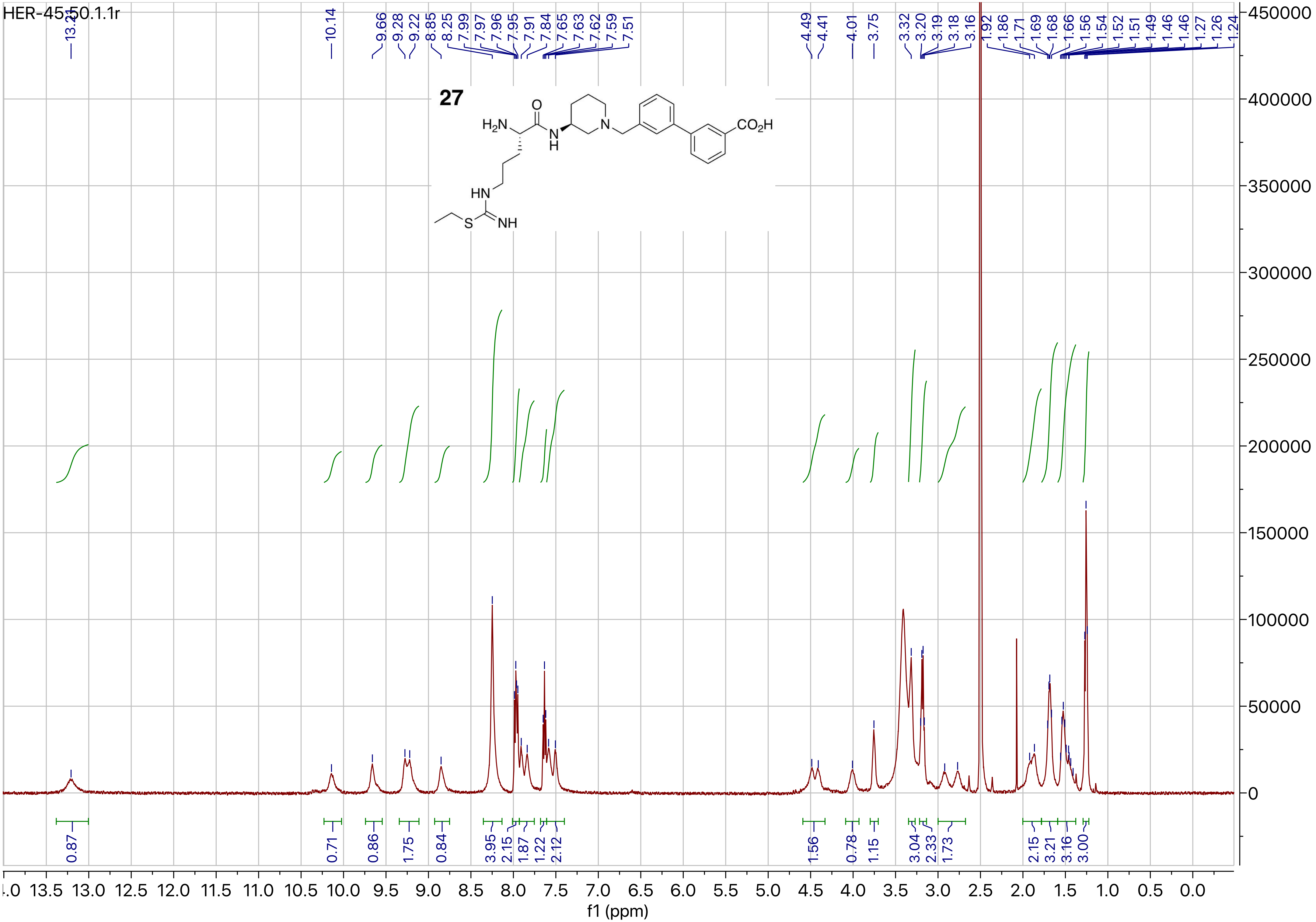
HER-45-50.1.1r
13.21
10.14
9.66
9.28
9.22
8.85
8.25
7.99
7.97
7.96
7.95
7.91
7.84
7.65
7.63
7.62
7.59
7.51
4.49
4.41
4.01
3.75
3.32
3.20
3.19
3.18
3.16
1.92
1.86
1.71
1.69
1.68
1.66
1.56
1.54
1.52
1.51
1.49
1.46
1.46
1.27
1.26
1.24
27
O
H2N
N
H
N
CO2H
HN
S
NH
0.87
0.71
0.86
1.75
0.84
3.95
2.15
1.87
1.22
2.12
1.56
0.78
1.15
3.04
2.33
1.73
2.15
3.21
3.16
3.00
450000
400000
350000
300000
250000
200000
150000
100000
50000
0
13.5 13.0 12.5 12.0 11.5 11.0 10.5 10.0 9.5 9.0 8.5 8.0 7.5 7.0 6.5 6.0 5.5 5.0 4.5 4.0 3.5 3.0 2.5 2.0 1.5 1.0 0.5 0.0
f1 (ppm)

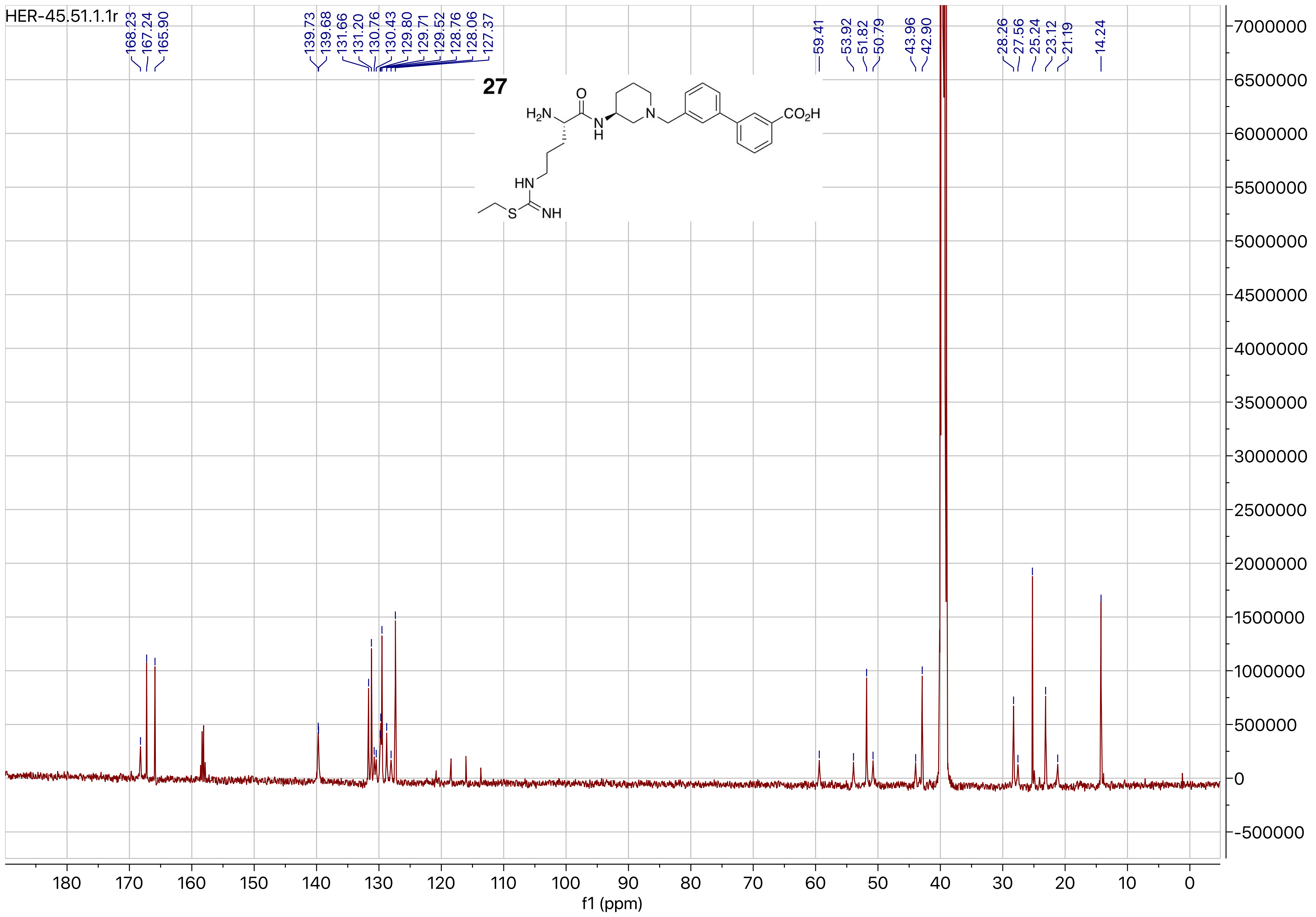
HER-45.51.1.1r
168.23
167.24
165.90
139.73
139.68
131.66
131.20
130.76
130.43
129.80
129.71
129.52
128.76
128.06
127.37
59.41
53.92
51.82
50.79
43.96
42.90
28.26
27.56
25.24
23.12
21.19
14.24
27
H2N
O
N
H
N
CO2H
HN
S
NH
7000000
6500000
6000000
5500000
5000000
4500000
4000000
3500000
3000000
2500000
2000000
1500000
1000000
500000
0
-500000
180
170
160
150
140
130
120
110
100
90
80
70
60
50
40
30
20
10
0
f1 (ppm)

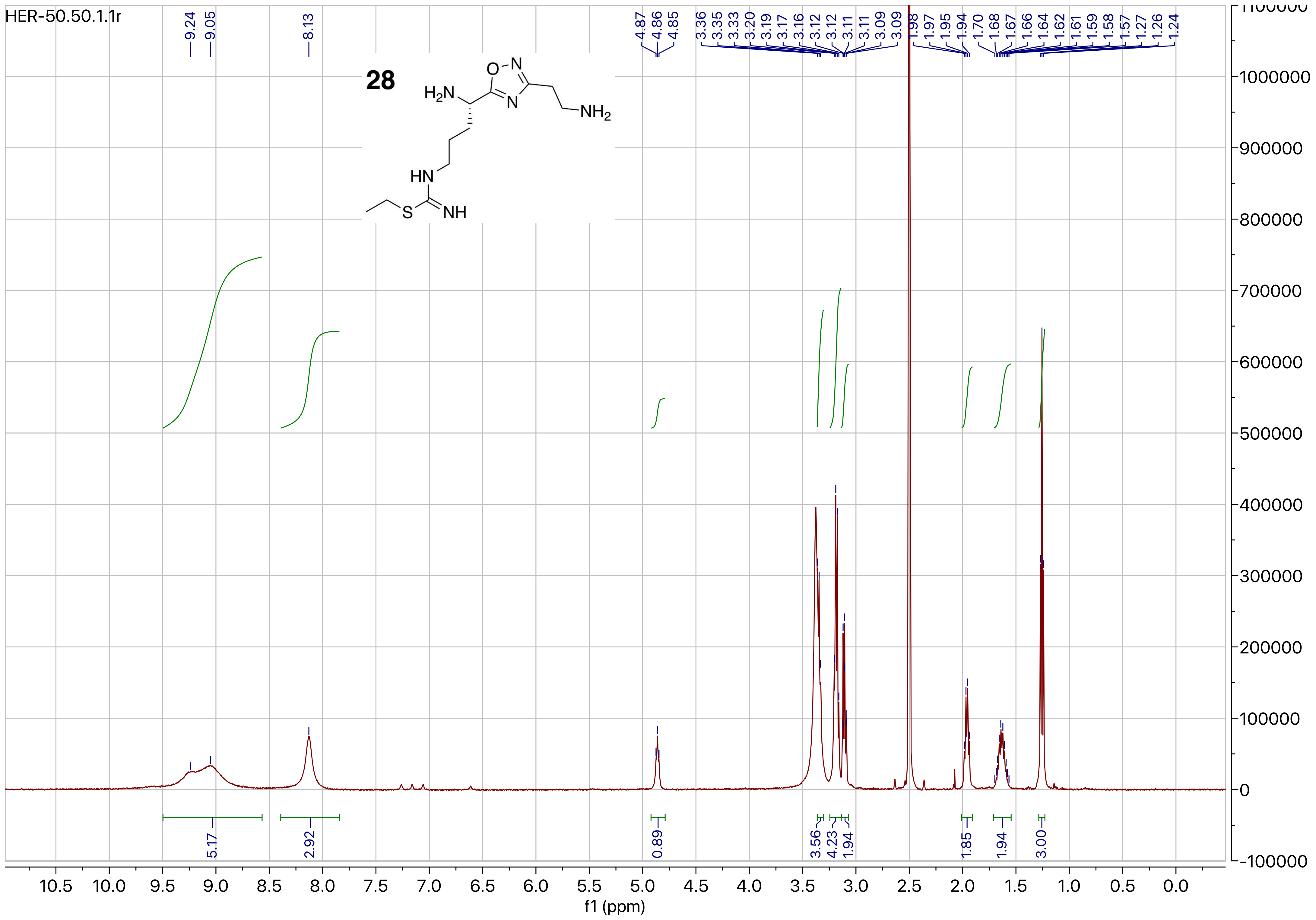

HER-50.50.1.1r
28
H2N
NH2
HN
S
NH
O
N
N
9.24
9.05
8.13
4.87
4.86
4.85
3.36
3.35
3.33
3.20
3.19
3.17
3.16
3.12
3.12
3.11
3.11
3.09
3.09
1.98
1.97
1.95
1.94
1.70
1.68
1.67
1.66
1.64
1.62
1.61
1.59
1.58
1.57
1.27
1.26
1.24
5.17
2.92
0.89
3.56
4.23
1.94
1.85
1.94
3.00
f1 (ppm)

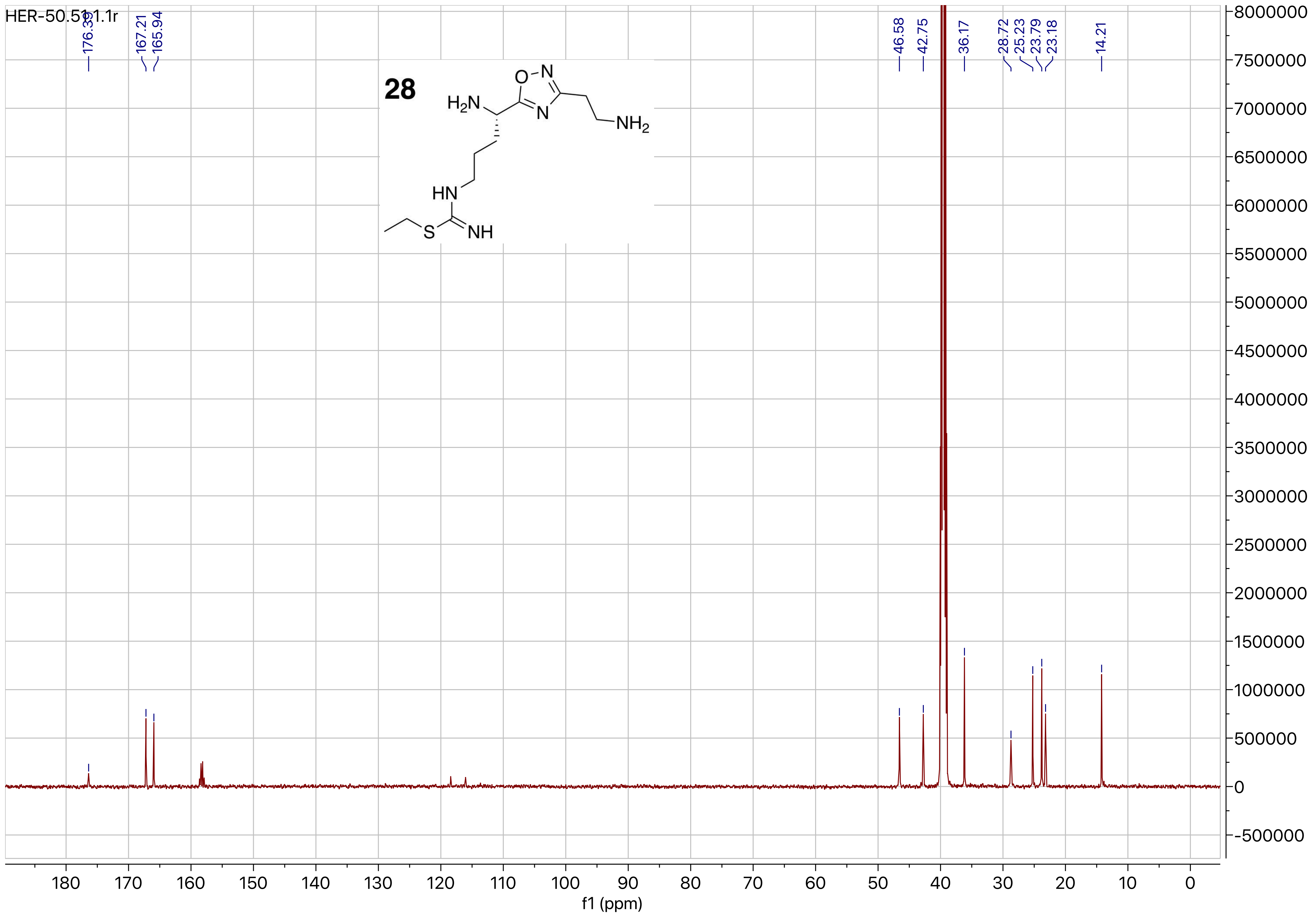
HER-50.511.1r
176.39
167.21
165.94
46.58
42.75
36.17
28.72
25.23
23.79
23.18
14.21
28
H_2N
O
N
N
NH_2
HN
S
NH
f1 (ppm)
180 170 160 150 140 130 120 110 100 90 80 70 60 50 40 30 20 10 0
8000000 7500000 7000000 6500000 6000000 5500000 5000000 4500000 4000000 3500000 3000000 2500000 2000000 1500000 1000000 500000 0 -500000

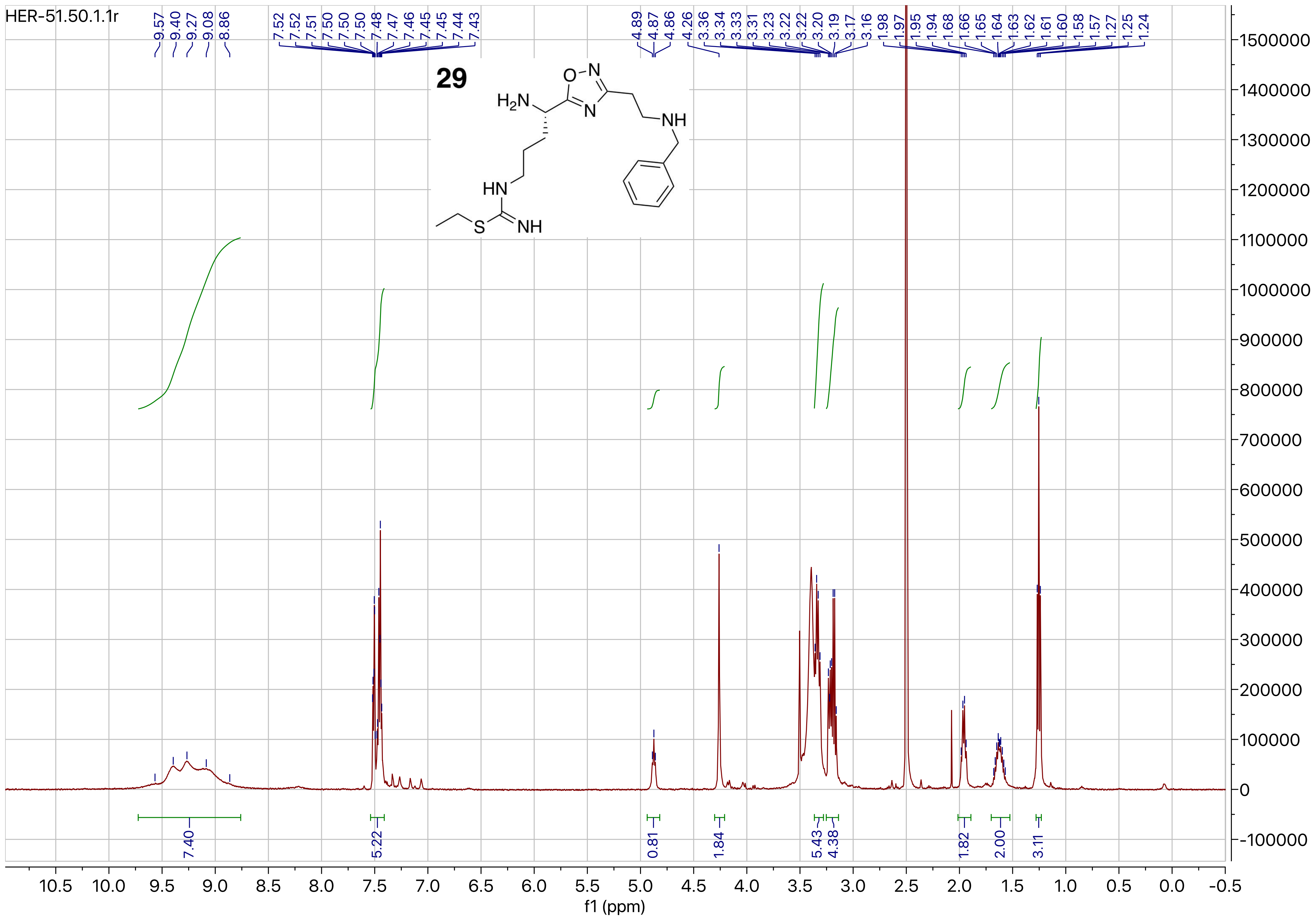

HER-51.50.1.1r
9.57 9.40 9.27 9.08 8.86
7.52 7.52 7.51 7.50 7.50 7.50 7.48 7.47 7.46 7.45 7.45 7.44 7.43
4.89 4.87 4.86 4.26 3.36 3.34 3.33 3.31 3.23 3.22 3.22 3.20 3.19 3.17 3.16 1.98 1.97 1.95 1.94 1.68 1.66 1.65 1.64 1.63 1.62 1.61 1.60 1.58 1.57 1.27 1.25 1.24
29
O
N
N
H2N
NH
HN
S
NH
7.40
5.22
0.81
1.84
5.43
4.38
1.82
2.00
3.11
1500000
1400000
1300000
1200000
1100000
1000000
900000
800000
700000
600000
500000
400000
300000
200000
100000
0
-100000
10.5 10.0 9.5 9.0 8.5 8.0 7.5 7.0 6.5 6.0 5.5 5.0 4.5 4.0 3.5 3.0 2.5 2.0 1.5 1.0 0.5 0.0 -0.5
f1 (ppm)

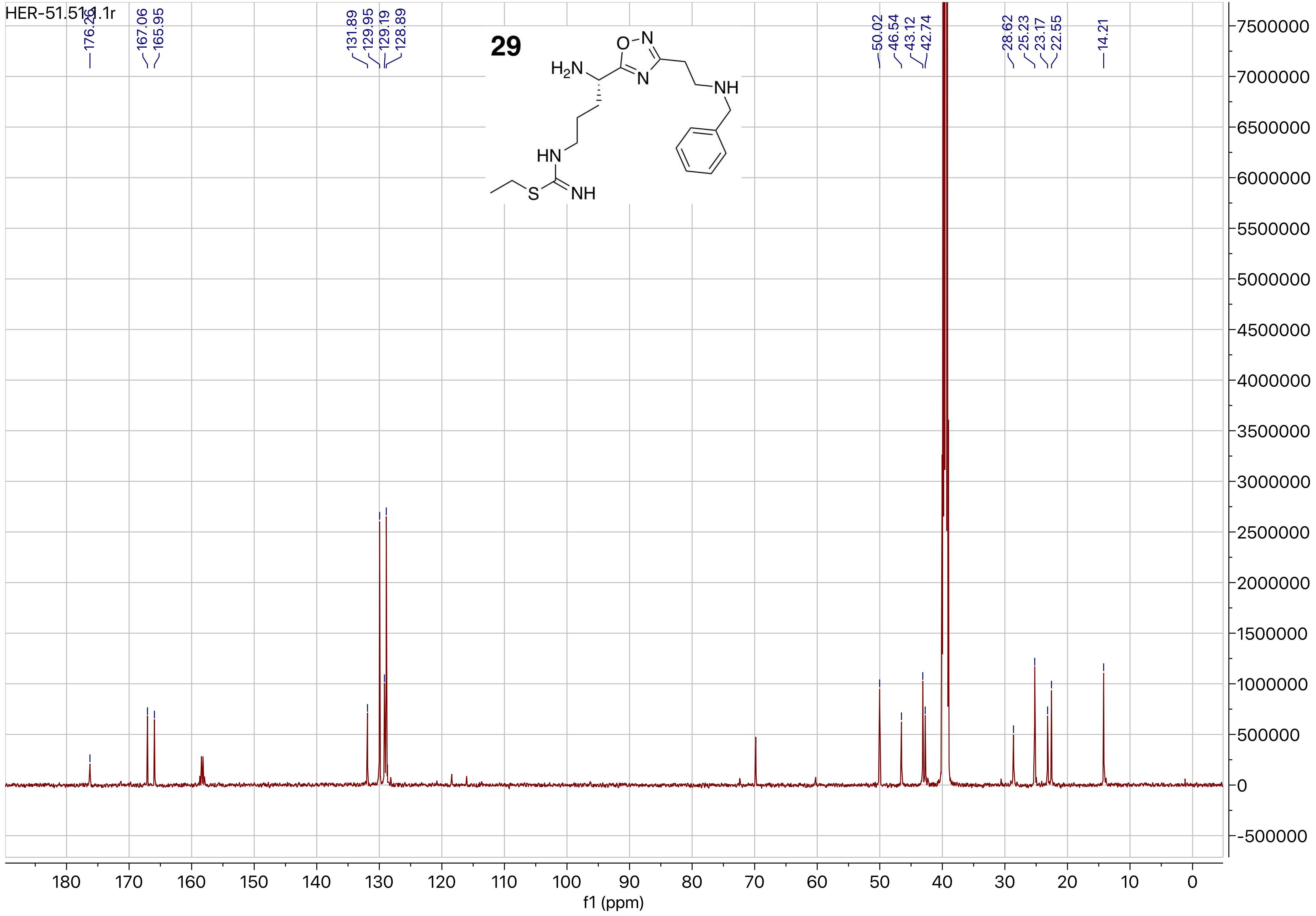

HER-51.511.1r
176.26
167.06
165.95
131.89
129.95
129.19
128.89
50.02
46.54
43.12
42.74
28.62
25.23
23.17
22.55
14.21
29
H2N
O
N
N
NH
HN
S
NH
f1 (ppm)

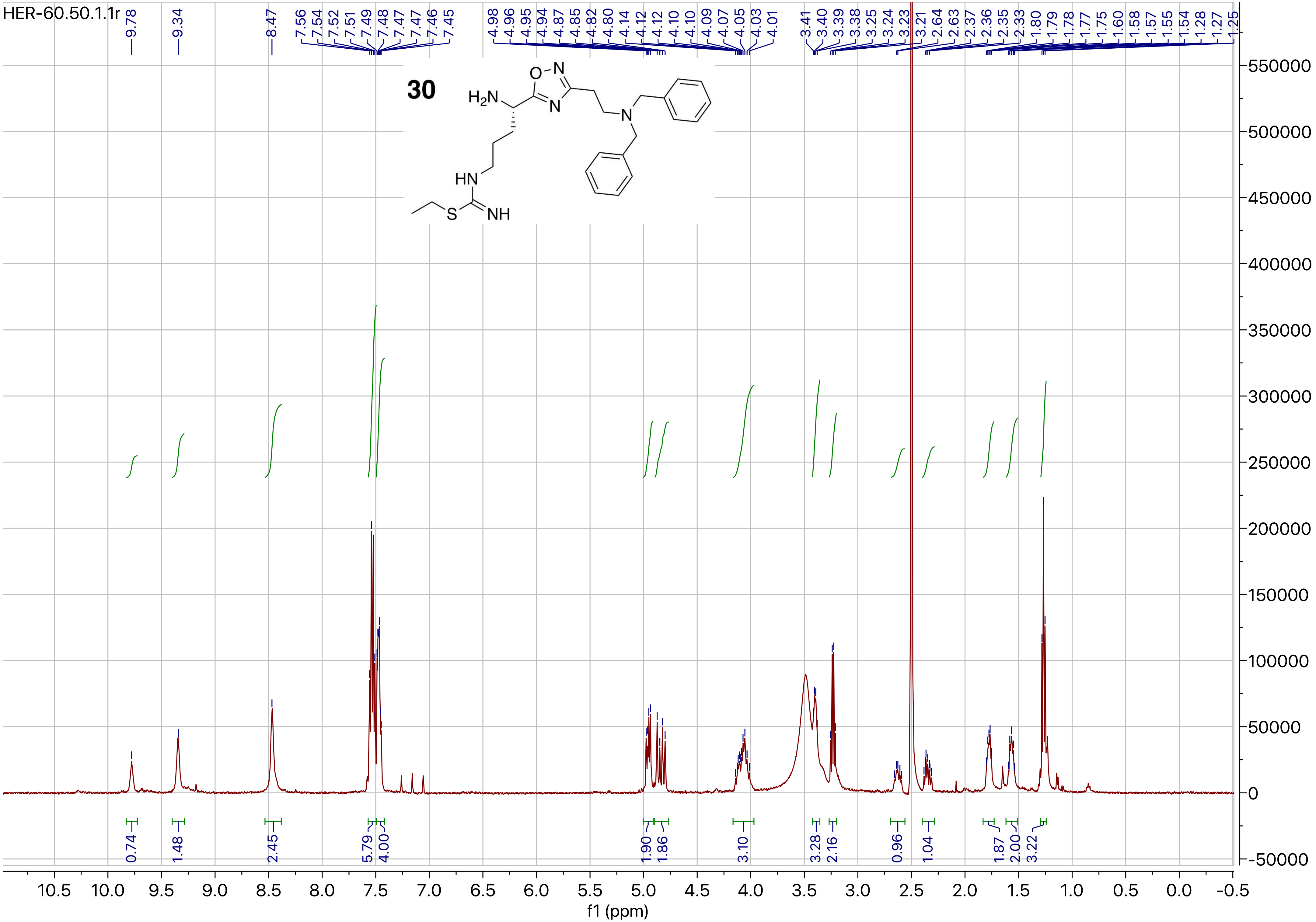

HER-60.50.1.1r
30
f1 (ppm)
9.78
9.34
8.47
7.56 7.54 7.52 7.51 7.49 7.48 7.47 7.47 7.46 7.45
4.98 4.96 4.95 4.94 4.87 4.85 4.82 4.80 4.14 4.12 4.12 4.10 4.10 4.09 4.07 4.05 4.03 4.01
3.41 3.40 3.39 3.38 3.25 3.24 3.23 3.21 2.64 2.63 2.37 2.36 2.35 2.33 1.80 1.79 1.78 1.77 1.75 1.60 1.58 1.57 1.55 1.54 1.28 1.27 1.25
0.74 1.48 2.45 5.79 4.00 1.90 1.86 3.10 3.28 2.16 0.96 1.04 1.87 2.00 3.22

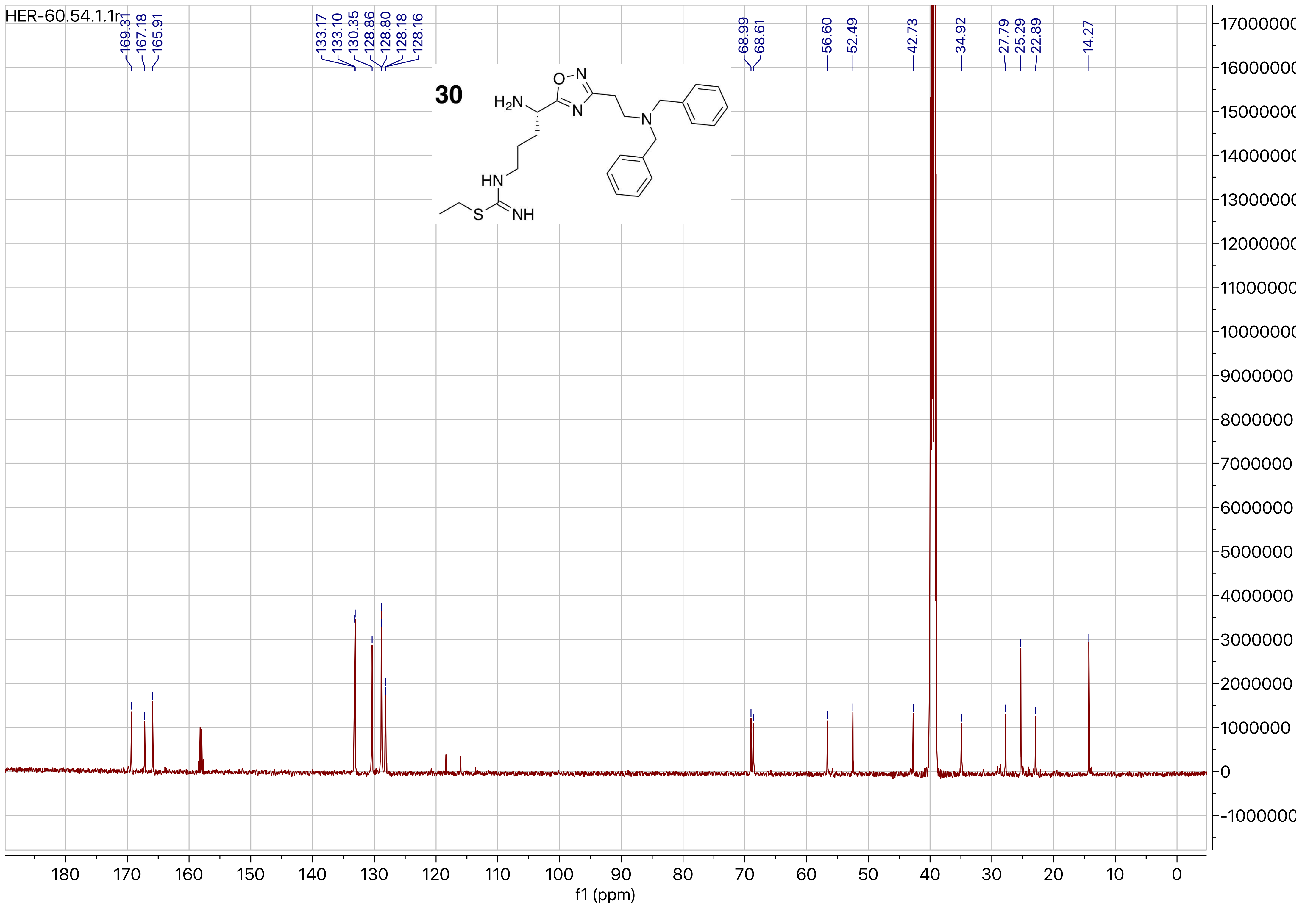
HER-60.54.1.1r
169.31
167.18
165.91
133.17
133.10
130.35
128.86
128.80
128.18
128.16
68.99
68.61
56.60
52.49
42.73
34.92
27.79
25.29
22.89
14.27
30
f1 (ppm)

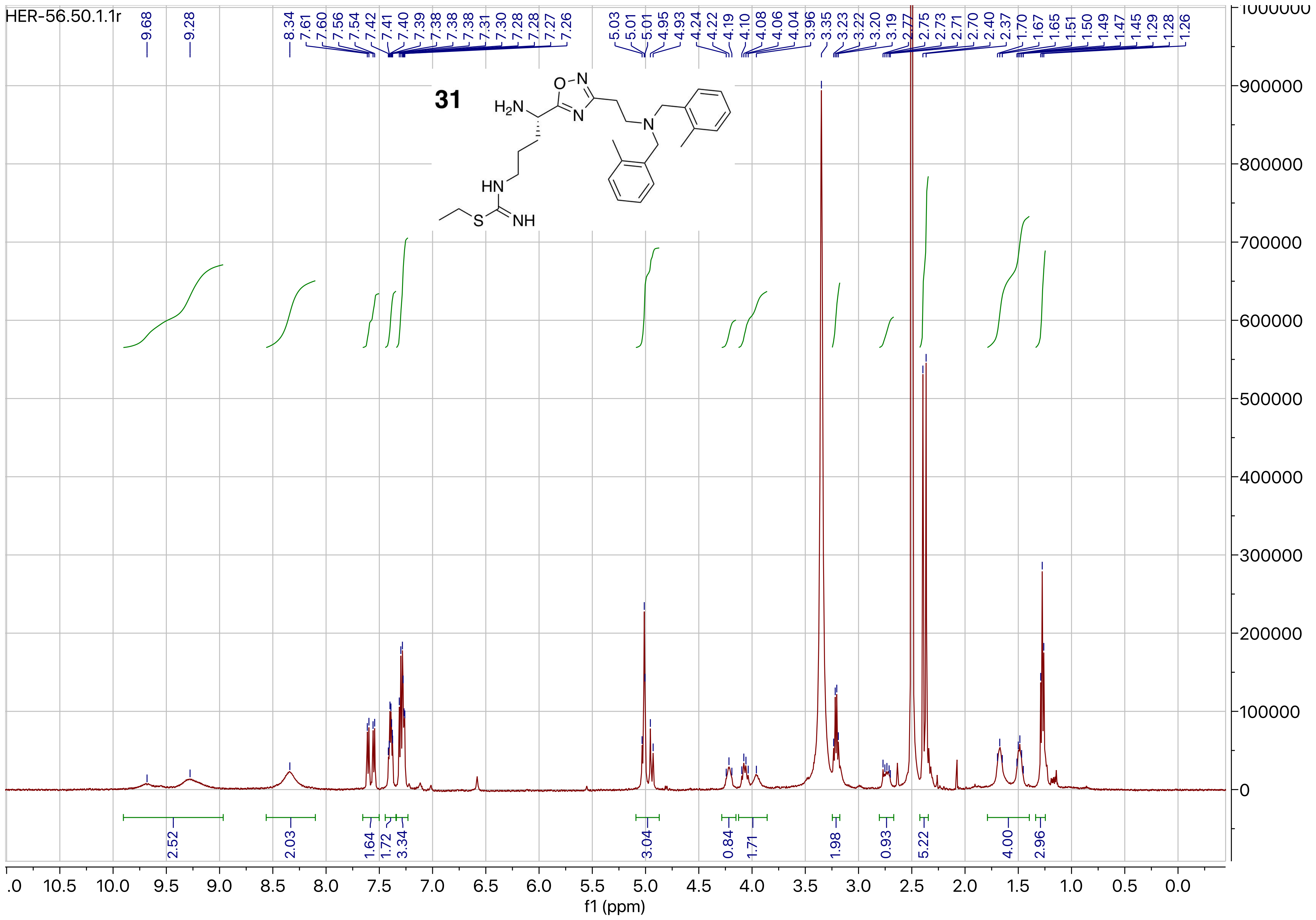
HER-56.50.1.1r
9.68
9.28
8.34
7.61
7.60
7.56
7.54
7.42
7.41
7.40
7.39
7.38
7.38
7.38
7.31
7.30
7.28
7.28
7.27
7.26
5.03
5.01
5.01
4.95
4.93
4.24
4.22
4.19
4.10
4.08
4.06
4.04
3.96
3.35
3.23
3.22
3.20
3.19
2.77
2.75
2.73
2.71
2.70
2.40
2.37
1.70
1.67
1.65
1.51
1.50
1.49
1.47
1.45
1.29
1.28
1.26
31
H_2N
O
N
N
N
HN
S
NH
2.52
2.03
1.64
1.72
3.34
3.04
0.84
1.71
1.98
0.93
5.22
4.00
2.96
1000000
900000
800000
700000
600000
500000
400000
300000
200000
100000
0
f1 (ppm)

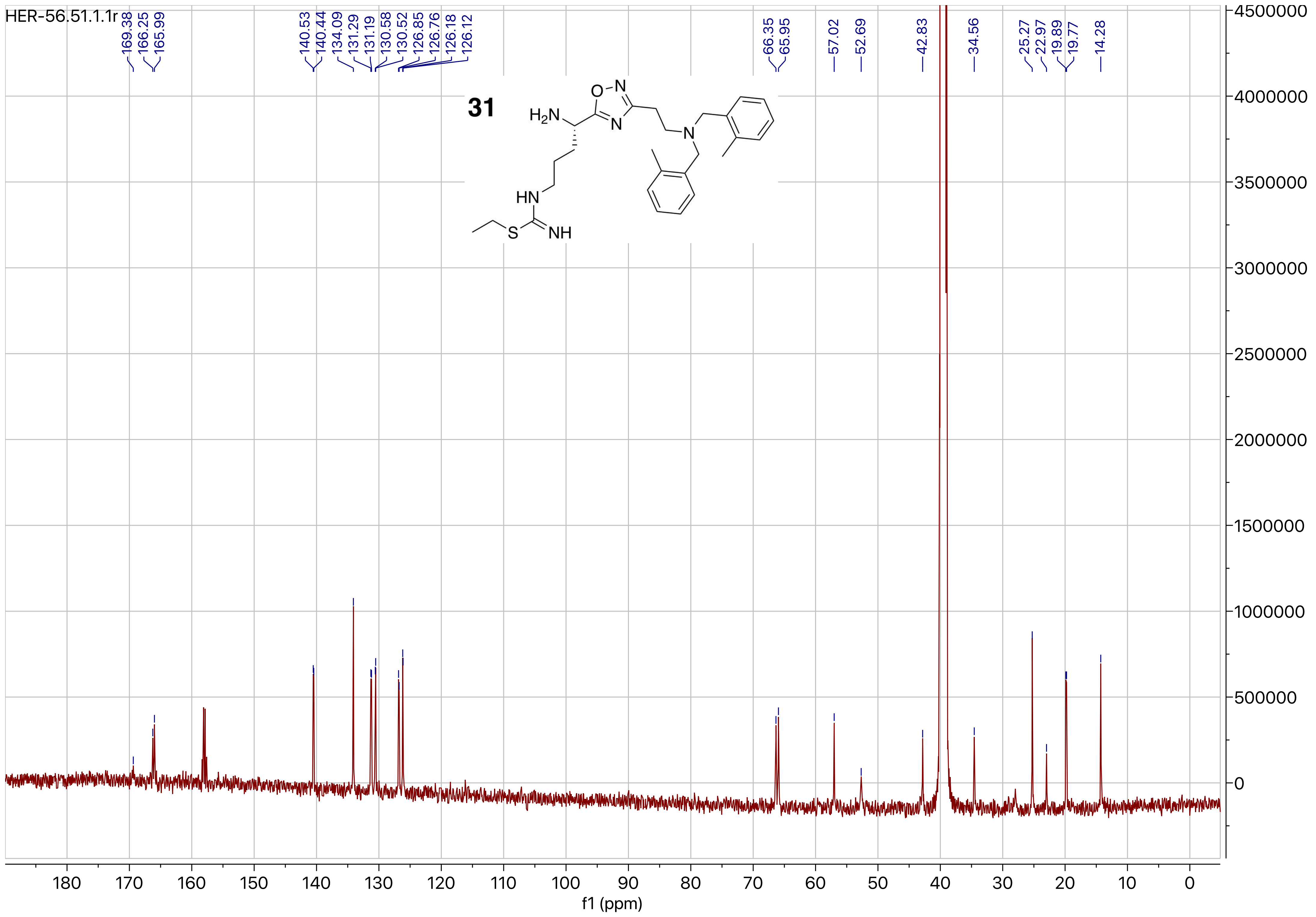

HER-56.51.1.1r
169.38
166.25
165.99
140.53
140.44
134.09
131.29
131.19
130.58
130.52
126.85
126.76
126.18
126.12
66.35
65.95
57.02
52.69
42.83
34.56
25.27
22.97
19.89
19.77
14.28
31
H2N
O
N
N
N
HN
S
NH
4500000
4000000
3500000
3000000
2500000
2000000
1500000
1000000
500000
0
180
170
160
150
140
130
120
110
100
90
80
70
60
50
40
30
20
10
0
f1 (ppm)

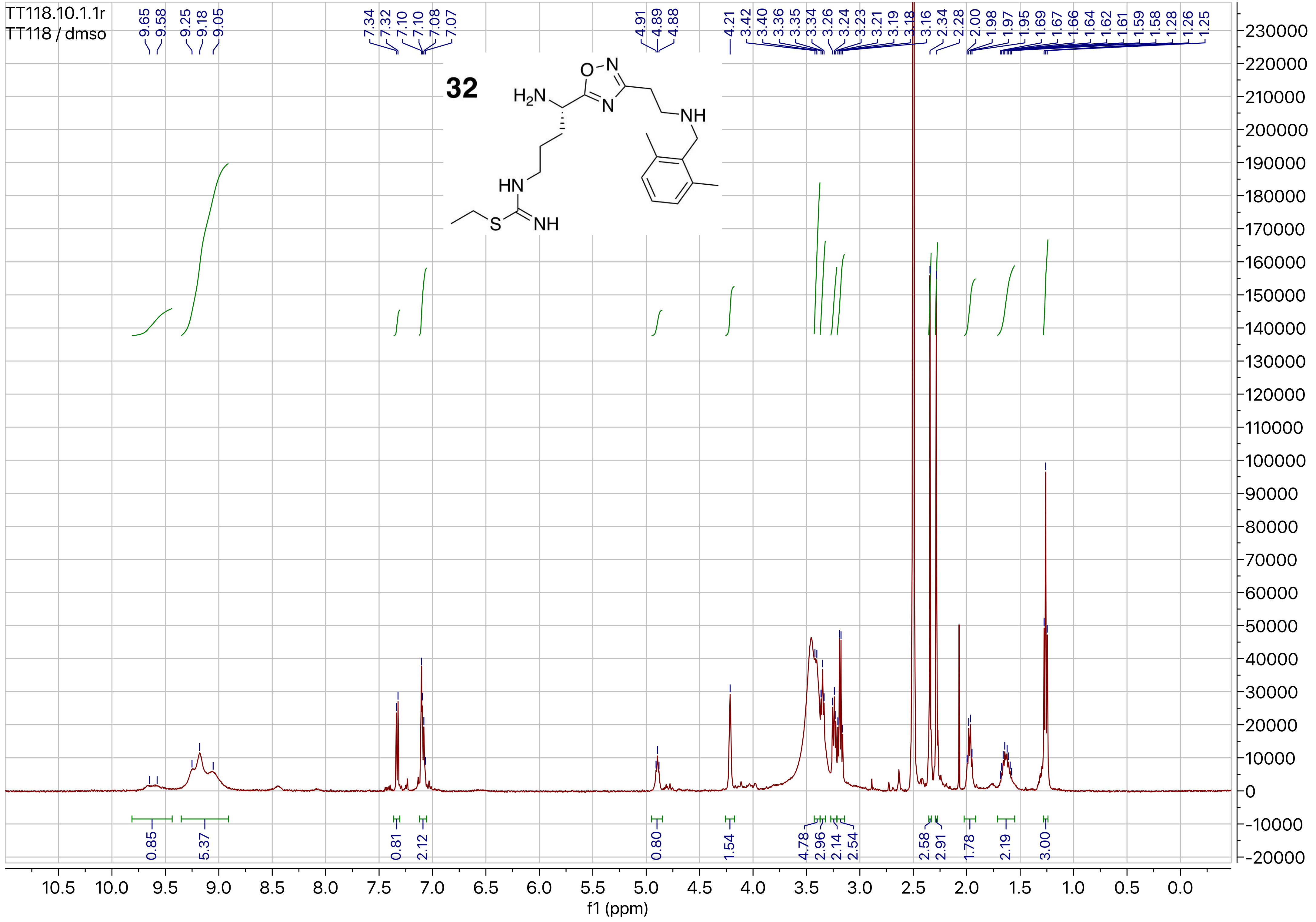
TT118.10.1.1r
TT118 / dmso
9.65 9.58 9.25 9.18 9.05
7.34 7.32 7.10 7.10 7.08 7.07
4.91 4.89 4.88
4.21 3.42 3.40 3.36 3.35 3.34 3.26 3.24 3.23 3.21 3.19 3.18 3.16 2.34 2.28 2.00 1.98 1.97 1.95 1.69 1.67 1.66 1.64 1.62 1.61 1.59 1.58 1.28 1.26 1.25
32
H_2N
O
N
N
NH
HN
S
NH
0.85 5.37 0.81 2.12 0.80 1.54 4.78 2.96 2.14 2.54 2.58 2.91 1.78 2.19 3.00
10.5 10.0 9.5 9.0 8.5 8.0 7.5 7.0 6.5 6.0 5.5 5.0 4.5 4.0 3.5 3.0 2.5 2.0 1.5 1.0 0.5 0.0
f1 (ppm)
230000 220000 210000 200000 190000 180000 170000 160000 150000 140000 130000 120000 110000 100000 90000 80000 70000 60000 50000 40000 30000 20000 10000 0 -10000 -20000

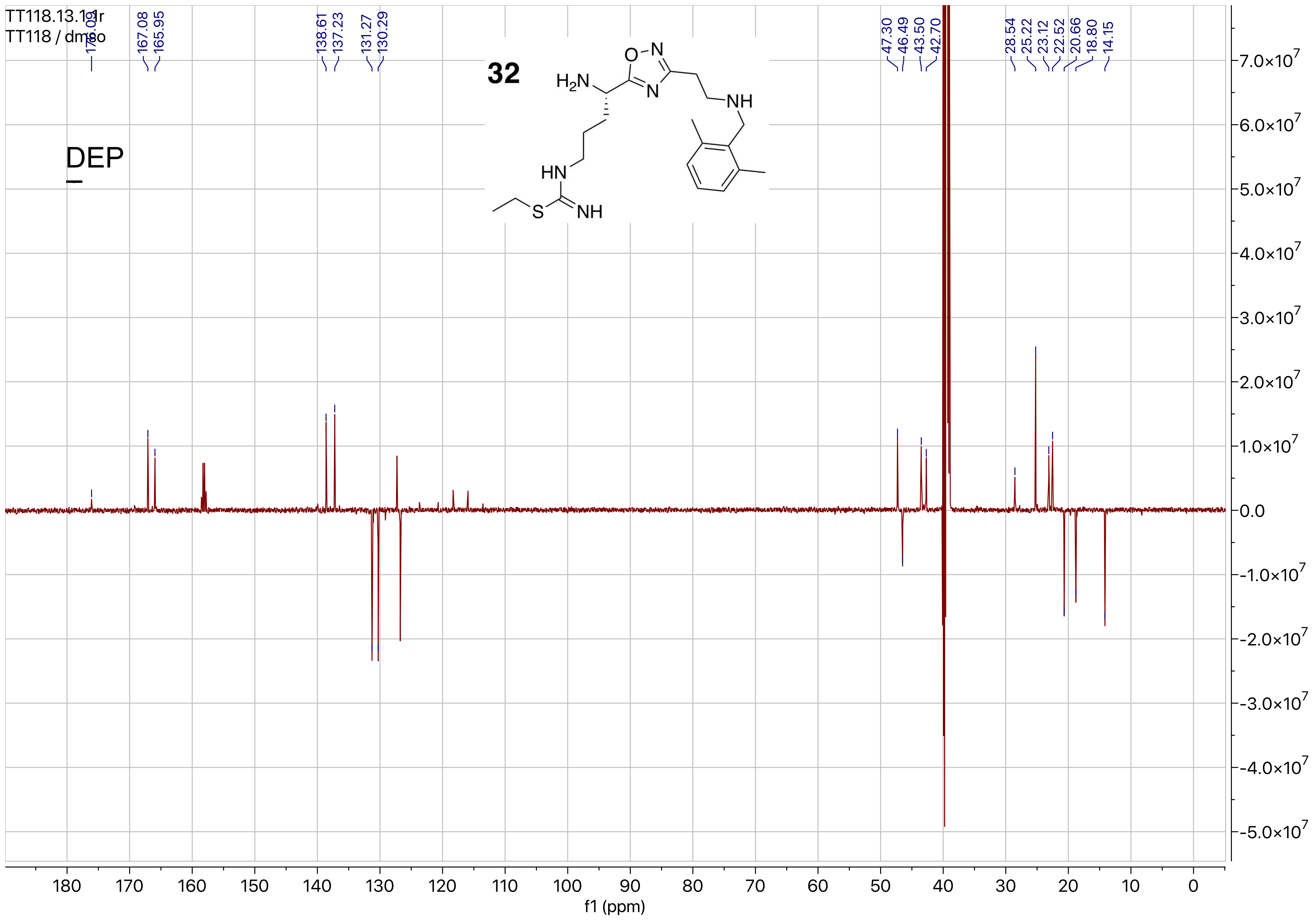

TT118 / dmso
DEP
32
167.08
165.95
138.61
137.23
131.27
130.29
47.30
46.49
43.50
42.70
28.54
25.22
23.12
22.52
20.66
18.80
14.15
f1 (ppm)

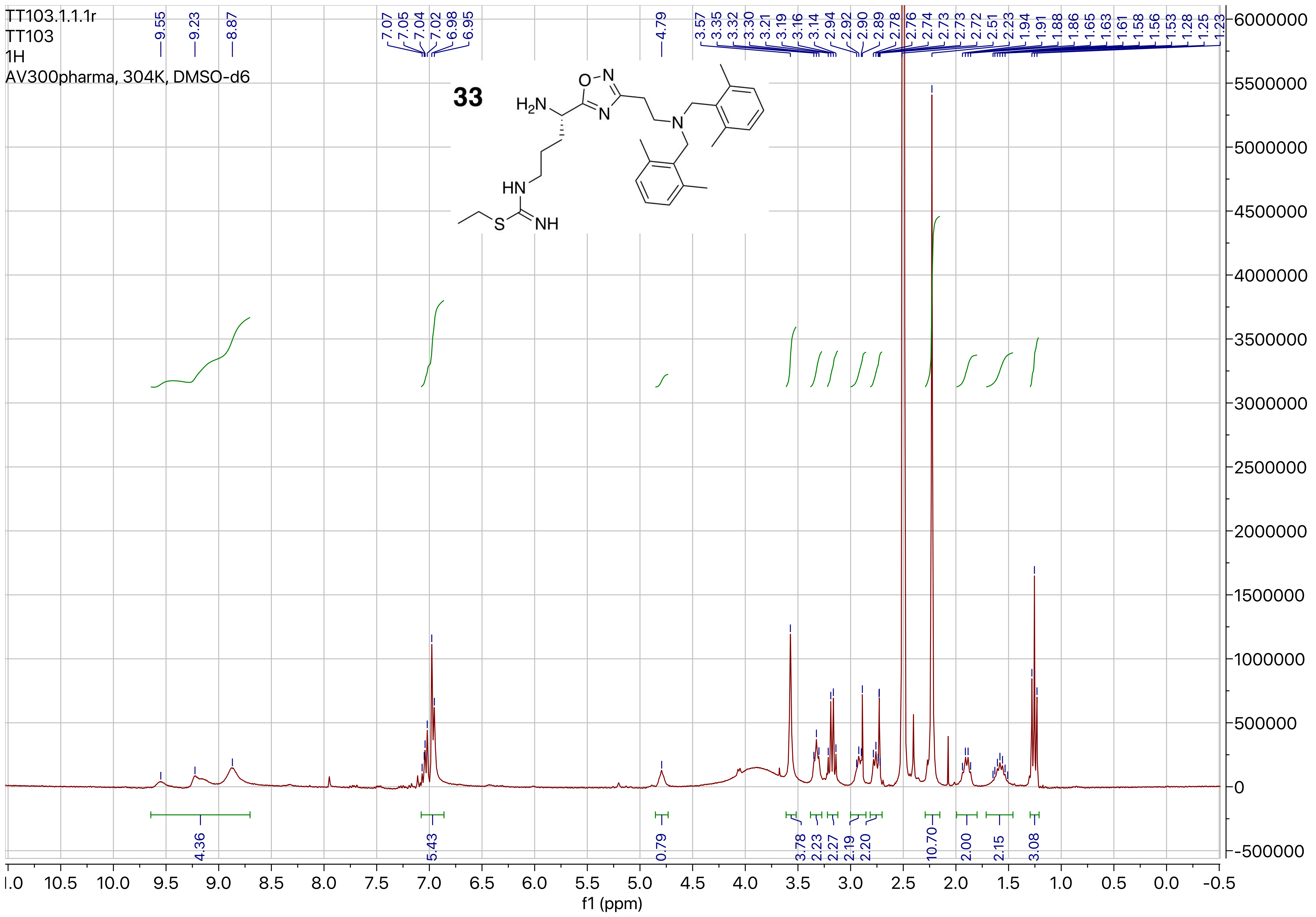
TT103.1.1.1r
TT103
1H
AV300pharma, 304K, DMSO-d6
33
H2N
O–N
N
N
HN
S
NH
9.55
9.23
8.87
7.07
7.05
7.04
7.02
6.98
6.95
4.79
3.57
3.35
3.32
3.30
3.21
3.19
3.16
3.14
2.94
2.92
2.90
2.89
2.78
2.76
2.74
2.73
2.73
2.72
2.51
2.23
1.94
1.91
1.88
1.86
1.65
1.63
1.61
1.58
1.56
1.53
1.28
1.25
1.23
4.36
5.43
0.79
3.78
2.23
2.27
2.19
2.20
10.70
2.00
2.15
3.08
f1 (ppm)

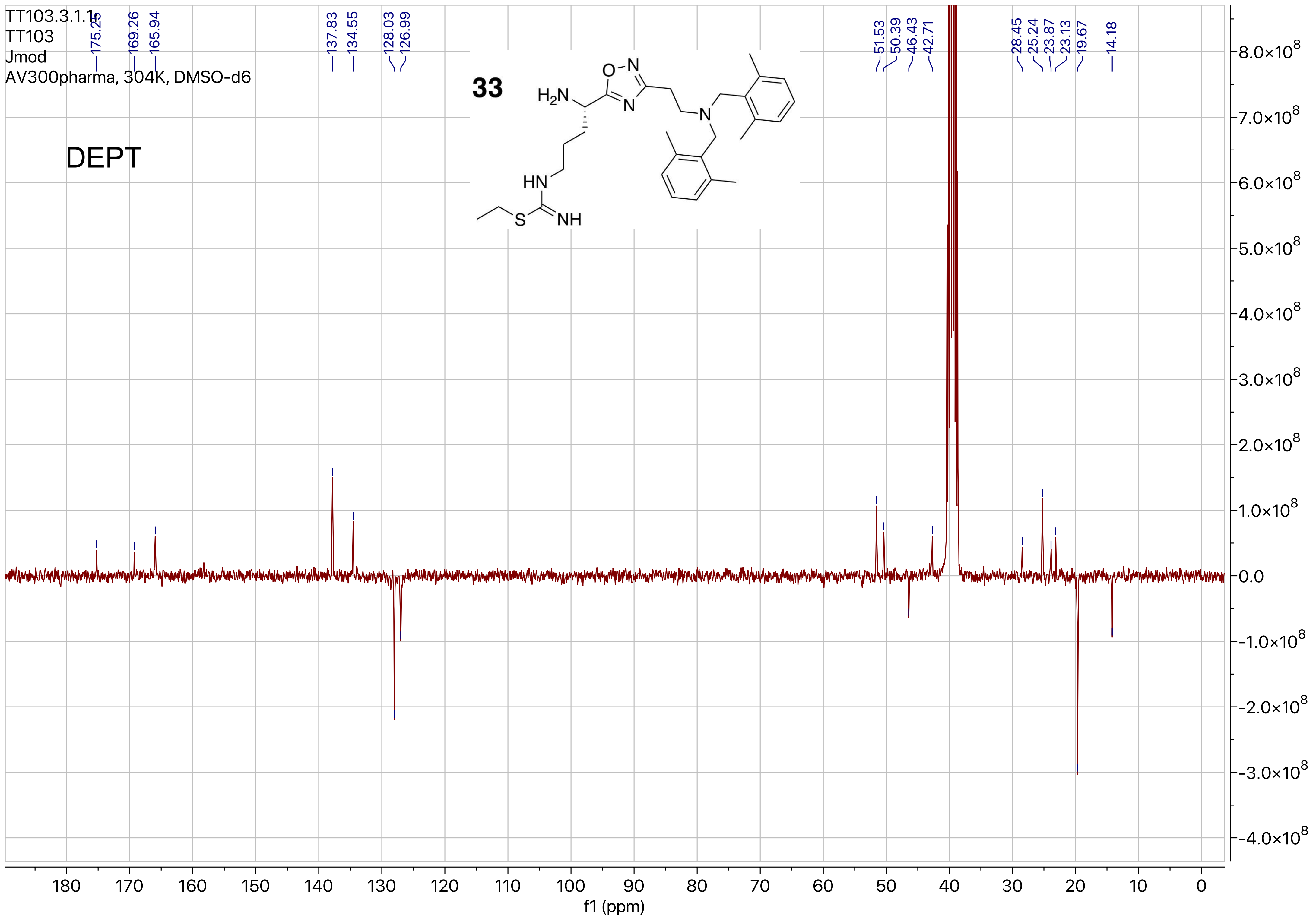
TT103.3.1.1
TT103
Jmod
AV300pharma, 304K, DMSO-d6
DEPT
33
175.25
169.26
165.94
137.83
134.55
128.03
126.99
51.53
50.39
46.43
42.71
28.45
25.24
23.87
23.13
19.67
14.18
f1 (ppm)

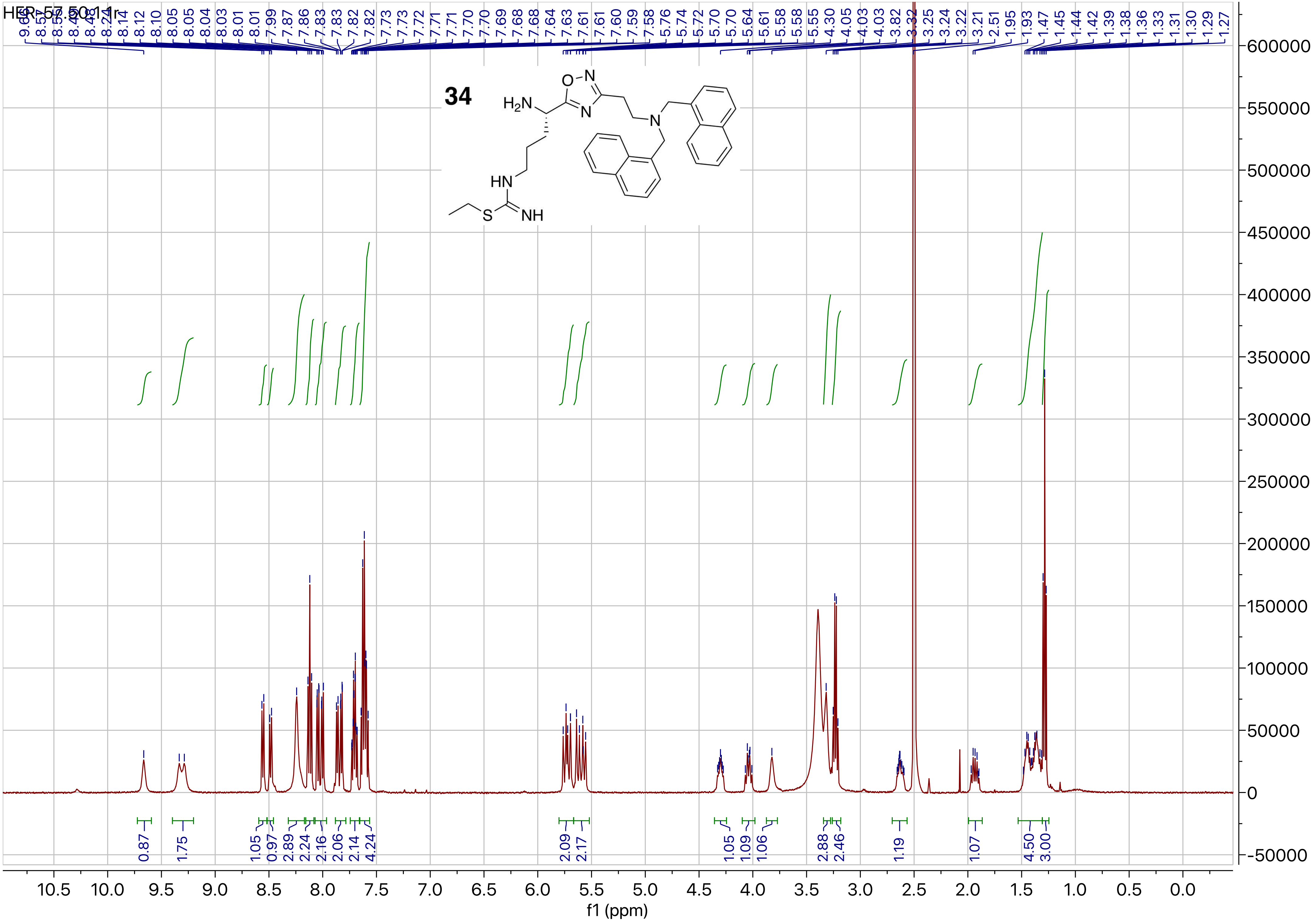
HER-57.50.1.1r
34
H2N
O-N
N
N
HN
S
NH
0.87
1.75
1.05
0.97
2.89
2.24
2.16
2.06
2.14
4.24
2.09
2.17
1.05
1.09
1.06
2.88
2.46
1.19
1.07
4.50
3.00
f1 (ppm)

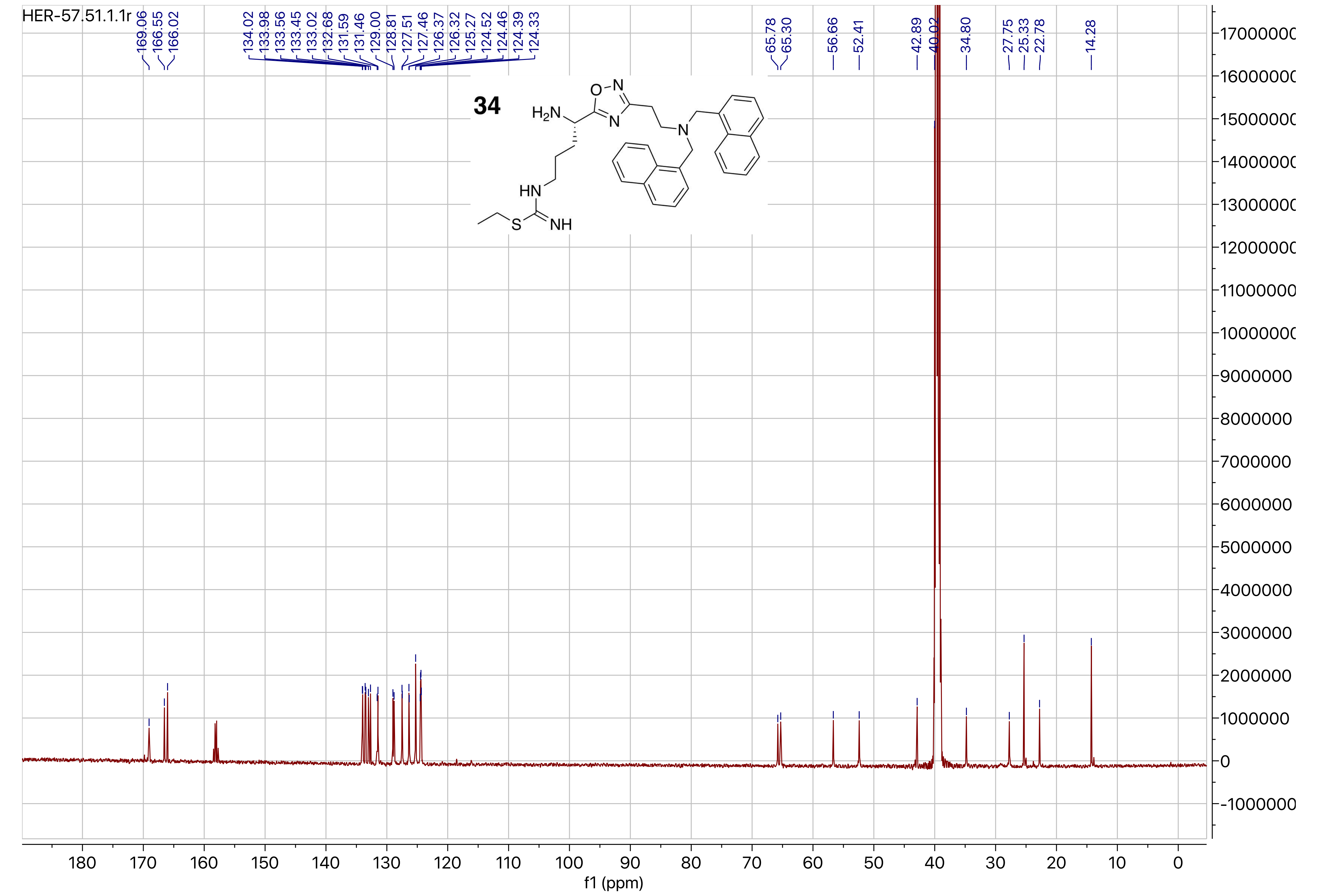
HER-57.51.1.1r
169.06
166.55
166.02
134.02
133.98
133.56
133.45
133.02
132.68
131.59
131.46
129.00
128.81
127.51
127.46
126.37
126.32
125.27
124.52
124.46
124.39
124.33
65.78
65.30
56.66
52.41
42.89
40.02
34.80
27.75
25.33
22.78
14.28
34
H2N
O
N
N
N
HN
S
NH
17000000
16000000
15000000
14000000
13000000
12000000
11000000
10000000
9000000
8000000
7000000
6000000
5000000
4000000
3000000
2000000
1000000
0
-1000000
180
170
160
150
140
130
120
110
100
90
80
70
60
50
40
30
20
10
0
f1 (ppm)

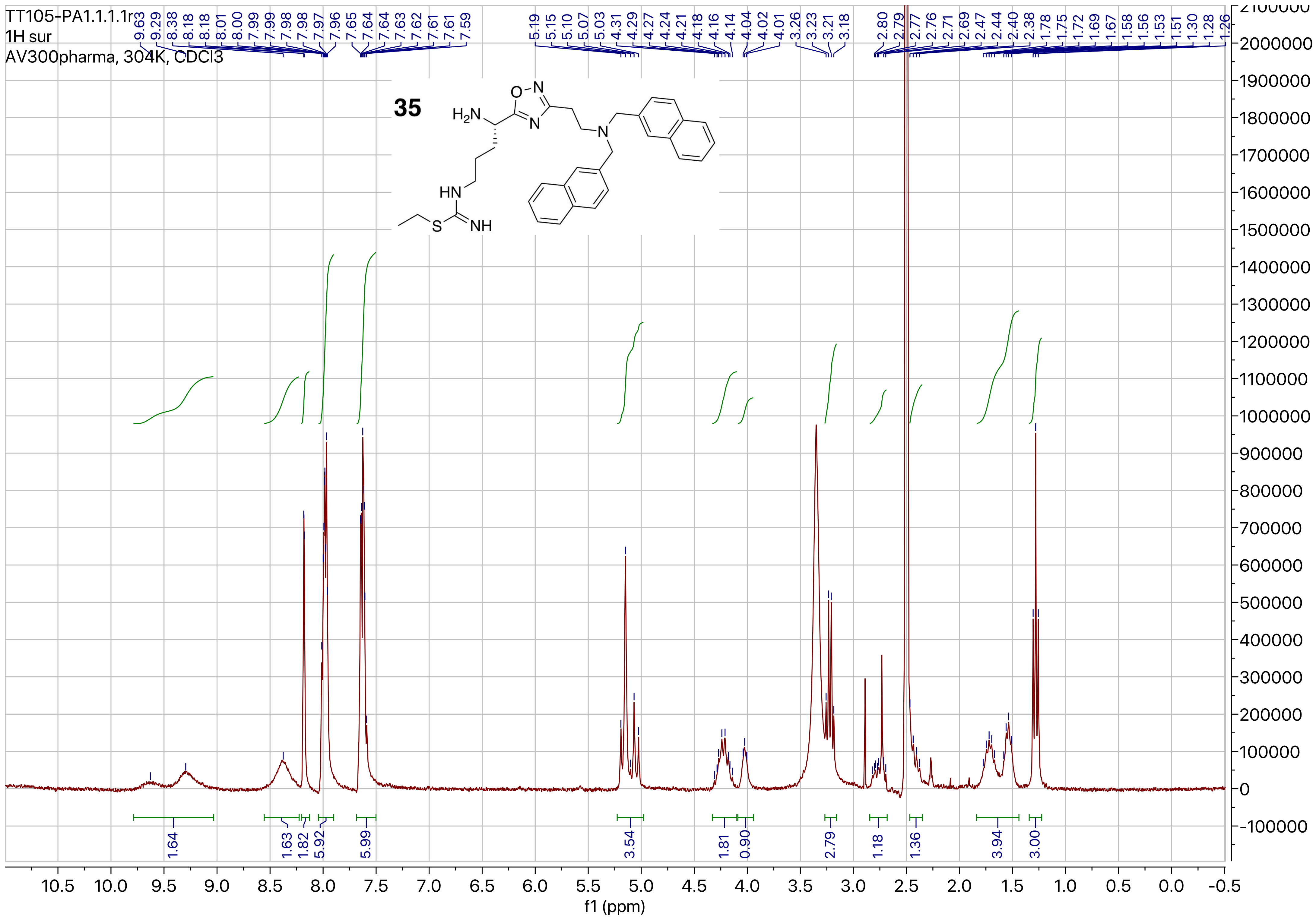
TT105-PA1.1.1.1r
1H sur
AV300pharma, 304K, CDCl3
9.63 9.29 8.38 8.18 8.18 8.01 8.00 7.99 7.99 7.98 7.98 7.97 7.96 7.65 7.64 7.64 7.63 7.62 7.61 7.61 7.59
5.19 5.15 5.10 5.07 5.03 4.31 4.29 4.27 4.24 4.21 4.18 4.16 4.14 4.04 4.02 4.01 3.26 3.23 3.21 3.18
2.80 2.79 2.77 2.76 2.71 2.69 2.47 2.44 2.40 2.38 1.78 1.75 1.72 1.69 1.67 1.58 1.56 1.53 1.51 1.30 1.28 1.26
35
H2N
O–N
N
N
HN
S
NH
1.64 1.63 1.82 5.92 5.99 3.54 1.81 0.90 2.79 1.18 1.36 3.94 3.00
f1 (ppm)

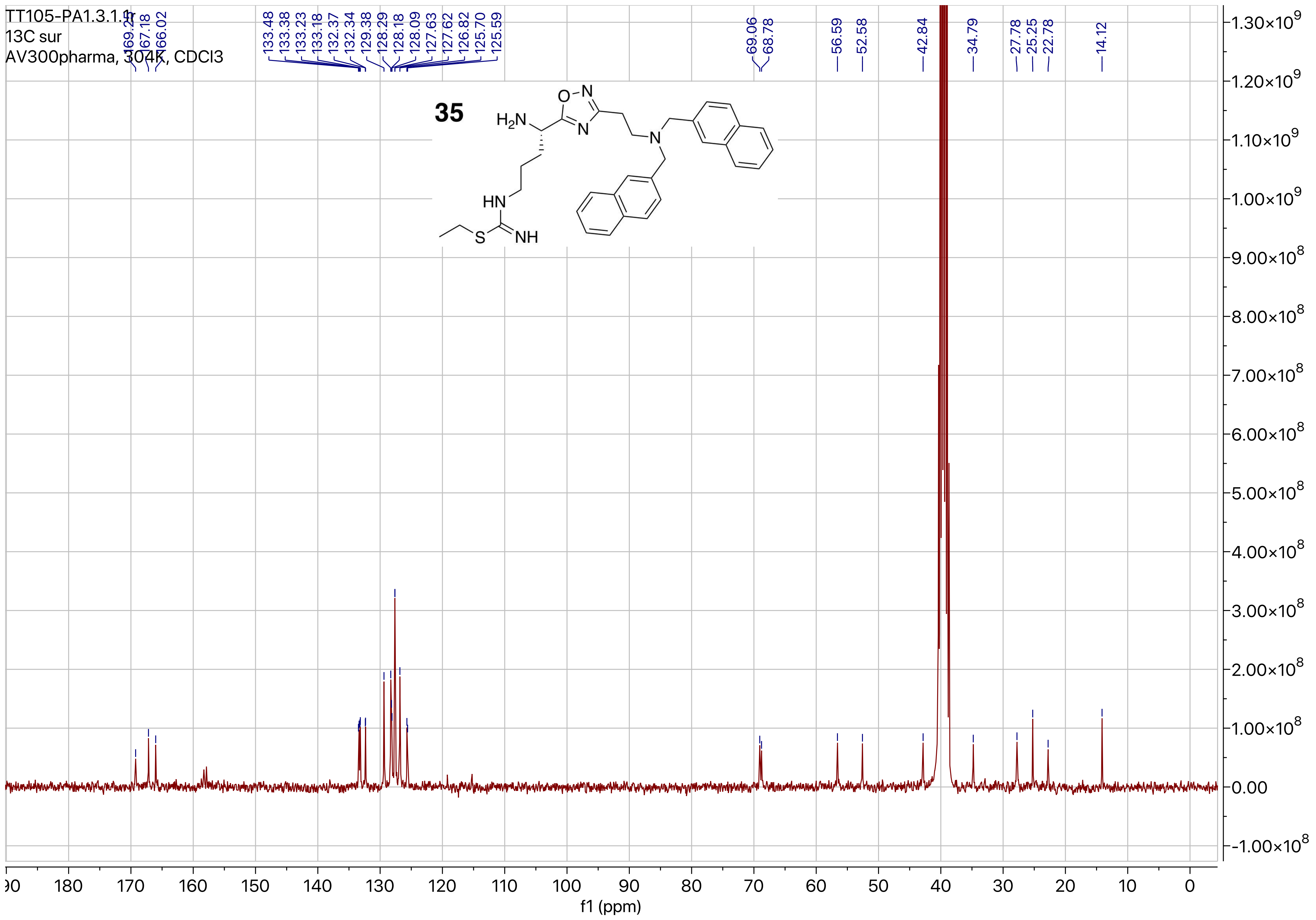

TT105-PA1.3.1.1r
13C sur
AV300pharma, 304K, CDCl3
169.2
167.18
166.02
133.48
133.38
133.23
133.18
132.37
132.34
129.38
128.29
128.18
128.09
127.63
127.62
126.82
125.70
125.59
69.06
68.78
56.59
52.58
42.84
34.79
27.78
25.25
22.78
14.12
35
O
N
N
H_2N
N
HN
S
NH
1.30×10^9
1.20×10^9
1.10×10^9
1.00×10^9
9.00×10^8
8.00×10^8
7.00×10^8
6.00×10^8
5.00×10^8
4.00×10^8
3.00×10^8
2.00×10^8
1.00×10^8
0.00
-1.00×10^8
180
170
160
150
140
130
120
110
100
90
80
70
60
50
40
30
20
10
0
f1 (ppm)

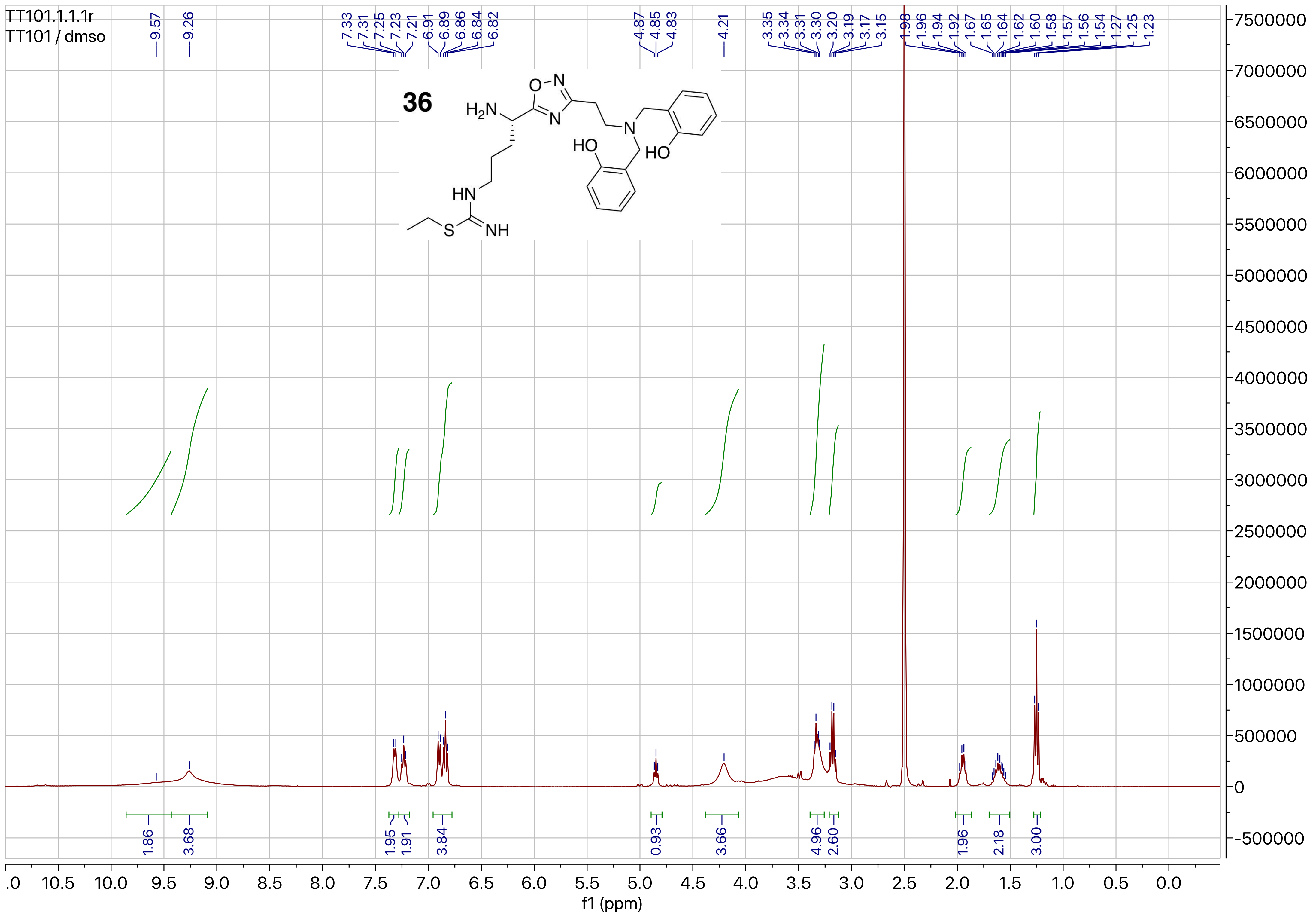

TT101.1.1.1r
TT101 / dmso
9.57
9.26
7.33
7.31
7.25
7.23
7.21
6.91
6.89
6.86
6.84
6.82
4.87
4.85
4.83
4.21
3.35
3.34
3.31
3.30
3.20
3.19
3.17
3.15
1.98
1.96
1.94
1.92
1.67
1.65
1.64
1.62
1.60
1.58
1.57
1.56
1.54
1.27
1.25
1.23
36
H_2N
O
N
N
N
HO
HO
HN
S
NH
1.86
3.68
1.95
1.91
3.84
0.93
3.66
4.96
2.60
1.96
2.18
3.00
7500000
7000000
6500000
6000000
5500000
5000000
4500000
4000000
3500000
3000000
2500000
2000000
1500000
1000000
500000
0
-500000
.0
10.5
10.0
9.5
9.0
8.5
8.0
7.5
7.0
6.5
6.0
5.5
5.0
4.5
4.0
3.5
3.0
2.5
2.0
1.5
1.0
0.5
0.0
f1 (ppm)

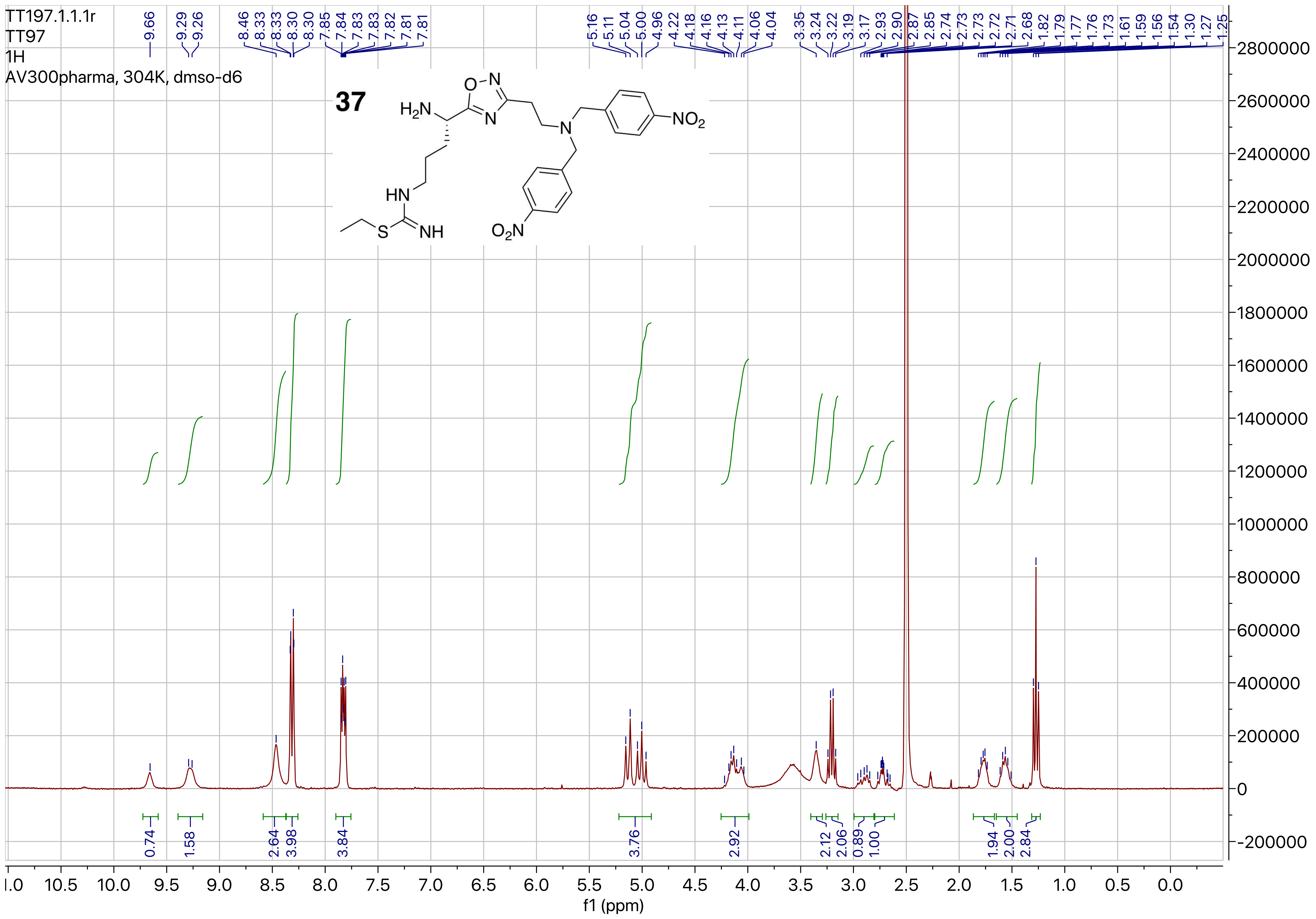

TT197.1.1.1r
TT97
1H
AV300pharma, 304K, dmso-d6
9.66
9.29
9.26
8.46
8.33
8.33
8.30
8.30
7.85
7.84
7.83
7.83
7.82
7.81
7.81
5.16
5.11
5.04
5.00
4.96
4.22
4.18
4.16
4.13
4.11
4.06
4.04
3.35
3.24
3.22
3.19
3.17
2.93
2.90
2.87
2.85
2.74
2.73
2.73
2.72
2.71
2.68
1.82
1.79
1.77
1.76
1.73
1.61
1.59
1.56
1.54
1.30
1.27
1.25
37
H2N
O
N
N
N
NO2
HN
S
NH
O2N
0.74
1.58
2.64
3.98
3.84
3.76
2.92
2.12
2.06
0.89
1.00
1.94
2.00
2.84
f1 (ppm)

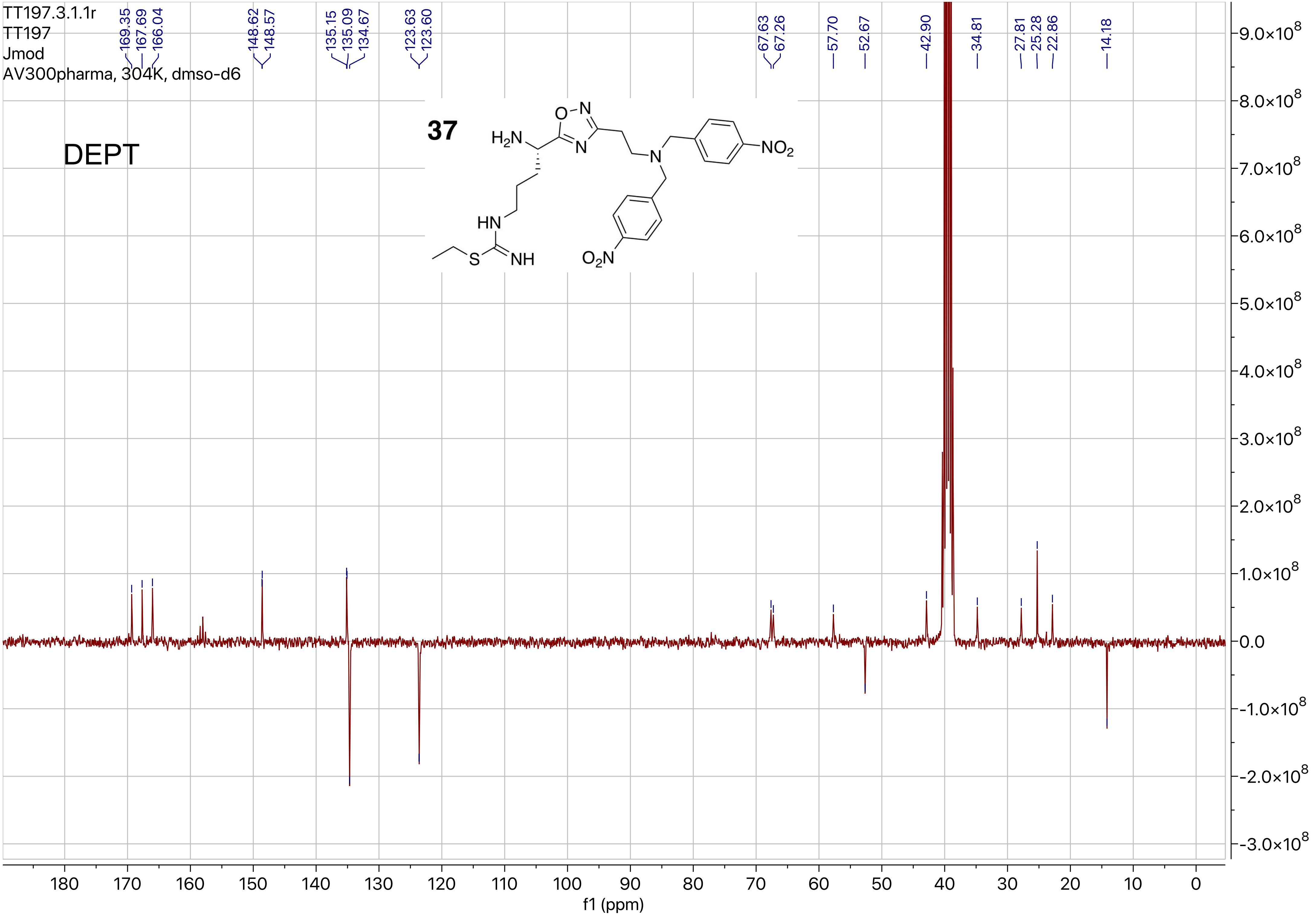
TT197.3.1.1r
TT197
Jmod
AV300pharma, 304K, dmso-d6
DEPT
169.35
167.69
166.04
148.62
148.57
135.15
135.09
134.67
123.63
123.60
67.63
67.26
57.70
52.67
42.90
34.81
27.81
25.28
22.86
14.18
37
H2N
O
N
N
N
NO2
HN
S
NH
O2N
f1 (ppm)

TT196.1.1.1r
TT196
1H
AV300pharma, 304K, dmso-d6

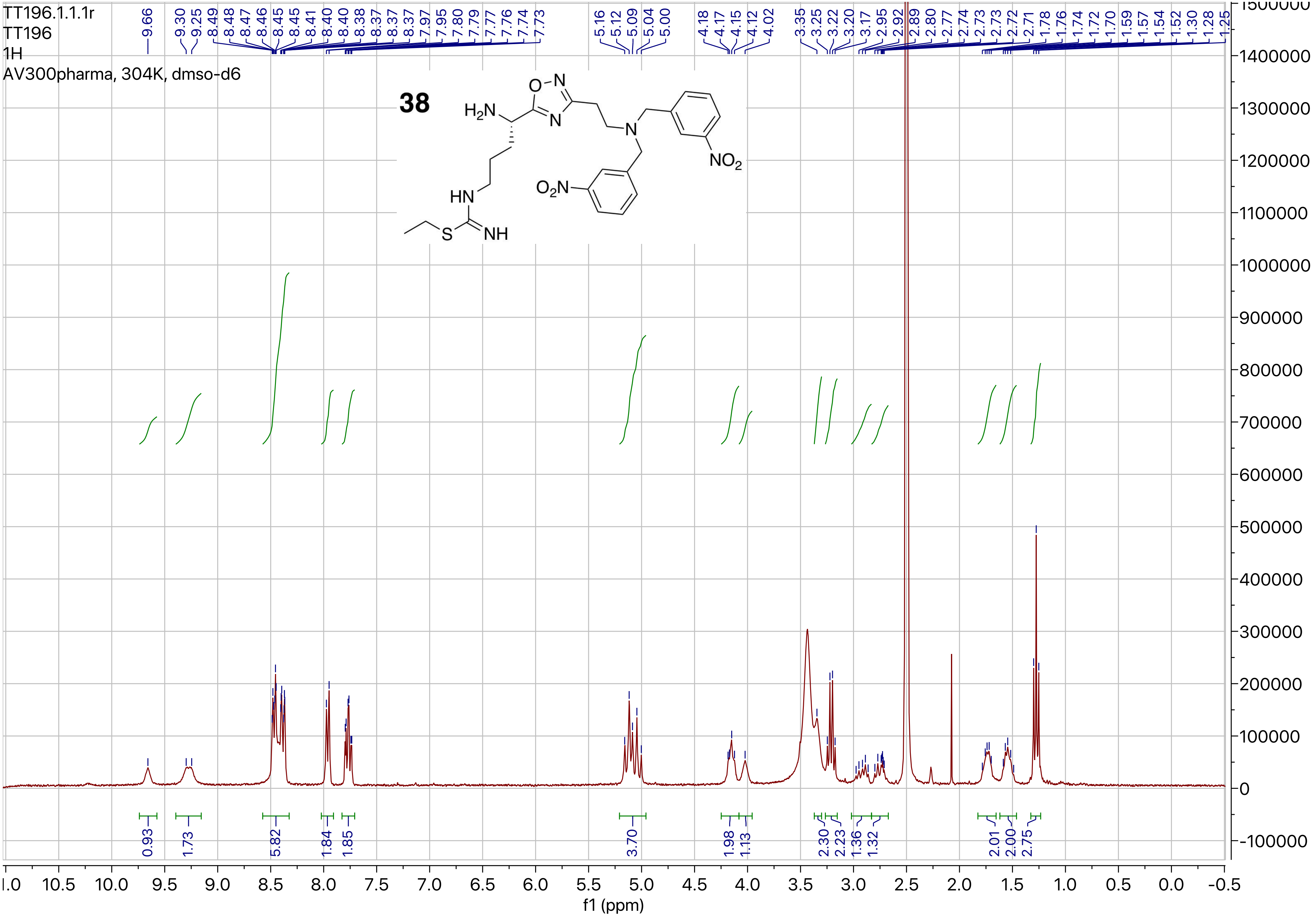

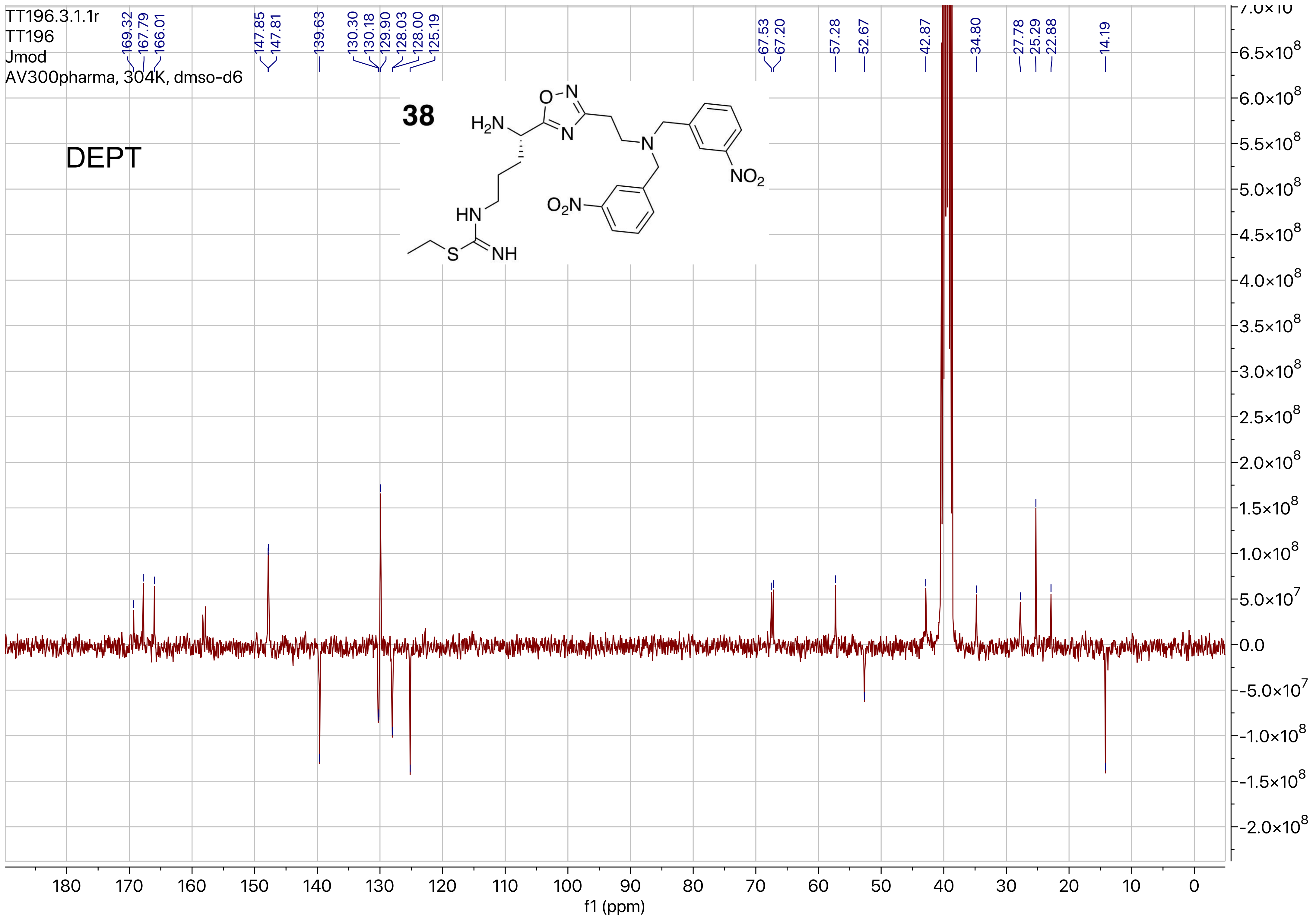
TT196.3.1.1r
TT196
Jmod
AV300pharma, 304K, dmso-d6
DEPT
38
169.32
167.79
166.01
147.85
147.81
139.63
130.30
130.18
129.90
128.03
128.00
125.19
67.53
67.20
57.28
52.67
42.87
34.80
27.78
25.29
22.88
14.19
f1 (ppm)

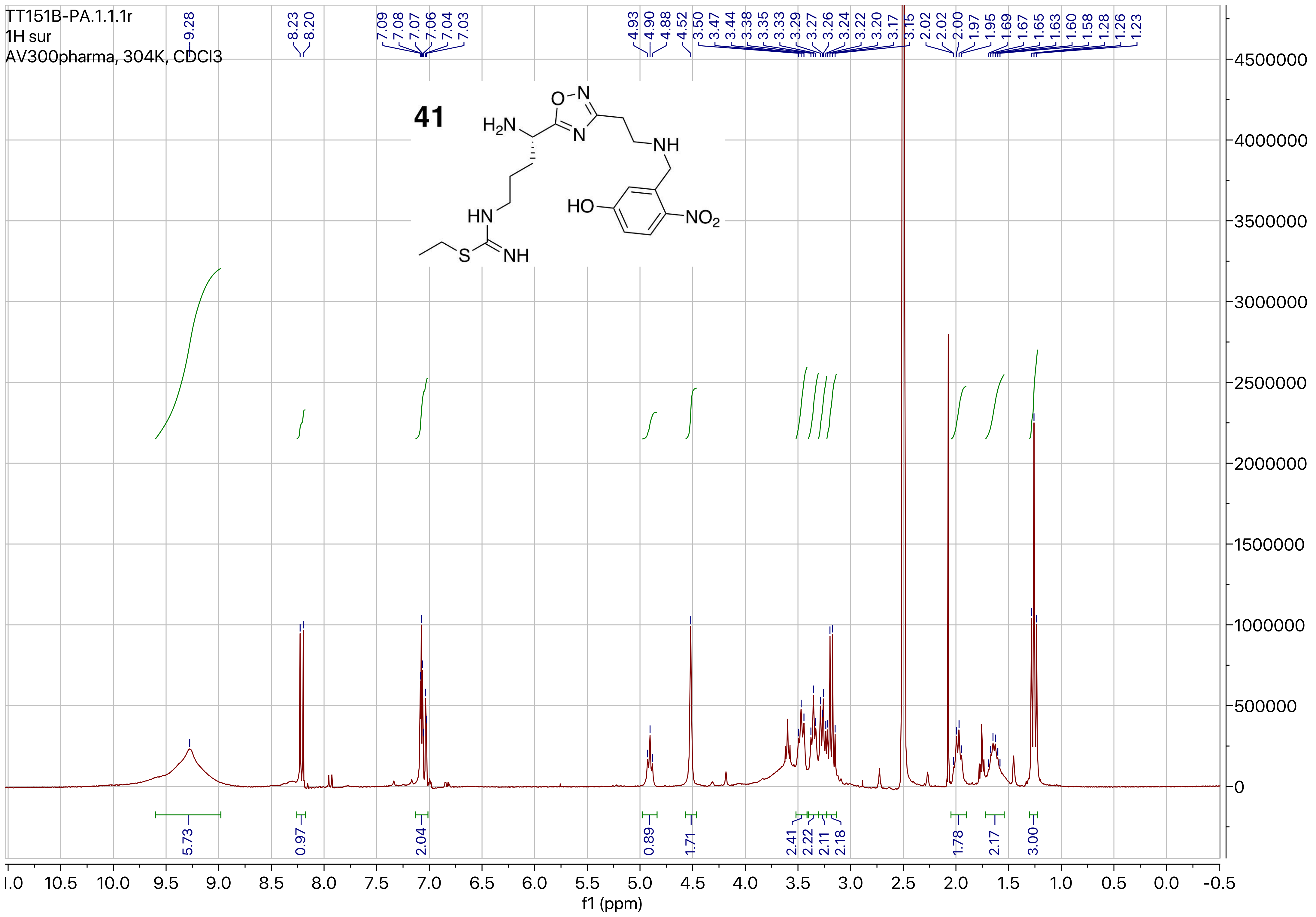
TT151B-PA.1.1.1r
1H sur
AV300pharma, 304K, CDCl3
9.28
8.23
8.20
7.09
7.08
7.07
7.06
7.04
7.03
4.93
4.90
4.88
4.52
3.50
3.47
3.44
3.38
3.35
3.33
3.29
3.27
3.26
3.24
3.22
3.20
3.17
3.15
2.02
2.02
2.00
1.97
1.95
1.69
1.67
1.65
1.63
1.60
1.58
1.28
1.26
1.23
41
H2N
O
N
N
NH
HO
NO2
HN
S
NH
5.73
0.97
2.04
0.89
1.71
2.41
2.22
2.11
2.18
1.78
2.17
3.00
4500000
4000000
3500000
3000000
2500000
2000000
1500000
1000000
500000
0
f1 (ppm)

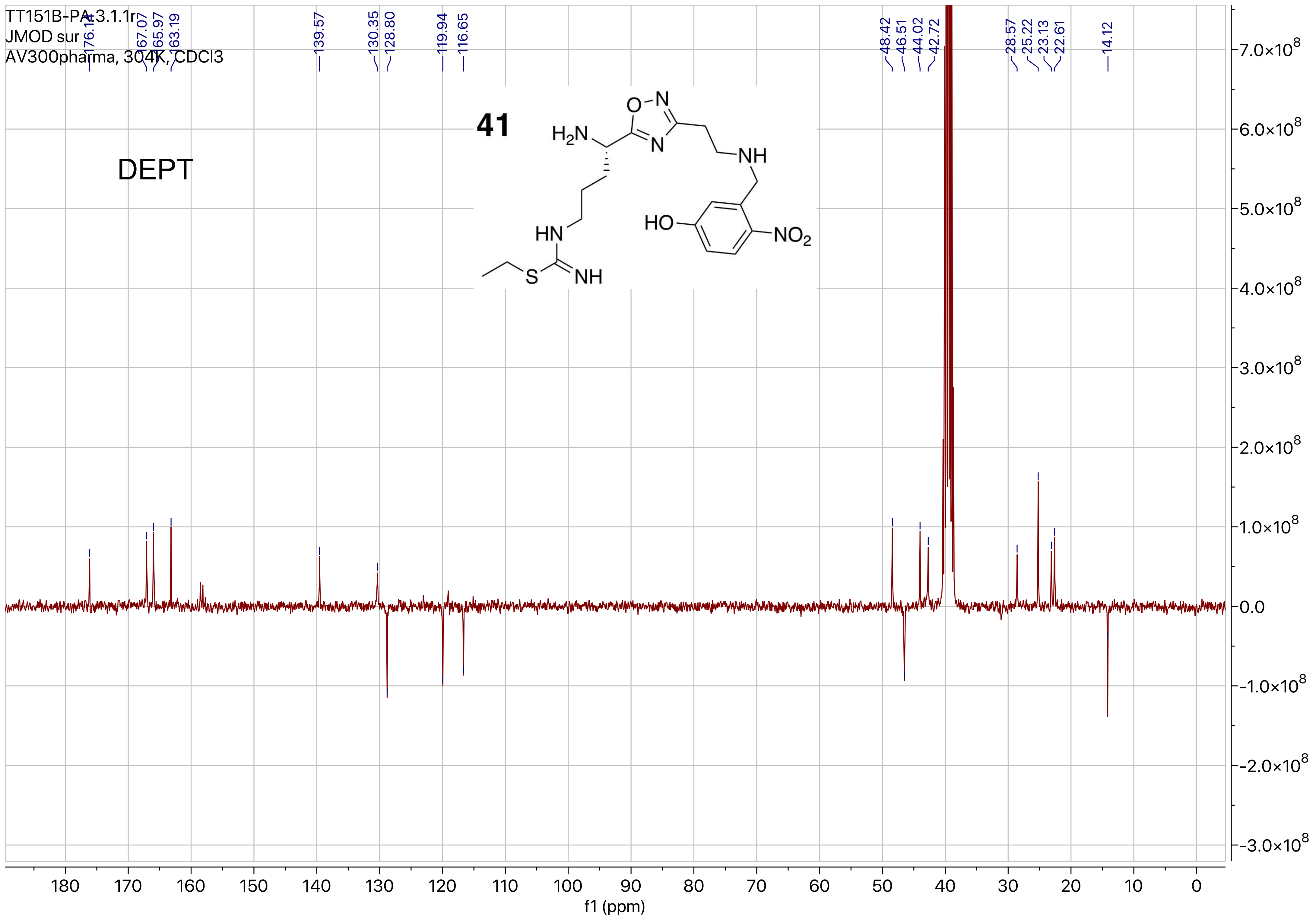
TT151B-PA.3.1.1r
JMOD sur
AV300pharma, 304K, CDCl3
176.12
167.07
165.97
163.19
139.57
130.35
128.80
119.94
116.65
48.42
46.51
44.02
42.72
28.57
25.22
23.13
22.61
14.12
DEPT
41
H_2N
O
N
N
NH
HO
NO_2
HN
S
NH
7.0×10^8
6.0×10^8
5.0×10^8
4.0×10^8
3.0×10^8
2.0×10^8
1.0×10^8
0.0
-1.0×10^8
-2.0×10^8
-3.0×10^8
180
170
160
150
140
130
120
110
100
90
80
70
60
50
40
30
20
10
0
f1 (ppm)

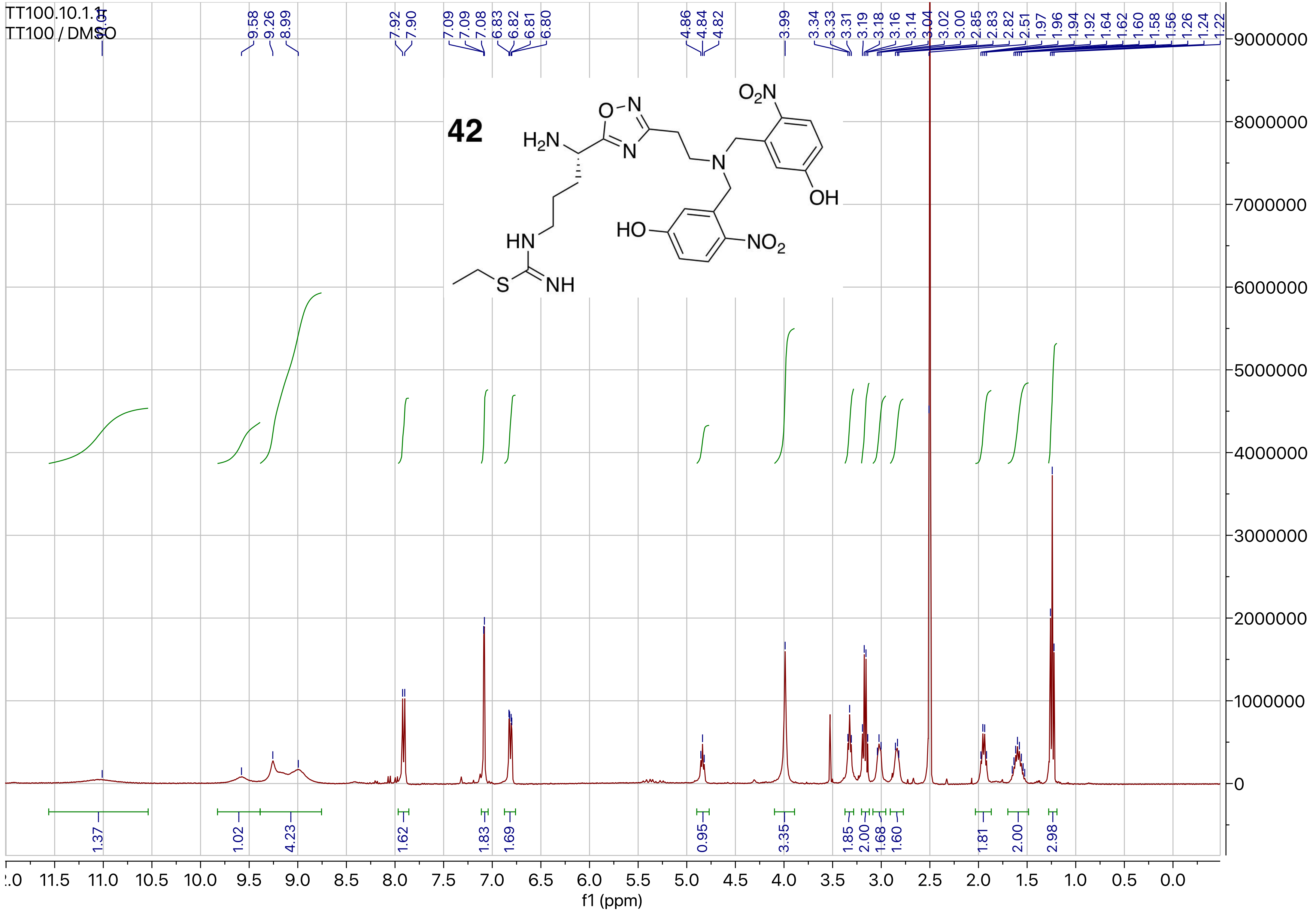
TT100.10.1.1r
TT100 / DMSO
9.58 9.26 8.99 7.92 7.90 7.09 7.09 7.08 6.83 6.82 6.81 6.80 4.86 4.84 4.82 3.99 3.34 3.33 3.31 3.19 3.18 3.16 3.14 3.04 3.02 3.00 2.85 2.83 2.82 2.51 1.97 1.96 1.94 1.92 1.64 1.62 1.60 1.58 1.56 1.26 1.24 1.22
42
H_2N
O−N
N
O_2N
N
OH
HN
HO
NO_2
S
NH
1.37 1.02 4.23 1.62 1.83 1.69 0.95 3.35 1.85 2.00 1.68 1.60 1.81 2.00 2.98
11.5 11.0 10.5 10.0 9.5 9.0 8.5 8.0 7.5 7.0 6.5 6.0 5.5 5.0 4.5 4.0 3.5 3.0 2.5 2.0 1.5 1.0 0.5 0.0
f1 (ppm)
9000000 8000000 7000000 6000000 5000000 4000000 3000000 2000000 1000000 0

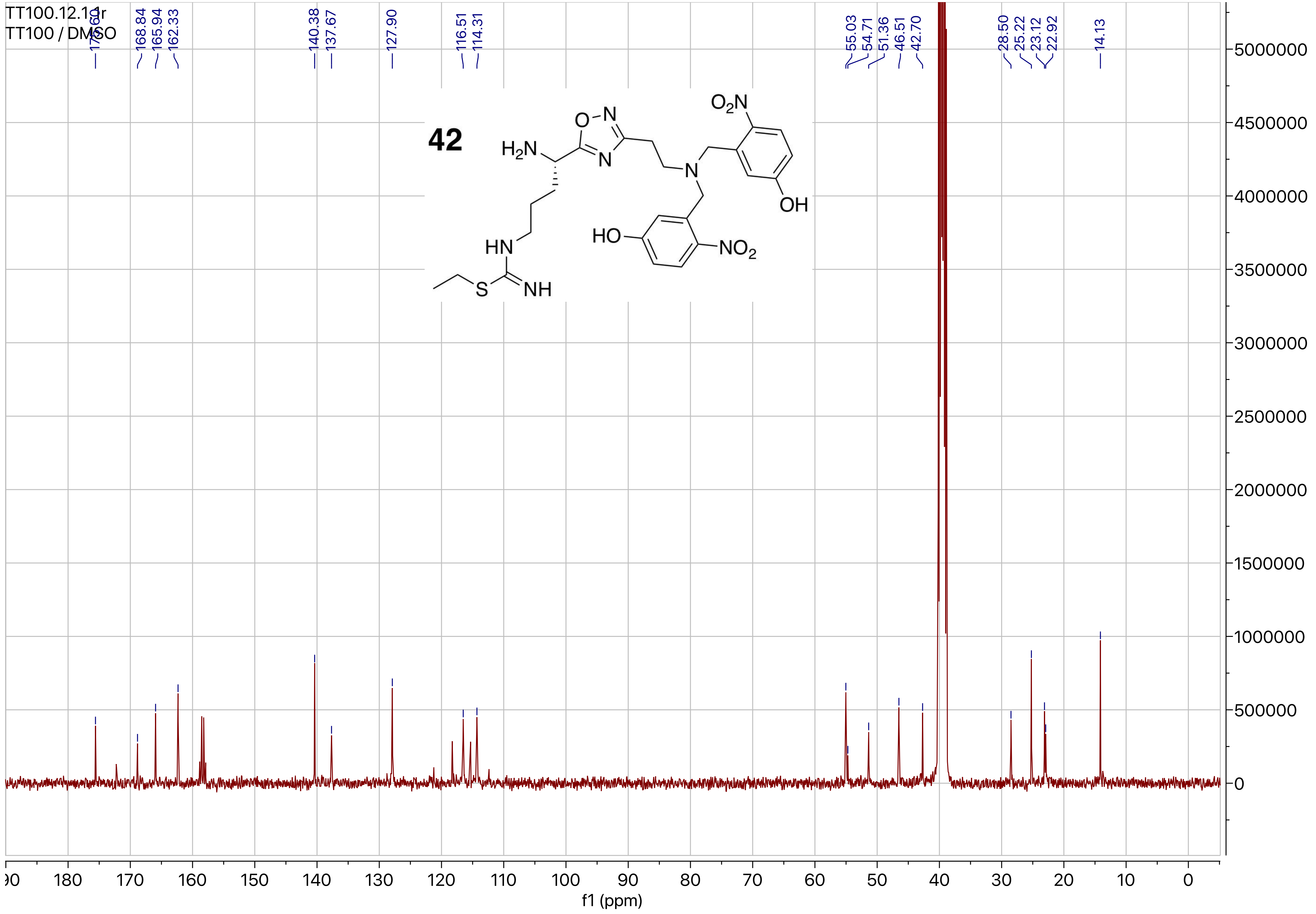
TT100.12.11r
TT100 / DMSO
168.84
165.94
162.33
140.38
137.67
127.90
116.51
114.31
55.03
54.71
51.36
46.51
42.70
28.50
25.22
23.12
22.92
14.13
42
O
N
H2N
N
N
O2N
OH
HO
NO2
HN
S
NH
5000000
4500000
4000000
3500000
3000000
2500000
2000000
1500000
1000000
500000
0
180
170
160
150
140
130
120
110
100
90
80
70
60
50
40
30
20
10
0
f1 (ppm)
